# Interaction-Specific Changes in the Transcriptome of *Polynucleobacter asymbioticus* Caused by Varying Protistan Communities

**DOI:** 10.3389/fmicb.2019.01498

**Published:** 2019-07-09

**Authors:** Daniela Beisser, Christina Bock, Martin W. Hahn, Matthijs Vos, Bernd Sures, Sven Rahmann, Jens Boenigk

**Affiliations:** ^1^Biodiversity, University of Duisburg-Essen, Essen, Germany; ^2^Research Institute for Limnology, University of Innsbruck, Mondsee, Austria; ^3^Theoretical and Applied Biodiversity, Ruhr-University Bochum, Bochum, Germany; ^4^Aquatic Ecology, University of Duisburg-Essen, Essen, Germany; ^5^Centre for Water and Environmental Research (ZWU), University of Duisburg-Essen, Essen, Germany; ^6^Genome Informatics, University of Duisburg-Essen, Essen, Germany

**Keywords:** transcriptomics, *Polynucleobacter asymbioticus*, chrysophyceae, predator-prey, trophic modes

## Abstract

We studied the impact of protist grazing and exudation on the growth and transcriptomic response of the prokaryotic prey species *Polynucleobacter asymbioticus*. Different single- and multi-species communities of chrysophytes were used to determine a species-specific response to the predators and the effect of chrysophyte diversity. We sequenced the mRNA of *Pn. asymbioticus* in communities with three single chrysophyte species (*Chlorochromonas danica, Poterioochromonas malhamensis* and *Poteriospumella lacustris*) and all combinations. The molecular responses of *Pn. asymbioticus* significantly changed in the presence of predators with different trophic modes and combinations of species. In the single-species samples we observed significant differences related to the relative importance of grazing and exudation in the protist-bacteria interaction, i.e., to the presence of either the heterotrophic *Ps. lacustris* or the mixotrophic *C. danica*. When grazing dominates the interaction, as in the presence of *Ps. lacustris*, genes acting in stress response are up-regulated. Further genes associated with transcription and translation are down-regulated indicating a reduced growth of *Pn. asymbioticus*. In contrast, when the potential use of algal exudates dominates the interaction, genes affiliated with iron transport are up-regulated. Rapid phototrophic growth of chrysophytes, with a high demand on soluble iron, could thus lead to iron-limitation and cause changes in the iron metabolism of *Pn. asymbioticus*. Additionally, we observe a benefit for *Pn. asymbioticus* from a more diverse protistan community, which could be due to shifts in the relative importance of phototrophy in the mixotrophic chrysophytes when competing for food with other species. Our study highlights the importance of biotic interactions and the specificity of such interactions, in particular the differential effect of grazing and algal exudation in the interaction of bacteria with mixotrophic protists.

## 1. Introduction

Many ecological processes and patterns in aquatic systems such as competition, community structure, and nutrient flow are influenced by predation and modulated by interactions between aquatic microorganisms. Heterotrophic and mixotrophic chrysomonads are abundant members of freshwater communities (Carrick and Fahnenstiel, 1989; Carrias et al., 1998; Auer and Arndt, 2001; Boenigk and Arndt, 2002; Weitere and Arndt, 2003) and by grazing on bacteria, function as a link between microbial secondary production and higher trophic levels. Among their prey organisms are ultramicrobacteria from the genus *Polynucleobacter*. Free-living strains of Polynucleobacter of *Polynucleobacter* possess a wide distribution in freshwater habitats, identified from a broad variety of habitats located in all climatic zones and distinguished by various chemical conditions (Hahn et al., 2003; Percent et al., 2008; Jezberová et al., 2010; Newton et al., 2011). Thus, this genus comprises an important part of the freshwater bacterioplankton. The isolation and characterization of a large number of isolates associated with the genus *Polynucleobacter* has provided extensive knowledge into preferred substrates, temperature and pH ranges and the ability to grow anaerobically (Hahn et al., 2009, 2010). The genus *Polynucleobacter* is described as being chemo-organotrophic and aerobic, with some strains being capable of facultative anaerobic growth. It is ubiquitous in a very broad range lake systems including alkaline, acidic, oligotrophic high mountain and eutrophic shallow lakes, rivers, ponds and raised bogs (Hahn et al., 2009; Jezberová et al., 2010; Jezbera et al., 2011 and within). By contrast, the species *Pn. asymbioticus* is adapted to slightly acidic conditions and dwells in shallow ponds (Hahn et al., 2012, 2016b).

From grazing experiments with single predator species, it is known that chrysophytes feed on small ultramicrobacteria but prefer larger bacteria (Boenigk et al., 2006; Foster and Chrzanowski, 2012). Predation on *Polynucleobacter* strains by chrysophytes was investigated in detail and edibility was demonstrated for all investigated strains (Boenigk et al., 2004). Vulnerability to predation was found to depend on the size ratio between the chrysophyte predator and the *Polynucleobacter* prey cells. The relative importance of bacterivory differs between chrysophyte species, with species being exclusively phagotrophic and predominantly phototrophic species (Schmidtke et al., 2006). In addition to direct interactions, indirect factors such as toxicity and resistance by either predator or prey, competition, avoidance or the dominant mode of nutrition, may shape interactions. For several chrysophyte species it has been shown that extracted toxins or the filtrate of flagellate cultures inhibit bacterial growth (Hansen, 1973; Boenigk, 2004; Blom and Pernthaler, 2010). This antibiotic effect may affect the growth of some aquatic bacteria and potentially affect microbial community composition. On the other hand, photosynthetically-active organisms provide resource subsidies and exchange resources with heterotrophic microorganisms (Pernthaler et al., 1997; Jezbera et al., 2006; Salcher et al., 2007; Marcarelli et al., 2011; Wyatt and Turetsky, 2015). Their dissolved exudates are rich in a great variety of organic compounds, corresponding to 20% of the carbon they fix by photosynthesis (Aaronson et al., 1971; Kristiansen and Škaloud, 2017). These compounds include carbohydrates, enzymes, and vitamins (Aaronson et al., 1971; Myklestad, 1995; Biddanda and Benner, 1997) which can be readily consumed by bacteria and stimulate bacterial respiration and growth.

To our knowledge the molecular response of bacteria to varying modes of nutrition in single protistan species and communities has not yet been investigated. Few studies exist which focus on the detection of microbial interactions between bacteria and protists, e.g., using metaomics approaches or single-cell genomics (Yoon et al., 2011; Stepanauskas, 2015; Gong et al., 2016; Krabberød et al., 2017). In a study applying transcriptomics and mass spectrometry (Song et al., 2015) observed multiple changes in the bacterium *Pseudomonas fluorescens*, especially in lipopeptide biosynthesis and accumulation, upon protozoan grazing by *Naegleria americana* in soil and rhizosphere environments. Another study by Moustafa et al. (2010) focused on the changes in the transcriptomic profile of the photosynthetic dinoflagellate *Alexandrium tamarense* in the presence of bacteria, showing significant impact on key metabolic processes such as photosynthesis.

The focus of the present study is to investigate the gene expression response of *Pn. asymbioticus* in a gradient of protist-bacteria interactions dominated by exudation to interactions dominated by grazing and increasing complexity of the predator community. Therefore, *Pn. asymbioticus* is exposed to three single flagellate species *Chlorochromonas danica, Poterioochromonas malhamensis*, and *Poteriospumella lacustris* and all combinations, exhibiting varying degrees of photo- and phagotrophy. All three species are among the best investigated model species in ecophysiological experiments [*C. danica* originally described as *Ochromonas danica* (Andersen et al., 2017), *P. malhamensis* originally published as *Ochromonas malhamensis* (Andersen et al., 2017), and *Ps. lacustris* originally published as *Spumella*-like flagellate strain JBM10 (Grossmann et al., 2016)]. These species are common in lake ecosystems, *Ps. lacustris* is a heterotrophic nanoflagellates particularly abundant during the warm season (corresponding to the C3 clade in Nolte et al., 2010 whereas *Poterioochromonas* and *Ochromonas* (in the broader sense, i.e., including *Chlorochromonas*) are among the most abundant mixotrophic flagellates particularly in oligotrophic and slightly acidic lakes. All three species show generally similar ecological habitat preferences as the selected prey species *Pn. asymbioticus*. *Ps. lacustris* is exclusively phagotrophic and, due to its small cell size feeds efficiently on ultramicrobacteria (Boenigk, 2004). The growth rates of the heterotrophic *Ps. lacustris* exceed those of the mixotrophic chrysophytes at high bacterial concentrations (Boenigk et al., 2006). The nutrition of *C. danica*, in contrast, is predominantly phototrophic (Schmidtke et al., 2006). Further, corresponding to its larger cell size grazing on ultramicrobacteria is less efficient. *P. malhamensis* is in-between both with respect to cell size and its nutritional mode (Boenigk, 2004). Despite the presence of a chloroplast in *P. malhamensis*, indifference in growth of cultures in light and dark indicate that photosynthesis plays a minor role (Boenigk et al., 2006). Ingestion rates, in contrast, have been demonstrated to be higher than for *C. danica* (Schmidtke et al., 2006). The mixotrophic species *C. danica* and *P. malhamensis* release a major part of their photosynthesis products, when photosynthetically active, in the form of dissolved exudates from which bacteria could benefit (Myklestad, 1995; Biddanda and Benner, 1997). The relative importance of exudation and grazing thus differs systematically between these taxa. To identify shifts in the bacterial response which correlate with the nutritional mode of the predator species, we performed sequencing of metatranscriptomes to obtain mRNA read abundances of *Pn. asymbioticus* and the protistan species.

## 2. Methods

### 2.1. Experimental Setting

*Pn. asymbioticus* (strain QLW-P1DMWA-1^*T*^, Hahn et al., 2016b) was co-cultured with single- and multi-species chrysophyte communities containing the species *Chlorochromonas danica* (strain 933-7, Pringsheim, 1955), *Poterioochromonas malhamensis* (strain DS, Boenigk et al., 2006) and *Poteriospumella lacustris* (strain JBM10, Findenig et al., 2010), see Table 1. All chrysophyte species are able to feed on bacteria-size microorganisms, but can also perform photosynthesis in the case of mixotrophy (*C. danica* and *P. malhamensis*) or depend on the uptake of bacteria and nutrients in the case of a heterotrophic form of nutrition (*Ps. lacustris*). The chrysophyte species have been selected to cover the range of trophic modes from more phototrophic (*C. danica*) over more heterotrophic (*P. malhamensis*) to exclusively heterotrophic (*Ps. lacustris*).

**Table 1:**
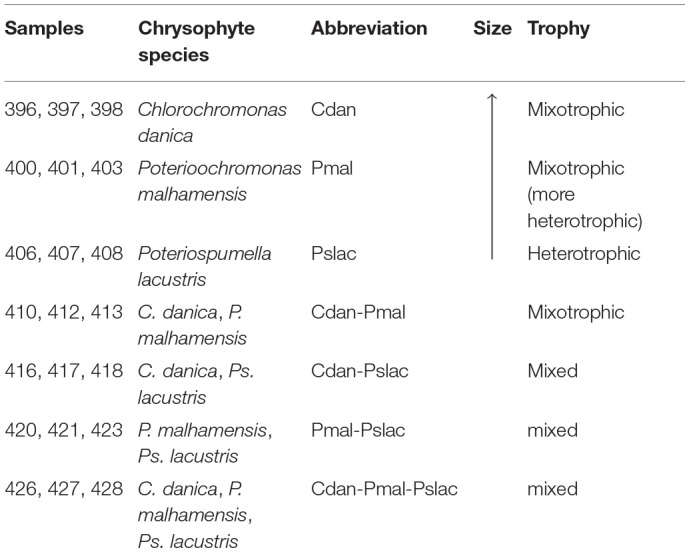
Experimental design. Overview of conditions and combinations of chrysophyte species which were grown with *Polynucleobacter asymbioticus*.

### 2.2. Culture Condition and Sample Preparation

Precultures of the axenic chrysophyte species were grown at 15°C in NSY (nutrient broth, soytone, yeast extract) medium (Hahn et al., 2004). Cultures were illuminated with 75–100 μE light intensity and a light:dark cycle of 16:8 h. For the experiment, cells were harvested by centrifugation at 3,000 g for 10 min at 20 °C and washed in modified DY-V medium (pH 6, Andersen, 2007).

*Pn. asymbioticus* was cultivated in NSY medium (3 g/L) under continuous shaking at room temperature. Prior to the experiment, cells were harvested by centrifugation at 5,000 g for 10 min at 20°C and washed three times with DY-V (pH 6) medium.

For the experiment, all samples were inoculated with the chrysophytes at the same biovolume, corresponding to 4.7 ×10^6^ μm^3^/ml in total for each treatment and 30 million bacteria per ml, which amounts to an 1,000-2,000 fold higher bacterial cell number than chrysophyte cells. In detail, the single-species treatments were inoculated with 17,000 cells per ml for *C. danica* (biovolume 278.94 μm^3^ per cell), 16,000 cells per ml for *P. malhamensis* (biovolume 294.94 μm^3^ per cell) and 30,000 cells per ml for *Ps. lacustris* (biovolume 156.75 μm^3^ per cell). Accordingly, half and 1/3 of the cells, respectively, were used for the multi-species treatments. Experiments were run in 3 replicates for 24 h in modified DY-V (pH 6) medium under the same settings as above. Bacteria were counted from formaldehyde-preserved subsamples by means of epifluorescence microscopy (Nikon Eclipse 80i) after DAPI (4′,6-diamidino-2-phenylindole) staining at 1000x magnification.

Samples were filtered onto 0.2 μm polycarbonate filters, air dried and immediately stabilized in RNA stabilization solution (LifeGuard Soil Preservation Solution, MoBio Laboratories Inc., Carlsbad, CA, USA). Filters were stored on –80°C until further processing.

### 2.3. RNA Extraction and Sequencing

RNA extraction was carried out under sterile conditions using TRIzol (Life Technologies, Paisley, Scotland–protocol modified). Prior to the RNA extraction the stabilization solution was removed from the filters. In brief, filters were ground in liquid nitrogen and incubated for 15 min in TRIzol at 15°C. Chloroform was added, shaken vigorously and incubated for 15 min at 15 °C. The mixture was centrifuged to achieve separation of phases. The aqueous phase was transferred to a new reaction tube and RNA was precipitated using isopropanol (incubation for 1 h at –20°C and centrifugation). The RNA pellet was washed three times in 75% ethanol and re-suspended in diethylpyrocarbonate (DEPC) water.

Sequencing was performed at the sequencing provider BGI (Hong Kong). They performed quality control of the samples, e.g., OD260/280 and OD260/230 with NanoDrop (Thermo Fisher Scientific, Waltham, MA, USA), concentration determination, 28S/18S and 23S/16S calculation and RIN test with Agilent 2100 (Agilent Technologies, Santa Clara, CA, USA), and performed rRNA depletion thereupon by mixing probes of Ribo-Zero rRNA Removal Kit (Bacteria), MRZB12424 and Ribo-Zero Gold rRNA Removal Kit (Human/Mouse/Rat), MRZG1224 (Illumina, San Diego, CA, USA). Sequencing libraries were prepared using TruSeq RNA Library Prep Kit v2 (Illumina, San Diego, CA, USA) after reverse transcription. Finally the libraries were paired-end sequenced with a read length of 100 bp on the Illumina HiSeq 4000 (Illumina, San Diego, CA, USA). Upon sequencing the data were filtered by an BGI-internal program called soapnuke to remove adapter sequences, contaminations and low-quality reads from the raw reads.

### 2.4. RNA-Seq Analysis

We obtained clean reads from BGI and rechecked the base qualities using the tools FASTQC (v0.11.3, Andrews, 2016) and MultiQC (v1.0, Ewels et al., 2016) and filtered the reads again with Cutadapt (v1.14, Martin, 2011). With Cutadapt potential Illumina sequencing adapters were removed and all bases with a quality value below 20 were trimmed, remaining sequences with a length below 70 bp were removed.

The clean reads were thereupon mapped to the genome of *Pn. asymbioticus* (strain QLW-P1DMWA-1, obtained from NCBI, BioProject: PRJNA224116) and a combination of the assembled genomes of *C. danica* strain 933-7, *P. malhamensis* strain DS and *Ps. lacustris* strain JBM10 (unpublished data). The read mapping was performed using Bowtie2 (v2.2.8, Langmead and Salzberg, 2012) with default parameters.

Reads were counted on gene-level using HTSeq-count (v0.9.1, Anders et al., 2014) and the GFF-files with intron/exon and gene boundaries for the species. HTSeq-count was used with the parameters –minequal 42, –nonunique=none, –secondary-alignment=ignore and –supplementary-alignments=ignore to minimize the false assignment of reads to homologous regions of a related species in the combined genome reference for the chrysophytes. All steps performed in the analysis were run using the Snakemake workflow management system (v4.0.0, Köster and Rahmann, 2012).

Subsequent differential expression analysis and plotting was performed in R (v3.3.2, R Development Core Team, 2008). We calculated size factors and performed a regularized logarithmic (rlog) transformation on the combined bacterial gene count matrix using DESeq2 (v1.14.1, Love et al., 2014). A principal component analysis was performed on the rlog transformed data and the biplot was visualized using ggplot2 (v2.2.0, Wickham, 2009). All other graphics were likewise created with the R package ggplot2, heatmaps with pheatmaps (Kolde, 2019) and GViz (Hahne and Ivanek, 2016) for genomic regions. Up- and downregulation of genes between conditions was calculated first by a likelihood ratio test (LRT) with DESeq2 (v1.14.1, Love et al., 2014). It compares a full model to a reduced model and is used for testing multiple terms at once conceptually similar to an analysis of variance (ANOVA), except that it uses an analysis of deviance (ANODEV), where the deviance captures the difference in likelihood between a full and a reduced model. A Wald test was used additionally to determine all pairwise differences between conditions. The DESeq2 model internally corrects for library size. Sequencing-depth normalized gene counts can be obtained from the model and throughout the manuscript, if not otherwise stated, these values are referred to as “normalized counts." Significant differentially expressed genes from the LRT were used to conduct a KEGG enrichment analysis (significance threshold: Benjamini-Hochberg corrected *p*-value < 0.05) and to visualize differentially expressed genes in KEGG pathways using own implementations in R and clusterProfiler (v3.6.0, Yu et al., 2012). Clusters of sequential differentially expressed genes, possibly representing operons, were calculated by expanding regions on each strand where the mean expression did not differ more than 2-fold and the length of the intergenic region was < 200 bp. We considered the annotation of these potential operons as a single unit because of co-regulation and joint transcription of the genes.

## 3. Results and Discussion

### 3.1. Responses of *Polynucleobacter*

*Polynucleobacter* was exposed to co-cultures of different single- and multi-species communities of three mixo- and heterotrophic chrysophytes. All of the chrysophytes feed on bacteria, whereby the grazing pressure is correlated with cell size and optimization of the predator-prey size ratio and is associated with the extent of reduction of the chloroplast (Graupner et al., 2018; Olefeld et al., 2018). Grazing on ultramicrobacteria is strongest for the smallest species *Ps. lacustris* and lowest for the species *C. danica* with the species *P. malhamensis* in between - corresponding to the decreasing cell size and the decreasing contribution of phototrophy in the nutrition from *C. danica* over *P. malhamensis* to *Ps. lacustris* (Boenigk et al., 2004, 2006; Schmidtke et al., 2006; Foster and Chrzanowski, 2012; Johnke et al., 2017).

**Figure 1:**
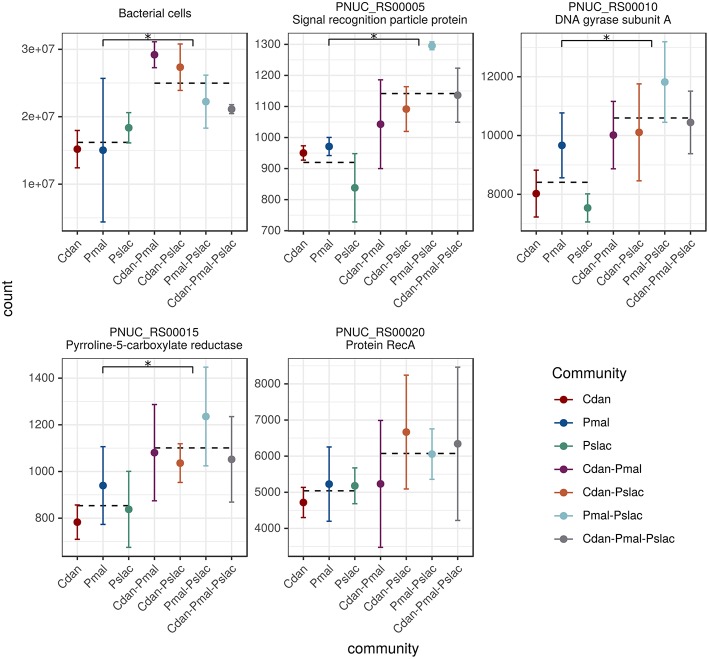
Bacterial cell counts and normalized read counts for housekeeping genes. The five subplots display the bacterial cell counts as well as normalized read counts of four housekeeping genes. The read counts act as further independent estimate of bacterial abundance changes. Since, by definition, the expression of housekeeping genes in a cell is relatively constant, changes in read counts arise from changes in the number of cells/organisms. The following housekeeping genes were considered: recombinase A, signal recognition particle protein genes, pyrroline-5-carboxylate reductase and DNA gyrase. For all counts the mean and the standard deviation over the replicates is shown colored by treatment. The means of the two tested groups (single-species vs. multi-species samples) are highlighted as dotted lines, asterisks mark significant differences in counts.

After 24 h of co-culturing with the different chrysophyte species mRNA was extracted for sequencing and bacterial cells were counted. In contrast to expectations, no significant difference in cell counts between the single-species treatments was observed (see Figure 1 and Table S1). This could be due to diverse strategies which allow bacteria to survive protistan grazing. The effects of predation on *Polynucleobacter* could be buffered by a high nutrient availability and growth despite the reduction by grazers as observed by Salcher et al. (2007) and Šimek et al. (2003, 2005). Further, the formation of inedible aggregates or microcolonies found in many bacterial species (Hahn and Höfle, 2001) could allow *Polynucleobacter* to escape predation. Apart from grazing, it was reported previously (Blom and Pernthaler, 2010) that *C. danica* and *P. malhamensis* have an antibiotic effect on many bacterial strains, which could affect the bacterial cell counts in the mixotrophic treatments. On the other hand, the samples containing both of the species exhibit the highest overall bacterial cell counts and do not seem to have a particular effect on bacterial growth (see Figure 1 and Table S1).

Surprisingly, we counted significantly less bacteria in the single-species samples compared to the multi-species samples (see Figure 1 top-left subplot, Welch Two Sample *t*-test, *p*-value < 0.001). We could confirm this finding using the sequencing data. Normalized read counts of four housekeeping genes *recA, ffh, proC* and *gyrA* (recombinase A, signal recognition particle protein genes, pyrroline-5-carboxylate reductase and DNA gyrase, respectively) were compared across treatments (see Figure 1). These housekeeping genes proved to be the most stably expressed ones out of 12 candidate genes in *Pectobacterium atrosepticum* (Takle et al., 2007) and some of the candidates were also used previously for *Pn. asymbioticus* (Hahn et al., 2016b). Since housekeeping genes are expressed in a cell at relatively constant rates, changes in read counts should arise from changes in the number of cells/organisms. Three of the four genes showed significant differences (adj. *p*-value < 0.01) between single-species samples and multi-species conditions based on Negative Binomial GLM Fitting and Wald statistics with DESeq2.

Multi-species communities differ in many aspects from the single-species protistan samples, including competition between the chrysophytes, possibly leading to a switch in nutritional mode in the mixotrophic species, increased exudate release, or predation among the chrysophyte species. Experiments carried out with mixed flagellate cultures showed for example that *P. malhamensis* ingested *Ps. lacustris* cells (Boenigk et al., 2006), potentially leading to a decrease in phagotrophic *Ps. lacustris* cells. The samples containing these two species exhibit higher bacterial cell counts compared to the single-species samples, but since this is the case for all multi-species samples, the specific effect can not be distinguished from a general benefit of a more diverse chrysophyte community. Regarding the release of exudates, *Pn. asymbioticus* has only limited capabilities to utilize carbohydrates in the form of saccharides, but it can utilize short-chain carboxylic acids, e.g., pyruvate, malate and succinate, and also diverse amino acids (Hahn et al., 2009, 2012). The composition of exudates matters and if exudates are very carbohydrate-rich, the strain may not be able to utilize the exudates as shown for the *Polynucleobacter* PnecC clade by Šimek et al. (2011) and Horňáket al. (2017). We, therefore, hypothesize that a more diverse flagellate community releases a more diverse exudate cocktail containing more substrates suitable for the bacterium.

Summarizing, the results from the bacterial cell counts indicate no significant changes due to phagotrophy or exudation in the single-species samples, but *Polynucleobacter* clearly benefits from a more diverse protist community, whether this comes from competition between the chrysophytes, an increased in exploitable exudates or decreased phagotrophy cannot be resolved at this point.

### 3.2. Transcriptomic Responses of *Polynucleobacter*

We performed mRNA sequencing to go beyond microscopic observations and gain insights into the molecular responses of *Polynucleobacter* to the changes in chrysophyte community. After quality control, we performed additional adapter trimming and removal of low quality bases and sequences, which, however, affected only 0.2% of the reads. In total, between 32.5 and 60.9% of the reads could be mapped to the *Pn. asymbioticus* genome (Table 2). Less reads mapped to annotated genes in the *Pn. asymbioticus* genome, on average 3,468,595 (2,298,974 to 4,900,315). While the number of mapped reads is generally high for the prokaryotes, the metatranscriptomes yield few eukaryotic reads in most samples (see Figure S1). In general, the relative read abundance of the chrysophytes represents the proportion of organisms in the mixed communities. Few reads were misassigned from *P. malhamensis* and *Ps. lacustris* to *C. danica* and the relative abundance of *Ps. lacustris* seems to be slightly reduced in all mixed samples.

**Table 2:** Sequencing and mapping statistics. Listed are the number of fragments from the sequencing company, percent of reads with a quality value above 20, percent of additionally trimmed reads, percent of mapped reads to the *Polynucleobacter asymbioticus* genome and mapped reads on genes per sample.

**Sample**	**Fragments**	**Reads > Q20 (%)**	**Trimmed (%)**	**Mapped to Pn. asymbioticus (%)**	**Mapped to Pn. asymbioticus genes**
396	17,560,434	99.14	0.2	36.5	2,940,551
397	19,294,290	99.15	0.2	32.6	2,905,916
398	20,354,521	99.12	0.2	47.2	4,403,439
400	14,201,955	97.96	0.2	34.5	2,298,974
401	16,052,767	99.05	0.2	32.5	2,436,092
403	18,425,963	99.12	0.2	44.2	3,755,776
406	15,931,886	99.11	0.2	54.7	3,930,025
407	17,713,359	99.12	0.2	42.3	3,440,719
408	20,480,468	99.09	0.2	39.1	3,671,773
410	14,288,108	99.08	0.2	59.4	3,926,373
412	17,643,655	99.09	0.2	50.8	4,123,679
413	17,569,308	99.13	0.2	60.9	4,900,315
416	14,856,821	99.12	0.2	57.2	3,888,116
417	14,914,081	99.06	0.2	44.0	3,026,763
418	19,011,458	99.15	0.2	42.4	3,687,603
420	16,173,417	98.41	0.2	42.9	3,233,300
421	16,923,006	99.05	0.2	50.2	3,923,094
423	10,966,577	98.46	0.2	47.7	2,447,812
426	15,905,741	99.06	0.2	41.6	3,084,399
427	13,998,166	99.03	0.2	37.7	2,441,184
428	20,458,705	99.08	0.2	46.0	4,374,605

**Figure 2:**
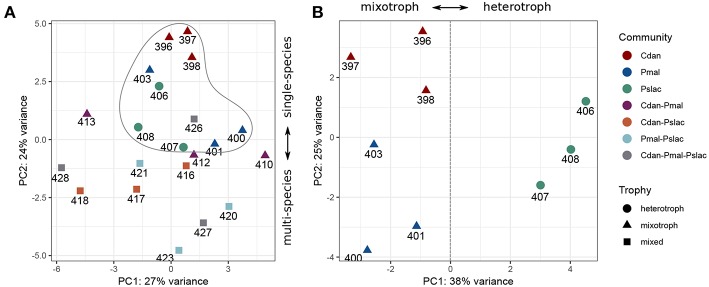
Principal component analysis based on normalized gene-wise read counts for *Pn. asymbioticus*. **(A)** For all samples, **(B)** for single community samples. Samples are colored according to the protistan community and the shape depicts the trophic mode of the community. The sample community clearly influences the gene expression of *Polynucleobacter*, which can be seen in a separation between single- and multi-species samples **(A)** as well as a separation according to trophy **(B)**.

The sequencing data reveals that the gene expression of *Polynucleobacter* is influenced differentially by the predominant type of interactions, i.e., exudation or grazing, and by the the presence of different chrysophyte communities. We performed a principal component analysis (PCA) on the rlog transformed expression data for all samples and the single-species samples (see Figures 2A,B). From the PCA with all samples (Figure 2A) we see a clustering of the single species communities (encircled) along the second principal component with an explained variance of 24% and considering only the single species samples, we observe a separation between samples containing mixotrophic chrysophytes and heterotrophic chrysophytes along the first principal component with an explained variance of 38% (Figure 2B). The effects interfere and the clustering by chrysophyte species and separation of trophic modes along the first axis is more obvious when considering only the samples where *Polynucleobacter* was cultured with a single species. The subsequent analyses first focus on these single-species samples and their impact on the gene expression of *Polynucleobacter*.

#### 3.2.1. Effect of Different Single Species

**Figure 3:**
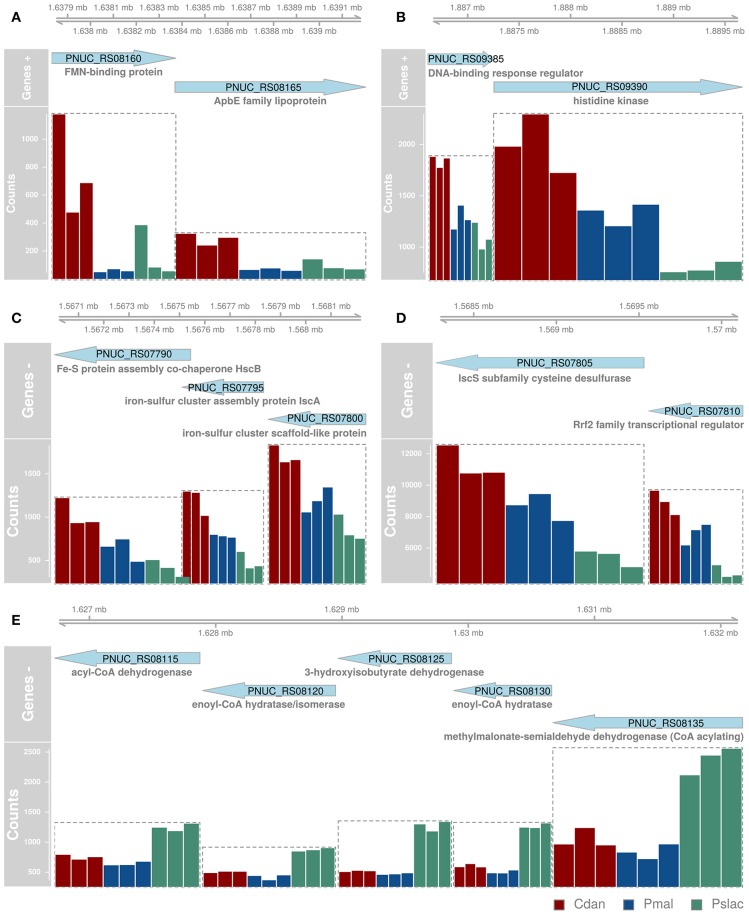
Most significant differentially expressed genes between single-species conditions which are structured in operons. Since many bacterial genes are organized in operons and expressed together, genes which likely belong to the same operon **(A-E)** are depicted here with their position in the genome (top), orientation (genes +, genes -) and normalized gene counts as bars colored by sample type. Since we did not want to overestimate gene functions of differential genes with similar functions belonging to the same operon, we had to determine the operons prior to functional analyses. All significant differentially expressed genes can be found in Figure S2 and the 20 most differentially expressed genes (independent of operon structure) are also depicted in Figure 4.

**Figure 4:**
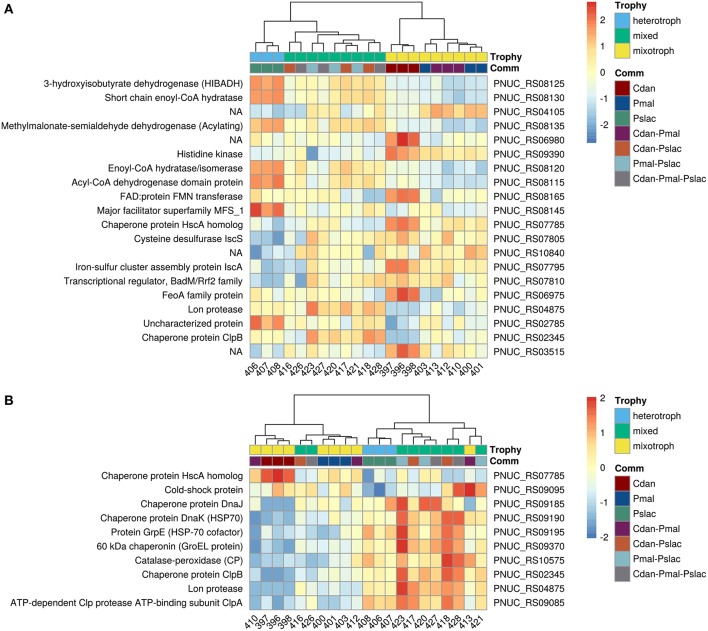
Heatmap of gene expression for *Pn. asymbioticus* in all samples. **(A)** Depicts the 20 most differentially expressed genes of 150 between the conditions in single-species samples ordered by Benjamini-Hochberg adjusted *p*-value. **(B)** depicts significant differentially expressed genes which are annotated to stress responses. The heatmaps show the z-transformed normalized read counts on log2 scale clustered using hierarchical clustering with the Ward-D2 method and euclidean distances (correlation distances yield the same clustering). The dendrograms are depicted above the heatmaps. As annotation of the samples the protistan community and the trophic mode of the community members is indicated below the dendrograms. In **(A)** we primarily observe a clustering of samples due to their gene expression according to single- and multi-species community and further a clustering by trophy. In **(B)** we observe a similar clustering by trophy and community, but with some outliers in the replicates (samples 416, 426, and 413).

Analysis of deviance between the single predator species samples yield 150 significant differentially expressed genes (Benjamini-Hochberg corrected *p*-value < 0.01) of 2,167 genes in *Polynucleobacter* (Table S2 and Figure S2). Pairwise tests between conditions revealed that most changes occur between samples containing *P. malhamensis* and *Ps. lacustris* with 113 significantly differentially expressed genes and between *C. danica* and *Ps. lacustris*. Between the latter two the changes are slightly lower with 98 affected genes. Fewest genes are significantly differentially expressed between *C. danica* and *P. malhamensis* resulting in only 26 genes. We observe the same pattern which we already discovered in the principal component analysis - differences in gene expression due to the presence of *Ps. lacustris* or between mixotrophic and heterotrophic modes of nutrition in the predator species. If we take a closer look at the 20 most significant genes, 10 belong to operon structures (see Figure 3) and 16 can be annotated to a gene name and therefore function (see Figure 4A). Including the genome annotation from NCBI, it is evident that several genes are involved in the degradation of amino acids and fatty acids such as the 3-hydroxyisobutyrate dehydrogenase (*HIBADH*, PNUC_RS08125), methylmalonate-semialdehyde dehydrogenase (PNUC_RS08135) and Acyl-CoA dehydrogenase domain protein (PNUC_RS08115) which act in amino acid catabolism or degradation and genes acting in the β-oxidation pathway and fatty acid breakdown such as the short chain enoyl-CoA hydratase (PNUC_RS08130) and enoyl-CoA hydratase/isomerase (PNUC_RS08120). All of these genes show a higher expression in the samples containing the heterotrophic *Ps. lacustris* and the analysis of the operon structure reveals that the afore mentioned genes lie consecutively on the same strand of the genome and display similar expression (Figure 3). It is likely that they are part of an operon region and that they are therefore co-expressed.

In plants it is known, that in metabolic stress situations the amino acid catabolism is important to generate alternative substrates for respiration (Schertl et al., 2017). Another higher expressed gene in the *Ps. lacustris* samples is the major facilitator superfamily transporter MFS1 (PNUC_RS08145) which is responsible for the import and export of various substrates including sugars, metabolites, oligosaccharides, amino acids, ions and drugs. A reduced expression in these samples is present for the genes annotated with chaperone protein HscA homolog (PNUC_RS07785) and cysteine desulfurase IscS (PNUC_RS07805). Both are responsible for the assembly and correct folding of proteins with iron-sulfur clusters. Considering the annotation of all significant differentially expressed genes, it is evident that several more genes can be annotated to stress response processes and proteins containing iron cofactors.

The 150 genes can be attributed to 4 main categories: 30 genes can be linked via the gene name, KEGG Orthology IDs or GO annotation to transcriptional or translational activity or their regulation, out of these 29 genes show lower expression in the heterotrophic samples. 17 genes contain iron-sulfur cluster or bind iron ions, 27 genes are part of the membrane or participate in the transport of ions or molecules across the membrane and 10 genes are involved in diverse stress responses (see Table S2 and Figure S3). While *Polynucleobacter* samples containing *C. danica* and *P. malhamensis* mainly show an upregulation of transcription and translation, in contrast samples containing *Ps. lacustris* show a higher expression of genes acting in general transport processes or response to stressors, e.g., chaperones DnaJ (PNUC_RS09185), DnaK (PNUC_RS09190), GrpE (PNUC_RS09195), GroEL (PNUC_RS09370), ClpB (PNUC_RS02345), ClpA (PNUC_RS09085), a potential stress responses of the Lon protease (PNUC_RS04875) and catalase-peroxidase (PNUC_RS10575) (see Figure 4B; Figure S3 and Table S1). In these samples higher stress is likely a result from higher grazing pressure while exudation; i.e., substrate availability, is presumably lower. Although from the counts of bacterial cells and housekeeping genes (Figure 1 and Table S1) there are less bacteria in samples 396–398 containing *C. danica*, protein synthesis catalyzed by ribosomes seems to be highly active in these samples, facilitating bacterial growth.

Further genes can be associated with iron, e.g., iron transport, iron-sulfur cluster assembly or iron-binding. Iron is essential for almost all organisms as it acts as important trace metal and cofactor of many cellular proteins. At high concentrations, however, it can be toxic due to the formation of reactive oxygen species, therefore, its concentration must be carefully managed. In the environment iron is mostly present in the ferric form (Fe^3+^), which is very poorly soluble, not in the better soluble ferrous form (Fe^2+^). Several iron uptake systems exist, but in the analyzed *Pn. asymbioticus* and most Chrysophyceae only ferrous-uptake systems are known, such as the ferrous iron transporter FeoAB (Munch, 1972; Ishida et al., 1982; Van Dork, 1982; Hahn et al., 2016a). In many bacteria it is regulated by the transcriptional regulator Fur (Hantke, 1981; Bagg and Neilands, 1987). The Fur protein is capable of binding DNA and thereby prevents the expression of downstream genes such as *feoA* and *feoB*. Under low concentrations of iron, it undergoes a conformational change that prevents DNA binding and lifts the repression. Fur was identified by a BLAST search with the Fur protein sequence of *Polynucleobacter necessarius* in *Pn. asymbioticus*. Fur expression is slightly lower in the single-species samples compared to the multi-species samples. In contrast, the expression of its regulated genes *feoA* and *feoB* (Figure 3A; Figure S3) is about 4-fold increased in the samples containing *C. danica*, compared to all other samples, indicating potential iron limitation in these samples. The medium contains Fe^3+^ as iron source, which can neither be utilized by *Pn. asymbioticus* [see comparison of strains in Hahn et al. (2016a)] nor the chrysophytes. For *Ochromonas* sp. it has been shown that it feeds on iron-rich bacteria for iron acquisition in the absence of dissolved iron, since for the predominantly phototrophic chrysophyte iron is highly relevant to assemble proteins necessary for photosynthetic electron transport and chlorophyll synthesis (Maranger et al., 1998; Lie et al., 2018). In other chrysophyte species it was suggested as important limiting micrometal, whose supplementation favors the growth of the genera *Dinobryon, Synura*, and *Uroglena* (Munch, 1972; Ishida et al., 1982; Van Dork, 1982).

Additional iron-associated genes which are more highly expressed in *C. danica*-containing samples, mainly act in iron-sulfur cluster assembly (*isc* operon: including *IscR* (named *Rrf2* transcriptional regulator in *Pn. asymbioticus*, identified by BLAST searches), *IscS, IscU, IscA* and *HscA*), ferrous ion transport (e.g., *FeoA, FeoB, TonB*) or bind iron or 2Fe-2S (ferredoxin, 2Fe-2S-binding protein and lipopolysaccharide assembly protein B). The conserved *isc* operon (*iscRSUA-hscBA-fdx-iscX* in *E. coli*, Fontecave et al., 2005) contains the transcription factor *IscR* which represses transcription of the operon containing its own gene, assuming that the operon structure is the same for *Pn. asymbioticus*. On the contrary, we find very different normalized read counts for the *isc* operon genes, indicating that they are either not part of one operon or IsrR stronger represses downstream target genes. For *Rrf2* (*IscR*, PNUC_RS07810) and *IscS* (PNUC_RS07805) the counts for the *C. danica*-samples range around 10,000–12,000 reads, while *IscU* (PNUC_RS07800), *IscA* (PNUC_RS07795), *HscB* (PNUC_RS07790), *HscA* (PNUC_RS07785), and *fdx* (PNUC_RS07780) counts reach at most 3,000 reads (see Figure S3). The decrease in expression downstream of *IscR* could further indicate a shut-down of iron-sulfur cluster assembly due to limitations in iron.

In contrast, the iron-associated genes which are more highly expressed in *Ps. lacustris*-containing samples include a high potential iron-sulfur protein (HiPIP) with 4Fe-4S ferredoxins for anaerobic electron transfer and several cytochrome c family proteins, especially three subunits of the cytochrome c oxidase cbb3-type that function under microraerobic conditions. This functional module M00156 (Cytochrome c oxidase, cbb3-type), besides the module M00178 (Ribosome, bacteria), is also significantly enriched (Benjamini-Hochberg adjusted *p*-value < 0.05) in a KEGG enrichment analysis. But it has to be noted, that out of the 150 genes only 34 could be annotated with KEGG Orthology IDs.

The observation that samples containing *Ps. lacustris* show indications of a higher stress response also holds true when considering all samples. Figure 4B shows the z-transformed normalized read counts of significant genes annotated to stress response in the single-species samples for all treatments. Likewise, samples containing *Ps. lacustris* in the mixture (in the Figure 4B: Trophy green+blue vs. yellow), show mostly a higher stress response than the samples with only *C. danica* and *P. malhamensis*. In general, samples containing *Ps. lacustris* often display differential expression levels (also see Figure 4A: Trophy green+blue vs. yellow). Out of the 150 genes which were identified with analysis of deviance as significantly differentially expressed in the single-species samples, 90 genes show significant differential expression (Benjamini-Hochberg adjusted *p*-value < 0.01) when testing the samples containing *Ps. lacustris* against all other samples.

#### 3.2.2. Effect of Multi-Species Communities

From the bacterial count data and analysis of housekeeping genes we already observed variations between the single- and multi-species conditions. A test of differential expression in gene counts between the two groups of samples using Wald statistics in DESeq2 showed small but significant changes in 192 genes of *Polynucleobacter*. The annotation to KEGG modules, pathways and GO terms yielded an enrichment of the KEGG module M00157 (F-type ATPase, prokaryotes and chloroplasts) participating in oxidative phosphorylation with higher expression of genes in the single-species samples. In contrast, the multi-species samples showed an enrichment in the KEGG module M00118 (Glutathione biosynthesis, glutamate => glutathione). Glutathione (GSH) is an important antioxidant and capable of preventing damage caused by reactive oxygen species. Oxidative stress response systems as environmental defenses, e.g., the glutathione synthetase, are known to be particularly reduced in the closely related species *Polynucleobacter necessarius*, which is obligate symbiotic within ciliate *Euplotes* species (Heckmann, 1975; Heckmann et al., 1983; Heckmann and Schmidt, 1987; Vannini et al., 2005). Possibly, compared to free-living strains, interaction with the environment became less necessary (Boscaro et al., 2013).

**Figure 5:**
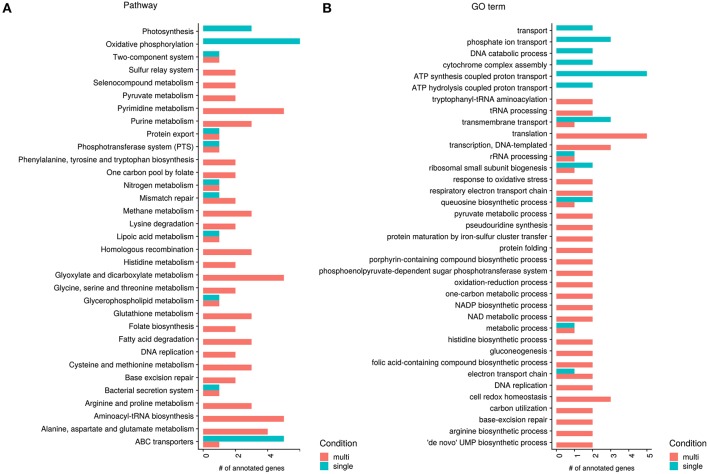
Significant genes between single- and multi-species conditions annotated to KEGG pathways and GO terms (molecular function). Shown are all KEGG pathway **(A)** and GO molecular function **(B)** annotations with at least two genes separated by higher expression in single- or multi-species samples. Red bars show higher expressed processes in the multi-species samples and green bars higher expression in the single species samples.

The KEGG and GO annotation of significant genes shows that several genes which are higher expressed in the single-species samples participate in oxidative phosphorylation or function as ABC transporters, or more generally in active transport (see Figure 5). The KEGG annotation to photosynthesis results from the same genes occurring in photosynthesis and oxidative phosphorylation, photosynthetic processes are not yet known for *Pn. asymbioticus* which makes changes in the oxidative phosphorylation more likely. The increased oxidative phosphorylation creates ATP, which can be used for metabolic processes and synthesis of biomolecules, but also active transport across membranes. ABC transporters are transmembrane proteins that actively translocate various substrates across the membrane. The annotation of one gene to an amino acid ABC transporter, hints to an uptake of substrates from the medium, possibly exudates from the mixotrophic protists. The specific substrates of the other ABC transporters are unknown.

Apparently in contrast to the assumptions made above, that in the single-species samples the mixotrophs have a more heterotrophic mode of nutrition, *Polynucleobacter* shows in the single-species samples a higher expression in genes annotated to (amino acid) ABC transporters. But it has been shown that a response to amino acid starvation and nutrient limitation is the selective transcription and translation of specific mRNAs associated with upregulation of genes with functions in amino acid biosynthesis, retention, and scavenging (Hosie and Poole, 2001; Taylor, 2013). Here an important mechanism is the upregulation of ABC transporters as an amino acid scavenger mechanism during periods of deficiency by a process called “adaptive regulation." Therefore, this does not speak against the assumption of a more heterotrophic mode of nutrition of the mixotrophic chrysophyte species in the single-species samples.

In the multi-species samples no specific functions turn up that hint to interactions with the chrysophyte species. Most significant higher expressed genes in these samples can be attributed to the primary metabolism including translation, transcription, amino acid, and carbohydrate metabolism.

### 3.3. Conclusions

The growth and/or transcriptomic response of *Pn. asymbioticus* shows:

- effects due to differences in the complexity of the chrysophyte community,
- effects attributable to the relative importance of grazing vs. exudation, i.e., the presence of
  - the heterotrophic chrysophyte *Ps. lacustris* in all samples and
  - the mixotrophic chrysophyte *C. danica* in the single-species samples.

From all parts of the analysis, cell counts, housekeeping gene and gene expression analysis, we observe that a more diverse predator community has the strongest effect on *Pn. asymbioticus*. Especially the impact of mixotrophic species depends on their dominant mode of nutrition (phagotrophy or phototrophy) (Rothhaupt, 1996b, 1997), which determines whether they act as predators of bacteria, compete with them for limiting nutrients (Thingstad et al., 1996) or provide nutrient-rich exudates from which *Polynucleobacter* benefits. We assume that in the multi-species communities, where we observed more bacteria, the mixotrophic species might change their mode of nutrition toward phototrophy due to competition for food with the other (heterotrophic) chrysophyte species, as e.g., described by Rothhaupt (1996a) for *C. danica* and *Spumella* species. This would lead to an increased release of photosynthetic exudates and a lower grazing pressure on *Polynucleobacter*. Alternatively, a more diverse chrysophyte community could release a more diverse exudate cocktail containing more substrates suitable for the bacterium. Both alternatives suggest a less nutritious environment in the presence of the single chrysophyte species. Accordingly, we detected a higher expression of genes annotated to (amino acid) ABC transporters in the single-species samples, which is likely due to an “adaptive regulation" of transporters as response to amino acid starvation and nutrient limitation under these conditions.

The presence of *Ps. lacustris*, the only exclusively phagotrophic species, caused significant changes in expression of 90 genes, of which a high number could be attributed to stress response pathways, presumably due to higher grazing pressure. Differences in gene expression for *C. danica*-containing samples could in many cases be ascribed to iron uptake and iron-sulfur cluster assembly. For *C. danica*, a predominantly phototrophic species, iron plays an important role as it is a component of many proteins involved in photosynthetic electron transport and chlorophyll synthesis. A rapid growth of *C. danica* under phototrophic growth could therefore lead to iron-deficient conditions and an upregulation of iron transporters as well as a shut-down of iron-sulfur cluster assembly in *Polynucleobacter*.

Unfortunately, there were not enough eukaryotic sequencing reads available to analyse the corresponding expression in chrysophytes in detail. Preliminary correlation analyses hint to possible interactions via membrane trafficking, but more information on chrysophyte genes and better annotations are necessary to understand these molecular interactions. The transcriptional response in chrysophytes would assist in the disentanglement of influencing factors including competition, nutrient limitation, toxin production and changes in trophy.

## Data Availability

The raw sequencing data analyzed for this study can be found in the Sequence Read Archive (SRA) at NCBI via the SRA accession: PRJNA523328 (https://www.ncbi.nlm.nih.gov/bioproject/PRJNA523328).

## Author Contributions

JB, SR, BS, and MV contributed conception and design of the study. CB performed the experiments. DB performed the bioinformatic and statistical analyses. DB, MH, and JB interpreted the results. DB wrote the first draft of the manuscript. CB, JB, and MH wrote sections of the manuscript. All authors contributed to manuscript revision, read, and approved the submitted version.

### Conflict of Interest Statement

The authors declare that the research was conducted in the absence of any commercial or financial relationships that could be construed as a potential conflict of interest.
