# Supplementary material for: Interaction-Specific Changes in the Transcriptome of Polynucleobacter asymbioticus Caused by Varying Protistan Communities
Source: Front Microbiol. 2019 Jul 9;10:1498. doi: 10.3389/fmicb.2019.01498 (PMC6629928; doi:10.3389/fmicb.2019.01498)
Supplement: Supplementary file 1 [file Data_Sheet_1.PDF]

Supplementary Material

1 SUPPLEMENTARY DATA

2 SUPPLEMENTARY TABLES AND FIGURES

2.1 Tables

Table S1. Bacterial cell counts. Cell counts for all samples after formaldehyde preservation by means of epifluorescence microscopy.

Table S2. Significant differentially expressed genes from ANODEV analysis. Differentially expressed genes are listed including test statistics (baseMean, log2FC, padj) and gene annotation (geneID, proteinName, pathway, GOMolecularFunction, GOBiologicalProcess, GOCellularComponent, KEGGOrthology).

2.2 Figures

Figure S1. Relative read abundance for different organisms in sample. Contribution of all organisms (*Pn. asymbioticus* (Pnasy), *C. danica* (Cdan), *P. malhamensis* (Pmal) and *Ps. lacustris* (Pslac)) to the community. While the bacterial counts are high, the metatranscriptomes contain few eukaryotic reads.

Figure S2. Significant differentially expressed genes from ANODEV analysis. Each gene is depicted with its genomic position, possible operon structure, orientation and normalized read counts for the single-species samples.

Figure S3. Significant differentially expressed genes from ANODEV analysis annotated to the categories iron, membrane & transport, regulation, transcription & translation and stress. Samples are sorted according to absence or presence of *Poteriospumella* in the samples.
