# Supplementary figures and images for: Interaction-Specific Changes in the Transcriptome of Polynucleobacter asymbioticus Caused by Varying Protistan Communities
Source: Front Microbiol. 2019 Jul 9;10:1498. doi: 10.3389/fmicb.2019.01498 (PMC6629928; doi:10.3389/fmicb.2019.01498)

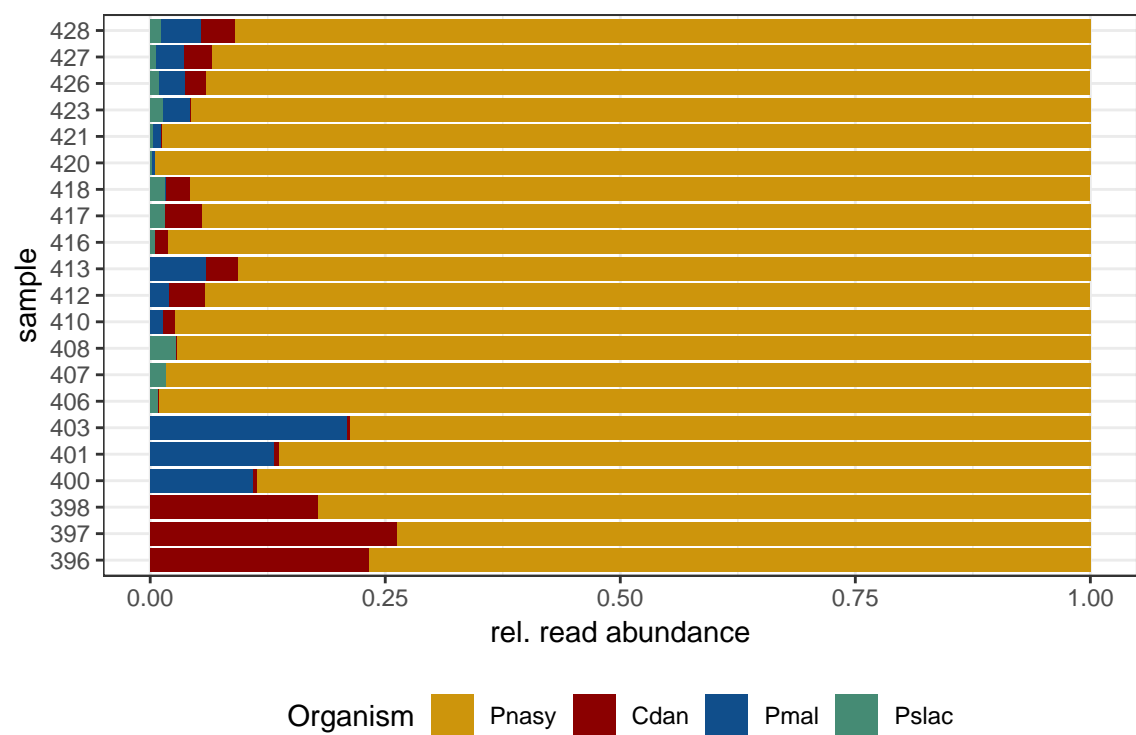

Supplement: Supplementary file 2 [file Data_Sheet_2.PDF]
