# Supplementary material for: Interaction-Specific Changes in the Transcriptome of Polynucleobacter asymbioticus Caused by Varying Protistan Communities
Source: Front Microbiol. 2019 Jul 9;10:1498. doi: 10.3389/fmicb.2019.01498 (PMC6629928; doi:10.3389/fmicb.2019.01498)
Supplement: Supplementary file 3 [file Data_Sheet_3.PDF]

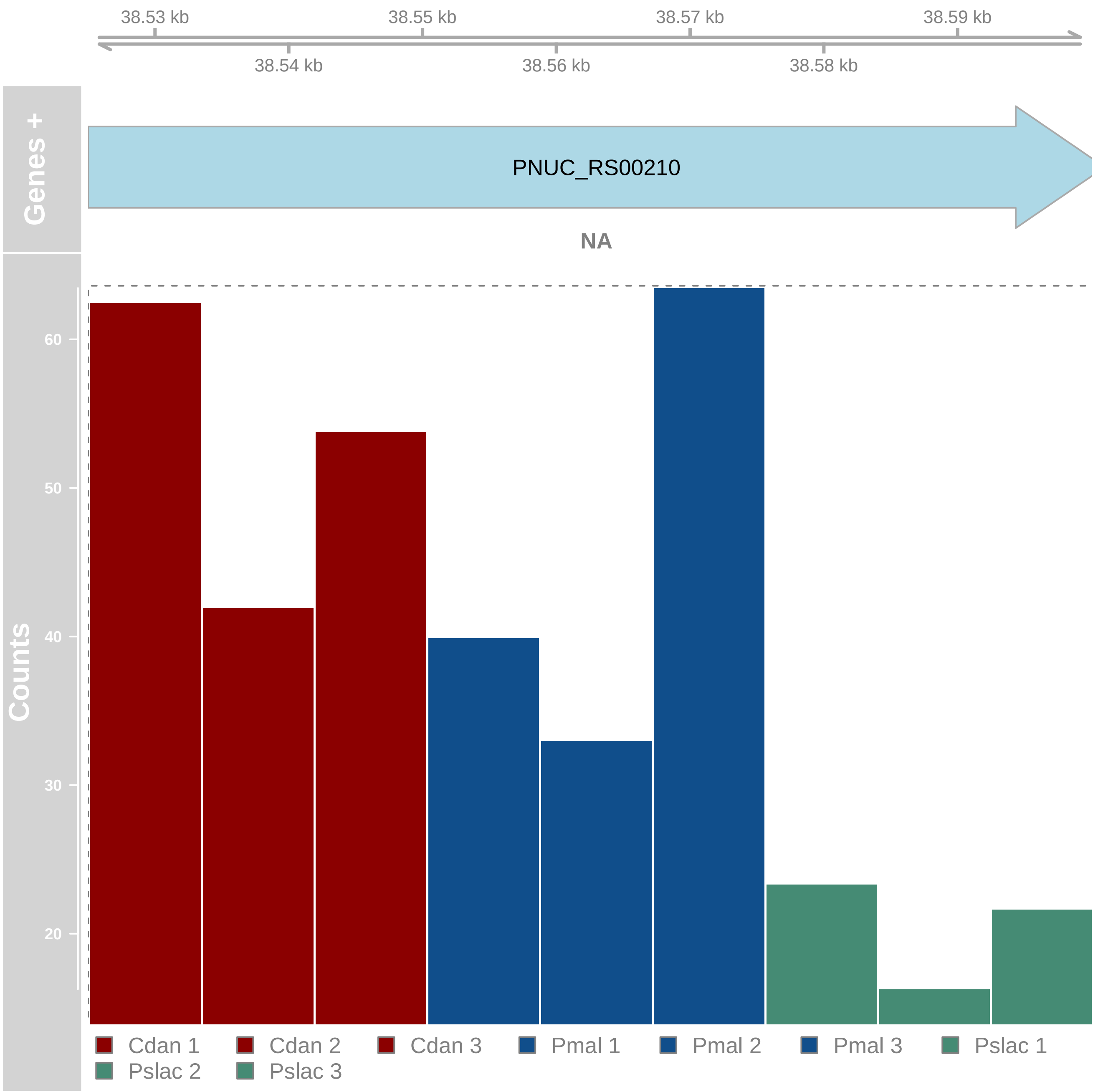
38.53 kb
38.55 kb
38.57 kb
38.59 kb
38.54 kb
38.56 kb
38.58 kb
Genes +
PNUC_RS00210
NA
Counts
60
50
40
30
20
Cdan 1
Cdan 2
Cdan 3
Pmal 1
Pmal 2
Pmal 3
Pslac 1
Pslac 2
Pslac 3

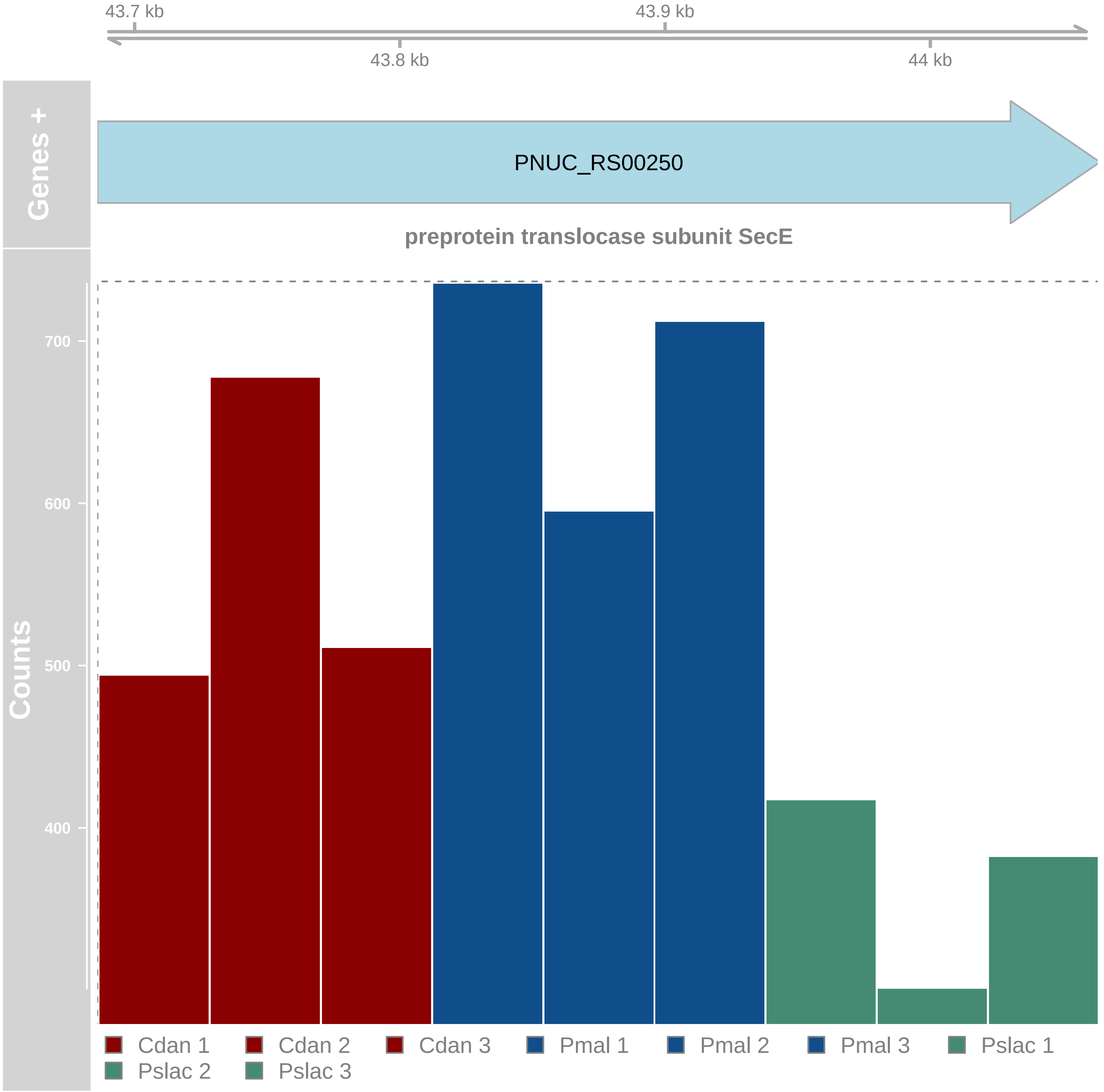

43.7 kb
43.8 kb
43.9 kb
44 kb
Genes +
PNUC_RS00250
preprotein translocase subunit SecE
Counts
700
600
500
400
Cdan 1
Cdan 2
Cdan 3
Pmal 1
Pmal 2
Pmal 3
Pslac 1
Pslac 2
Pslac 3

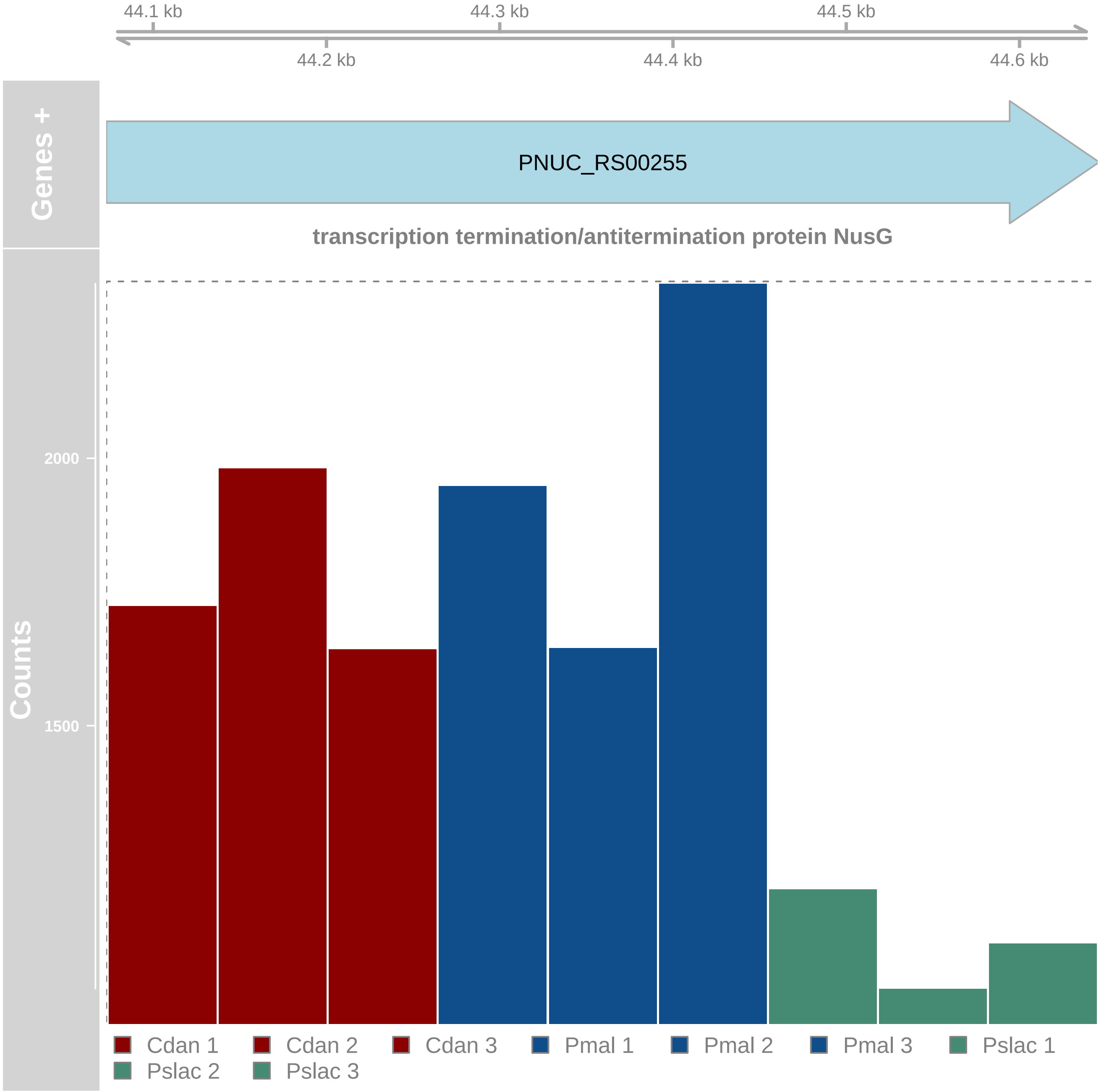

44.1 kb
44.3 kb
44.5 kb
44.2 kb
44.4 kb
44.6 kb
Genes +
PNUC_RS00255
transcription termination/antitermination protein NusG
Counts
2000
1500
Cdan 1
Cdan 2
Cdan 3
Pmal 1
Pmal 2
Pmal 3
Pslac 1
Pslac 2
Pslac 3

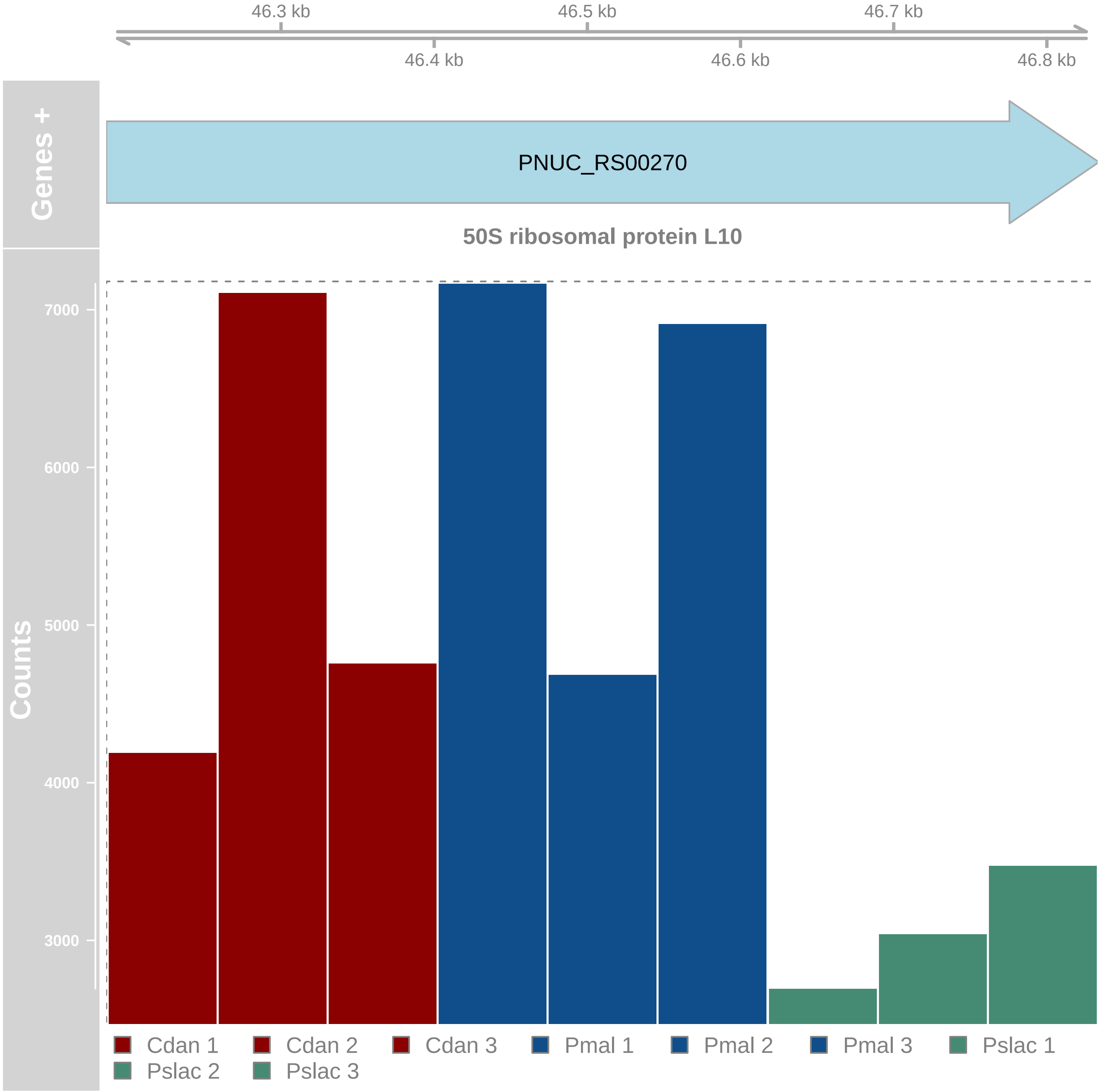
46.3 kb
46.5 kb
46.7 kb
46.4 kb
46.6 kb
46.8 kb
Genes +
PNUC_RS00270
50S ribosomal protein L10
Counts
7000
6000
5000
4000
3000
Cdan 1
Cdan 2
Cdan 3
Pmal 1
Pmal 2
Pmal 3
Pslac 1
Pslac 2
Pslac 3

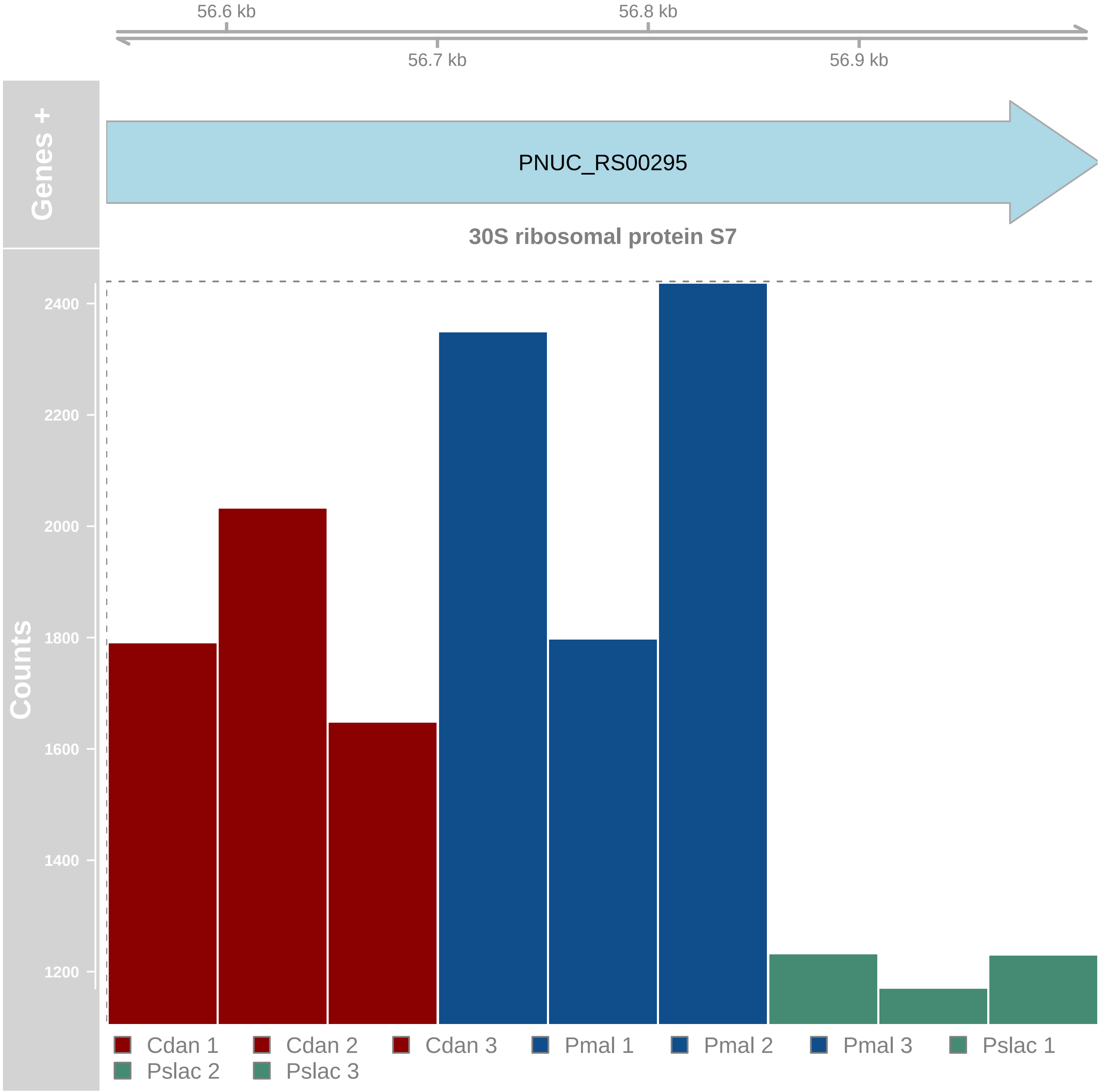
56.6 kb
56.8 kb
56.7 kb
56.9 kb
Genes +
PNUC_RS00295
30S ribosomal protein S7
Counts
2400
2200
2000
1800
1600
1400
1200
Cdan 1
Cdan 2
Cdan 3
Pmal 1
Pmal 2
Pmal 3
Pslac 1
Pslac 2
Pslac 3

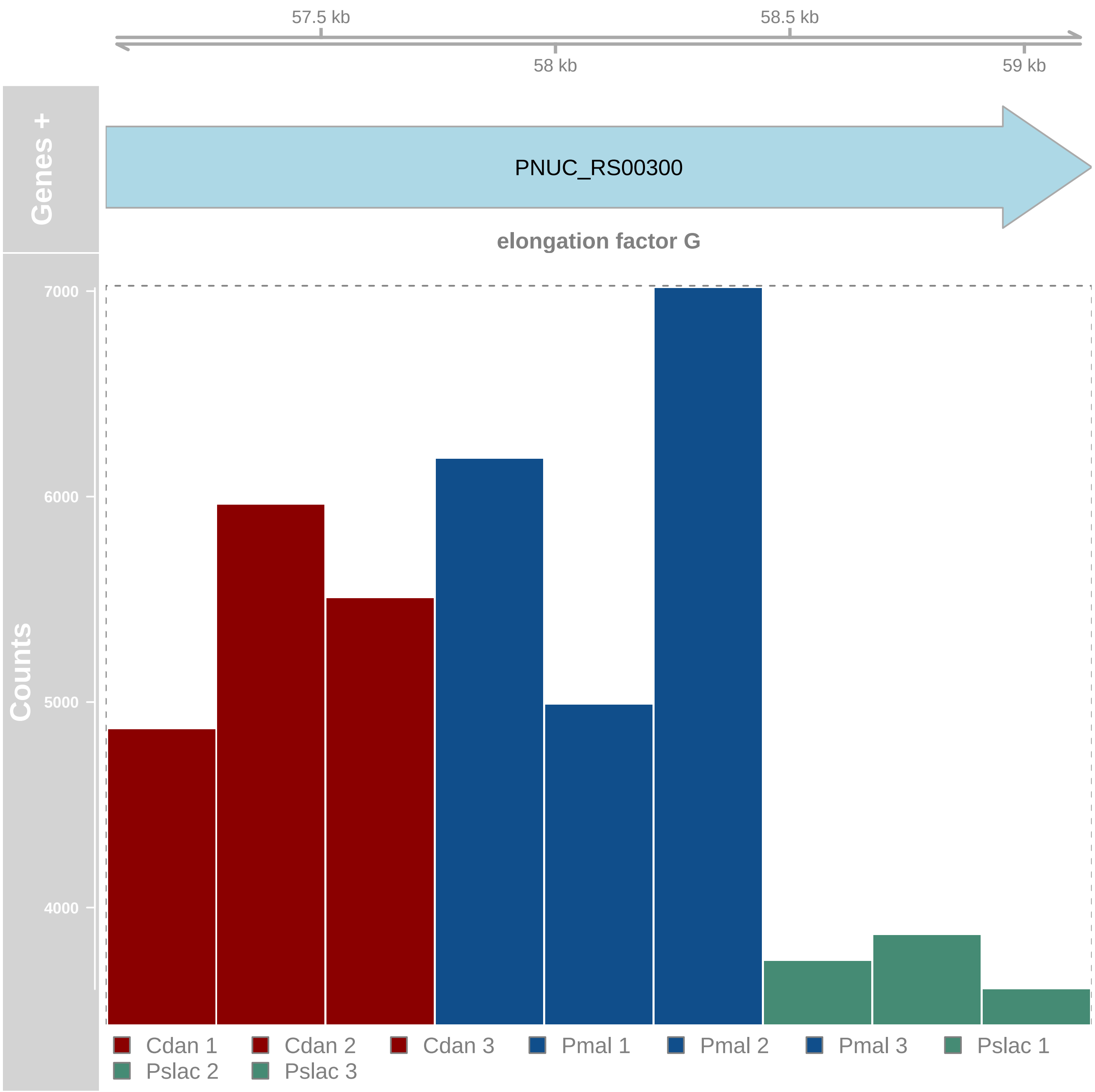

57.5 kb
58.5 kb
58 kb
59 kb
Genes +
PNUC_RS00300
elongation factor G
Counts
7000
6000
5000
4000
Cdan 1
Cdan 2
Cdan 3
Pmal 1
Pmal 2
Pmal 3
Pslac 1
Pslac 2
Pslac 3

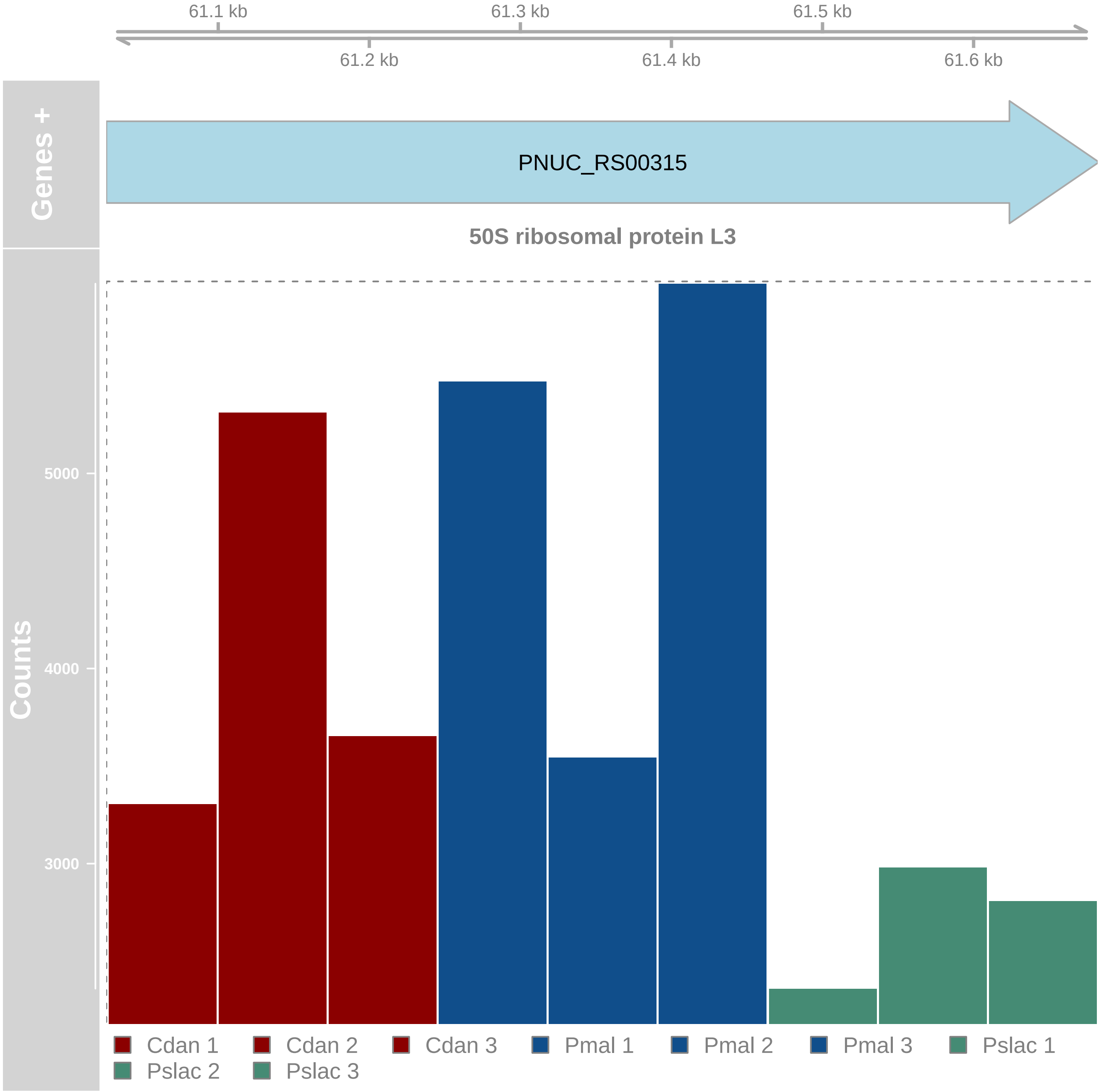

61.1 kb
61.3 kb
61.5 kb
61.2 kb
61.4 kb
61.6 kb
Genes +
PNUC_RS00315
50S ribosomal protein L3
Counts
5000
4000
3000
Cdan 1
Cdan 2
Cdan 3
Pmal 1
Pmal 2
Pmal 3
Pslac 1
Pslac 2
Pslac 3

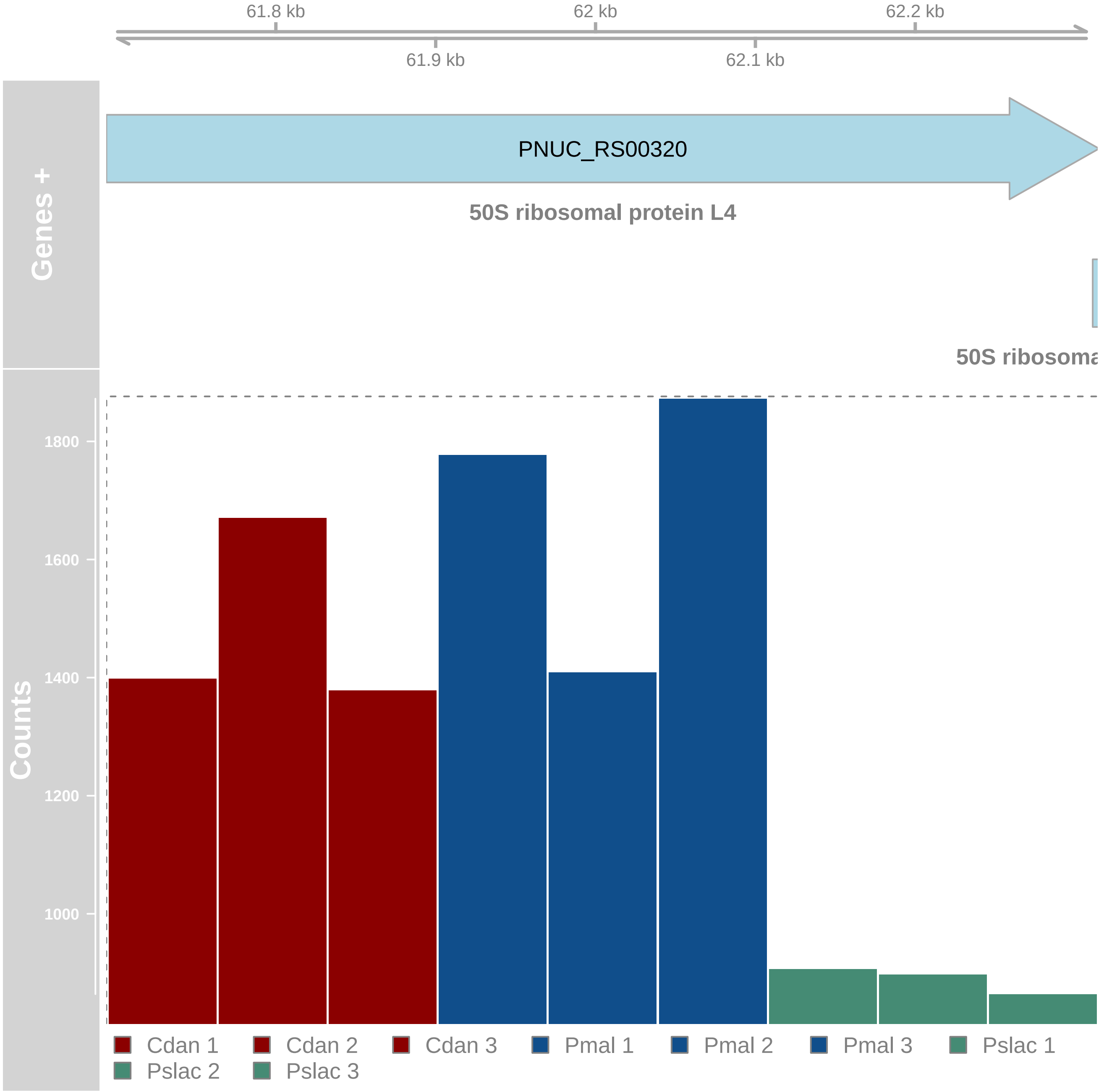
61.8 kb
62 kb
62.2 kb
61.9 kb
62.1 kb
Genes +
PNUC_RS00320
50S ribosomal protein L4
50S ribosoma
Counts
1800
1600
1400
1200
1000
Cdan 1
Cdan 2
Cdan 3
Pmal 1
Pmal 2
Pmal 3
Pslac 1
Pslac 2
Pslac 3

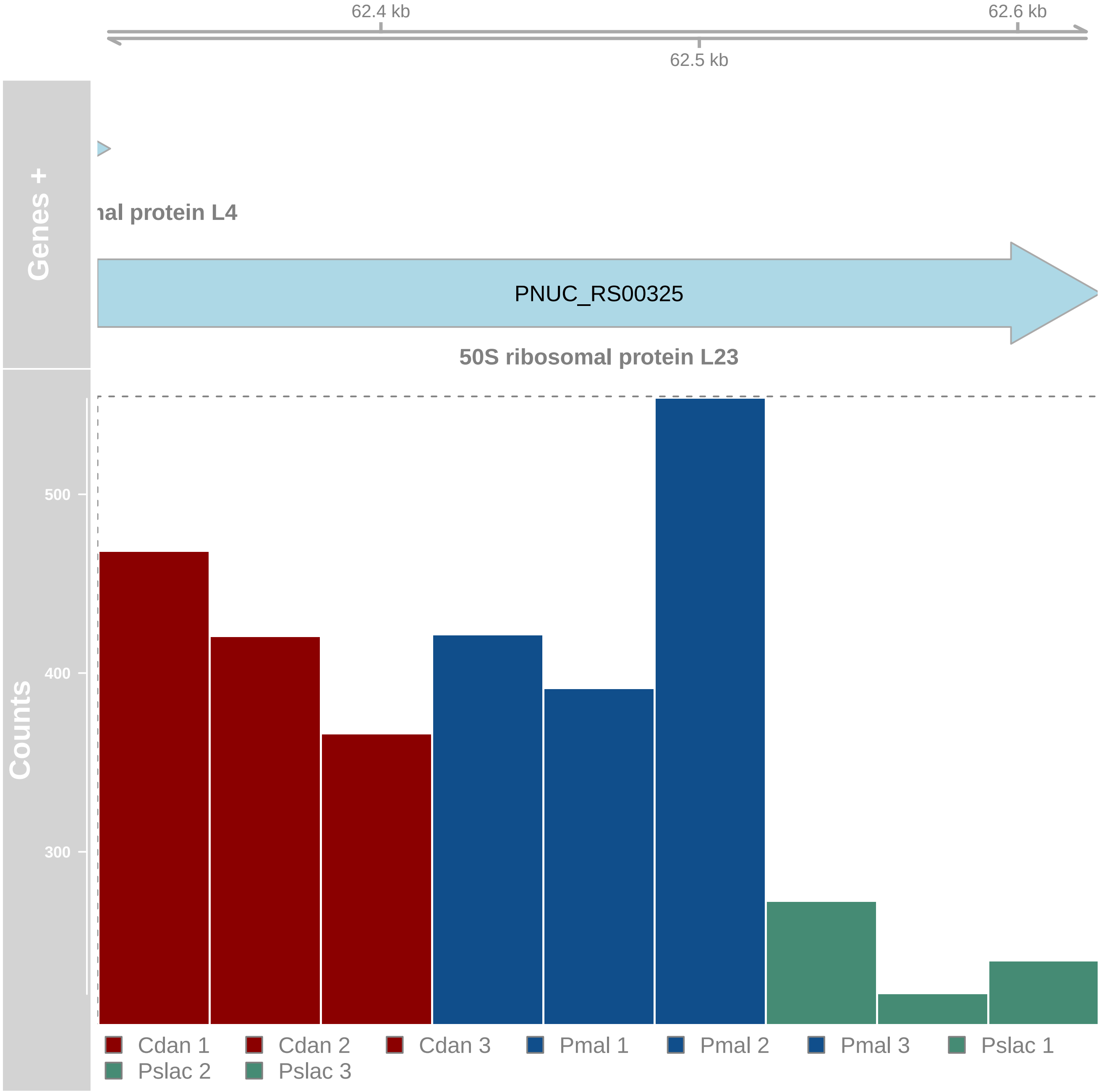
62.4 kb
62.6 kb
62.5 kb
Genes +
nal protein L4
PNUC_RS00325
50S ribosomal protein L23
Counts
500
400
300
Cdan 1
Cdan 2
Cdan 3
Pmal 1
Pmal 2
Pmal 3
Pslac 1
Pslac 2
Pslac 3

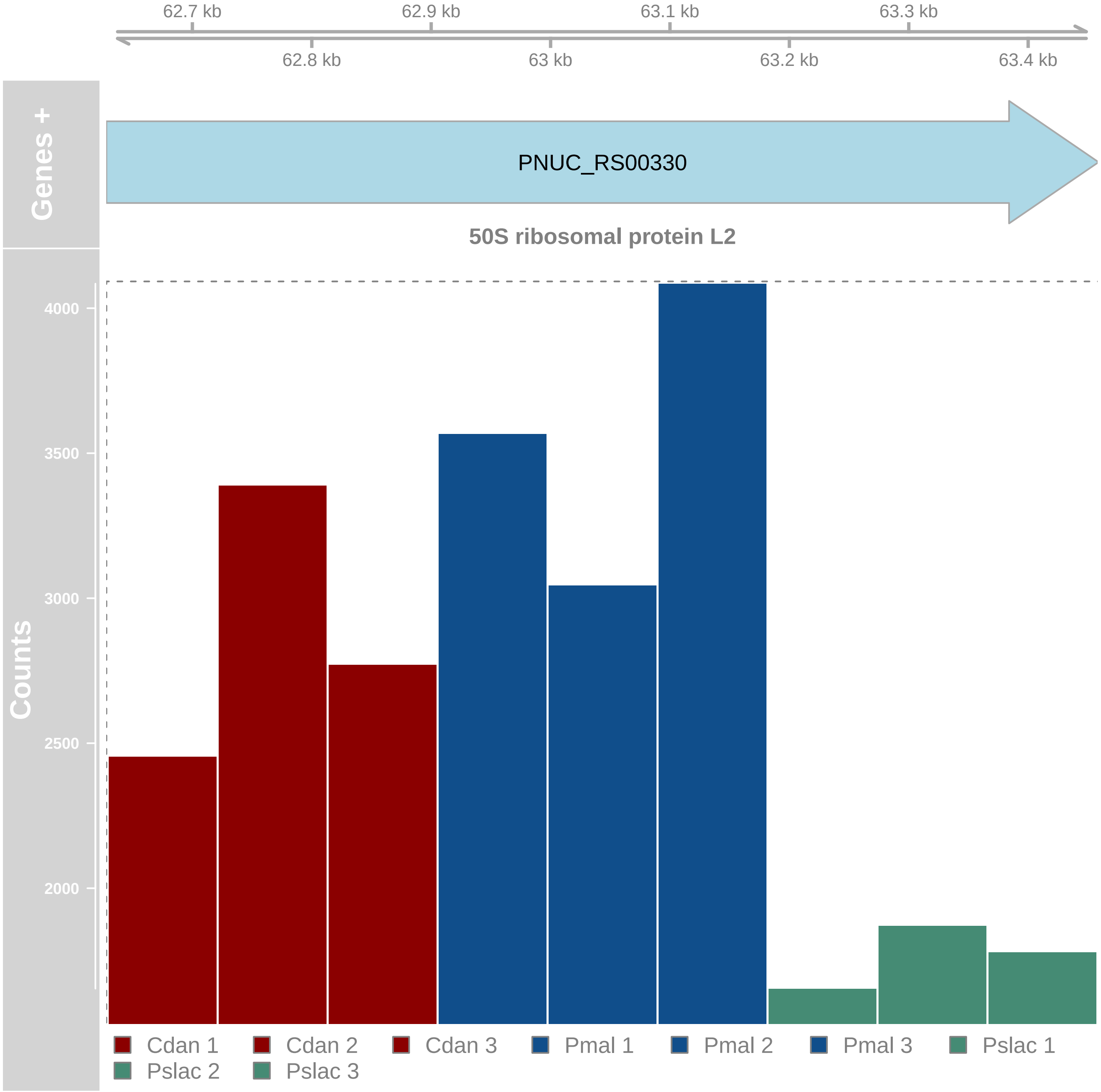

62.7 kb
62.9 kb
63.1 kb
63.3 kb
62.8 kb
63 kb
63.2 kb
63.4 kb
Genes +
PNUC_RS00330
50S ribosomal protein L2
Counts
4000
3500
3000
2500
2000
Cdan 1
Cdan 2
Cdan 3
Pmal 1
Pmal 2
Pmal 3
Pslac 1
Pslac 2
Pslac 3

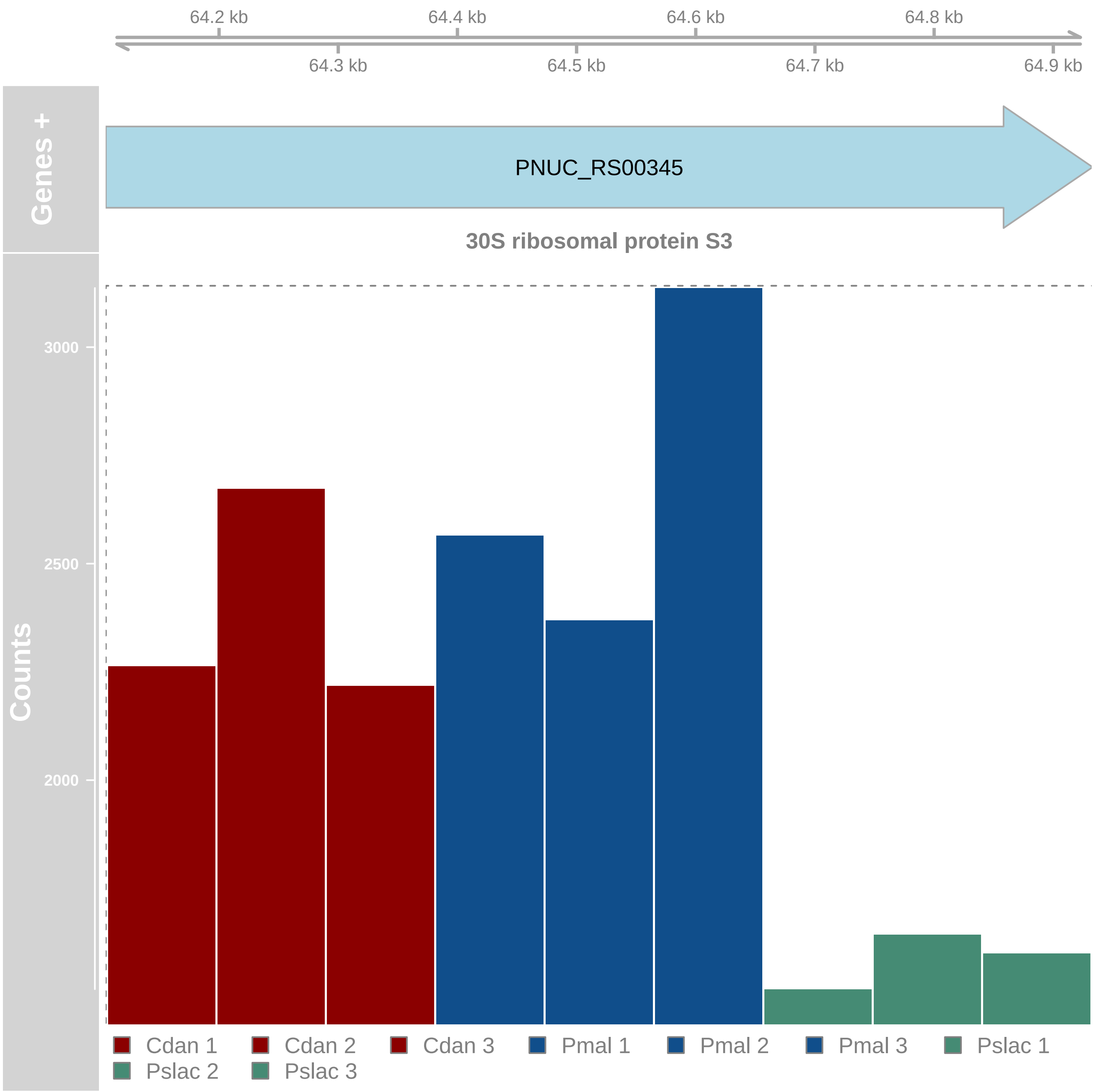
64.2 kb
64.4 kb
64.6 kb
64.8 kb
64.3 kb
64.5 kb
64.7 kb
64.9 kb
Genes +
PNUC_RS00345
30S ribosomal protein S3
Counts
3000
2500
2000
Cdan 1
Cdan 2
Cdan 3
Pmal 1
Pmal 2
Pmal 3
Pslac 1
Pslac 2
Pslac 3

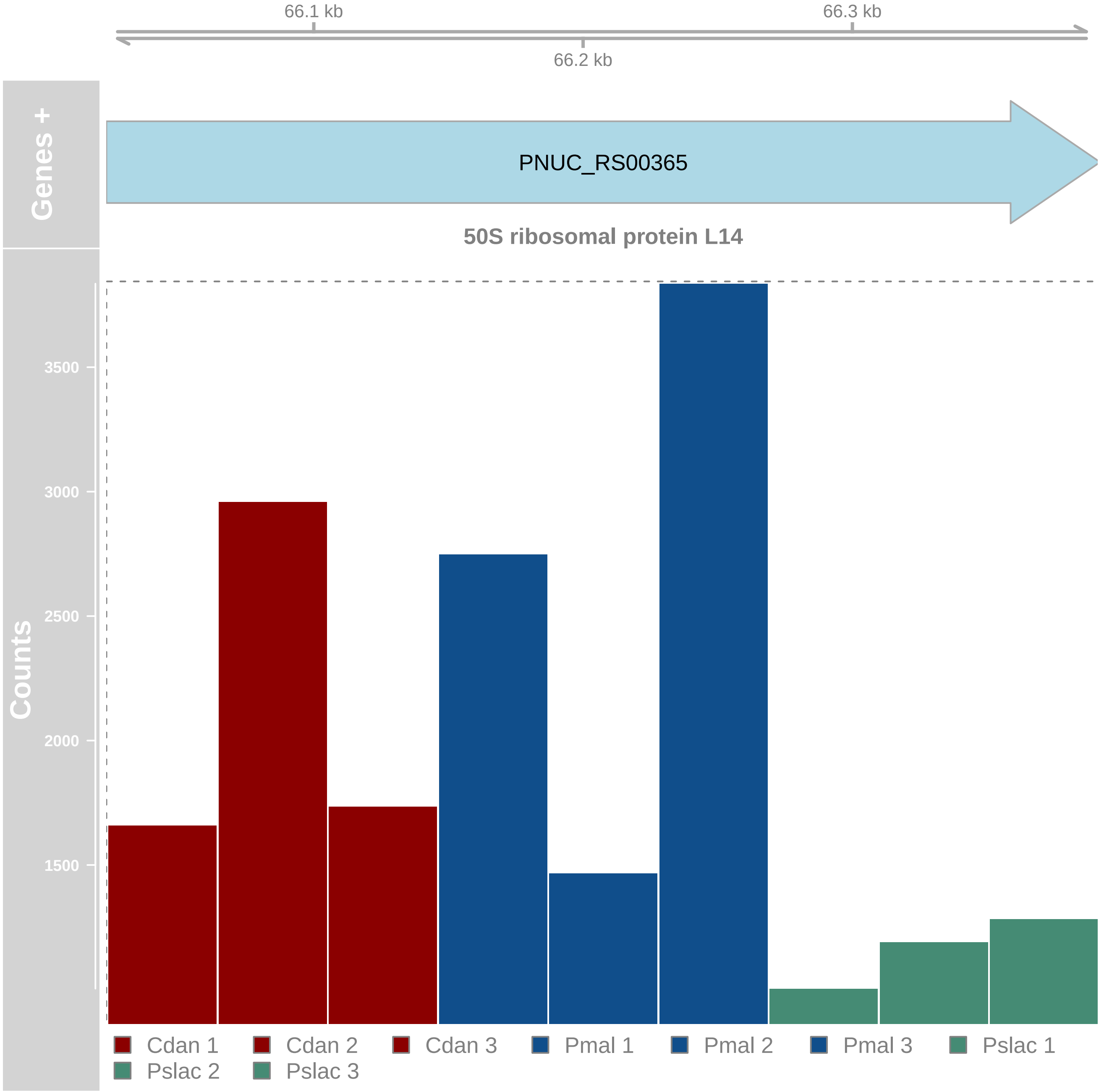
66.1 kb
66.2 kb
66.3 kb
Genes +
PNUC_RS00365
50S ribosomal protein L14
Counts
3500
3000
2500
2000
1500
Cdan 1
Cdan 2
Cdan 3
Pmal 1
Pmal 2
Pmal 3
Pslac 1
Pslac 2
Pslac 3

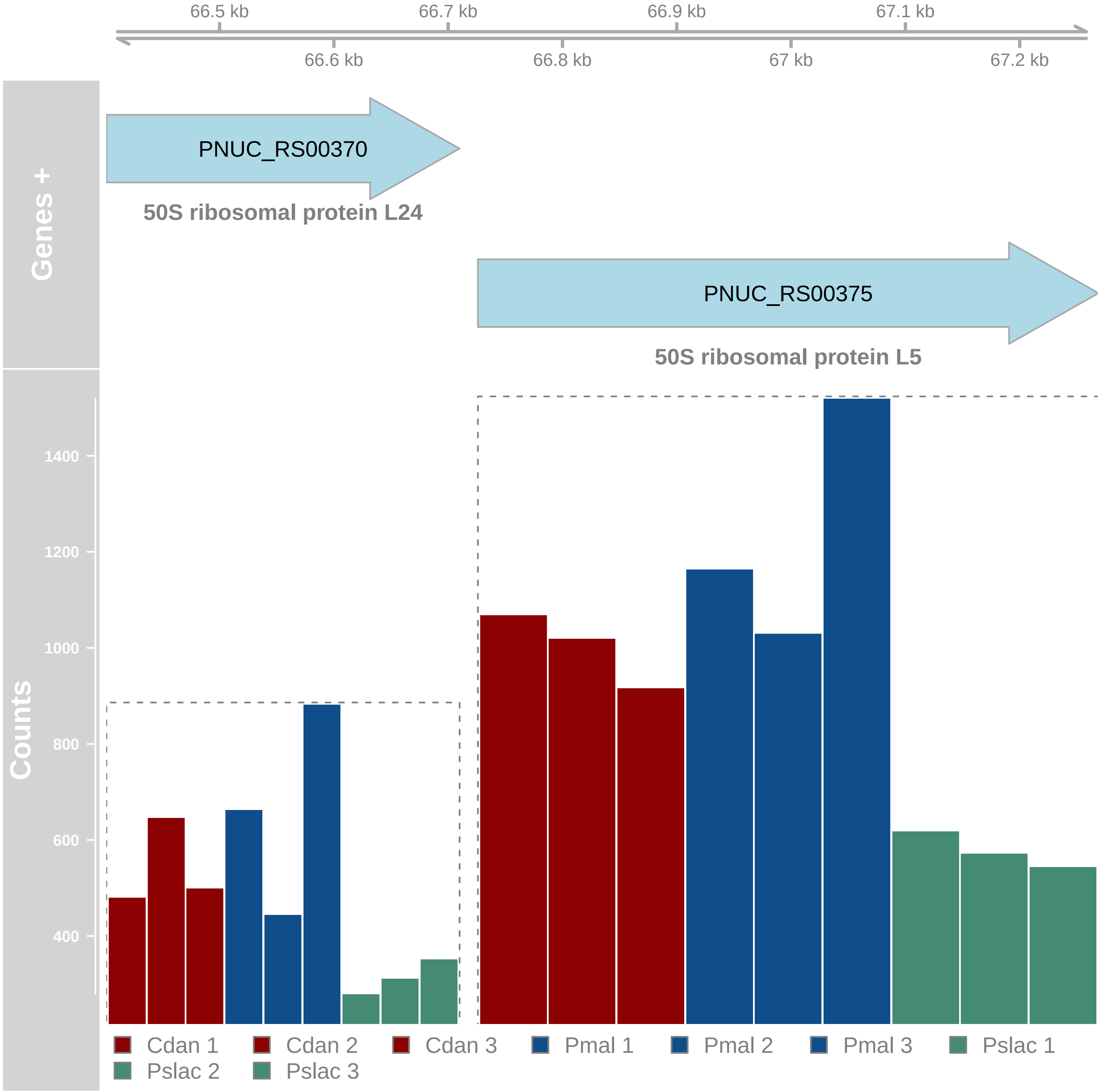
66.5 kb
66.7 kb
66.9 kb
67.1 kb
66.6 kb
66.8 kb
67 kb
67.2 kb
Genes +
PNUC_RS00370
50S ribosomal protein L24
PNUC_RS00375
50S ribosomal protein L5
Counts
1400
1200
1000
800
600
400
Cdan 1
Cdan 2
Cdan 3
Pmal 1
Pmal 2
Pmal 3
Pslac 1
Pslac 2
Pslac 3

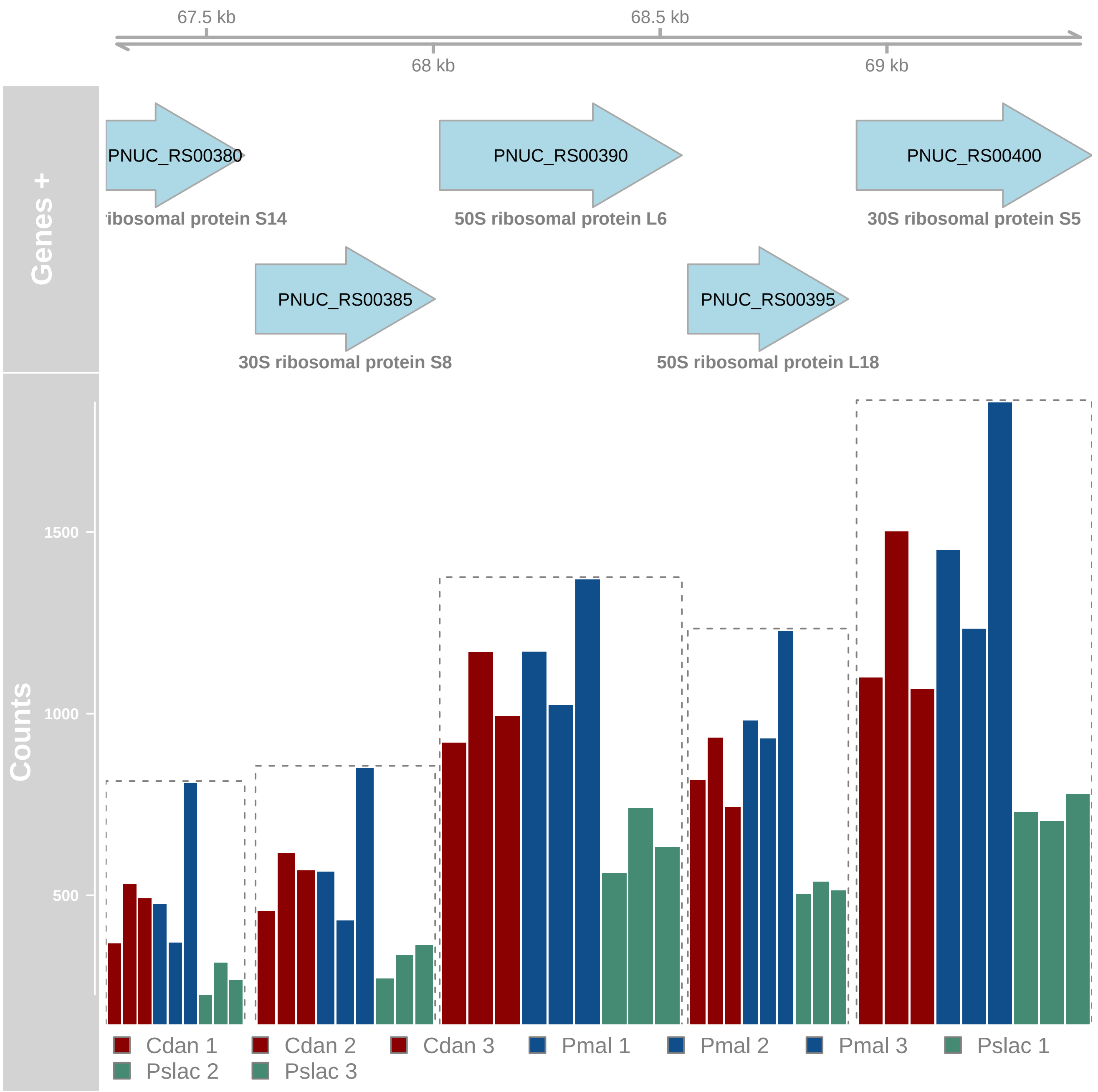

67.5 kb
68.5 kb
68 kb
69 kb
Genes +
PNUC_RS00380
ribosomal protein S14
PNUC_RS00390
50S ribosomal protein L6
PNUC_RS00400
30S ribosomal protein S5
PNUC_RS00385
30S ribosomal protein S8
PNUC_RS00395
50S ribosomal protein L18
Counts
1500
1000
500
Cdan 1
Cdan 2
Cdan 3
Pmal 1
Pmal 2
Pmal 3
Pslac 1
Pslac 2
Pslac 3

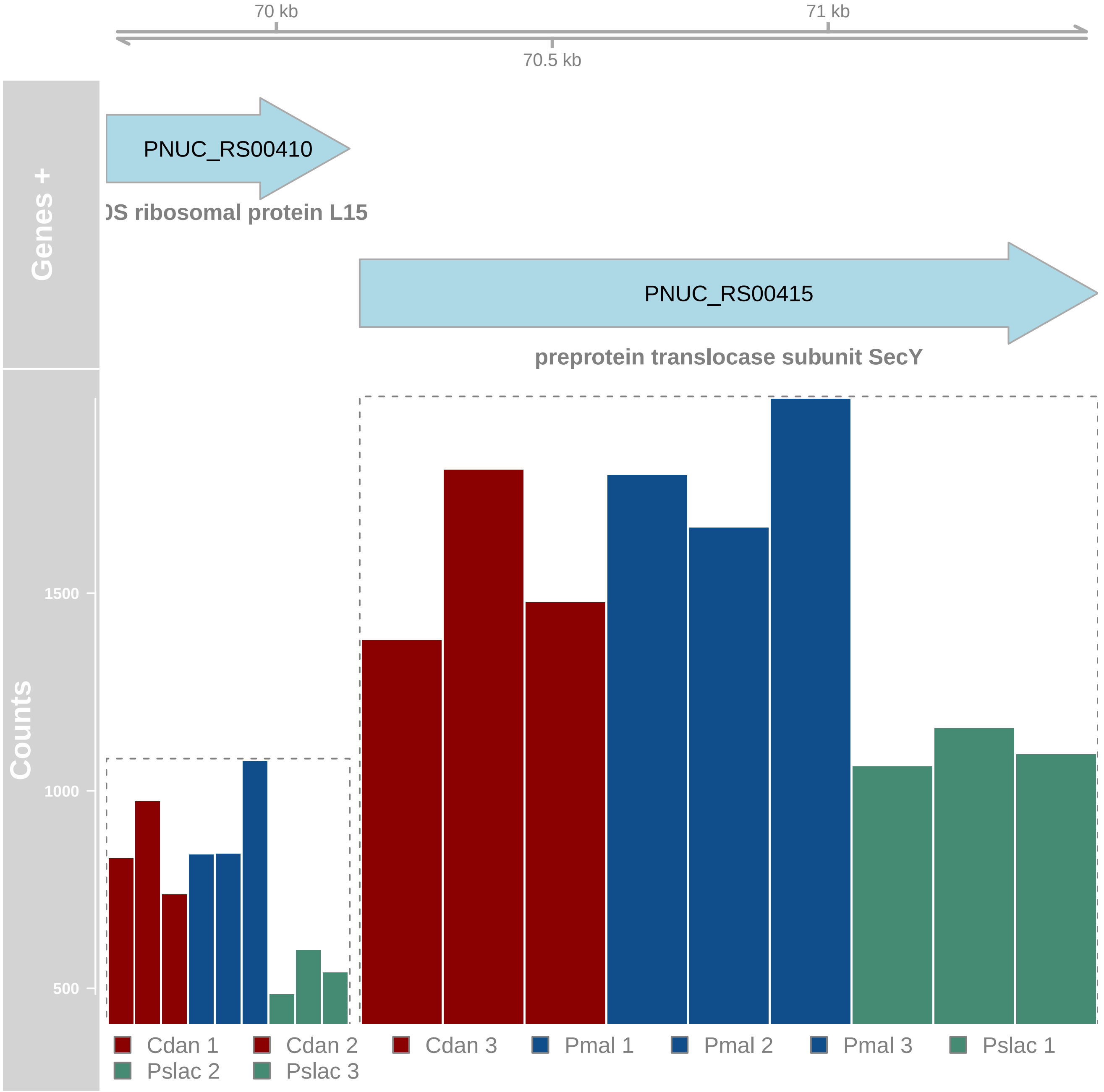
70 kb
71 kb
70.5 kb
Genes +
PNUC_RS00410
)S ribosomal protein L15
PNUC_RS00415
preprotein translocase subunit SecY
Counts
1500
1000
500
Cdan 1
Cdan 2
Cdan 3
Pmal 1
Pmal 2
Pmal 3
Pslac 1
Pslac 2
Pslac 3

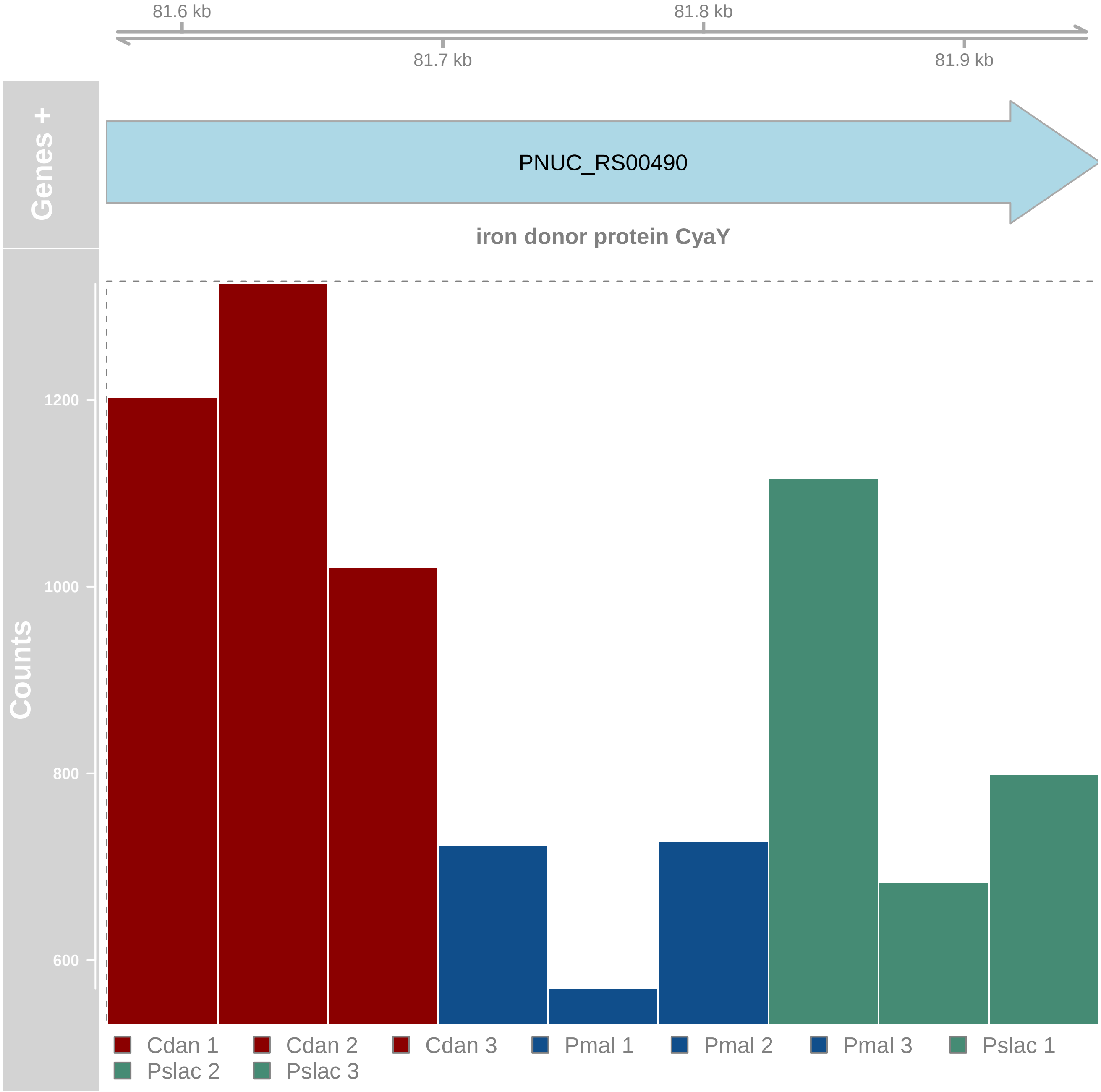

81.6 kb
81.8 kb
81.7 kb
81.9 kb
Genes +
PNUC_RS00490
iron donor protein CyaY
Counts
1200
1000
800
600
Cdan 1
Cdan 2
Cdan 3
Pmal 1
Pmal 2
Pmal 3
Pslac 1
Pslac 2
Pslac 3

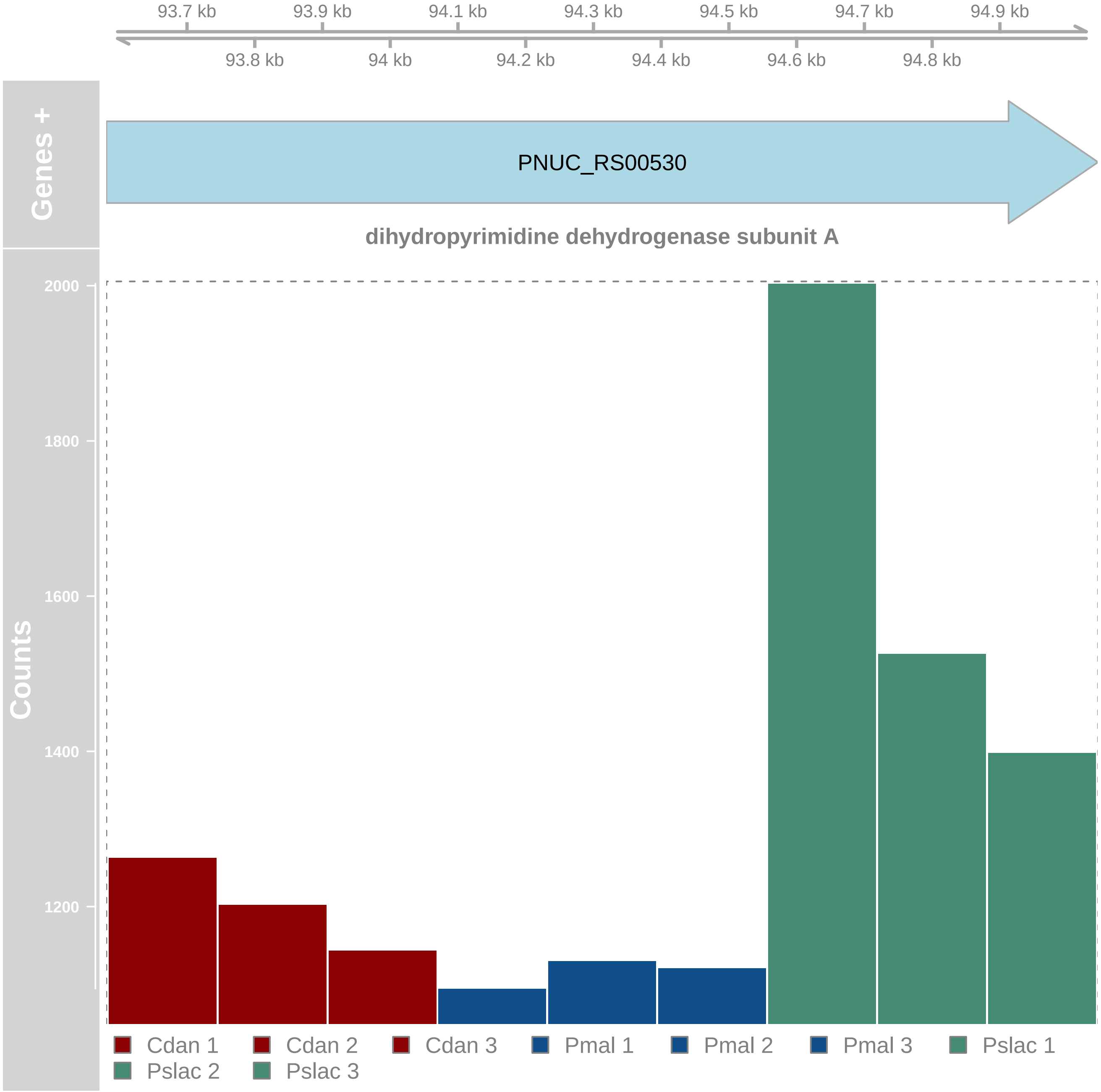
93.7 kb
93.9 kb
94.1 kb
94.3 kb
94.5 kb
94.7 kb
94.9 kb
93.8 kb
94 kb
94.2 kb
94.4 kb
94.6 kb
94.8 kb
Genes +
PNUC_RS00530
dihydropyrimidine dehydrogenase subunit A
Counts
2000
1800
1600
1400
1200
Cdan 1
Cdan 2
Cdan 3
Pmal 1
Pmal 2
Pmal 3
Pslac 1
Pslac 2
Pslac 3

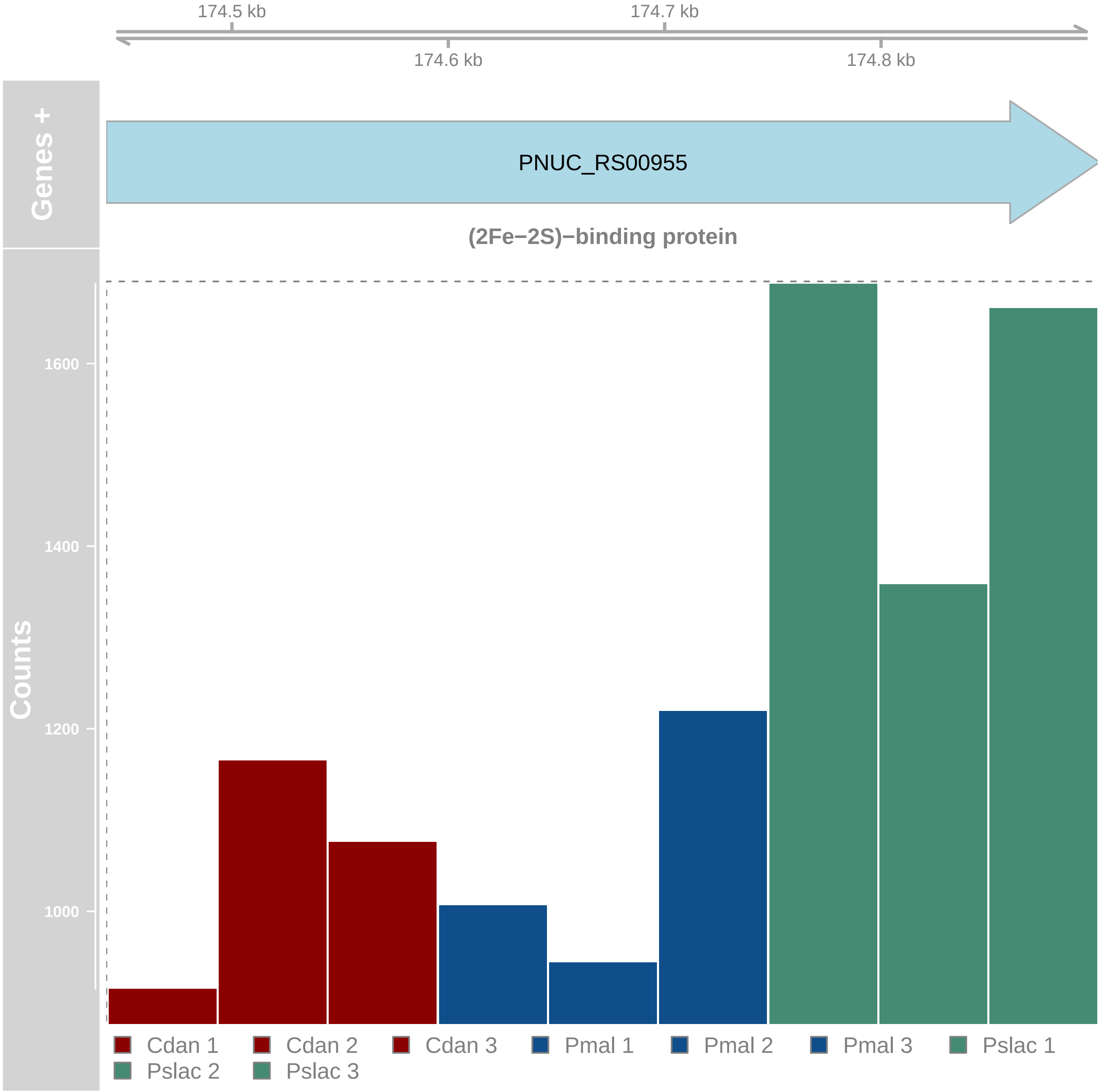

174.5 kb
174.7 kb
174.6 kb
174.8 kb
Genes +
PNUC_RS00955
(2Fe−2S)−binding protein
Counts
1600
1400
1200
1000
Cdan 1
Cdan 2
Cdan 3
Pmal 1
Pmal 2
Pmal 3
Pslac 1
Pslac 2
Pslac 3

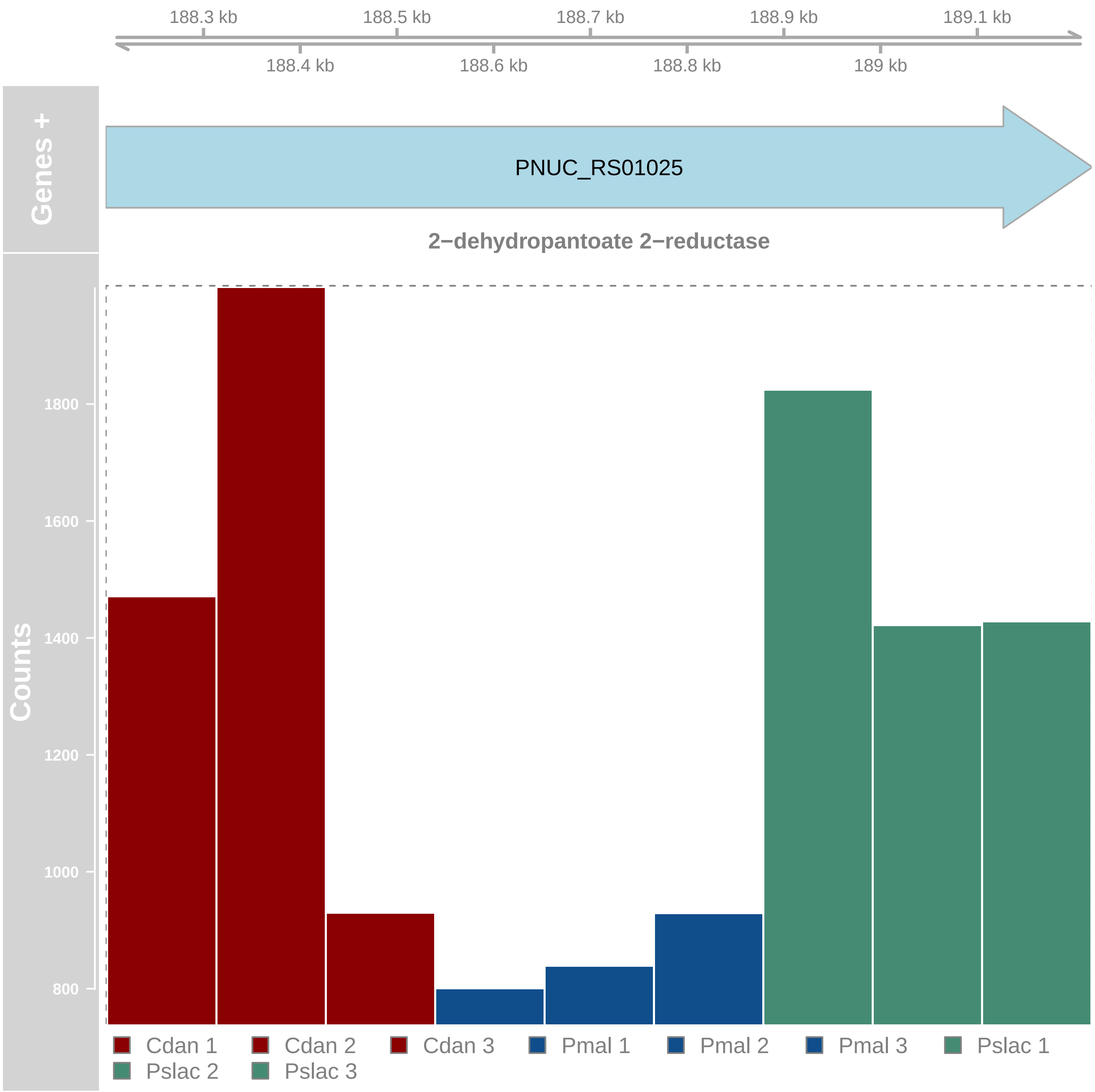

188.3 kb
188.5 kb
188.7 kb
188.9 kb
189.1 kb
188.4 kb
188.6 kb
188.8 kb
189 kb
Genes +
PNUC_RS01025
2−dehydropantoate 2−reductase
Counts
1800
1600
1400
1200
1000
800
Cdan 1
Cdan 2
Cdan 3
Pmal 1
Pmal 2
Pmal 3
Pslac 1
Pslac 2
Pslac 3

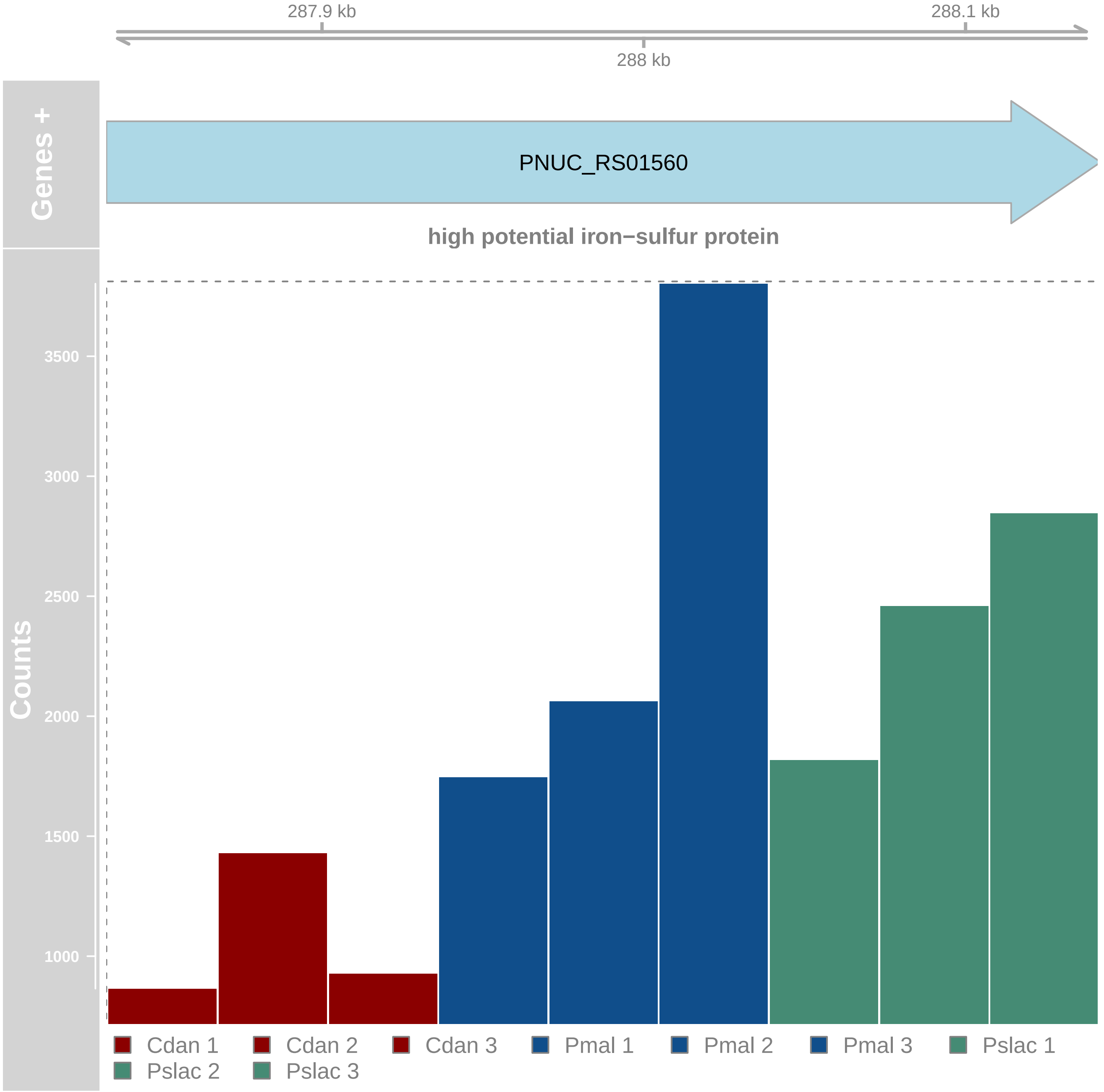
287.9 kb
288 kb
288.1 kb
Genes +
PNUC_RS01560
high potential iron−sulfur protein
Counts
3500
3000
2500
2000
1500
1000
Cdan 1
Cdan 2
Cdan 3
Pmal 1
Pmal 2
Pmal 3
Pslac 1
Pslac 2
Pslac 3

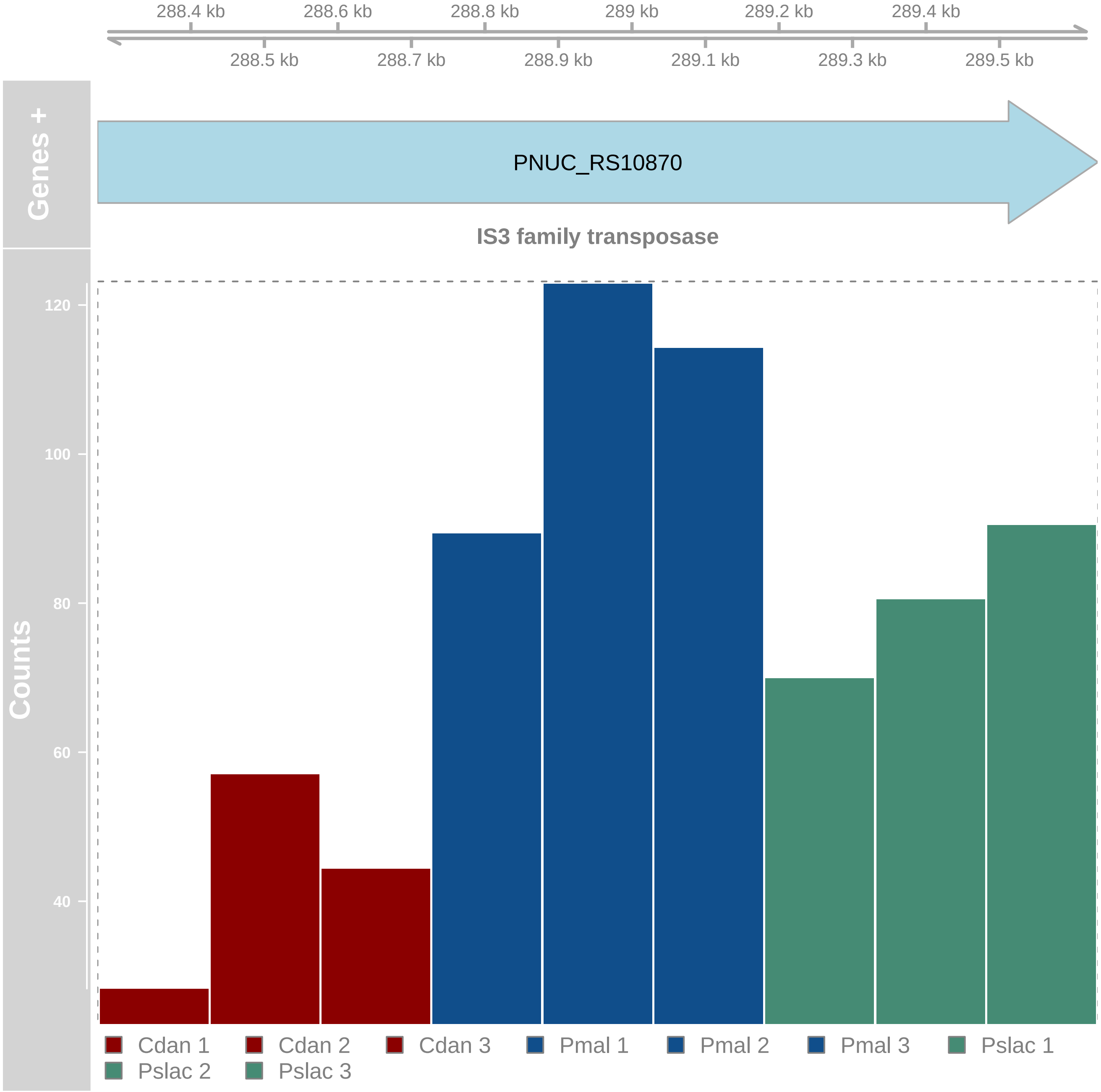
288.4 kb
288.6 kb
288.8 kb
289 kb
289.2 kb
289.4 kb
288.5 kb
288.7 kb
288.9 kb
289.1 kb
289.3 kb
289.5 kb
Genes +
PNUC_RS10870
IS3 family transposase
Counts
120
100
80
60
40
Cdan 1
Cdan 2
Cdan 3
Pmal 1
Pmal 2
Pmal 3
Pslac 1
Pslac 2
Pslac 3

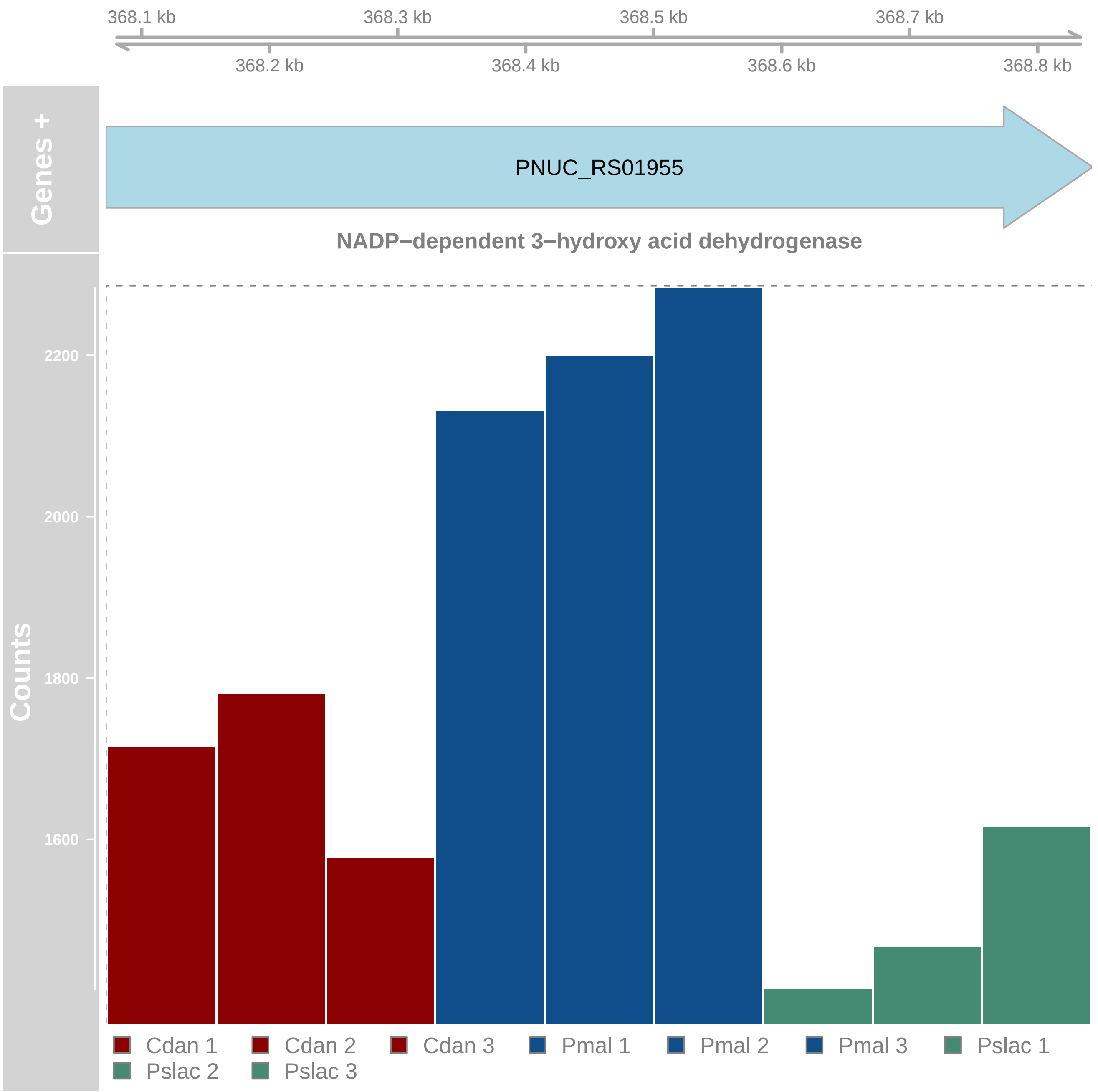
368.1 kb
368.2 kb
368.3 kb
368.4 kb
368.5 kb
368.6 kb
368.7 kb
368.8 kb
Genes +
PNUC_RS01955
NADP−dependent 3−hydroxy acid dehydrogenase
Counts
2200
2000
1800
1600
Cdan 1
Cdan 2
Cdan 3
Pmal 1
Pmal 2
Pmal 3
Pslac 1
Pslac 2
Pslac 3

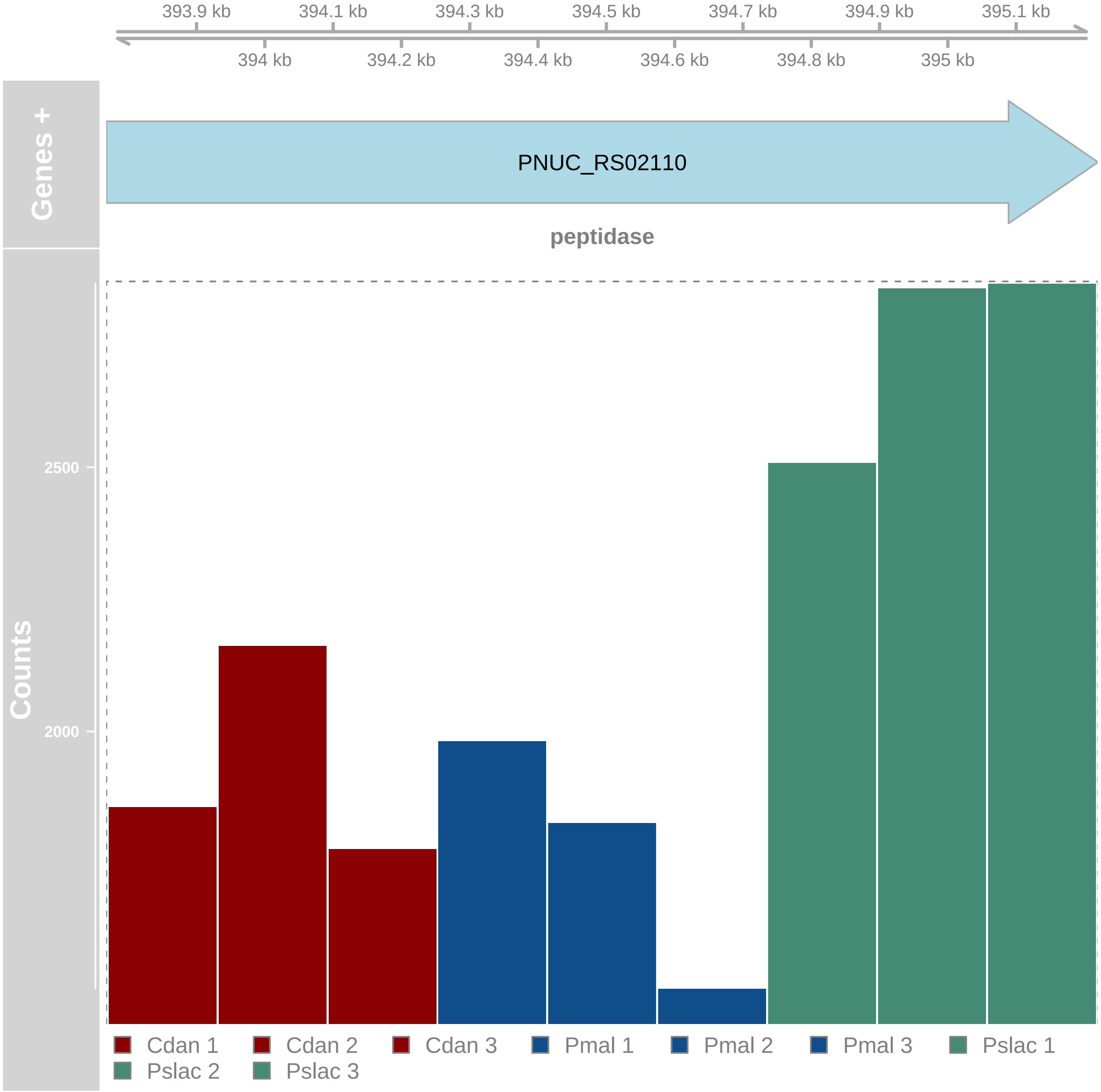

393.9 kb
394.1 kb
394.3 kb
394.5 kb
394.7 kb
394.9 kb
395.1 kb
394 kb
394.2 kb
394.4 kb
394.6 kb
394.8 kb
395 kb
Genes +
PNUC_RS02110
peptidase
Counts
2500
2000
Cdan 1
Cdan 2
Cdan 3
Pmal 1
Pmal 2
Pmal 3
Pslac 1
Pslac 2
Pslac 3

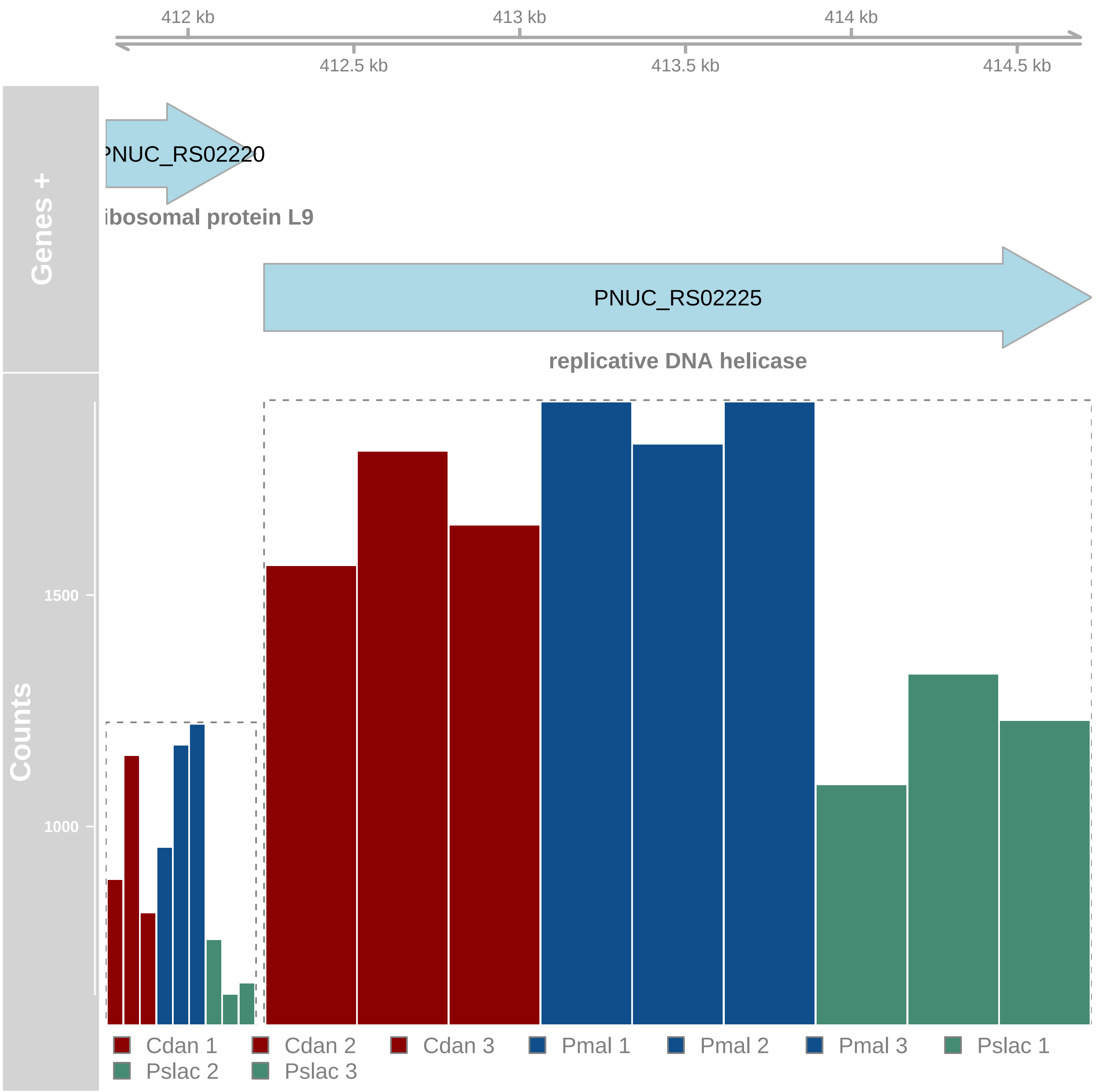
412 kb
413 kb
414 kb
412.5 kb
413.5 kb
414.5 kb
Genes +
PNUC_RS02220
ibosomal protein L9
PNUC_RS02225
replicative DNA helicase
Counts
1500
1000
Cdan 1
Cdan 2
Cdan 3
Pmal 1
Pmal 2
Pmal 3
Pslac 1
Pslac 2
Pslac 3

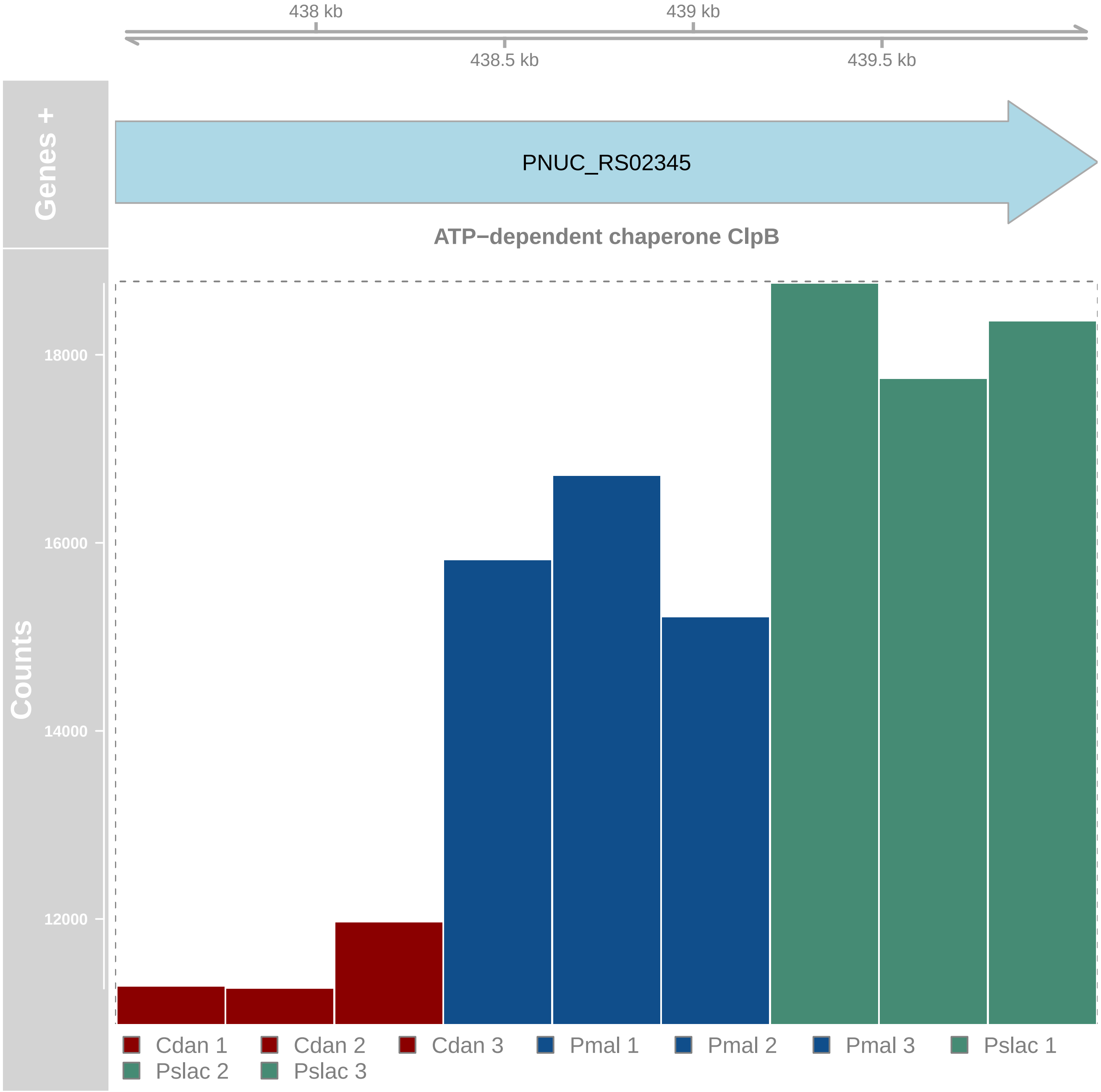

438 kb
439 kb
438.5 kb
439.5 kb
Genes +
PNUC_RS02345
ATP−dependent chaperone ClpB
Counts
18000
16000
14000
12000
Cdan 1
Cdan 2
Cdan 3
Pmal 1
Pmal 2
Pmal 3
Pslac 1
Pslac 2
Pslac 3

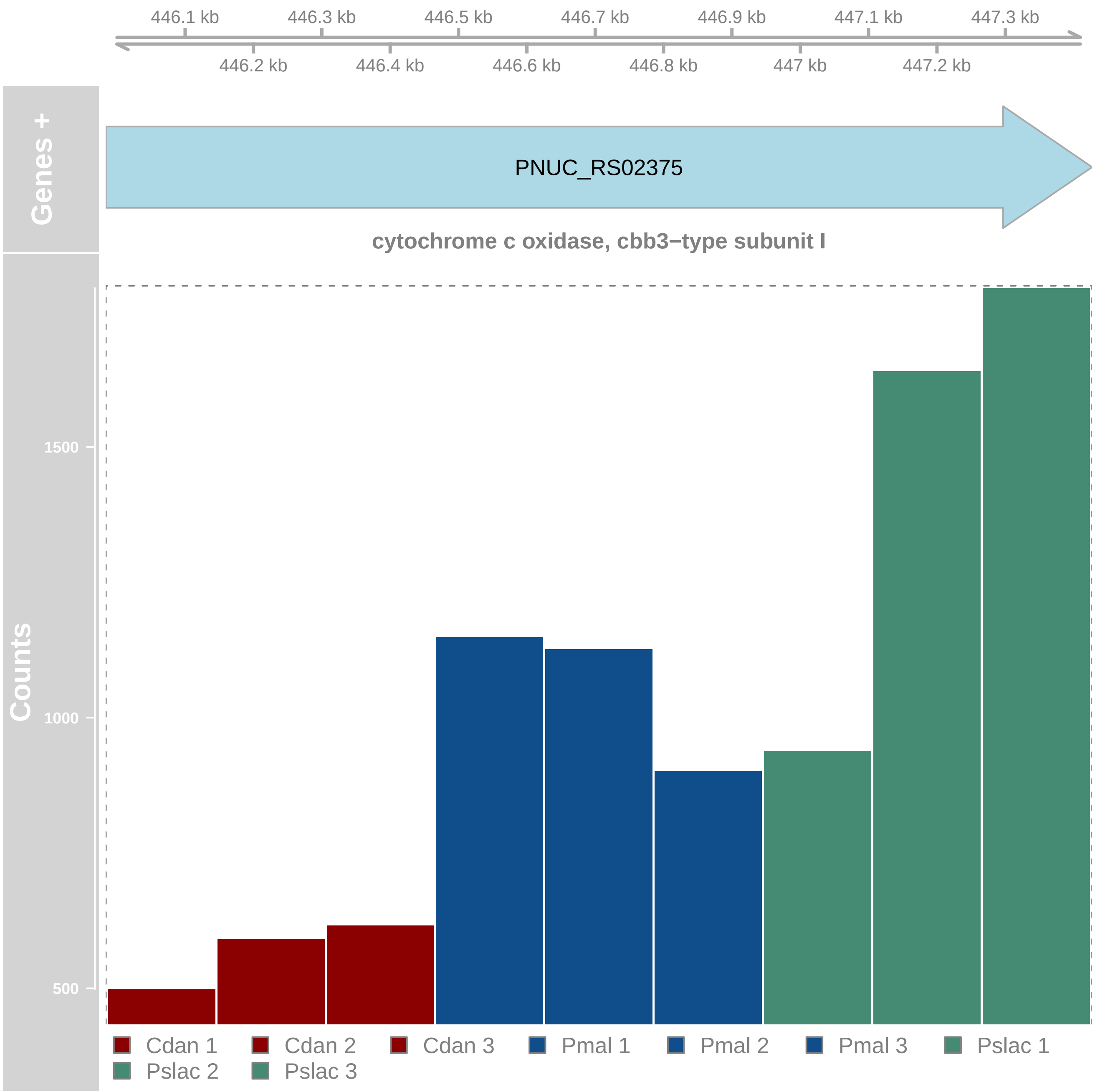

446.1 kb
446.3 kb
446.5 kb
446.7 kb
446.9 kb
447.1 kb
447.3 kb
446.2 kb
446.4 kb
446.6 kb
446.8 kb
447 kb
447.2 kb
Genes +
PNUC_RS02375
cytochrome c oxidase, cbb3−type subunit I
Counts
1500
1000
500
Cdan 1
Cdan 2
Cdan 3
Pmal 1
Pmal 2
Pmal 3
Pslac 1
Pslac 2
Pslac 3

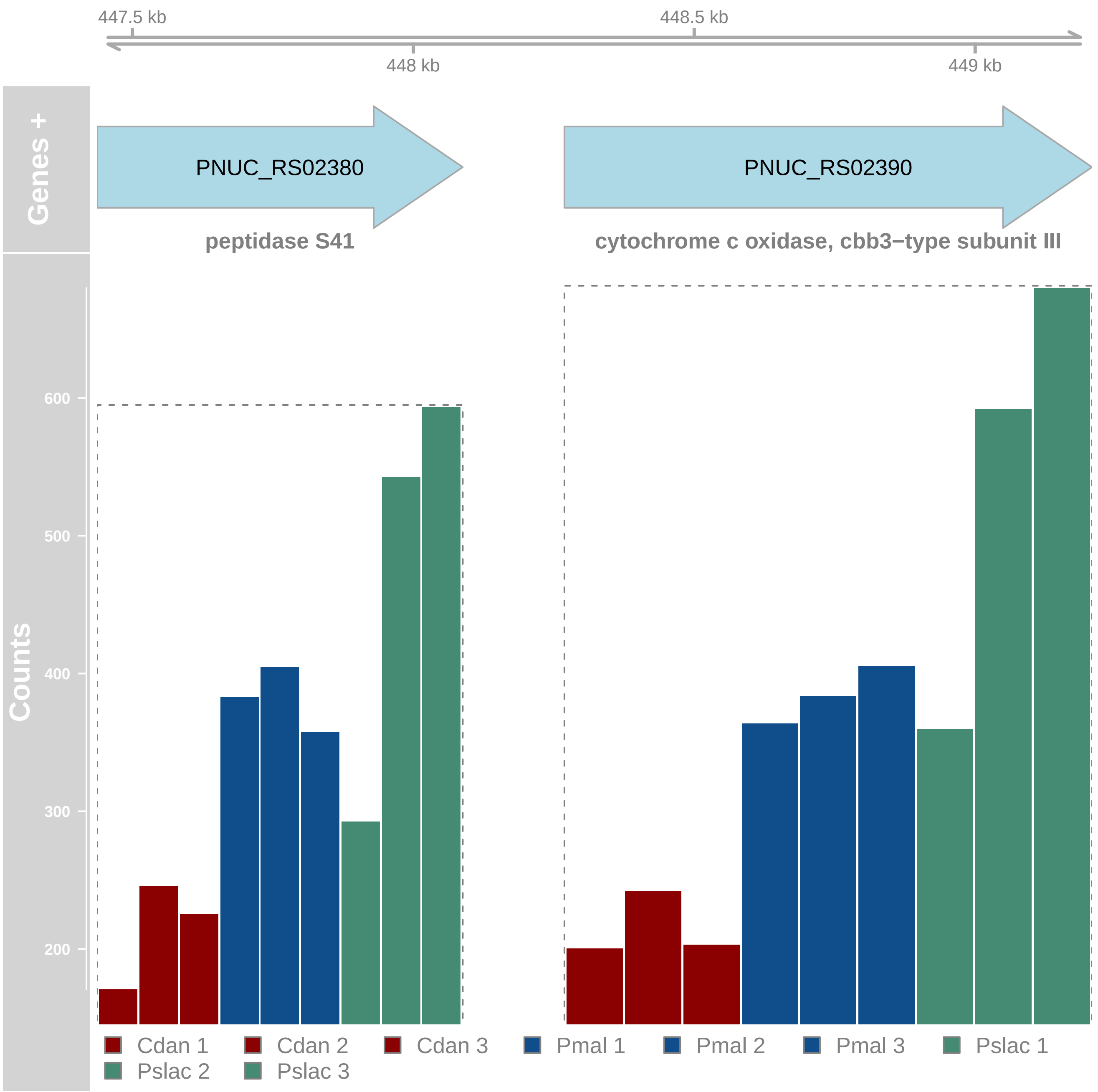

447.5 kb
448.5 kb
448 kb
449 kb
Genes +
PNUC_RS02380
PNUC_RS02390
peptidase S41
cytochrome c oxidase, cbb3−type subunit III
Counts
600
500
400
300
200
Cdan 1
Cdan 2
Cdan 3
Pmal 1
Pmal 2
Pmal 3
Pslac 1
Pslac 2
Pslac 3

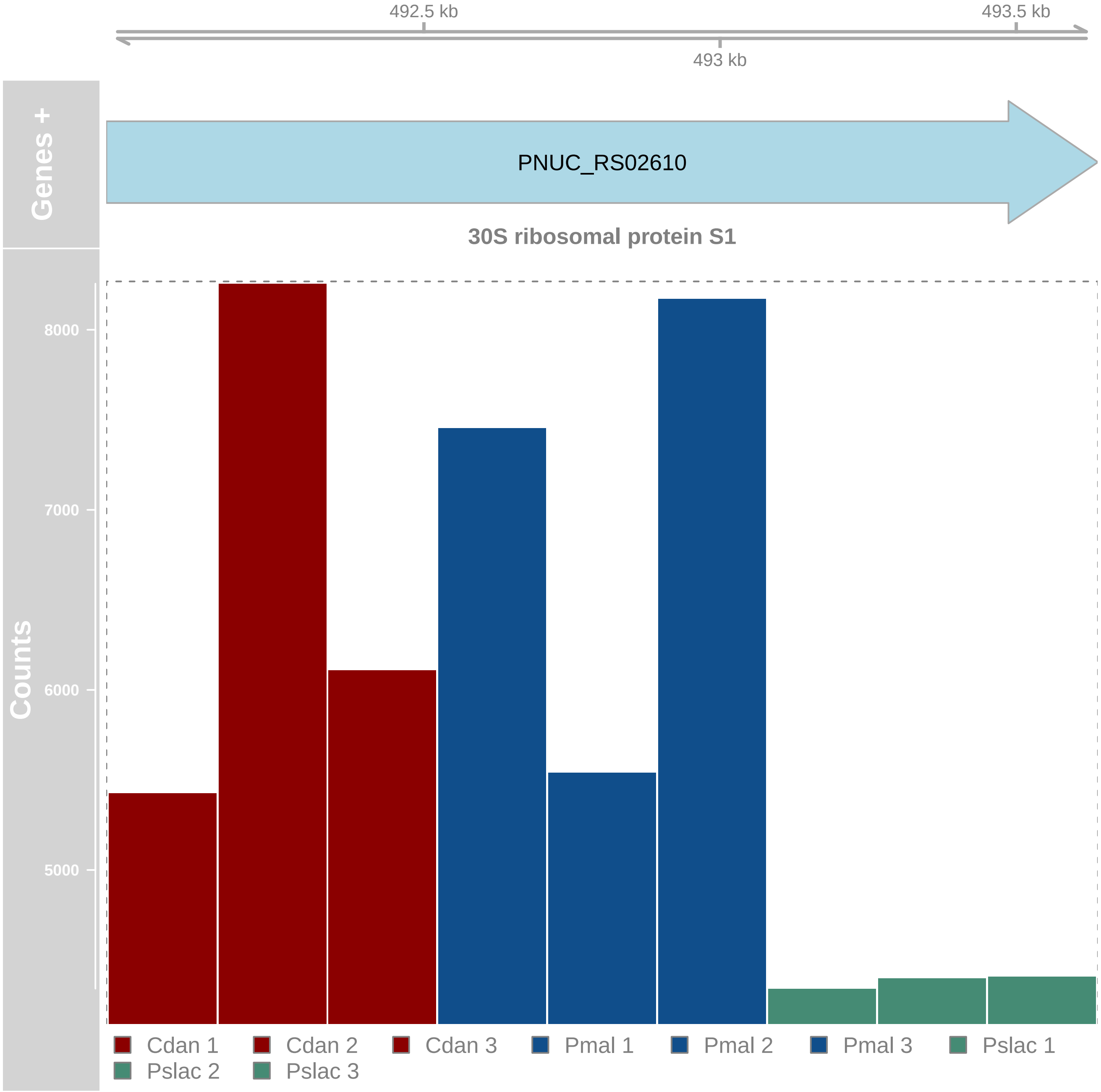
492.5 kb
493 kb
493.5 kb
Genes +
PNUC_RS02610
30S ribosomal protein S1
Counts
8000
7000
6000
5000
Cdan 1
Cdan 2
Cdan 3
Pmal 1
Pmal 2
Pmal 3
Pslac 1
Pslac 2
Pslac 3

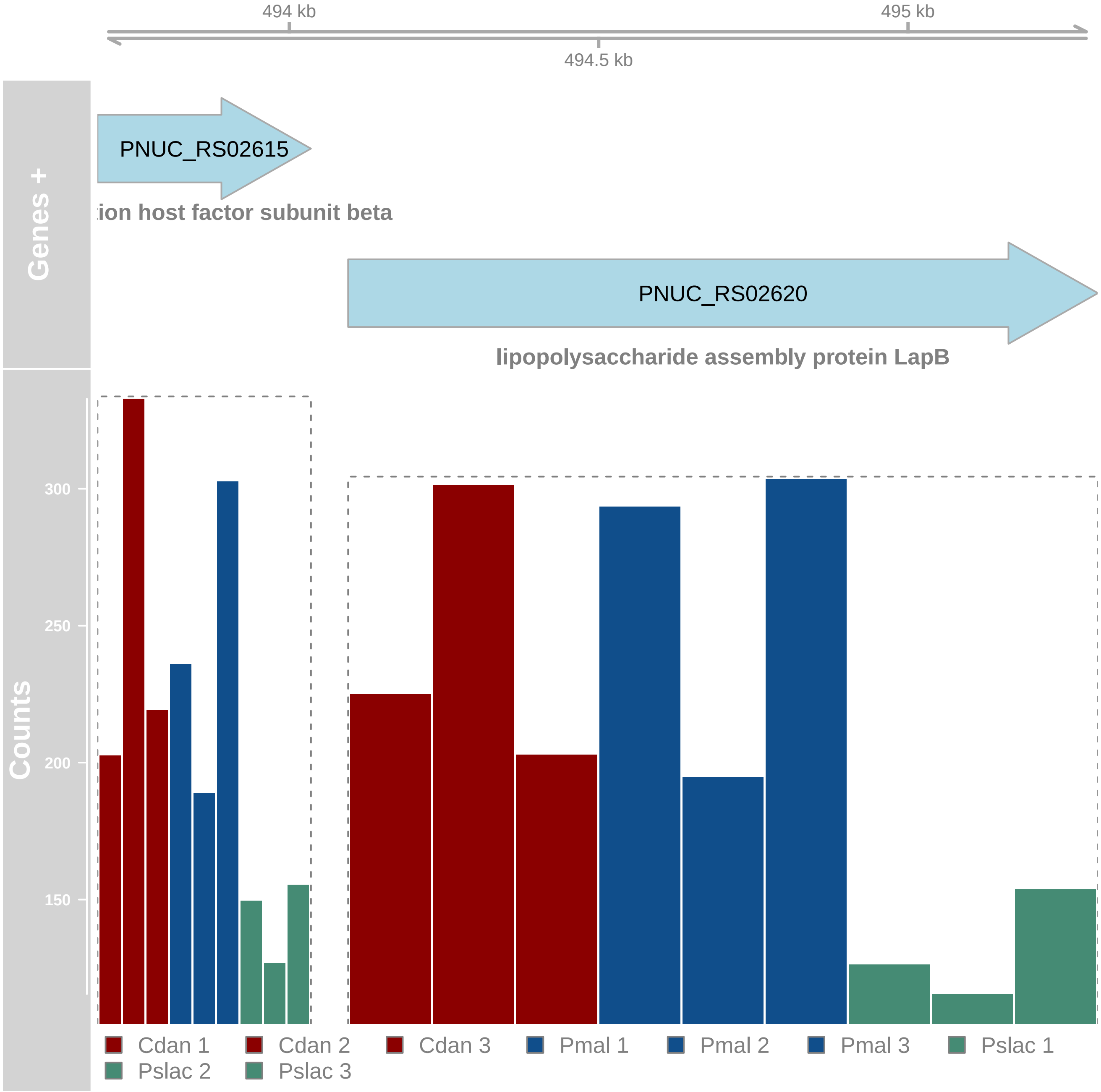

494 kb
495 kb
494.5 kb
Genes +
PNUC_RS02615
tion host factor subunit beta
PNUC_RS02620
lipopolysaccharide assembly protein LapB
Counts
300
250
200
150
Cdan 1
Cdan 2
Cdan 3
Pmal 1
Pmal 2
Pmal 3
Pslac 1
Pslac 2
Pslac 3

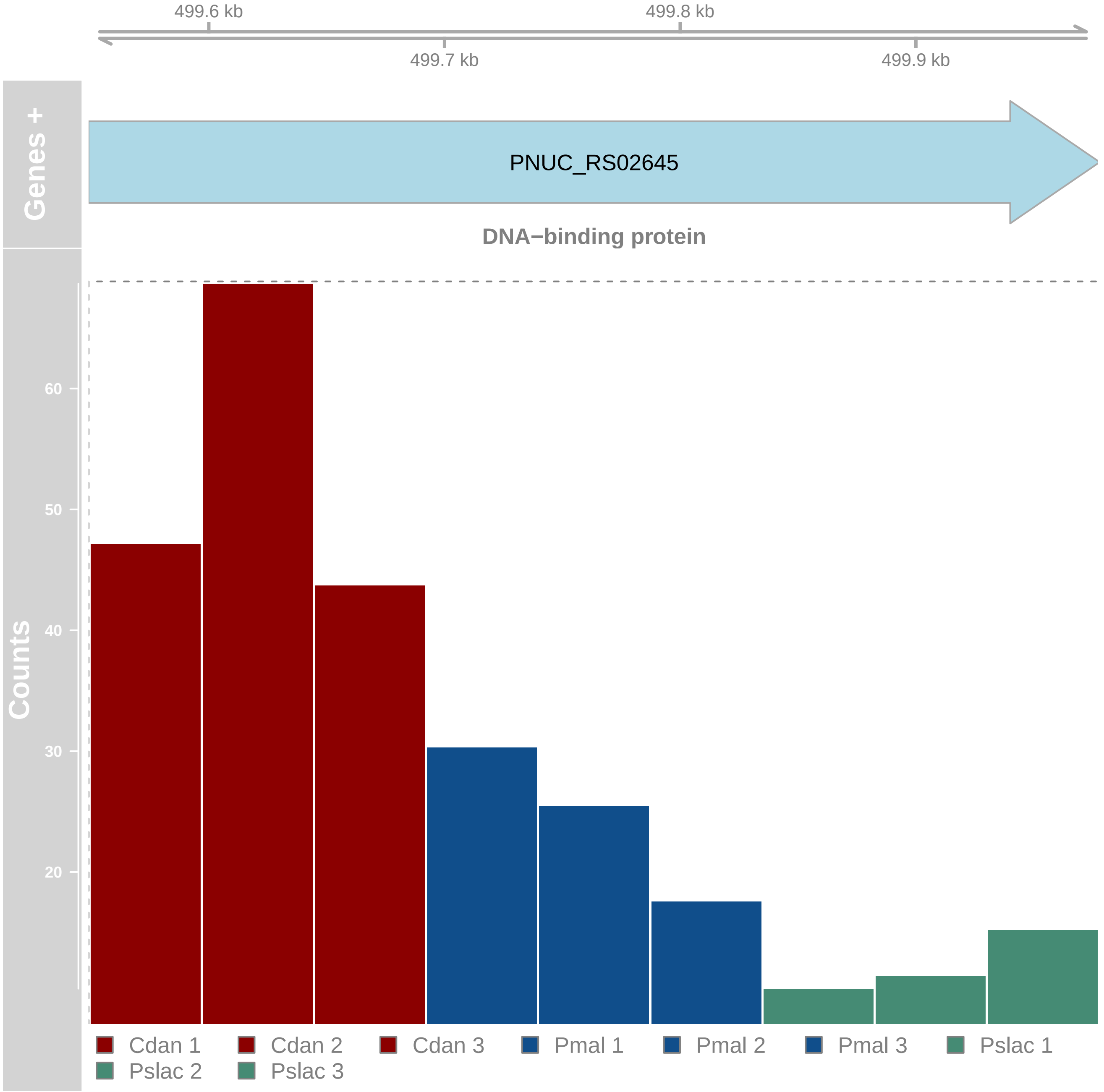
499.6 kb
499.8 kb
499.7 kb
499.9 kb
Genes +
PNUC_RS02645
DNA−binding protein
Counts
60
50
40
30
20
Cdan 1
Cdan 2
Cdan 3
Pmal 1
Pmal 2
Pmal 3
Pslac 1
Pslac 2
Pslac 3

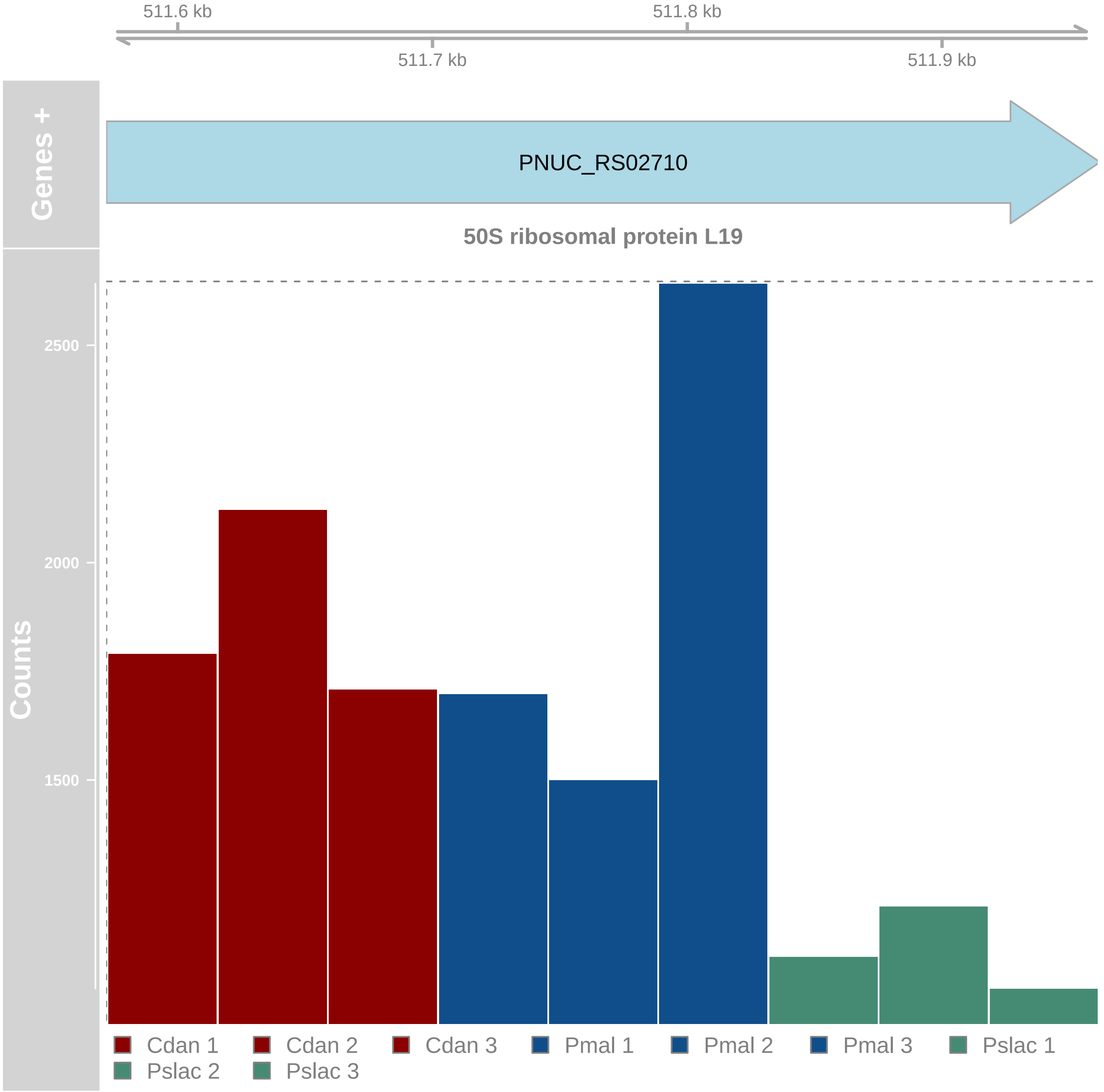
511.6 kb
511.8 kb
511.7 kb
511.9 kb
Genes +
PNUC_RS02710
50S ribosomal protein L19
Counts
2500
2000
1500
Cdan 1
Cdan 2
Cdan 3
Pmal 1
Pmal 2
Pmal 3
Pslac 1
Pslac 2
Pslac 3

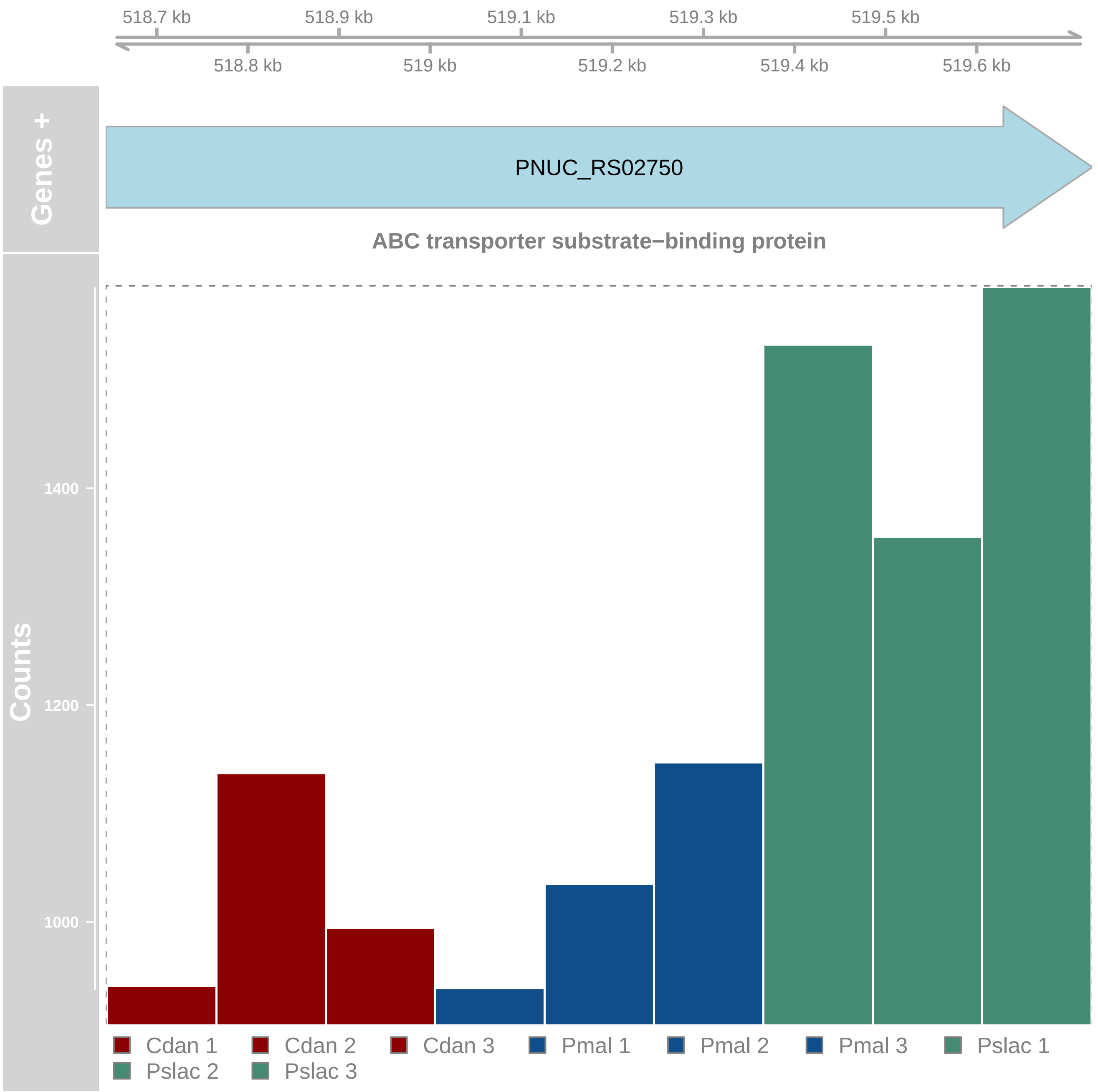

518.7 kb
518.9 kb
519.1 kb
519.3 kb
519.5 kb
518.8 kb
519 kb
519.2 kb
519.4 kb
519.6 kb
Genes +
PNUC_RS02750
ABC transporter substrate−binding protein
Counts
1400
1200
1000
Cdan 1
Cdan 2
Cdan 3
Pmal 1
Pmal 2
Pmal 3
Pslac 1
Pslac 2
Pslac 3

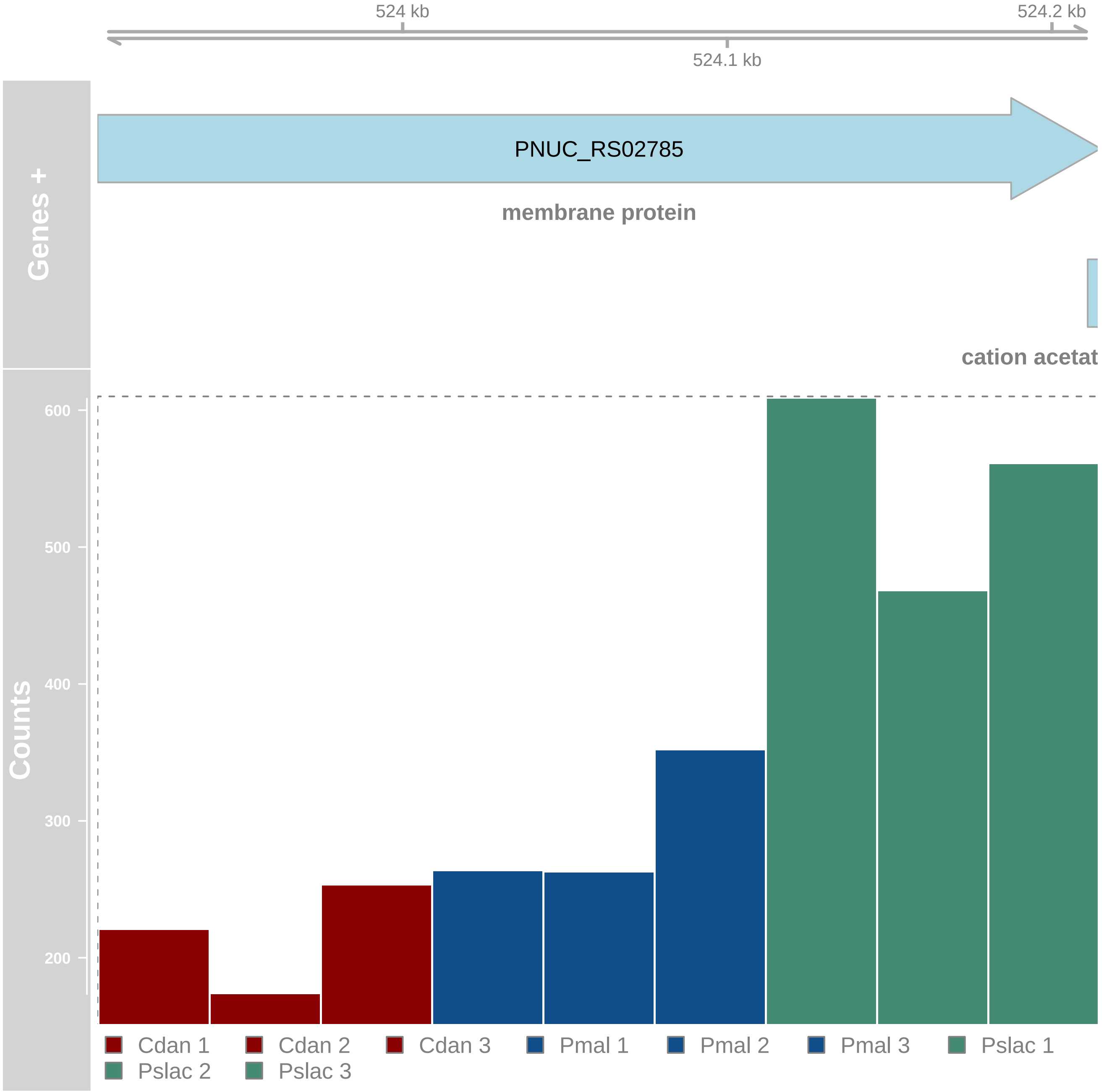
524 kb
524.2 kb
524.1 kb
Genes +
PNUC_RS02785
membrane protein
cation acetat
Counts
600
500
400
300
200
Cdan 1
Cdan 2
Cdan 3
Pmal 1
Pmal 2
Pmal 3
Pslac 1
Pslac 2
Pslac 3

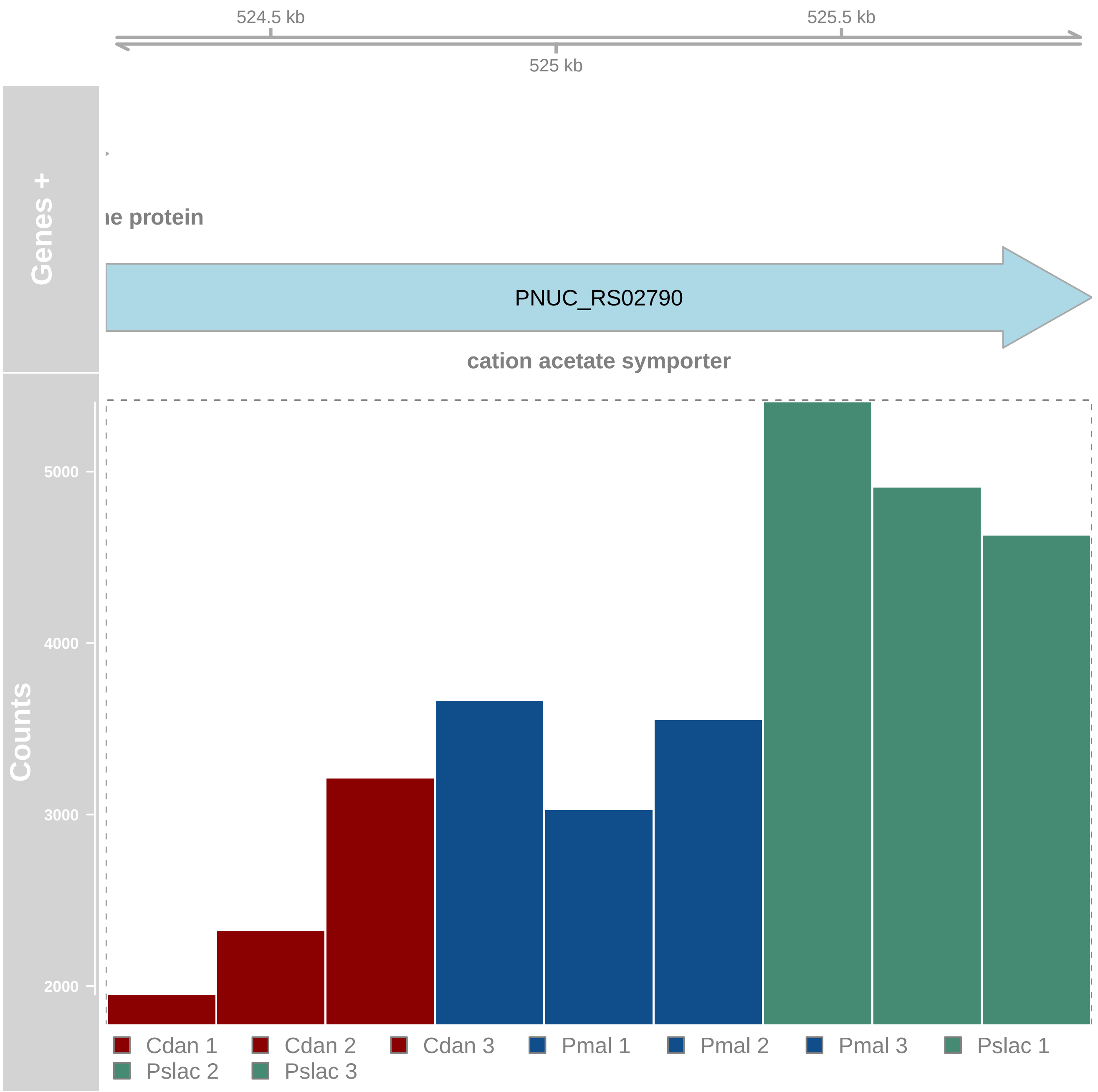

524.5 kb
525.5 kb
525 kb
Genes +
ne protein
PNUC_RS02790
cation acetate symporter
Counts
5000
4000
3000
2000
Cdan 1
Cdan 2
Cdan 3
Pmal 1
Pmal 2
Pmal 3
Pslac 1
Pslac 2
Pslac 3

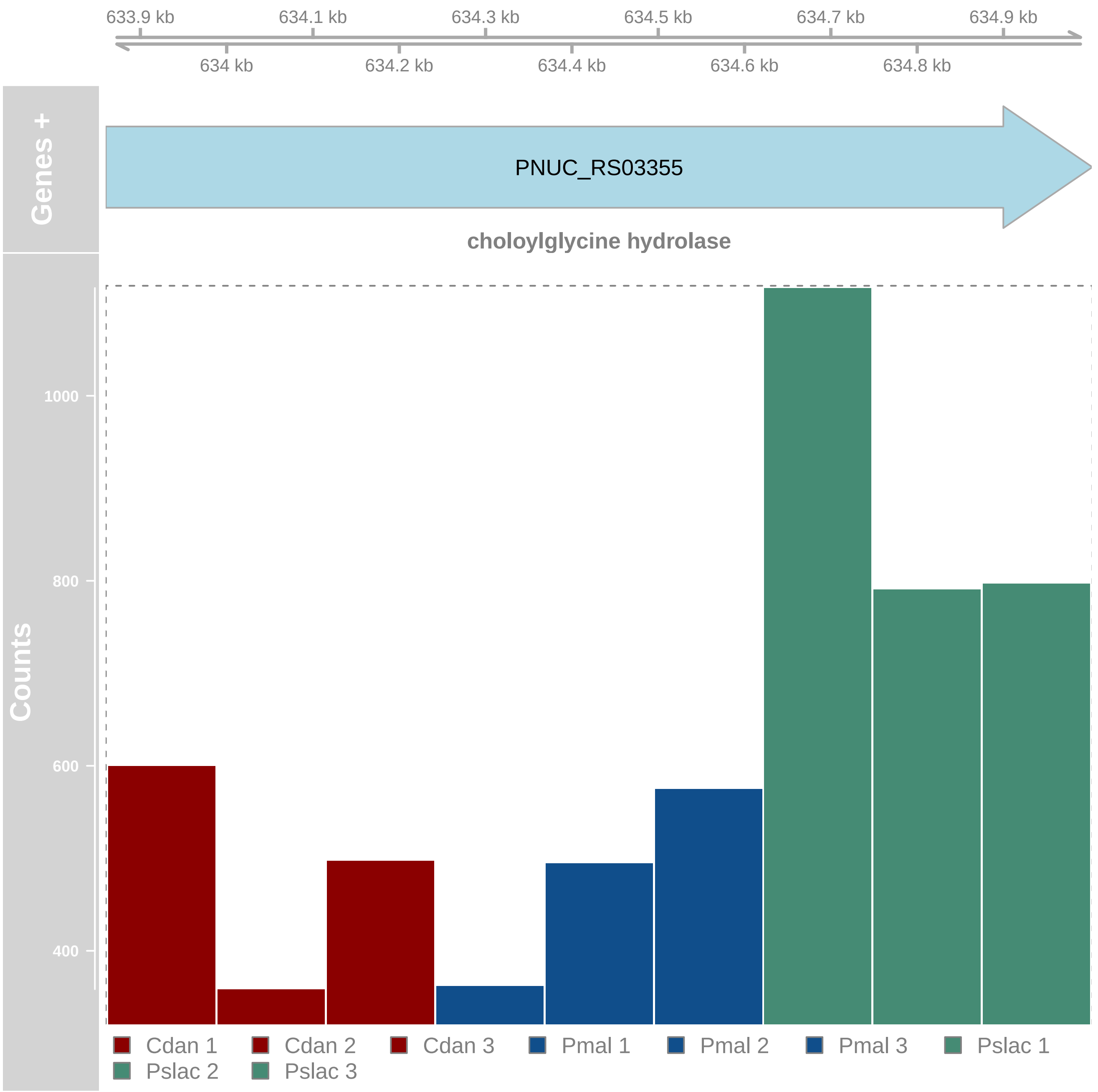
633.9 kb
634.1 kb
634.3 kb
634.5 kb
634.7 kb
634.9 kb
634 kb
634.2 kb
634.4 kb
634.6 kb
634.8 kb
Genes +
PNUC_RS03355
choloylglycine hydrolase
Counts
1000
800
600
400
Cdan 1
Cdan 2
Cdan 3
Pmal 1
Pmal 2
Pmal 3
Pslac 1
Pslac 2
Pslac 3

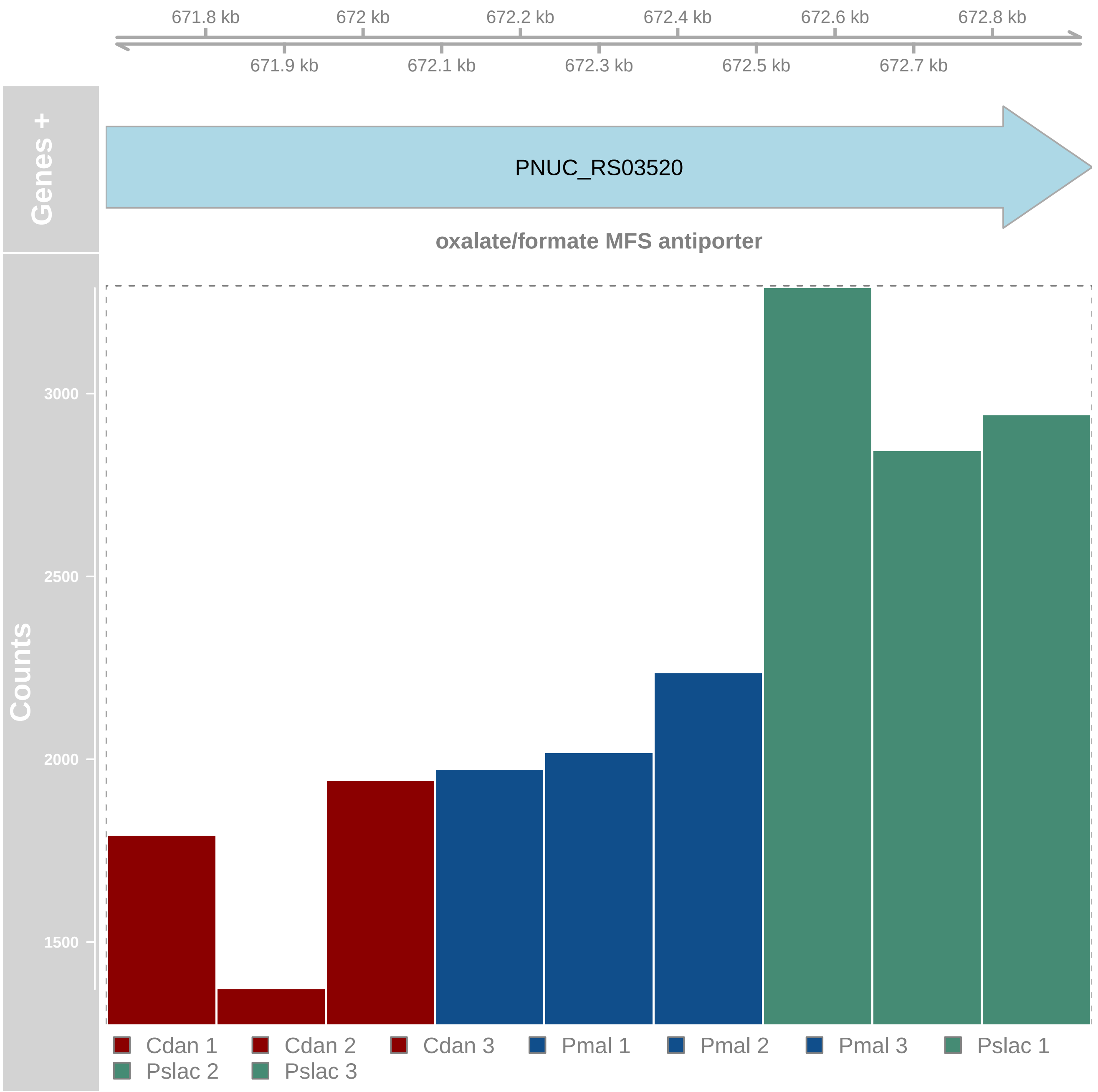
671.8 kb
672 kb
672.2 kb
672.4 kb
672.6 kb
672.8 kb
671.9 kb
672.1 kb
672.3 kb
672.5 kb
672.7 kb
Genes +
PNUC_RS03520
oxalate/formate MFS antiporter
Counts
3000
2500
2000
1500
Cdan 1
Cdan 2
Cdan 3
Pmal 1
Pmal 2
Pmal 3
Pslac 1
Pslac 2
Pslac 3

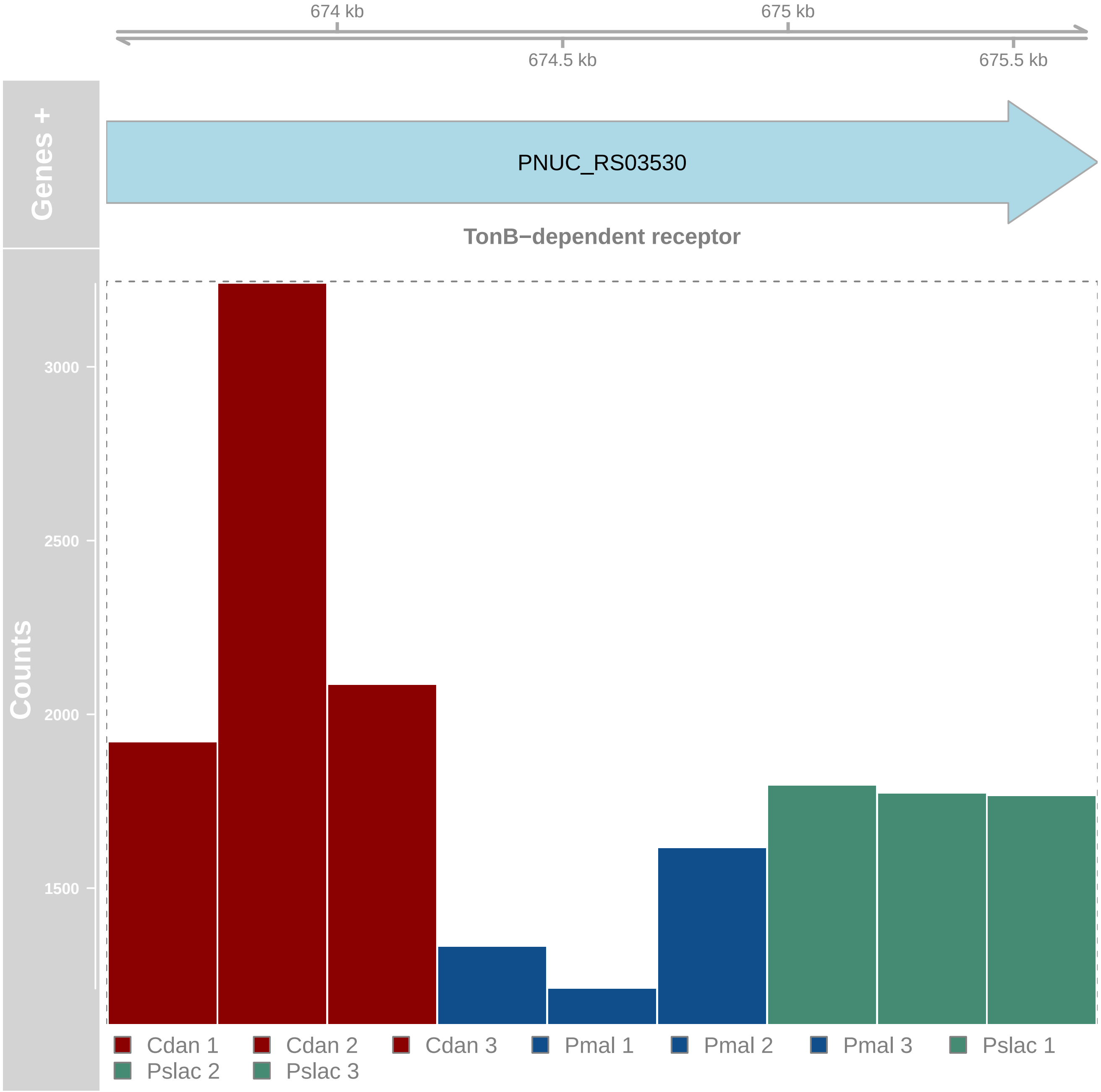
674 kb
675 kb
674.5 kb
675.5 kb
Genes +
PNUC_RS03530
TonB−dependent receptor
Counts
3000
2500
2000
1500
Cdan 1
Cdan 2
Cdan 3
Pmal 1
Pmal 2
Pmal 3
Pslac 1
Pslac 2
Pslac 3

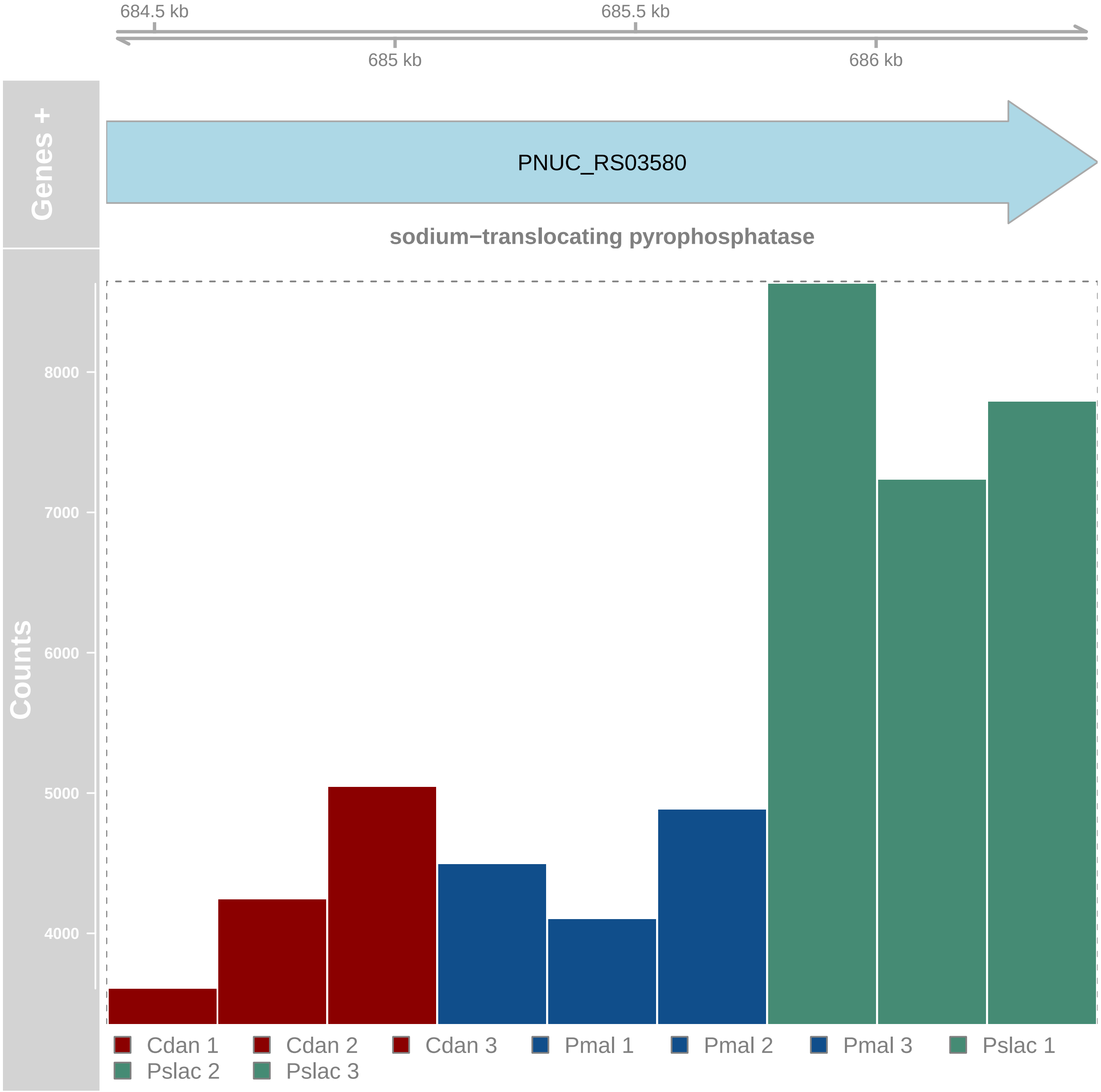
684.5 kb
685.5 kb
685 kb
686 kb
Genes +
PNUC_RS03580
sodium−translocating pyrophosphatase
Counts
8000
7000
6000
5000
4000
Cdan 1
Cdan 2
Cdan 3
Pmal 1
Pmal 2
Pmal 3
Pslac 1
Pslac 2
Pslac 3

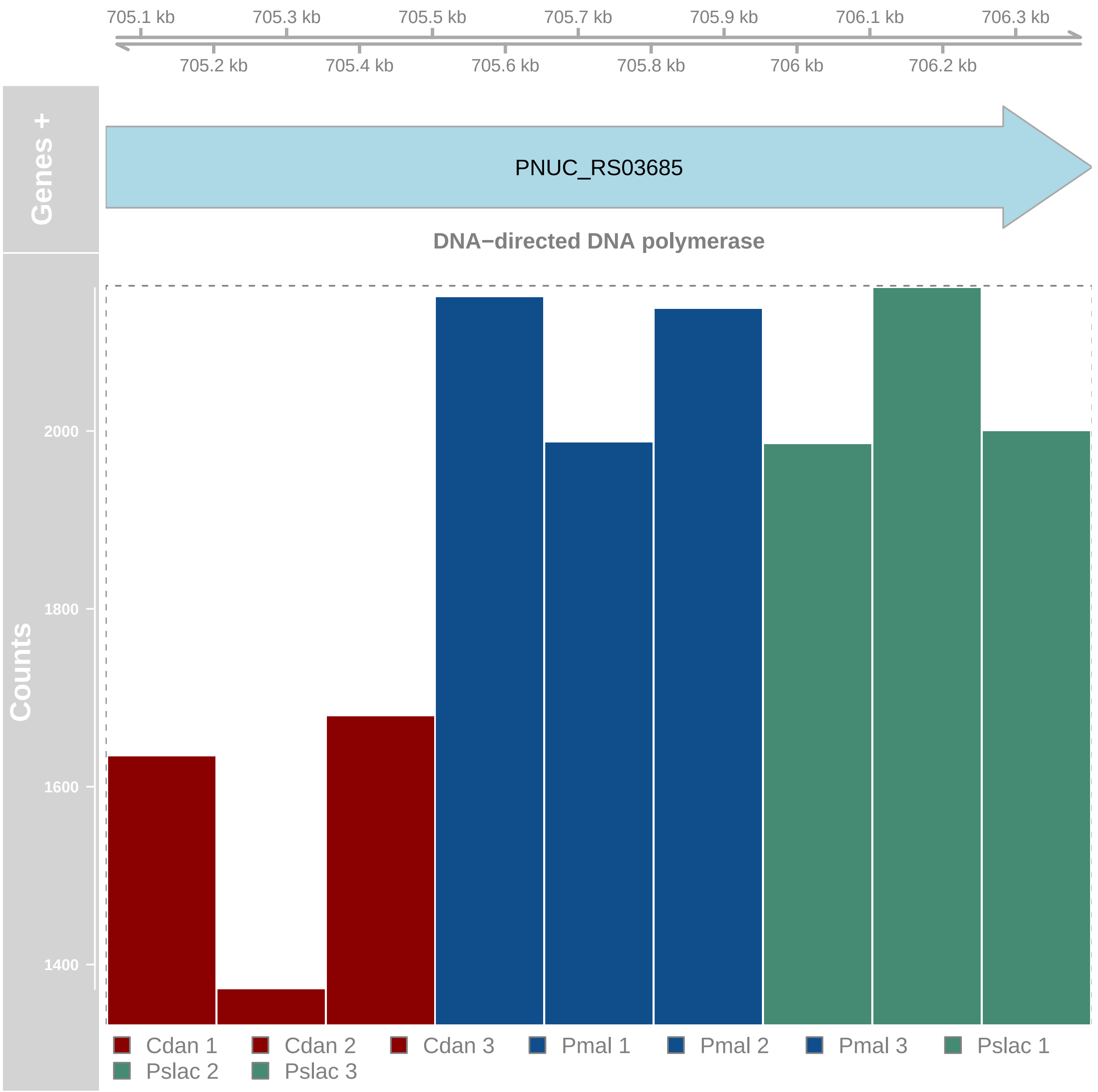
705.1 kb
705.2 kb
705.3 kb
705.4 kb
705.5 kb
705.6 kb
705.7 kb
705.8 kb
705.9 kb
706 kb
706.1 kb
706.2 kb
706.3 kb
Genes +
PNUC_RS03685
DNA−directed DNA polymerase
Counts
2000
1800
1600
1400
Cdan 1
Cdan 2
Cdan 3
Pmal 1
Pmal 2
Pmal 3
Pslac 1
Pslac 2
Pslac 3

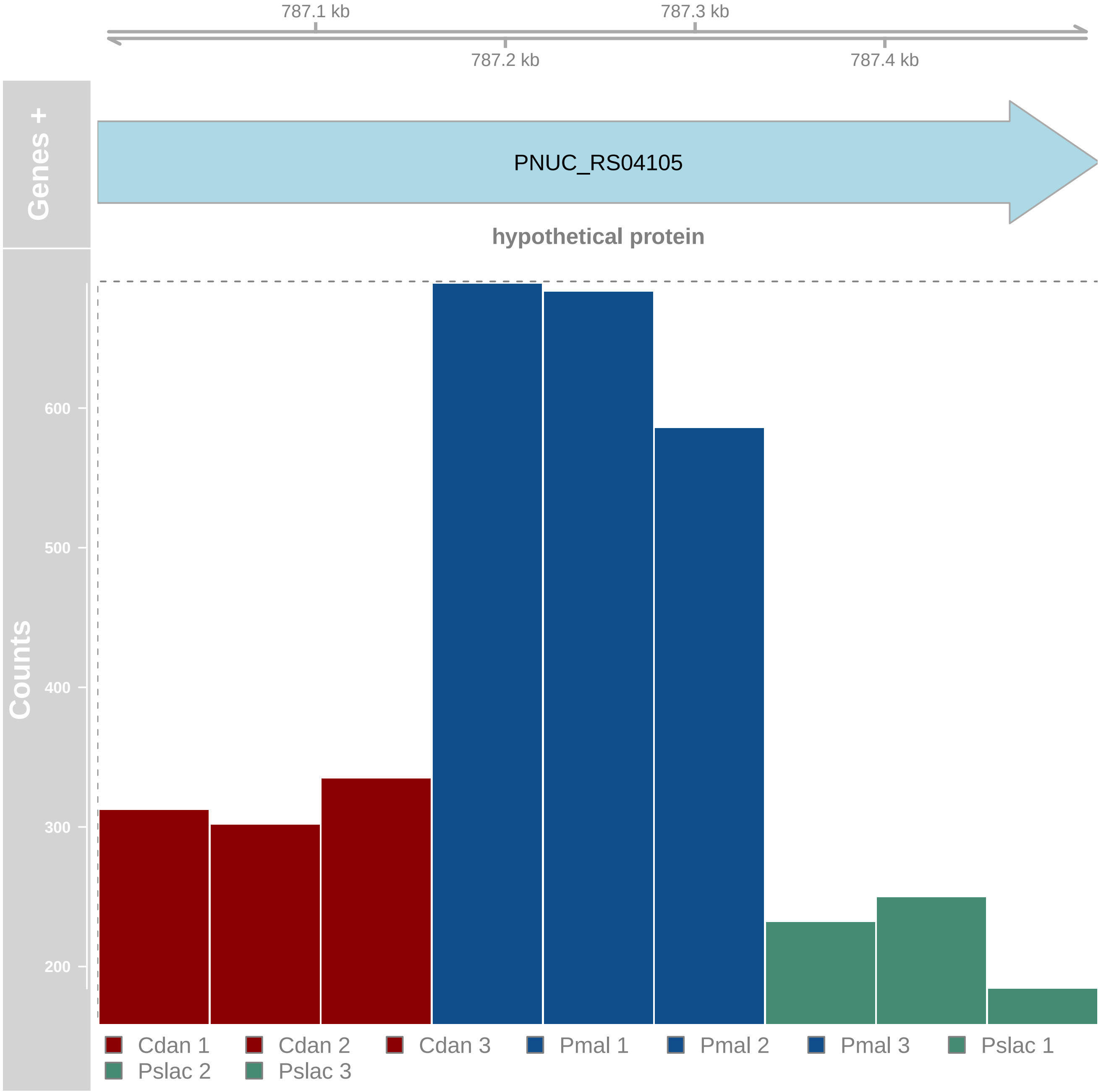

787.1 kb
787.3 kb
787.2 kb
787.4 kb
Genes +
PNUC_RS04105
hypothetical protein
Counts
600
500
400
300
200
Cdan 1
Cdan 2
Cdan 3
Pmal 1
Pmal 2
Pmal 3
Pslac 1
Pslac 2
Pslac 3

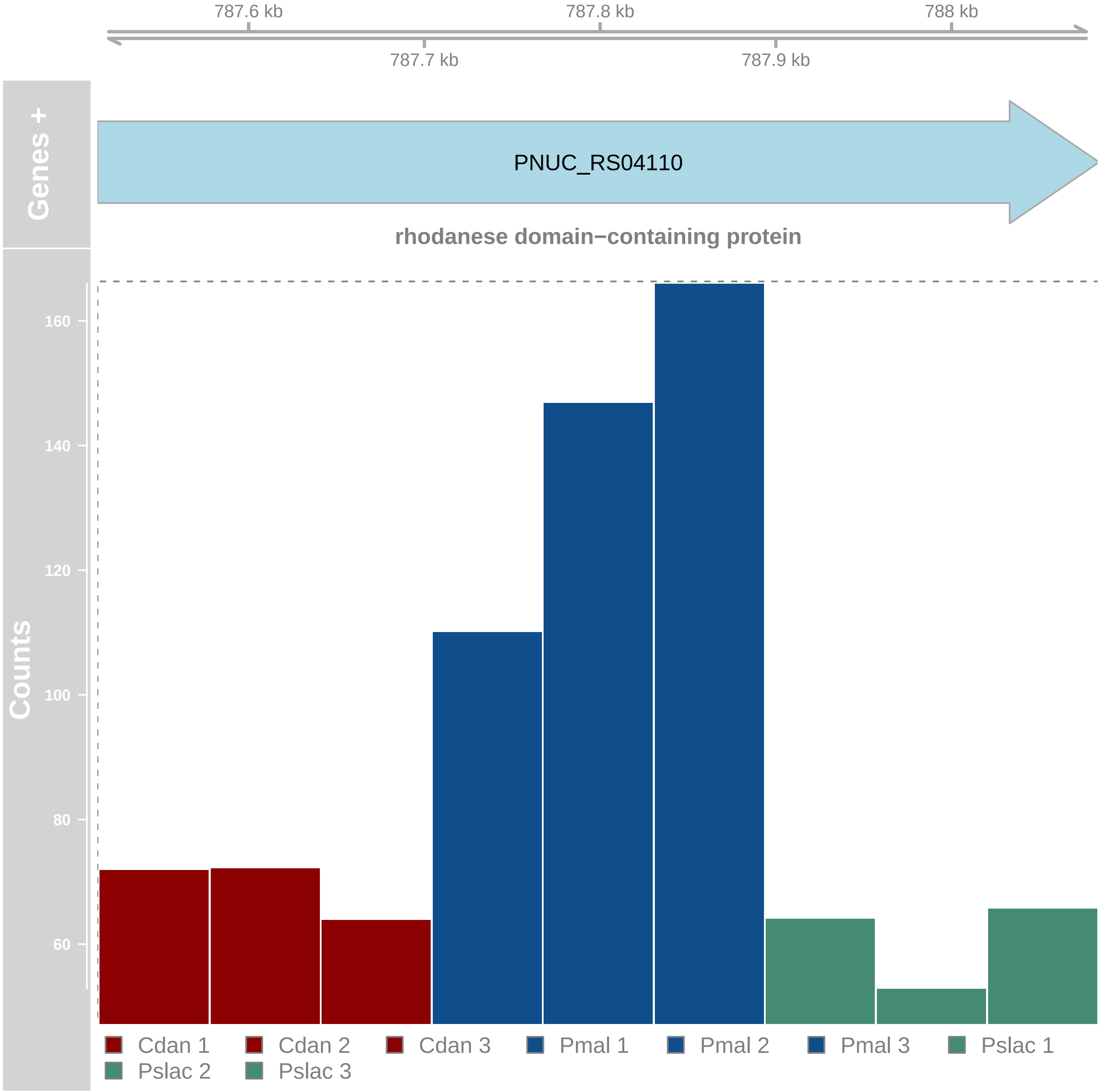

787.6 kb
787.7 kb
787.8 kb
787.9 kb
788 kb
Genes +
PNUC_RS04110
rhodanese domain−containing protein
Counts
160
140
120
100
80
60
Cdan 1
Cdan 2
Cdan 3
Pmal 1
Pmal 2
Pmal 3
Pslac 1
Pslac 2
Pslac 3

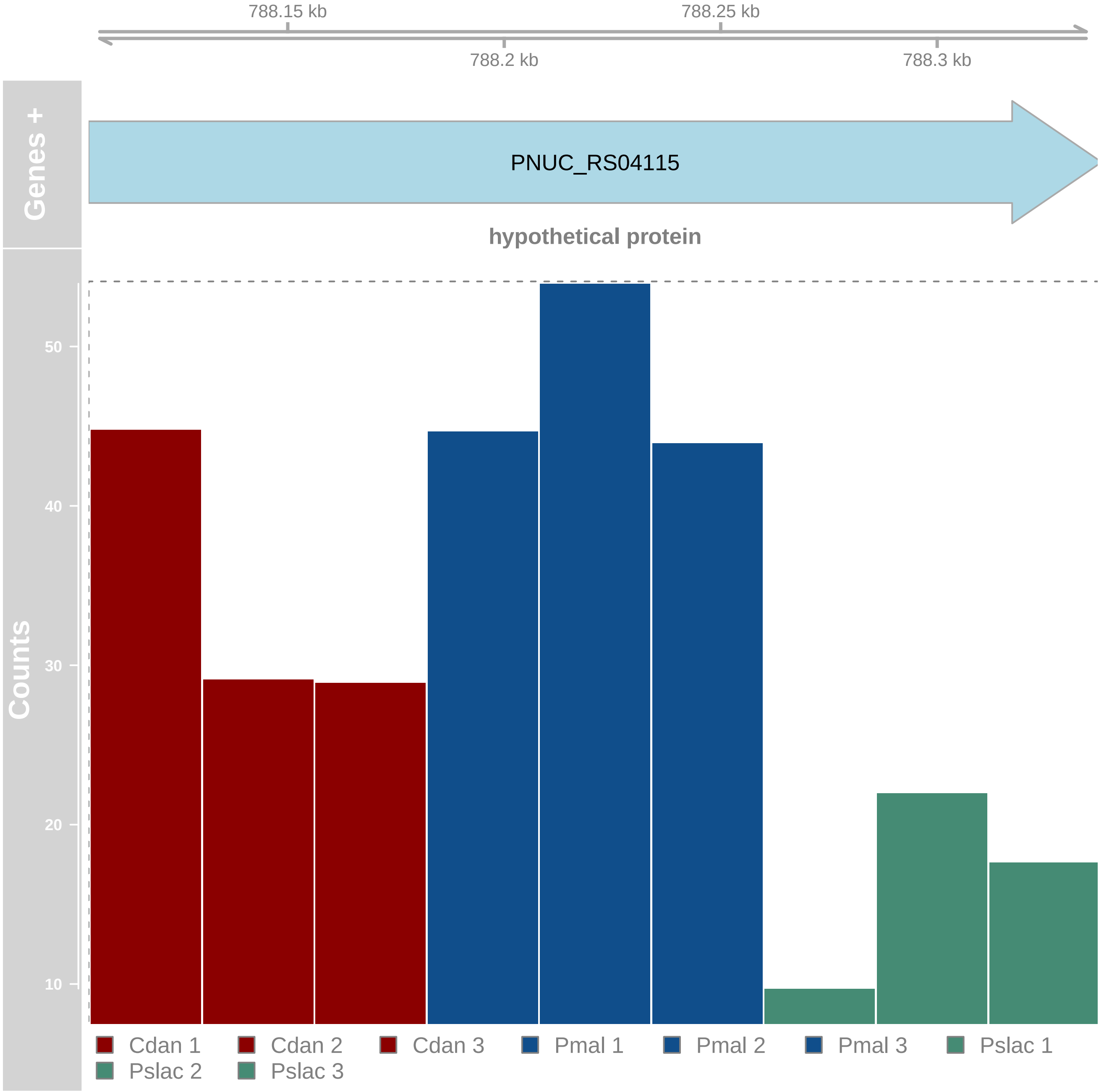
788.15 kb
788.25 kb
788.2 kb
788.3 kb
Genes +
PNUC_RS04115
hypothetical protein
Counts
50
40
30
20
10
Cdan 1
Cdan 2
Cdan 3
Pmal 1
Pmal 2
Pmal 3
Pslac 1
Pslac 2
Pslac 3

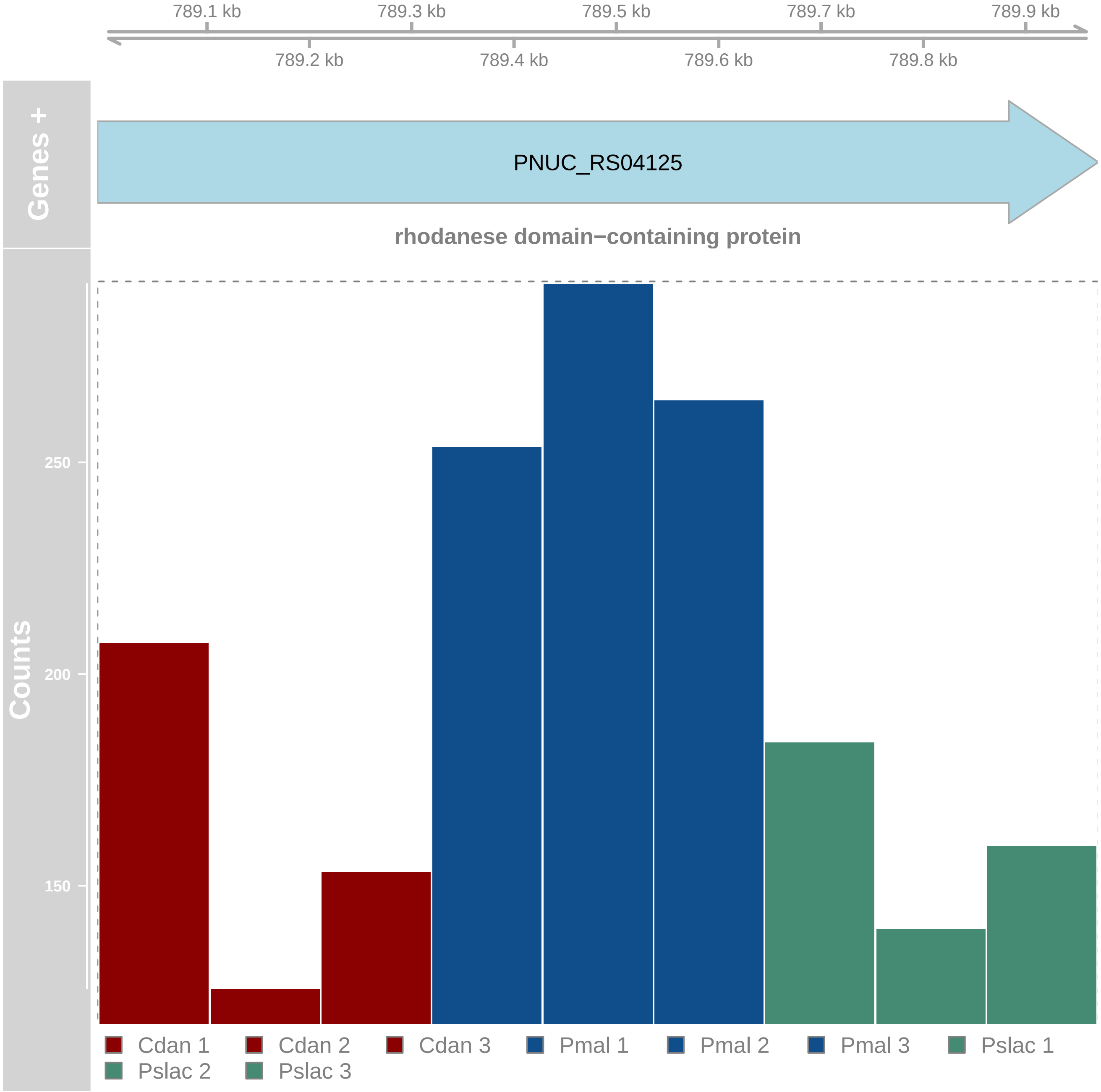
789.1 kb
789.2 kb
789.3 kb
789.4 kb
789.5 kb
789.6 kb
789.7 kb
789.8 kb
789.9 kb
Genes +
PNUC_RS04125
rhodanese domain−containing protein
Counts
250
200
150
Cdan 1
Cdan 2
Cdan 3
Pmal 1
Pmal 2
Pmal 3
Pslac 1
Pslac 2
Pslac 3

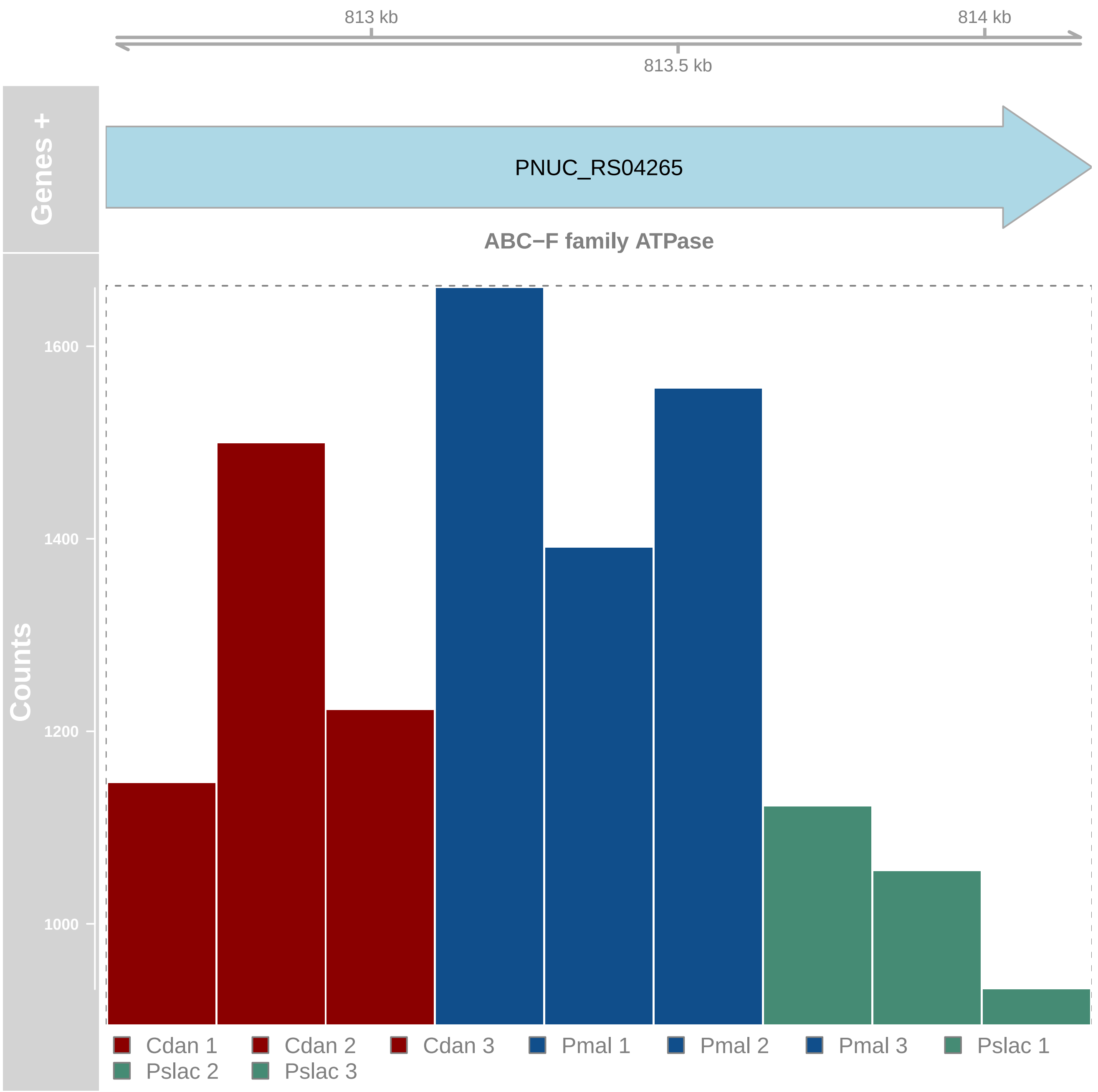
813 kb
814 kb
813.5 kb
Genes +
PNUC_RS04265
ABC−F family ATPase
Counts
1600
1400
1200
1000
Cdan 1
Cdan 2
Cdan 3
Pmal 1
Pmal 2
Pmal 3
Pslac 1
Pslac 2
Pslac 3

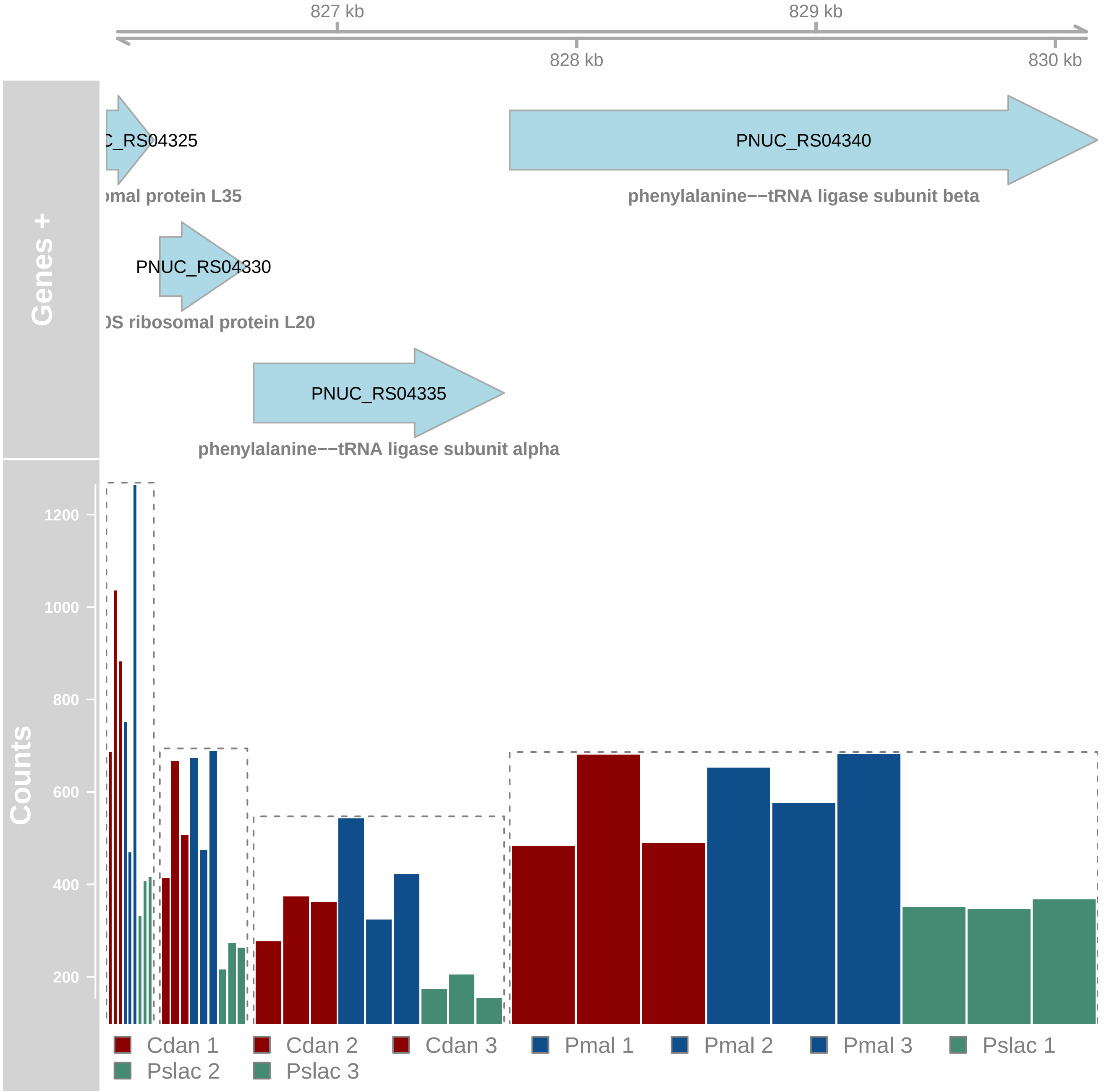
827 kb
829 kb
828 kb
830 kb
Genes +
C_RS04325
omal protein L35
PNUC_RS04340
phenylalanine−−tRNA ligase subunit beta
PNUC_RS04330
0S ribosomal protein L20
PNUC_RS04335
phenylalanine−−tRNA ligase subunit alpha
Counts
1200
1000
800
600
400
200
Cdan 1
Cdan 2
Cdan 3
Pmal 1
Pmal 2
Pmal 3
Pslac 1
Pslac 2
Pslac 3

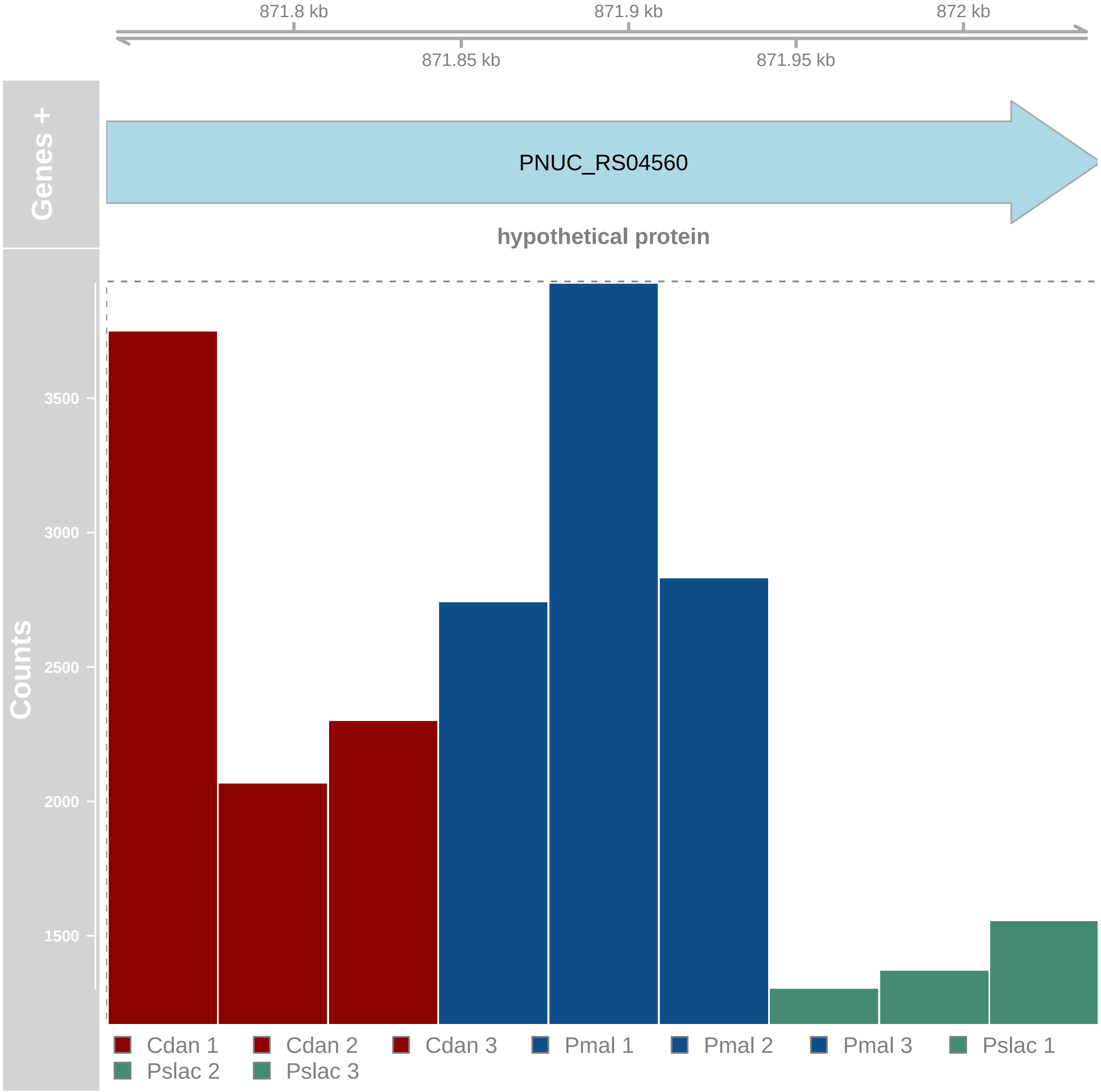
871.8 kb
871.85 kb
871.9 kb
871.95 kb
872 kb
Genes +
PNUC_RS04560
hypothetical protein
Counts
3500
3000
2500
2000
1500
Cdan 1
Cdan 2
Cdan 3
Pmal 1
Pmal 2
Pmal 3
Pslac 1
Pslac 2
Pslac 3

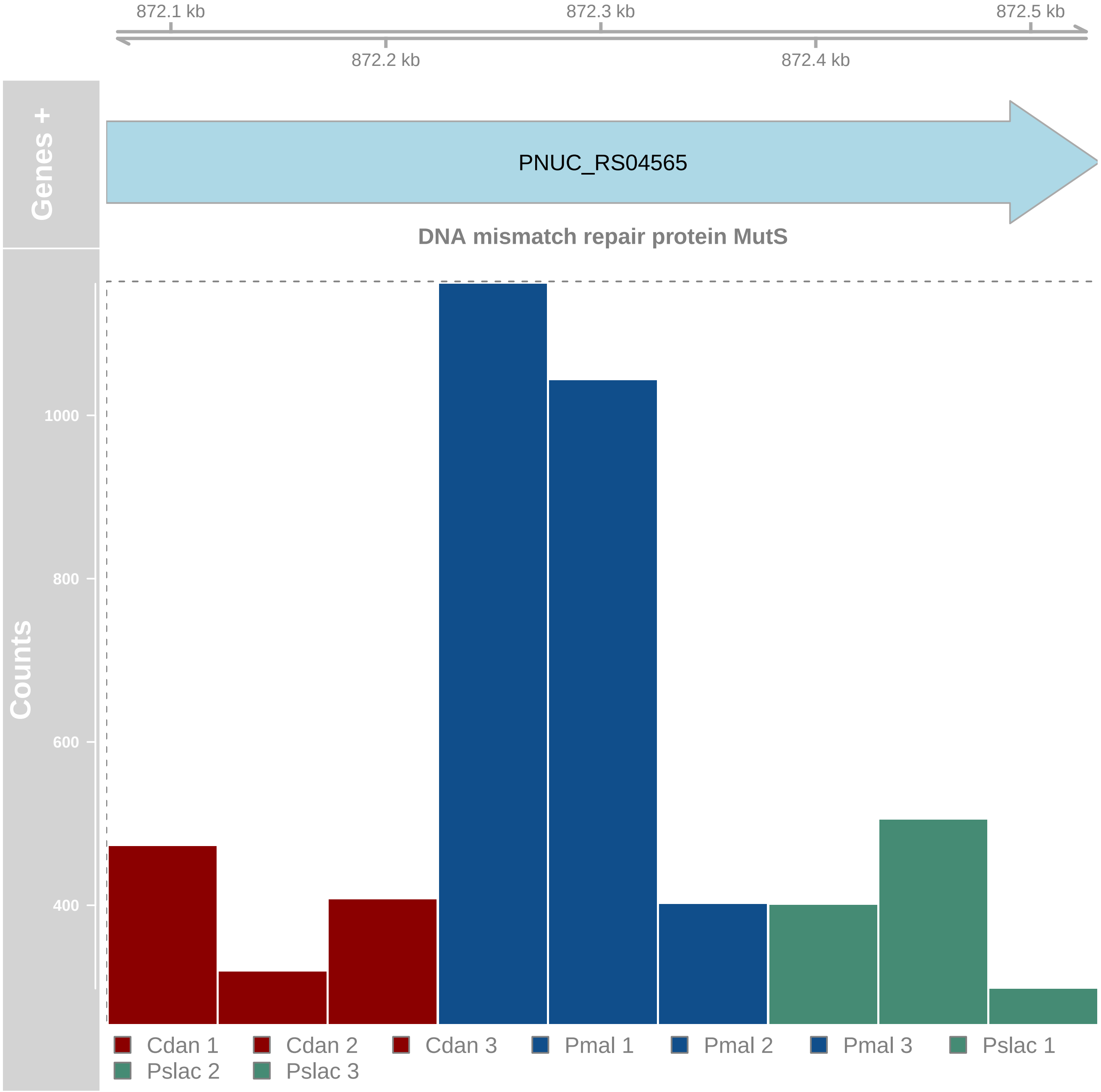
872.1 kb
872.2 kb
872.3 kb
872.4 kb
872.5 kb
Genes +
PNUC_RS04565
DNA mismatch repair protein MutS
Counts
1000
800
600
400
Cdan 1
Cdan 2
Cdan 3
Pmal 1
Pmal 2
Pmal 3
Pslac 1
Pslac 2
Pslac 3

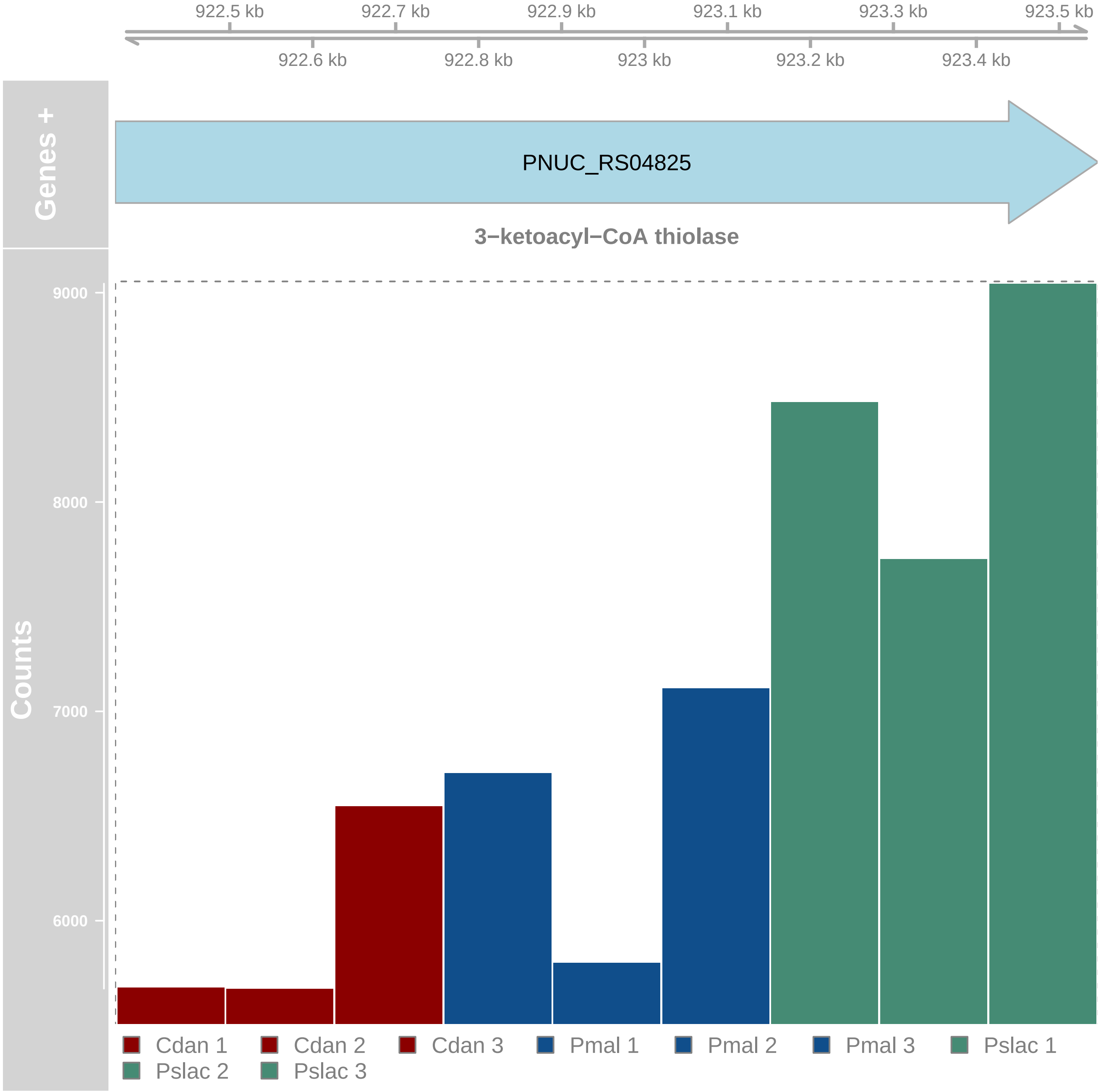
922.5 kb
922.7 kb
922.9 kb
923.1 kb
923.3 kb
923.5 kb
922.6 kb
922.8 kb
923 kb
923.2 kb
923.4 kb
Genes +
PNUC_RS04825
3−ketoacyl−CoA thiolase
Counts
9000
8000
7000
6000
Cdan 1
Cdan 2
Cdan 3
Pmal 1
Pmal 2
Pmal 3
Pslac 1
Pslac 2
Pslac 3

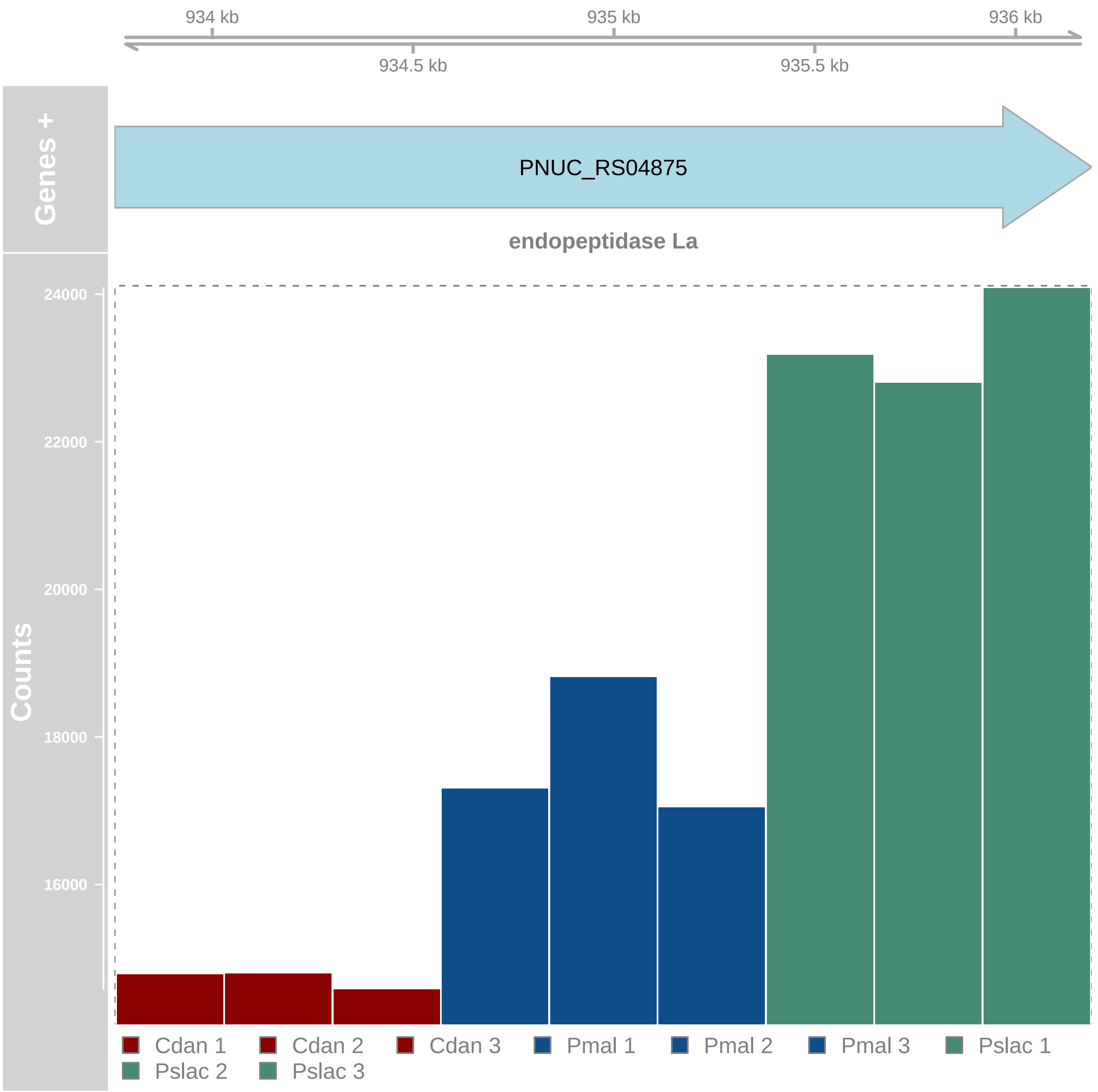
934 kb
935 kb
936 kb
934.5 kb
935.5 kb
Genes +
PNUC_RS04875
endopeptidase La
Counts
24000
22000
20000
18000
16000
Cdan 1
Cdan 2
Cdan 3
Pmal 1
Pmal 2
Pmal 3
Pslac 1
Pslac 2
Pslac 3

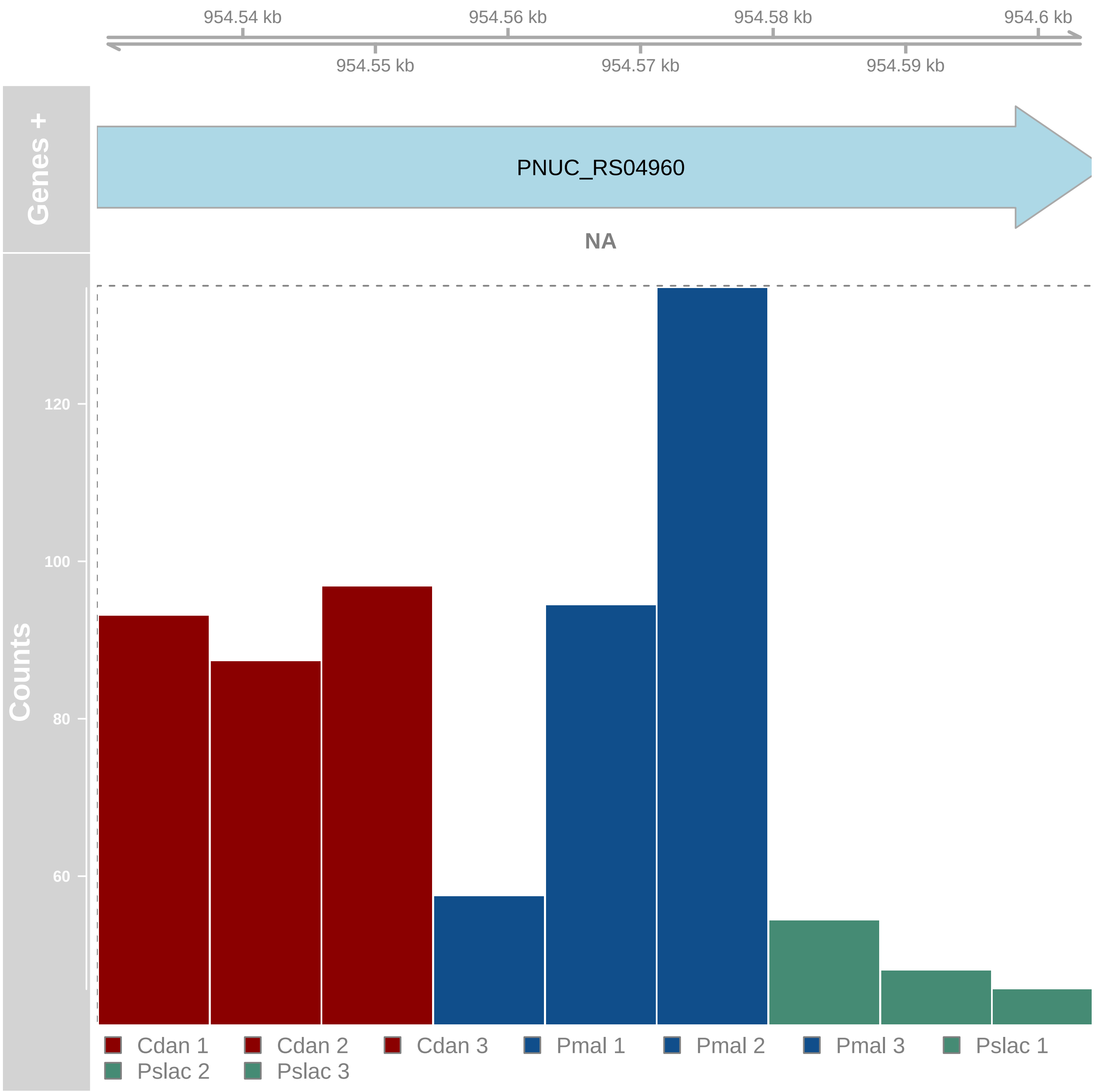
954.54 kb
954.56 kb
954.58 kb
954.6 kb
954.55 kb
954.57 kb
954.59 kb
Genes +
PNUC_RS04960
NA
Counts
120
100
80
60
Cdan 1
Cdan 2
Cdan 3
Pmal 1
Pmal 2
Pmal 3
Pslac 1
Pslac 2
Pslac 3

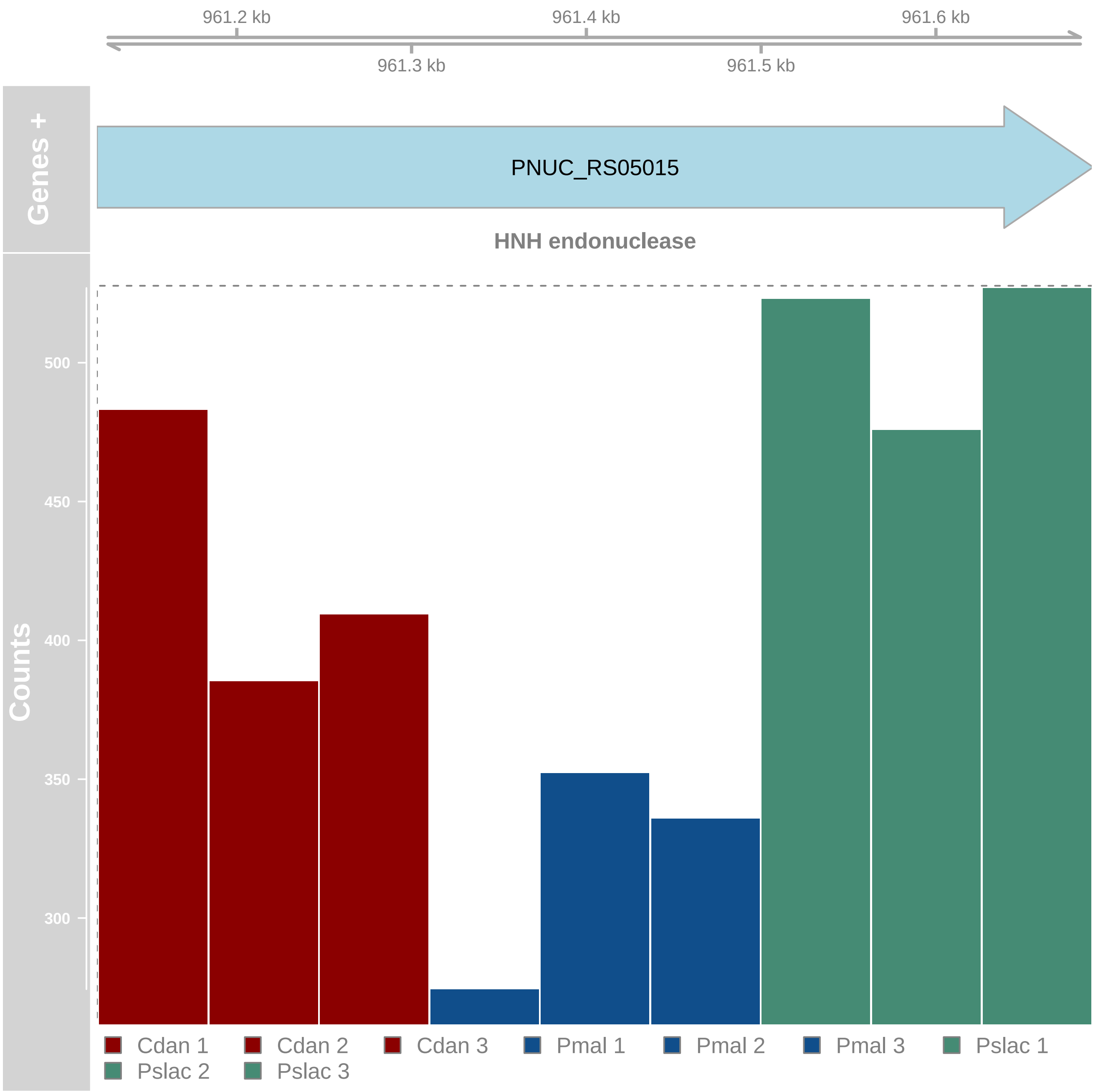

961.2 kb
961.4 kb
961.6 kb
961.3 kb
961.5 kb
Genes +
PNUC_RS05015
HNH endonuclease
Counts
500
450
400
350
300
Cdan 1
Cdan 2
Cdan 3
Pmal 1
Pmal 2
Pmal 3
Pslac 1
Pslac 2
Pslac 3

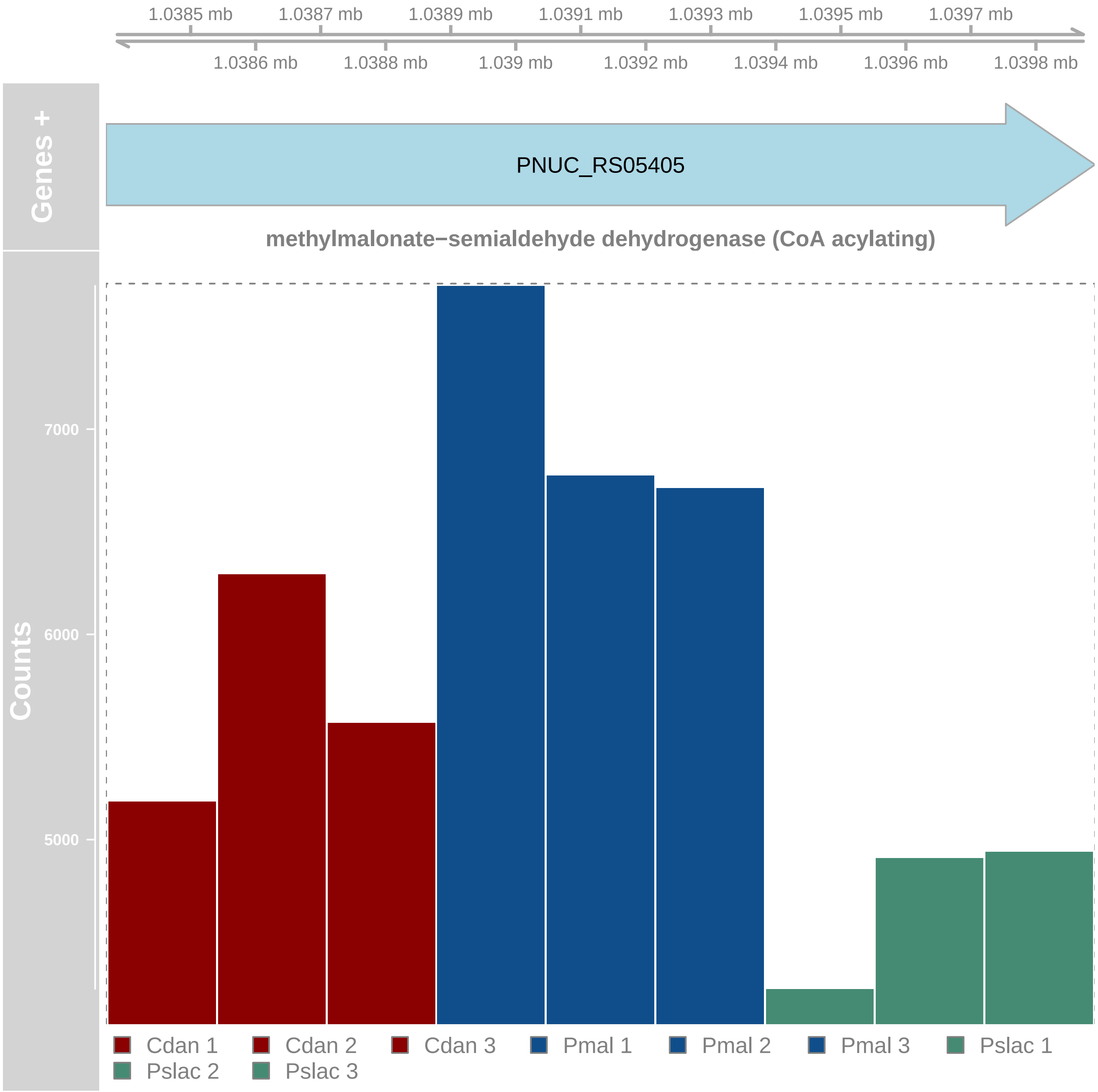
1.0385 mb
1.0387 mb
1.0389 mb
1.0391 mb
1.0393 mb
1.0395 mb
1.0397 mb
1.0386 mb
1.0388 mb
1.039 mb
1.0392 mb
1.0394 mb
1.0396 mb
1.0398 mb
Genes +
PNUC_RS05405
methylmalonate−semialdehyde dehydrogenase (CoA acylating)
Counts
7000
6000
5000
Cdan 1
Cdan 2
Cdan 3
Pmal 1
Pmal 2
Pmal 3
Pslac 1
Pslac 2
Pslac 3

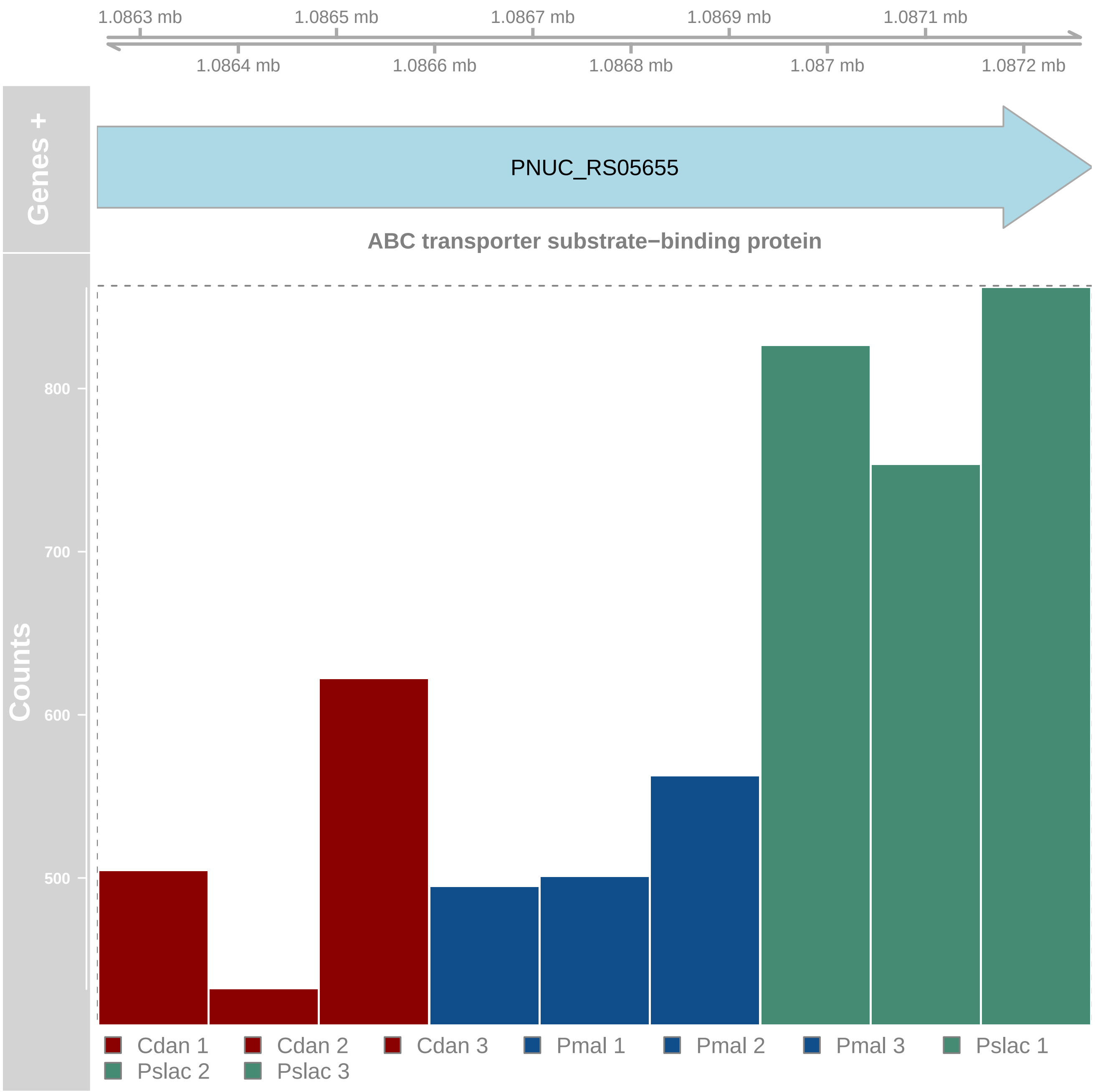

1.0863 mb
1.0864 mb
1.0865 mb
1.0866 mb
1.0867 mb
1.0868 mb
1.0869 mb
1.087 mb
1.0871 mb
1.0872 mb
Genes +
PNUC_RS05655
ABC transporter substrate−binding protein
Counts
800
700
600
500
Cdan 1
Cdan 2
Cdan 3
Pmal 1
Pmal 2
Pmal 3
Pslac 1
Pslac 2
Pslac 3

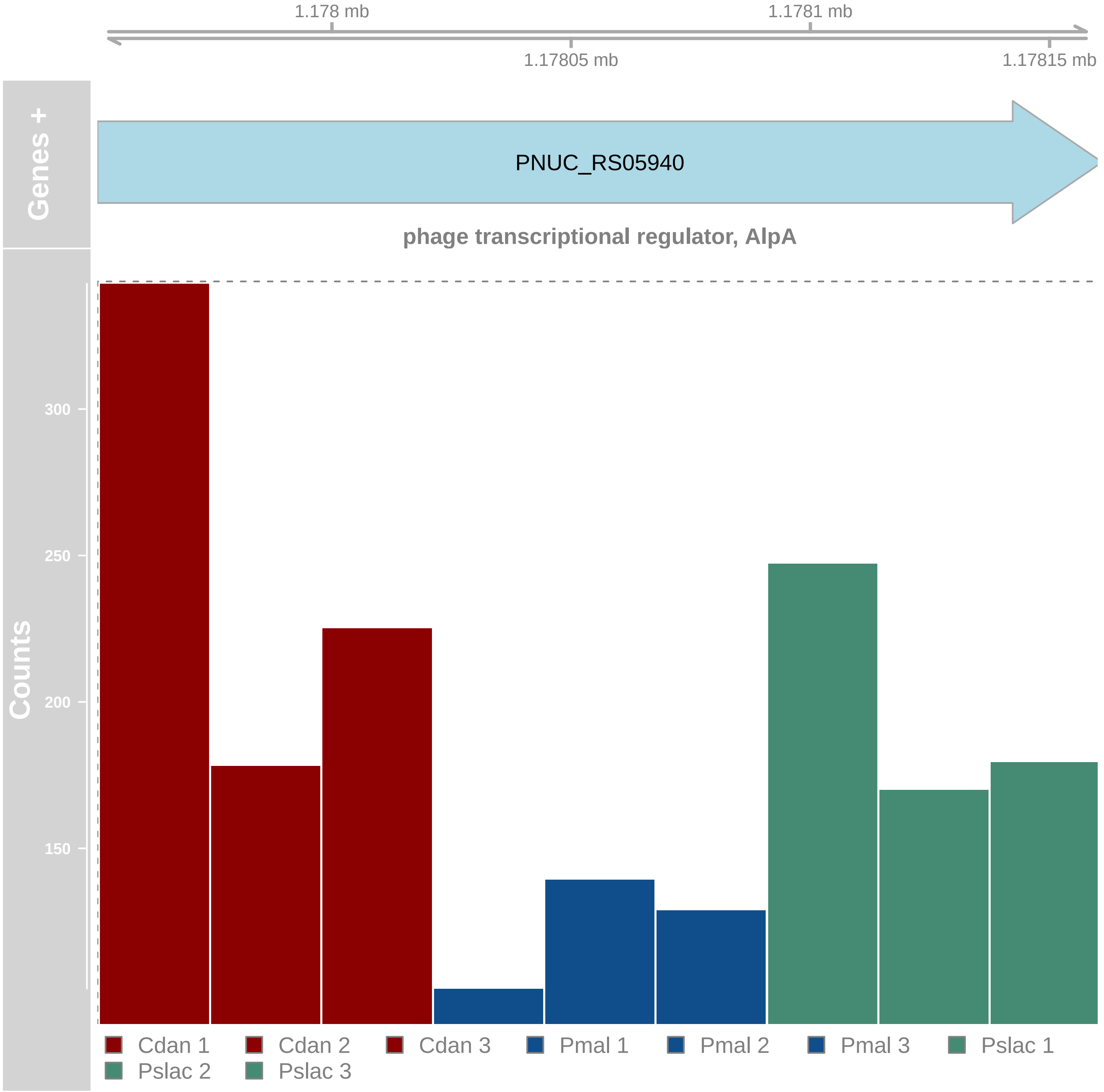
1.178 mb
1.1781 mb
1.17805 mb
1.17815 mb
Genes +
PNUC_RS05940
phage transcriptional regulator, AlpA
Counts
300
250
200
150
Cdan 1
Cdan 2
Cdan 3
Pmal 1
Pmal 2
Pmal 3
Pslac 1
Pslac 2
Pslac 3

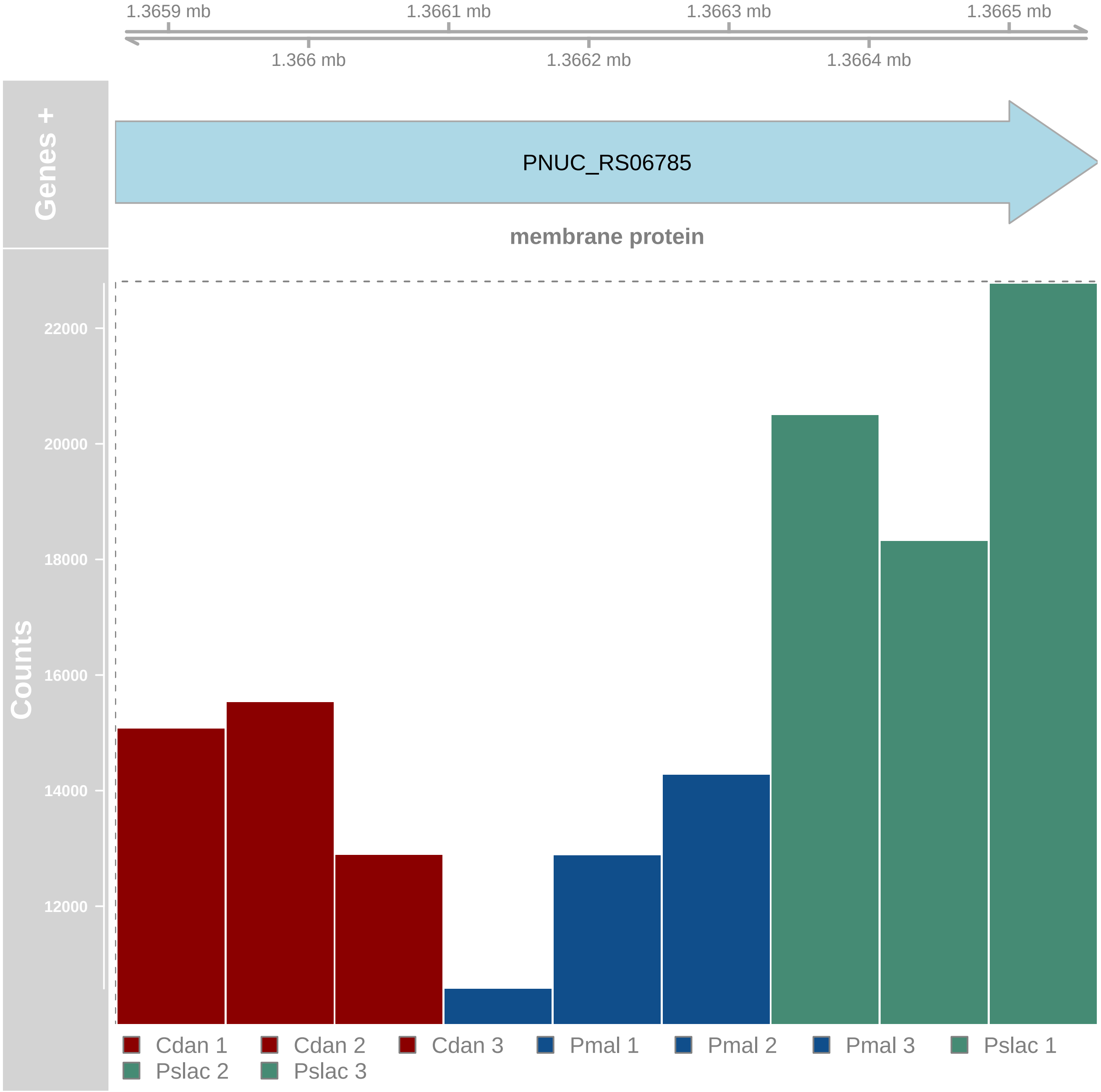
1.3659 mb
1.3661 mb
1.3663 mb
1.3665 mb
1.366 mb
1.3662 mb
1.3664 mb
Genes +
PNUC_RS06785
membrane protein
Counts
22000
20000
18000
16000
14000
12000
Cdan 1
Cdan 2
Cdan 3
Pmal 1
Pmal 2
Pmal 3
Pslac 1
Pslac 2
Pslac 3

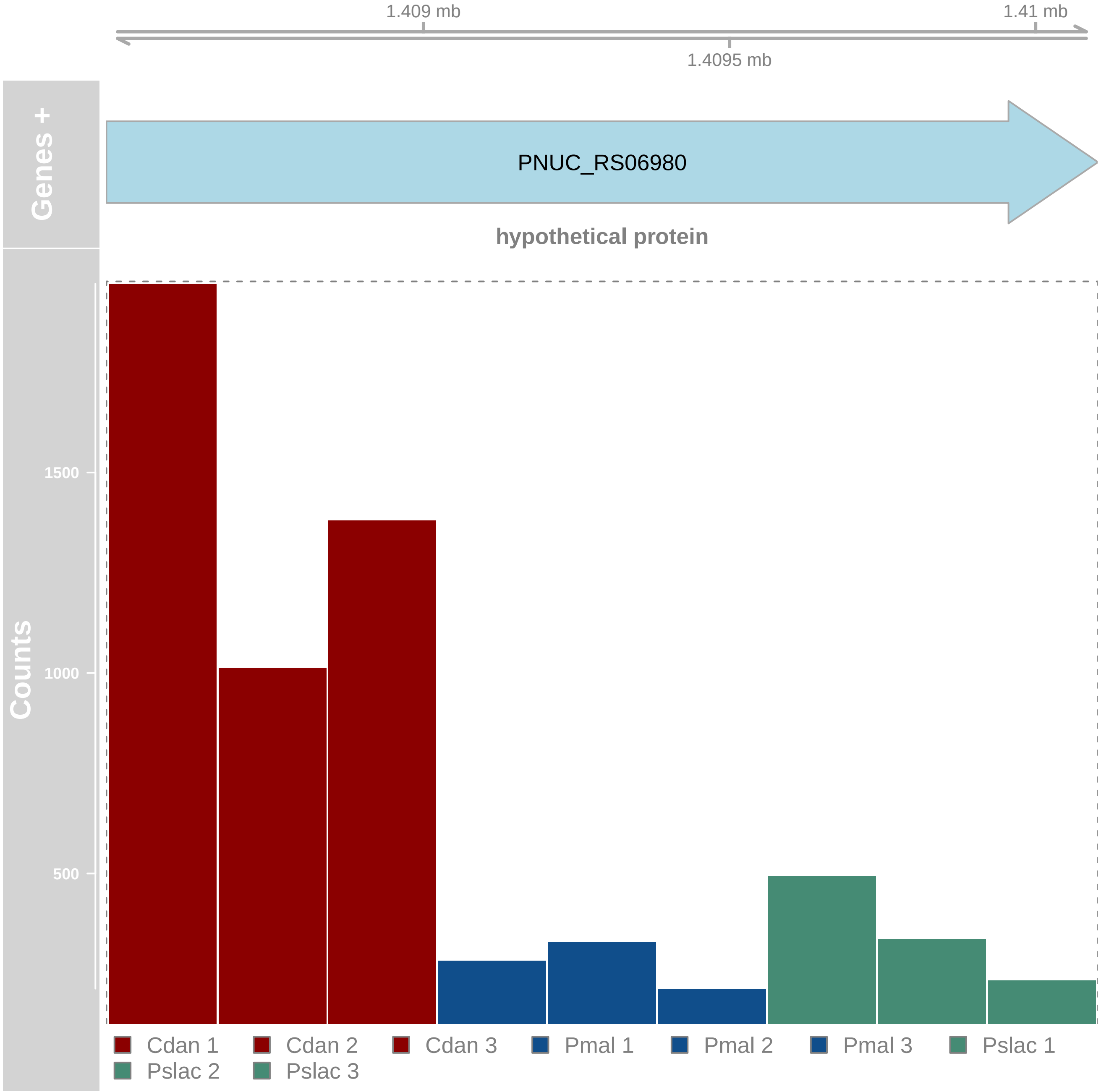

1.409 mb
1.41 mb
1.4095 mb
Genes +
PNUC_RS06980
hypothetical protein
Counts
1500
1000
500
Cdan 1
Cdan 2
Cdan 3
Pmal 1
Pmal 2
Pmal 3
Pslac 1
Pslac 2
Pslac 3

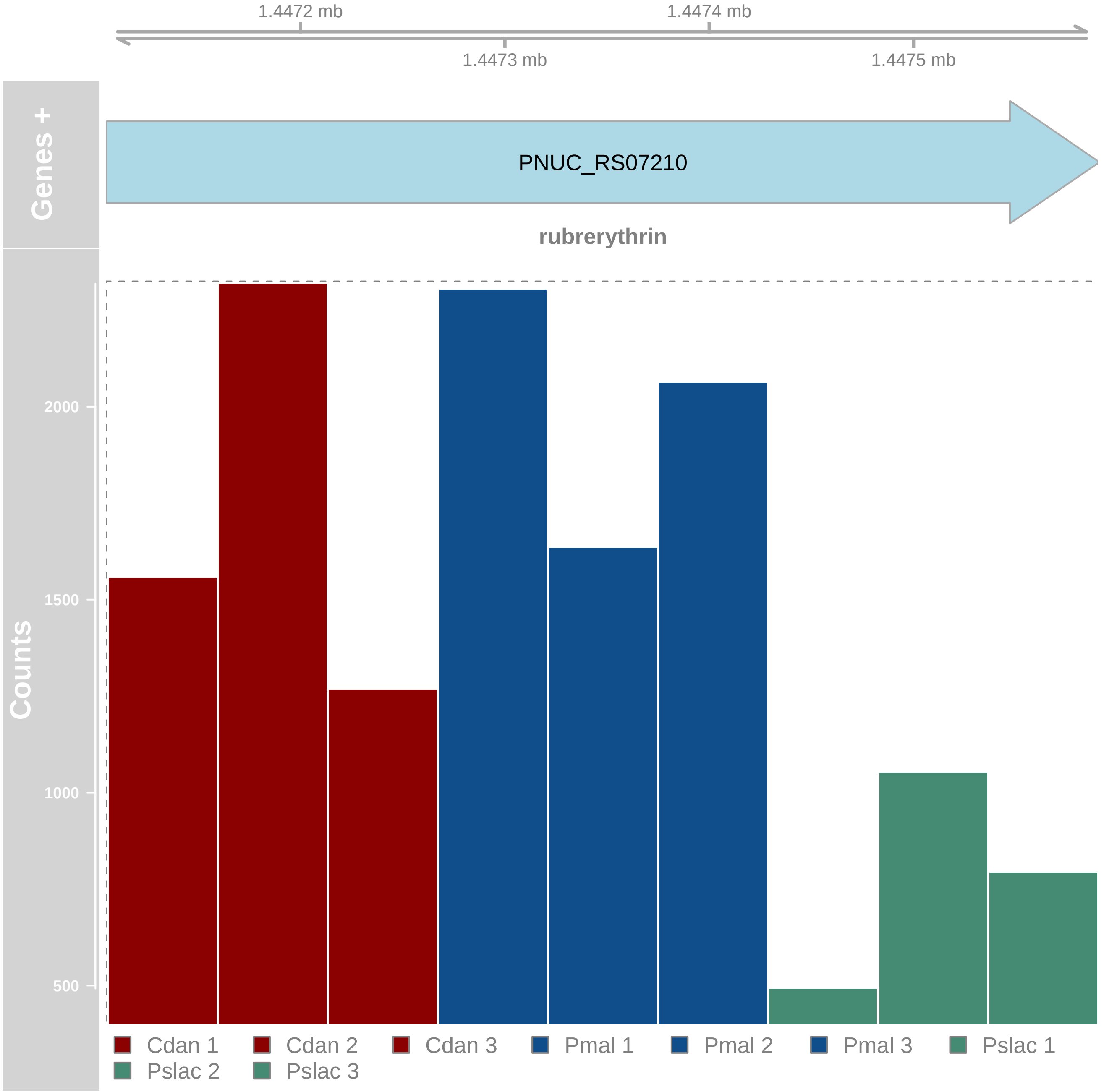
1.4472 mb
1.4474 mb
1.4473 mb
1.4475 mb
Genes +
PNUC_RS07210
rubrerythrin
Counts
2000
1500
1000
500
Cdan 1
Cdan 2
Cdan 3
Pmal 1
Pmal 2
Pmal 3
Pslac 1
Pslac 2
Pslac 3

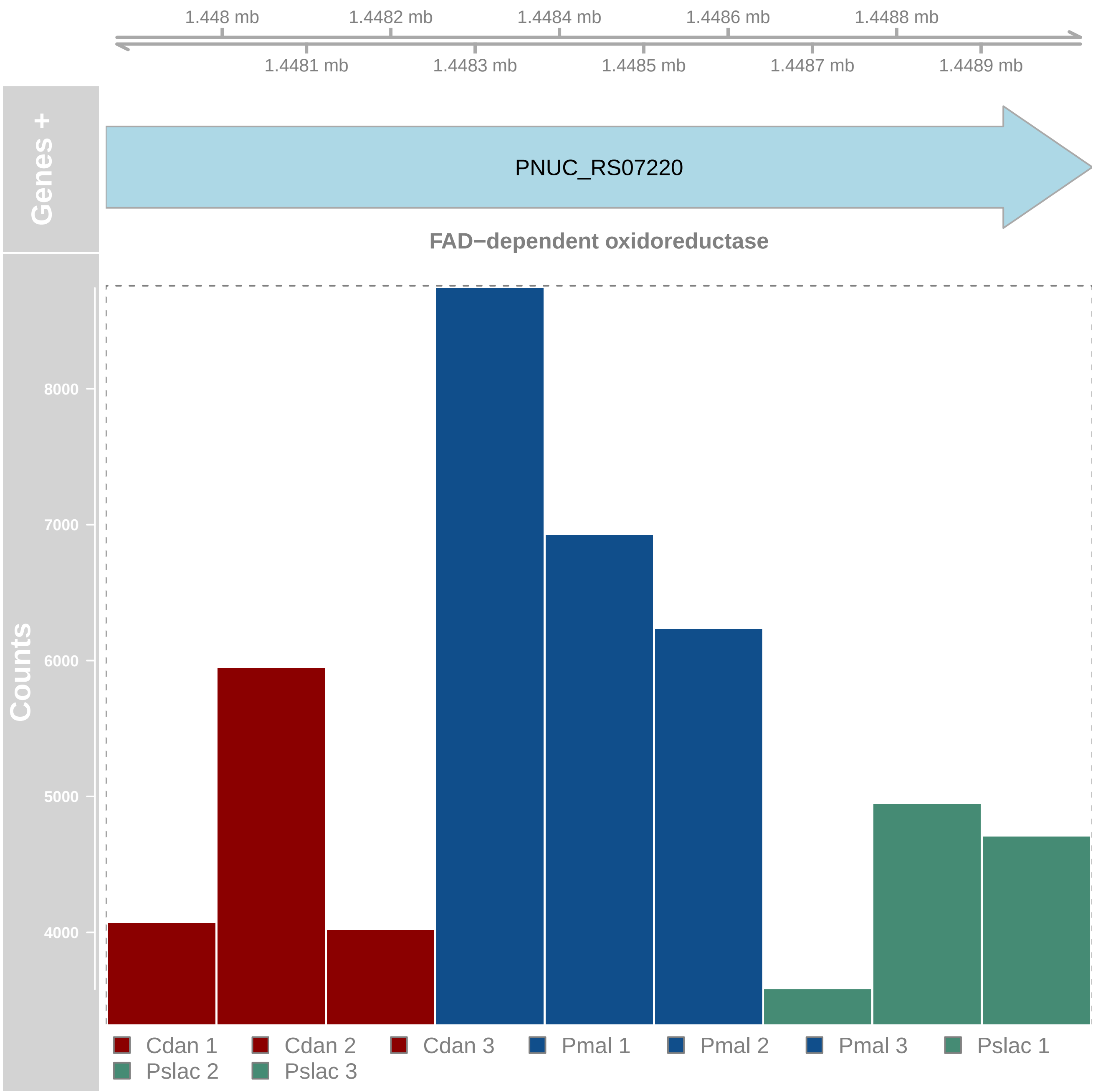
1.448 mb
1.4481 mb
1.4482 mb
1.4483 mb
1.4484 mb
1.4485 mb
1.4486 mb
1.4487 mb
1.4488 mb
1.4489 mb
Genes +
PNUC_RS07220
FAD−dependent oxidoreductase
Counts
8000
7000
6000
5000
4000
Cdan 1
Cdan 2
Cdan 3
Pmal 1
Pmal 2
Pmal 3
Pslac 1
Pslac 2
Pslac 3

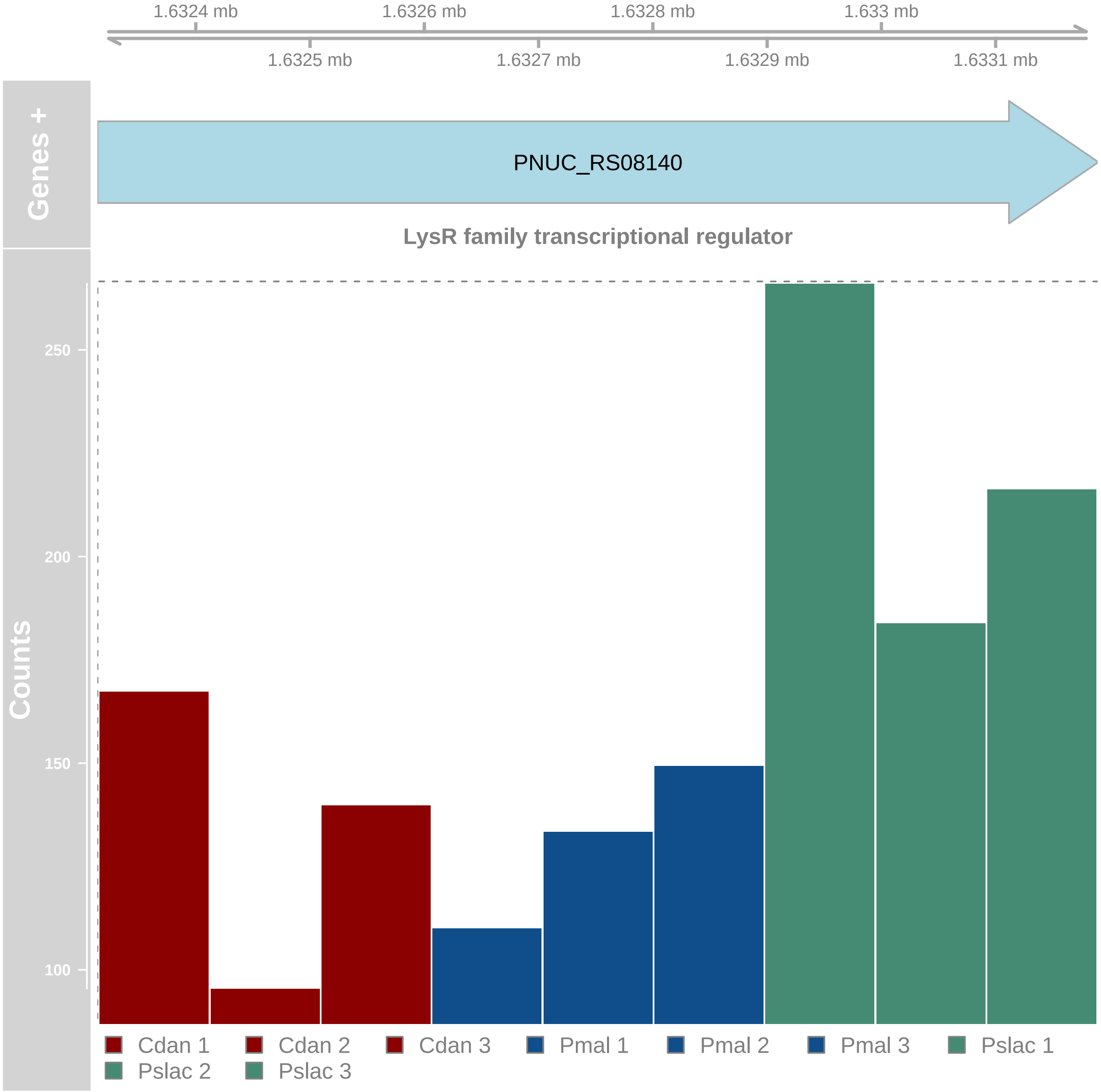
1.6324 mb
1.6325 mb
1.6326 mb
1.6327 mb
1.6328 mb
1.6329 mb
1.633 mb
1.6331 mb
Genes +
PNUC_RS08140
LysR family transcriptional regulator
Counts
250
200
150
100
Cdan 1
Cdan 2
Cdan 3
Pmal 1
Pmal 2
Pmal 3
Pslac 1
Pslac 2
Pslac 3

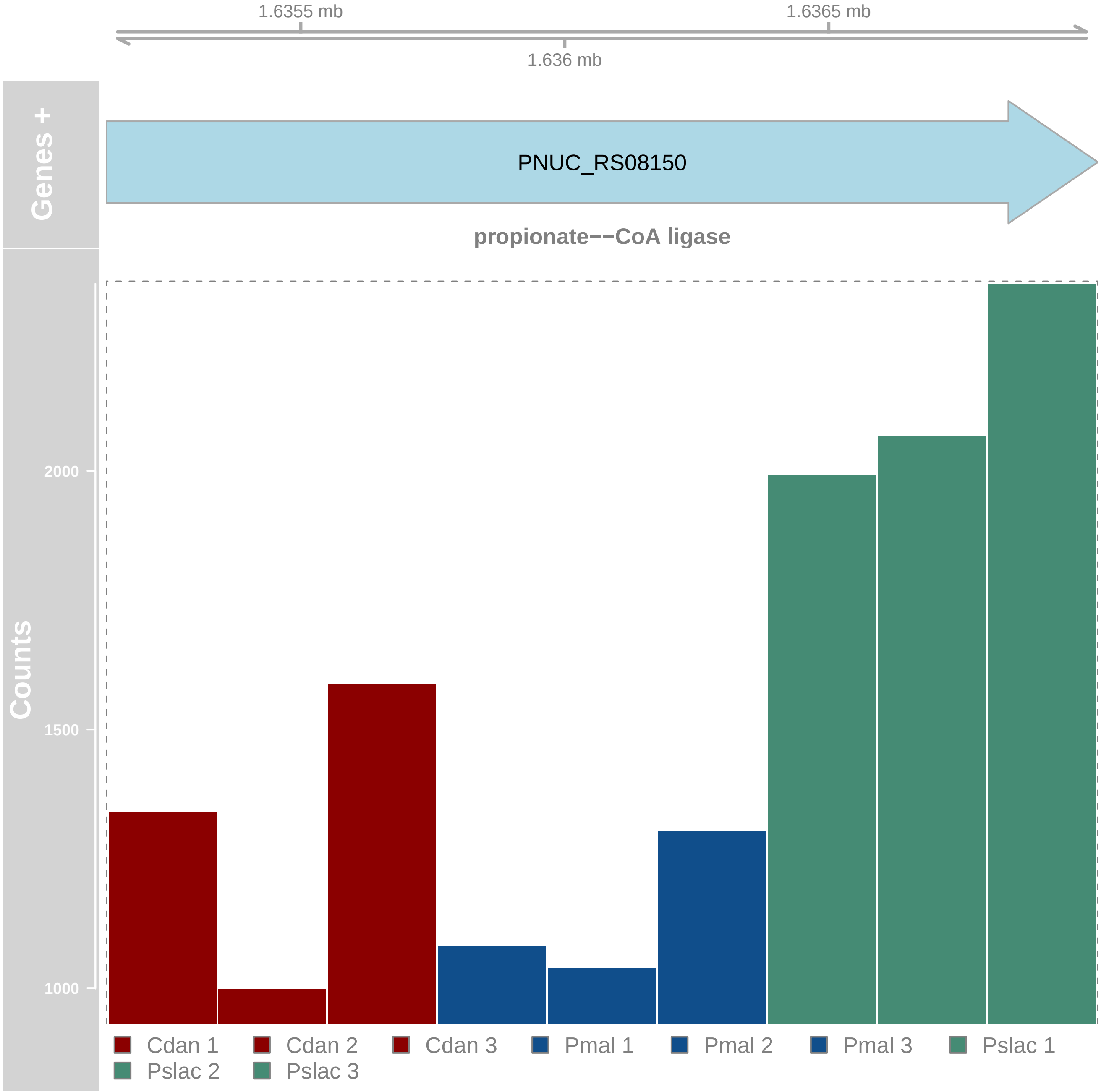
1.6355 mb
1.6365 mb
1.636 mb
Genes +
PNUC_RS08150
propionate−−CoA ligase
Counts
2000
1500
1000
Cdan 1
Cdan 2
Cdan 3
Pmal 1
Pmal 2
Pmal 3
Pslac 1
Pslac 2
Pslac 3

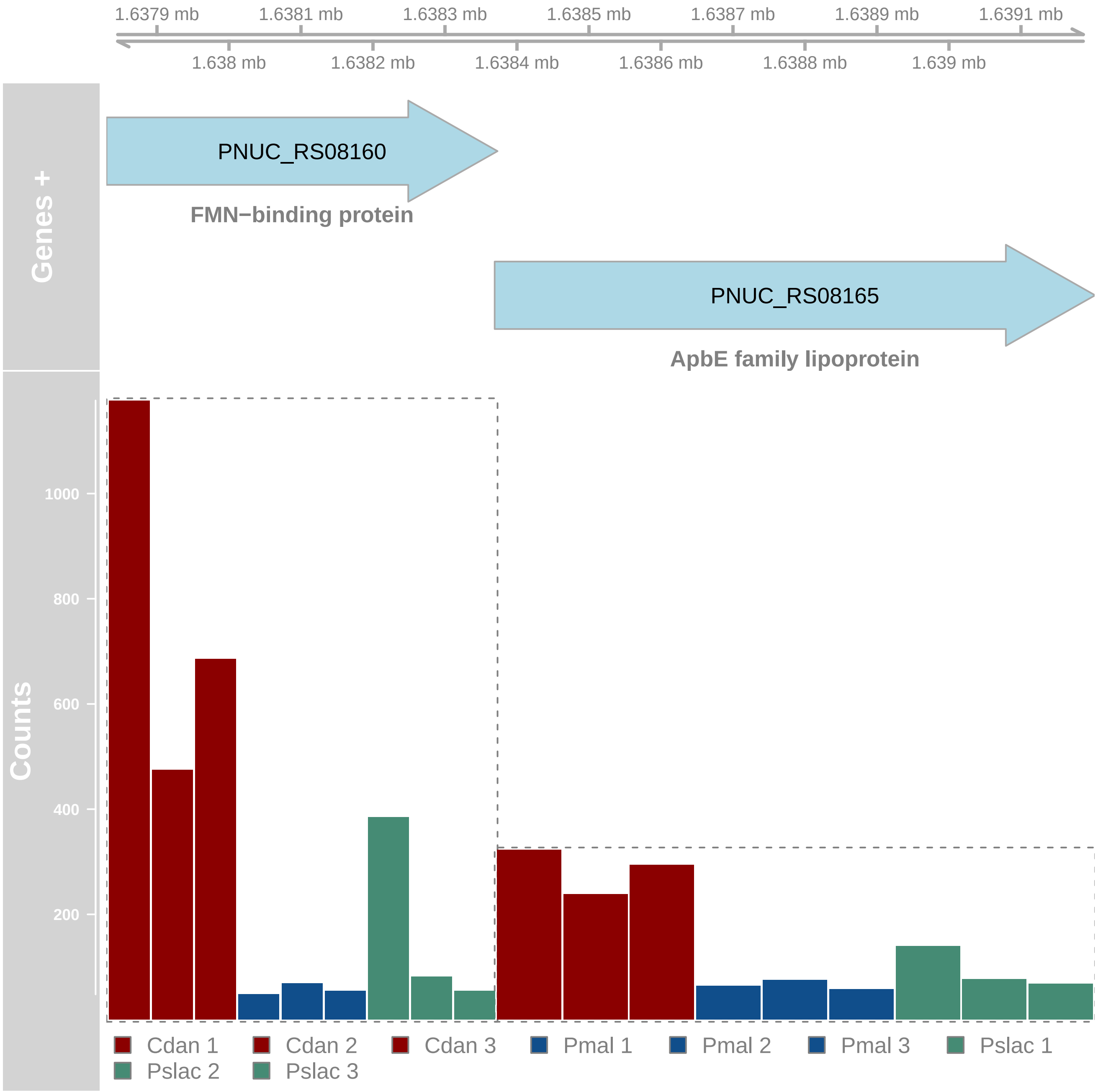

1.6379 mb
1.6381 mb
1.6383 mb
1.6385 mb
1.6387 mb
1.6389 mb
1.6391 mb
1.638 mb
1.6382 mb
1.6384 mb
1.6386 mb
1.6388 mb
1.639 mb
Genes +
PNUC_RS08160
FMN−binding protein
PNUC_RS08165
ApbE family lipoprotein
Counts
1000
800
600
400
200
Cdan 1
Cdan 2
Cdan 3
Pmal 1
Pmal 2
Pmal 3
Pslac 1
Pslac 2
Pslac 3

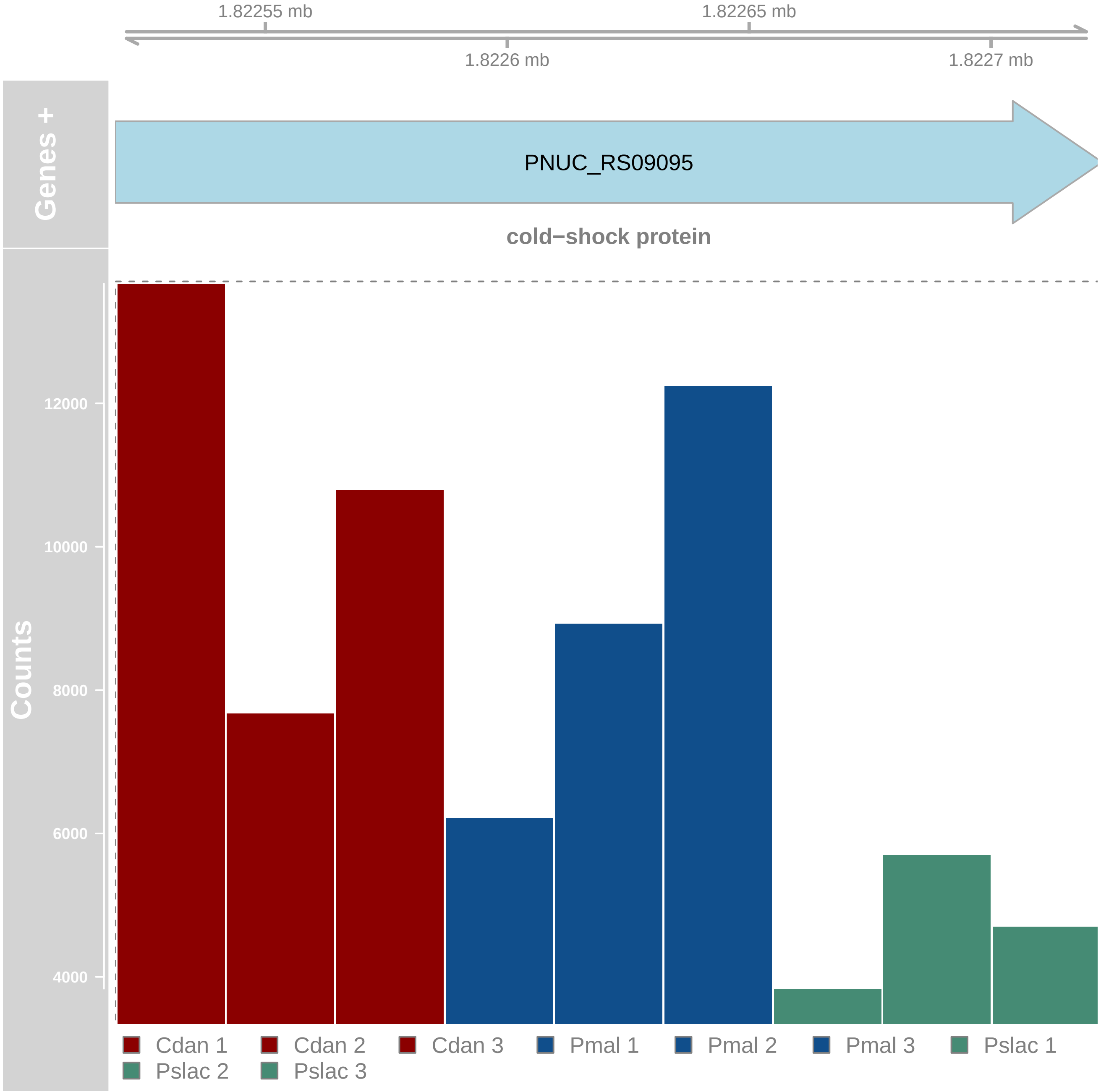
1.82255 mb
1.82265 mb
1.8226 mb
1.8227 mb
Genes +
PNUC_RS09095
cold−shock protein
Counts
12000
10000
8000
6000
4000
Cdan 1
Cdan 2
Cdan 3
Pmal 1
Pmal 2
Pmal 3
Pslac 1
Pslac 2
Pslac 3

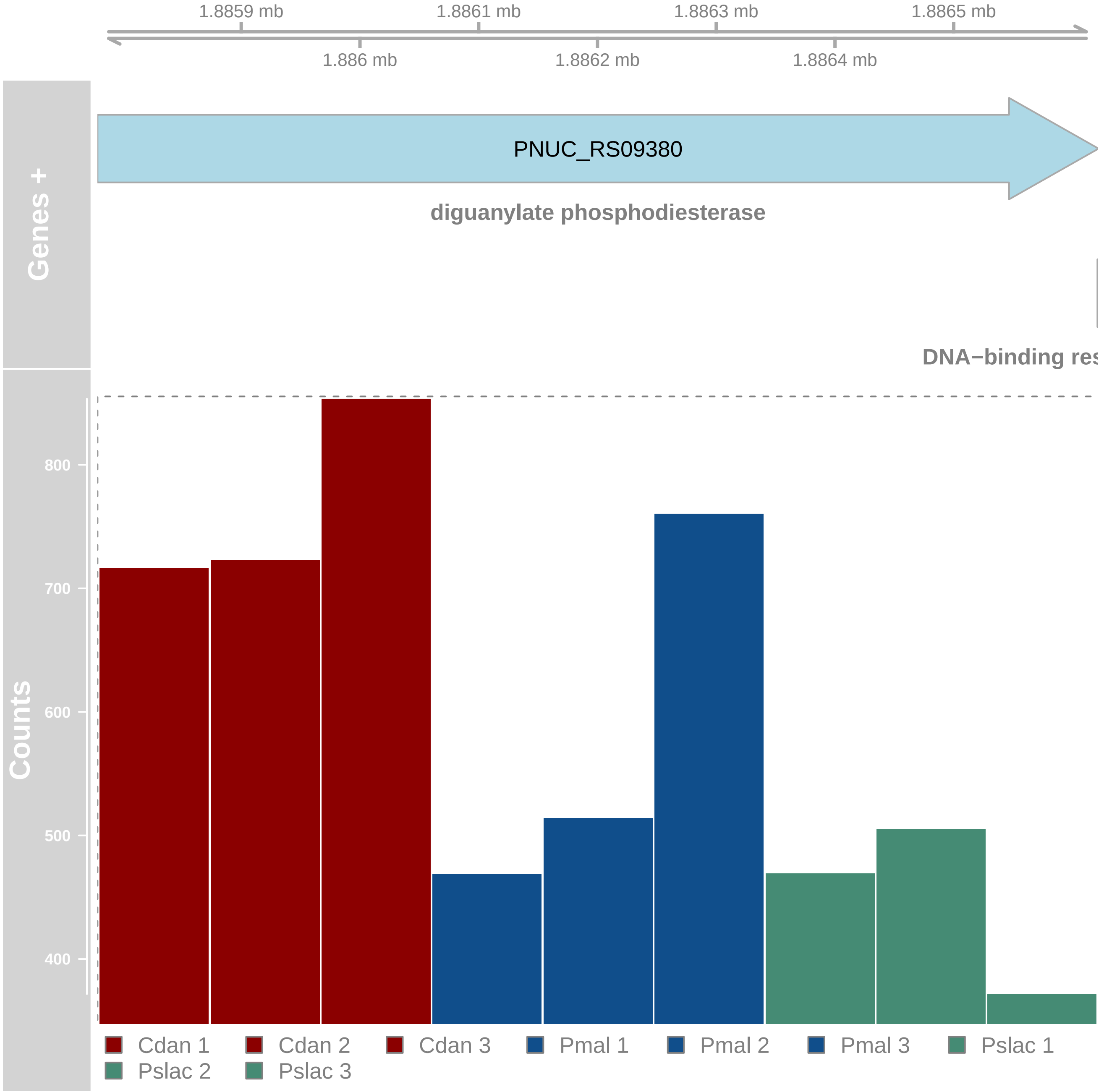
1.8859 mb
1.8861 mb
1.8863 mb
1.8865 mb
1.886 mb
1.8862 mb
1.8864 mb
Genes +
PNUC_RS09380
diguanylate phosphodiesterase
DNA−binding res
Counts
800
700
600
500
400
Cdan 1
Cdan 2
Cdan 3
Pmal 1
Pmal 2
Pmal 3
Pslac 1
Pslac 2
Pslac 3

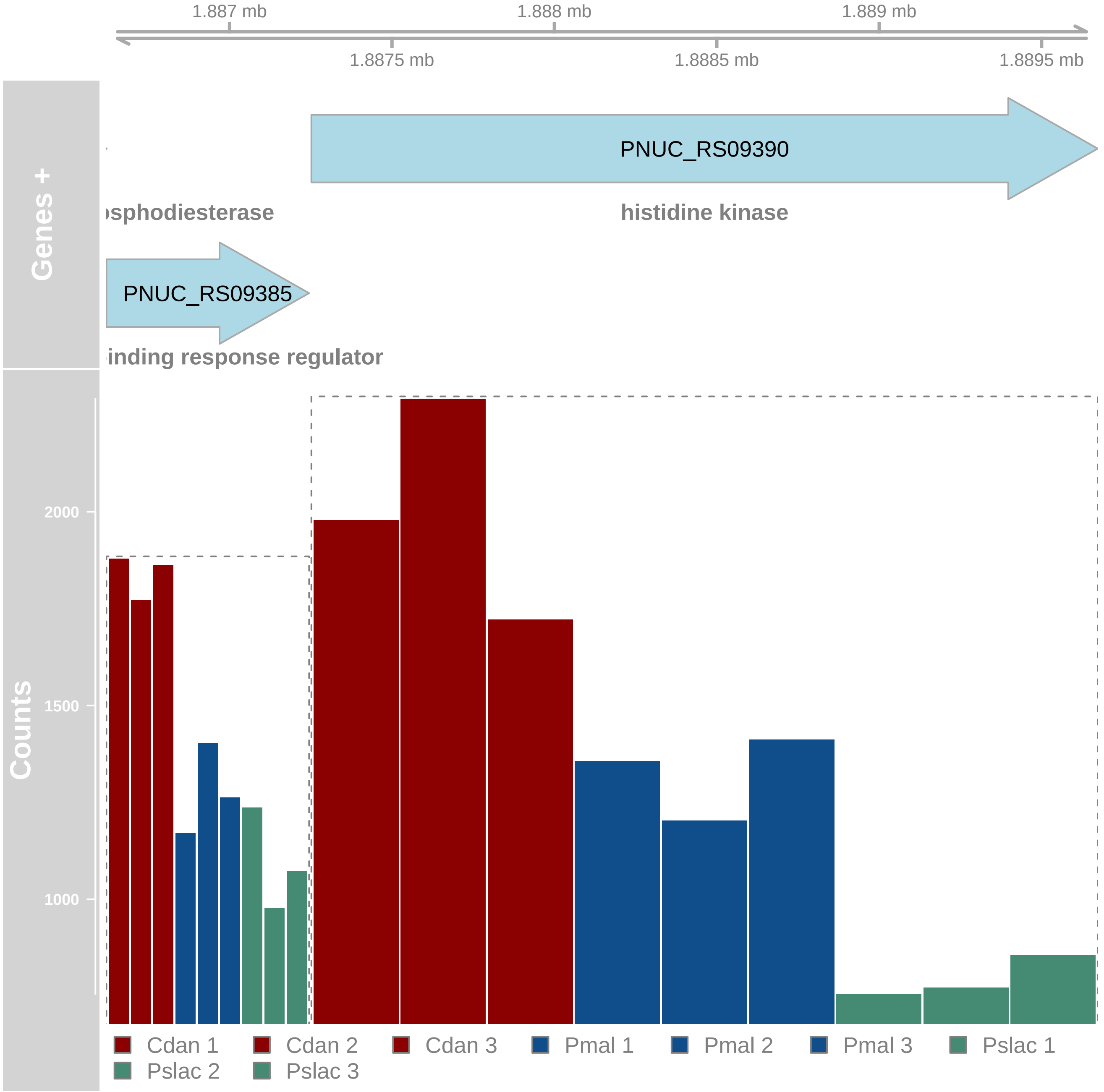

1.887 mb
1.888 mb
1.889 mb
1.8875 mb
1.8885 mb
1.8895 mb
Genes +
PNUC_RS09390
osphodiesterase
histidine kinase
PNUC_RS09385
inding response regulator
Counts
2000
1500
1000
Cdan 1
Cdan 2
Cdan 3
Pmal 1
Pmal 2
Pmal 3
Pslac 1
Pslac 2
Pslac 3

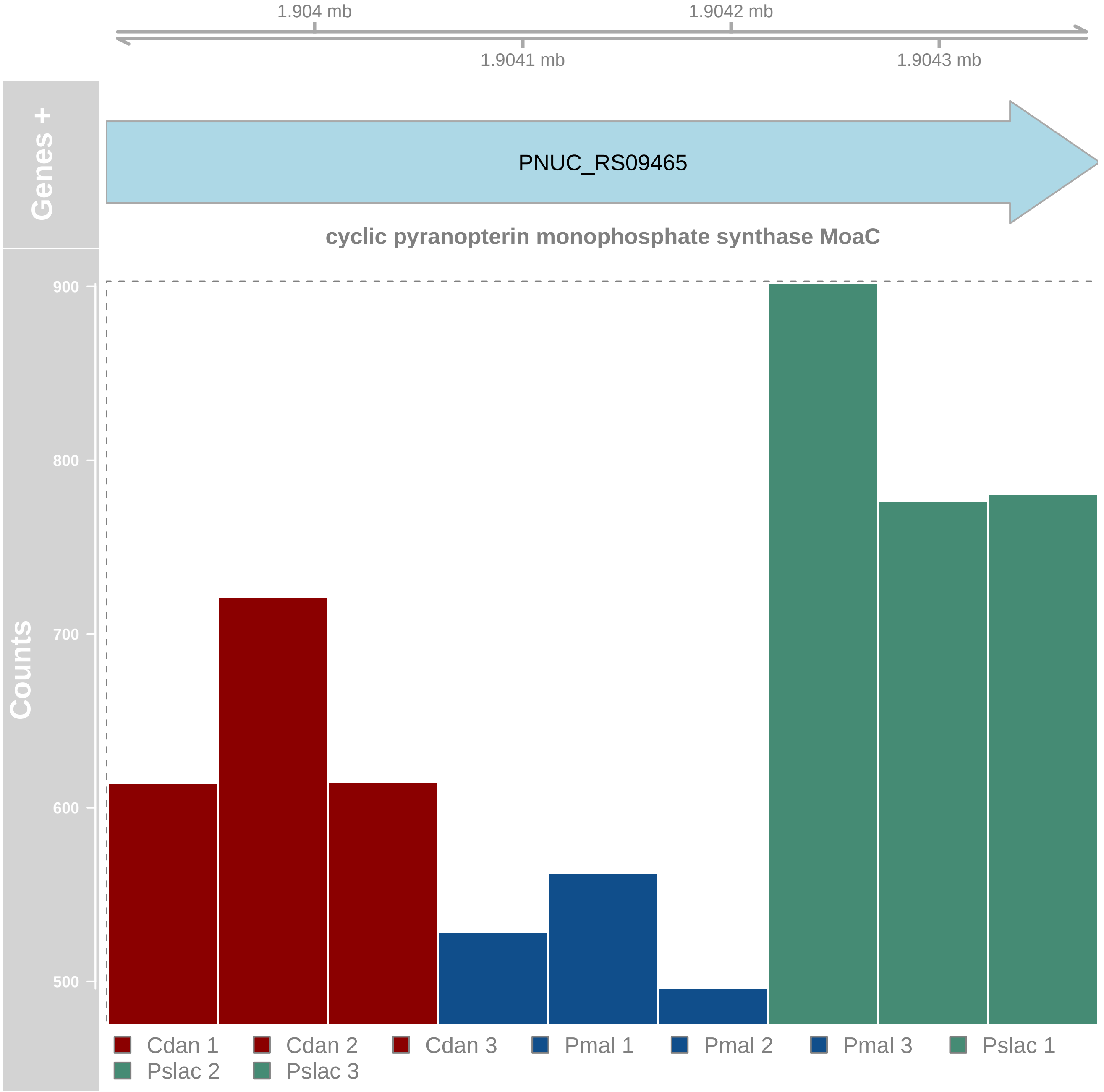

1.904 mb
1.9042 mb
1.9041 mb
1.9043 mb
Genes +
PNUC_RS09465
cyclic pyranopterin monophosphate synthase MoaC
Counts
900
800
700
600
500
Cdan 1
Cdan 2
Cdan 3
Pmal 1
Pmal 2
Pmal 3
Pslac 1
Pslac 2
Pslac 3

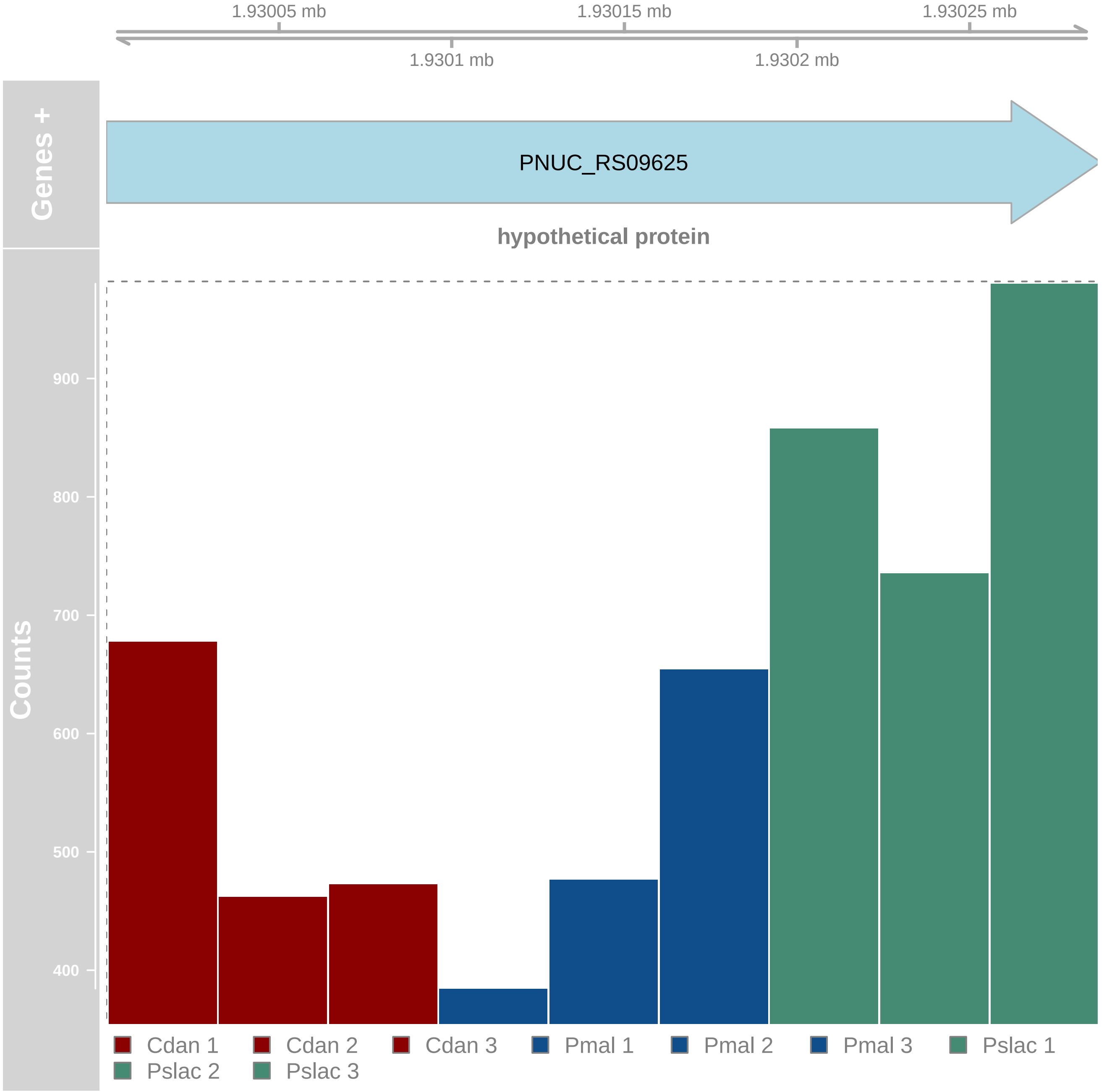

1.93005 mb
1.93015 mb
1.93025 mb
1.9301 mb
1.9302 mb
Genes +
PNUC_RS09625
hypothetical protein
Counts
900
800
700
600
500
400
Cdan 1
Cdan 2
Cdan 3
Pmal 1
Pmal 2
Pmal 3
Pslac 1
Pslac 2
Pslac 3

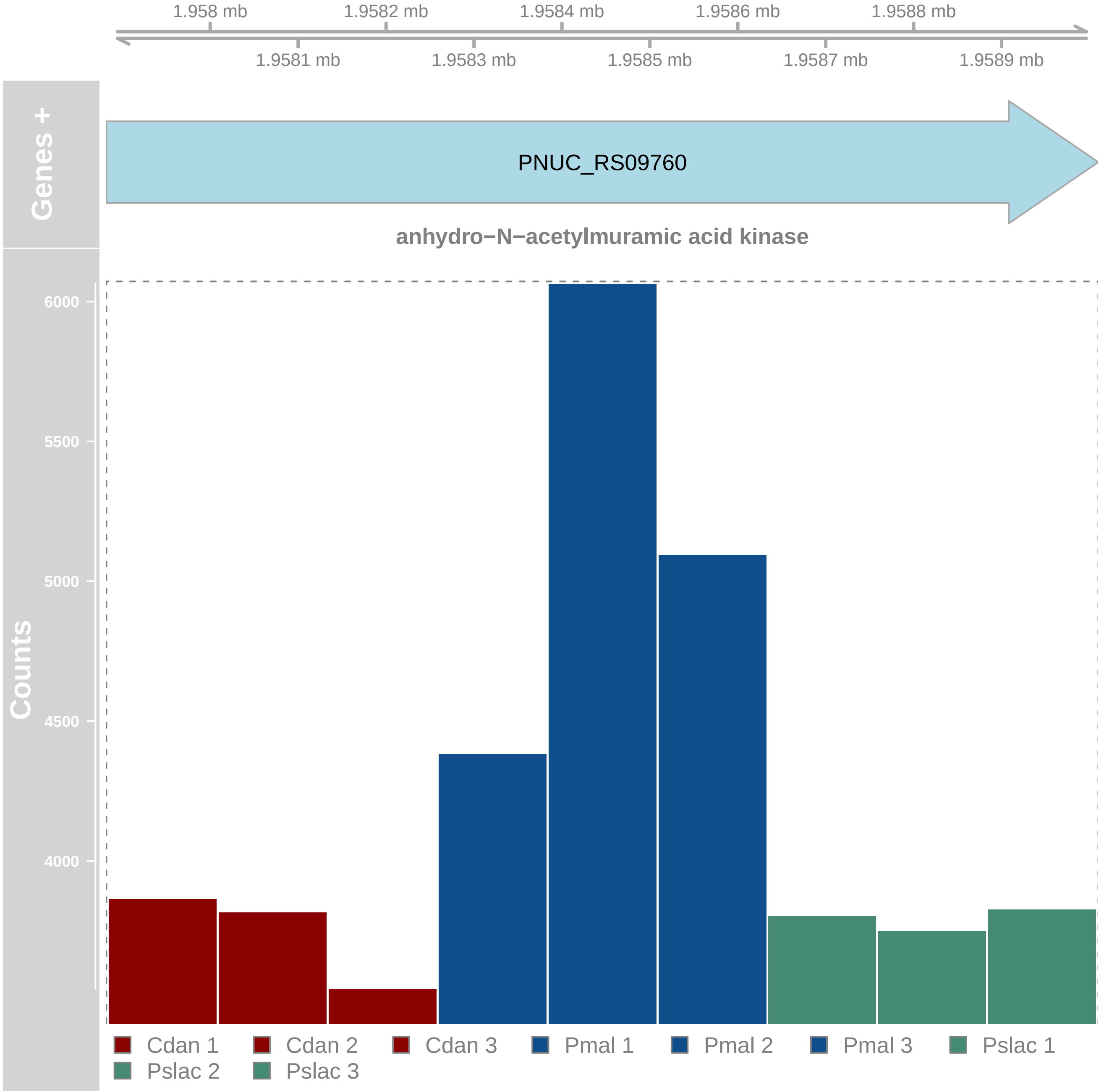

1.958 mb
1.9581 mb
1.9582 mb
1.9583 mb
1.9584 mb
1.9585 mb
1.9586 mb
1.9587 mb
1.9588 mb
1.9589 mb
Genes +
PNUC_RS09760
anhydro−N−acetylmuramic acid kinase
Counts
6000
5500
5000
4500
4000
Cdan 1
Cdan 2
Cdan 3
Pmal 1
Pmal 2
Pmal 3
Pslac 1
Pslac 2
Pslac 3

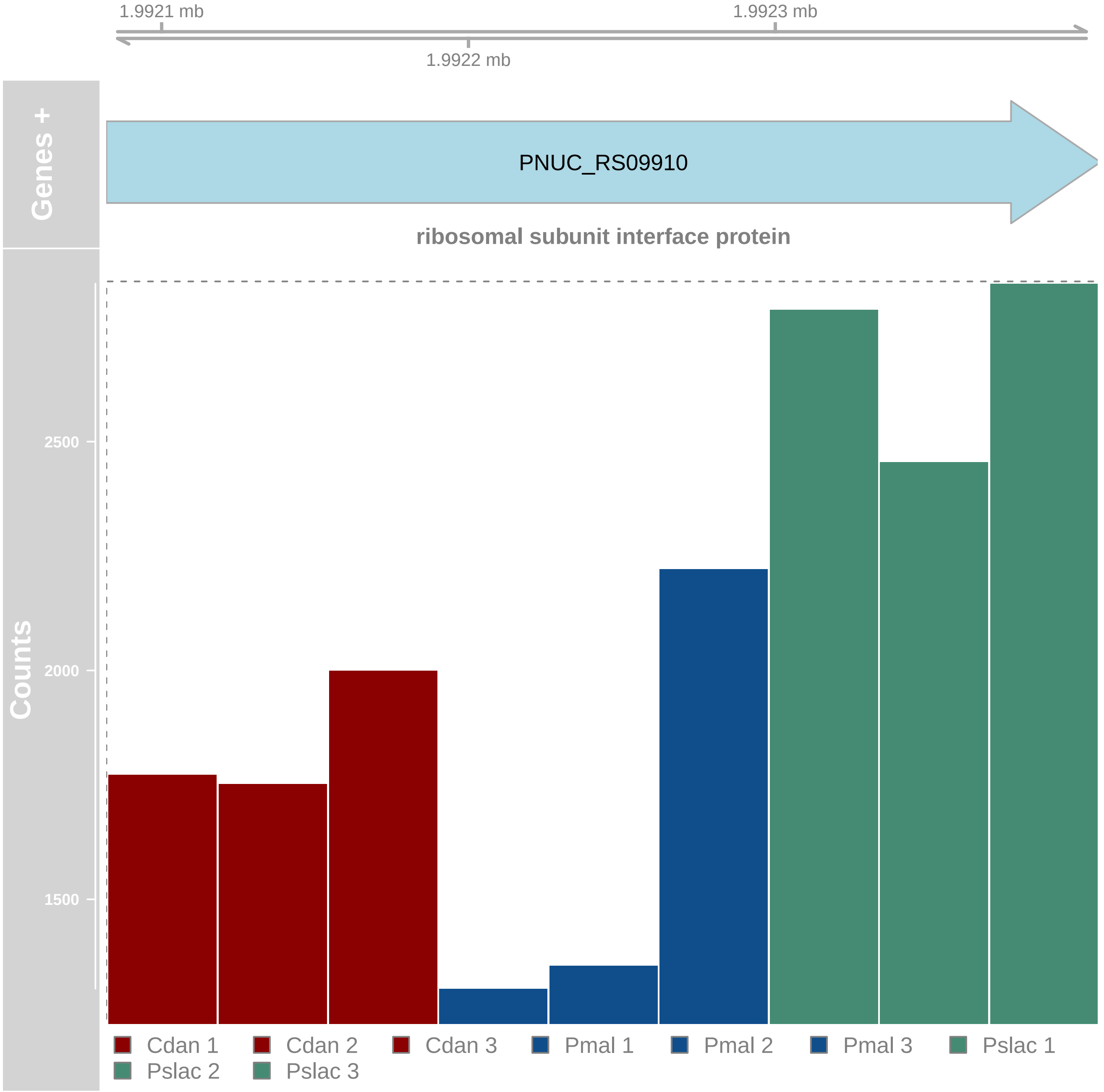

1.9921 mb
1.9922 mb
1.9923 mb
Genes +
PNUC_RS09910
ribosomal subunit interface protein
Counts
2500
2000
1500
Cdan 1
Cdan 2
Cdan 3
Pmal 1
Pmal 2
Pmal 3
Pslac 1
Pslac 2
Pslac 3

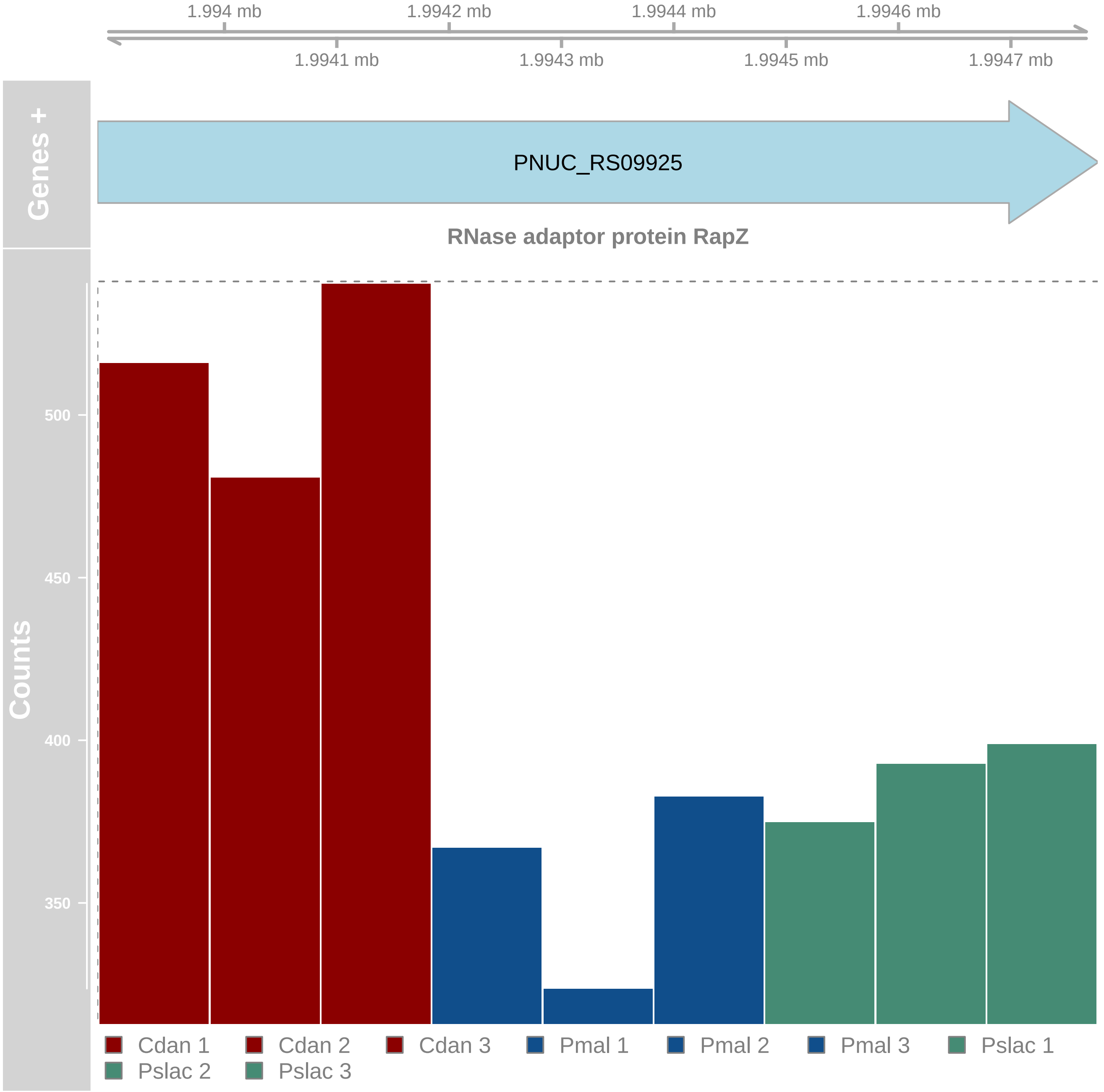
1.994 mb
1.9941 mb
1.9942 mb
1.9943 mb
1.9944 mb
1.9945 mb
1.9946 mb
1.9947 mb
Genes +
PNUC_RS09925
RNase adaptor protein RapZ
Counts
500
450
400
350
Cdan 1
Cdan 2
Cdan 3
Pmal 1
Pmal 2
Pmal 3
Pslac 1
Pslac 2
Pslac 3

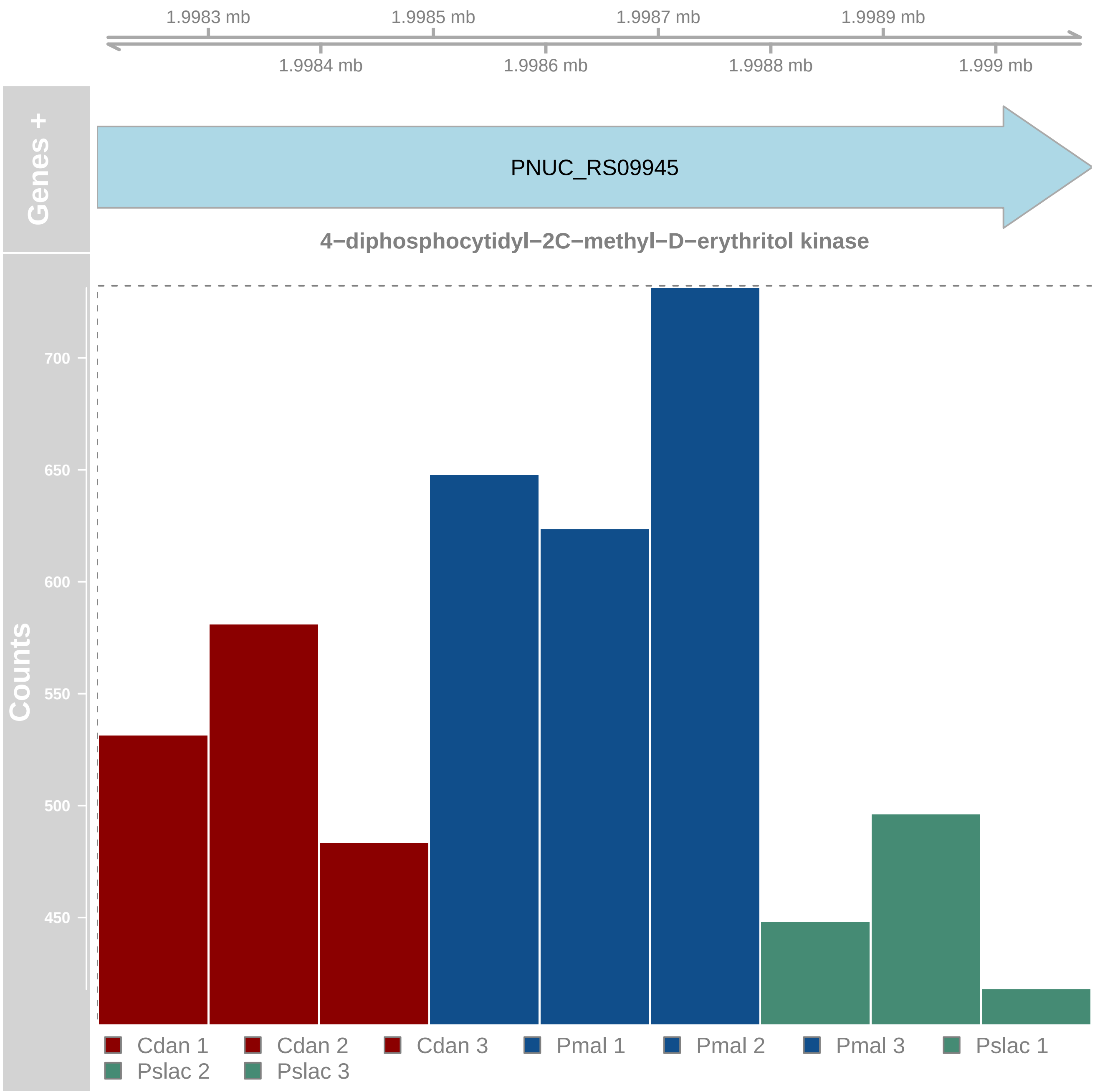

1.9983 mb
1.9984 mb
1.9985 mb
1.9986 mb
1.9987 mb
1.9988 mb
1.9989 mb
1.999 mb
Genes +
PNUC_RS09945
4−diphosphocytidyl−2C−methyl−D−erythritol kinase
Counts
700
650
600
550
500
450
Cdan 1
Cdan 2
Cdan 3
Pmal 1
Pmal 2
Pmal 3
Pslac 1
Pslac 2
Pslac 3

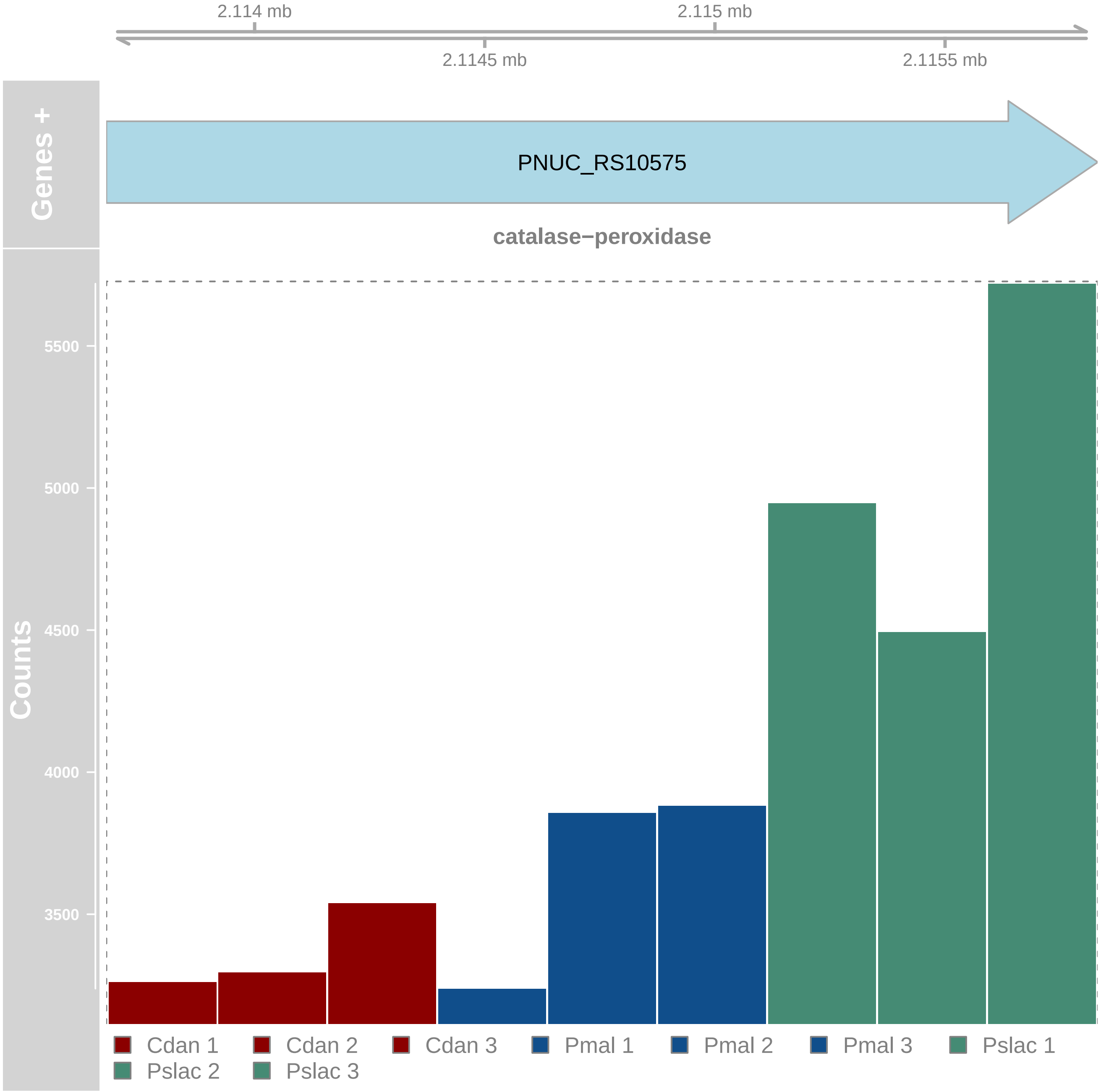
2.114 mb
2.115 mb
2.1145 mb
2.1155 mb
Genes +
PNUC_RS10575
catalase−peroxidase
Counts
5500
5000
4500
4000
3500
Cdan 1
Cdan 2
Cdan 3
Pmal 1
Pmal 2
Pmal 3
Pslac 1
Pslac 2
Pslac 3

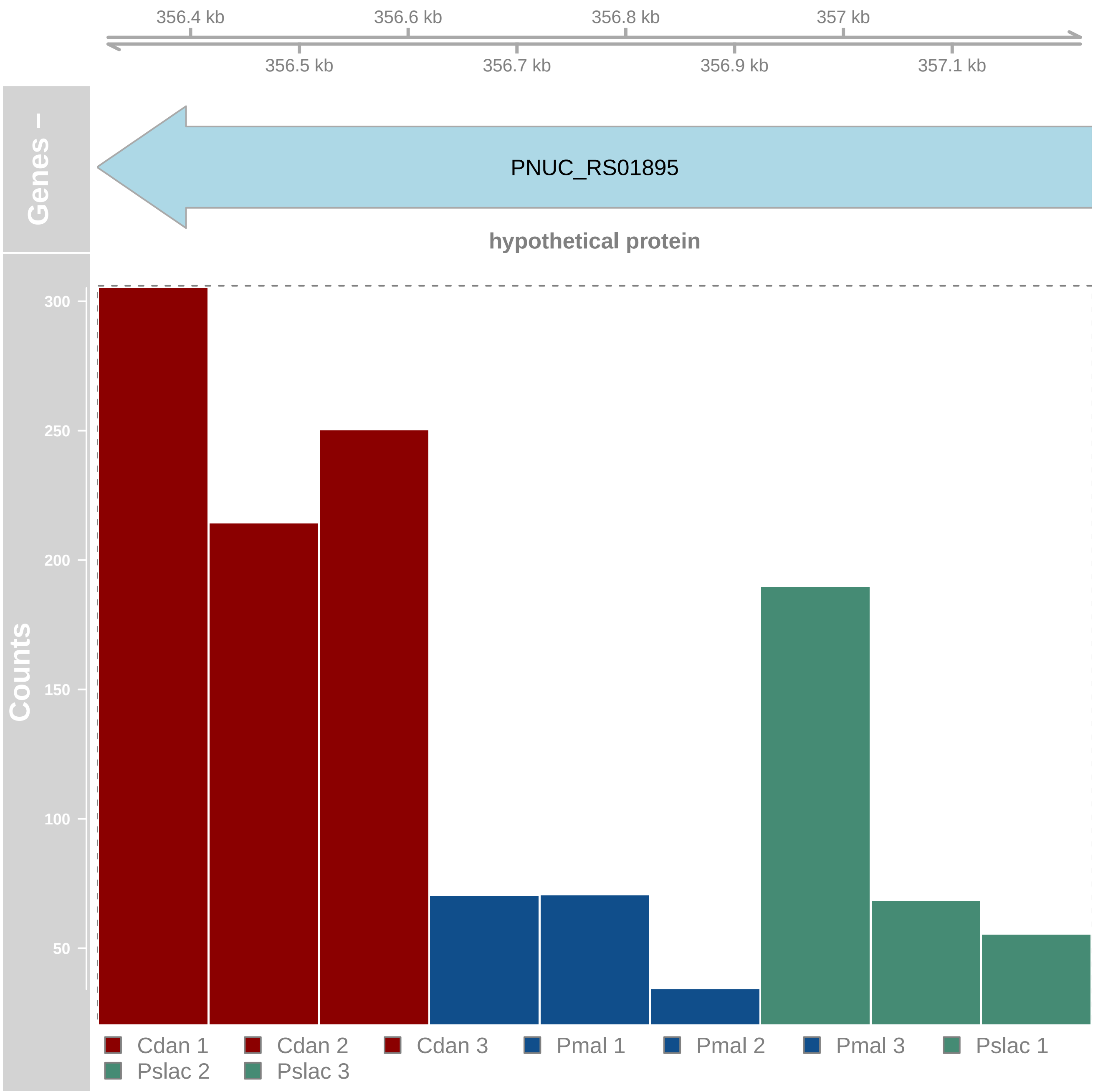
356.4 kb
356.6 kb
356.8 kb
357 kb
356.5 kb
356.7 kb
356.9 kb
357.1 kb
Genes –
PNUC_RS01895
hypothetical protein
Counts
300
250
200
150
100
50
Cdan 1
Cdan 2
Cdan 3
Pmal 1
Pmal 2
Pmal 3
Pslac 1
Pslac 2
Pslac 3

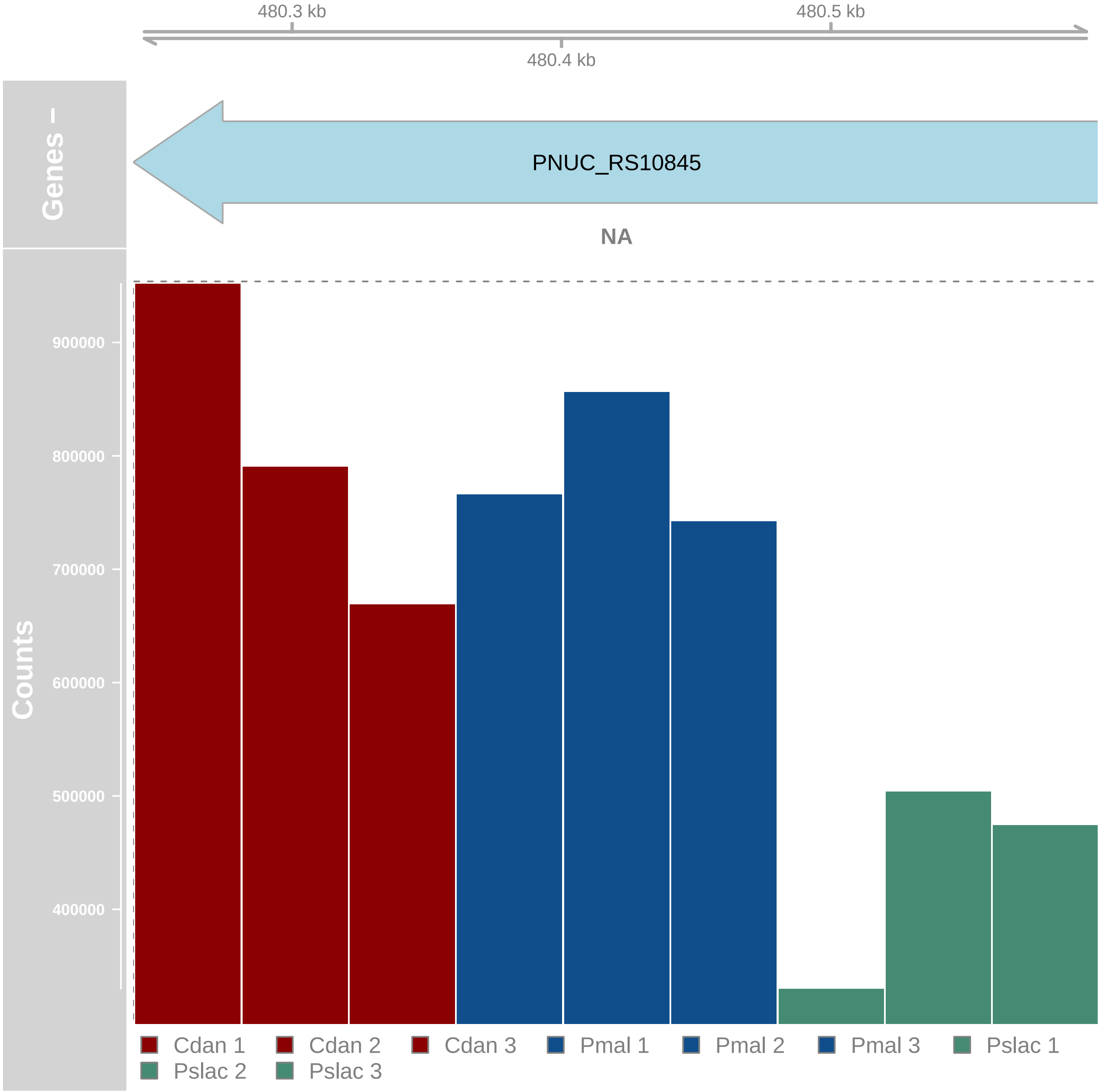
480.3 kb
480.4 kb
480.5 kb
Genes –
PNUC_RS10845
NA
Counts
900000
800000
700000
600000
500000
400000
Cdan 1
Cdan 2
Cdan 3
Pmal 1
Pmal 2
Pmal 3
Pslac 1
Pslac 2
Pslac 3

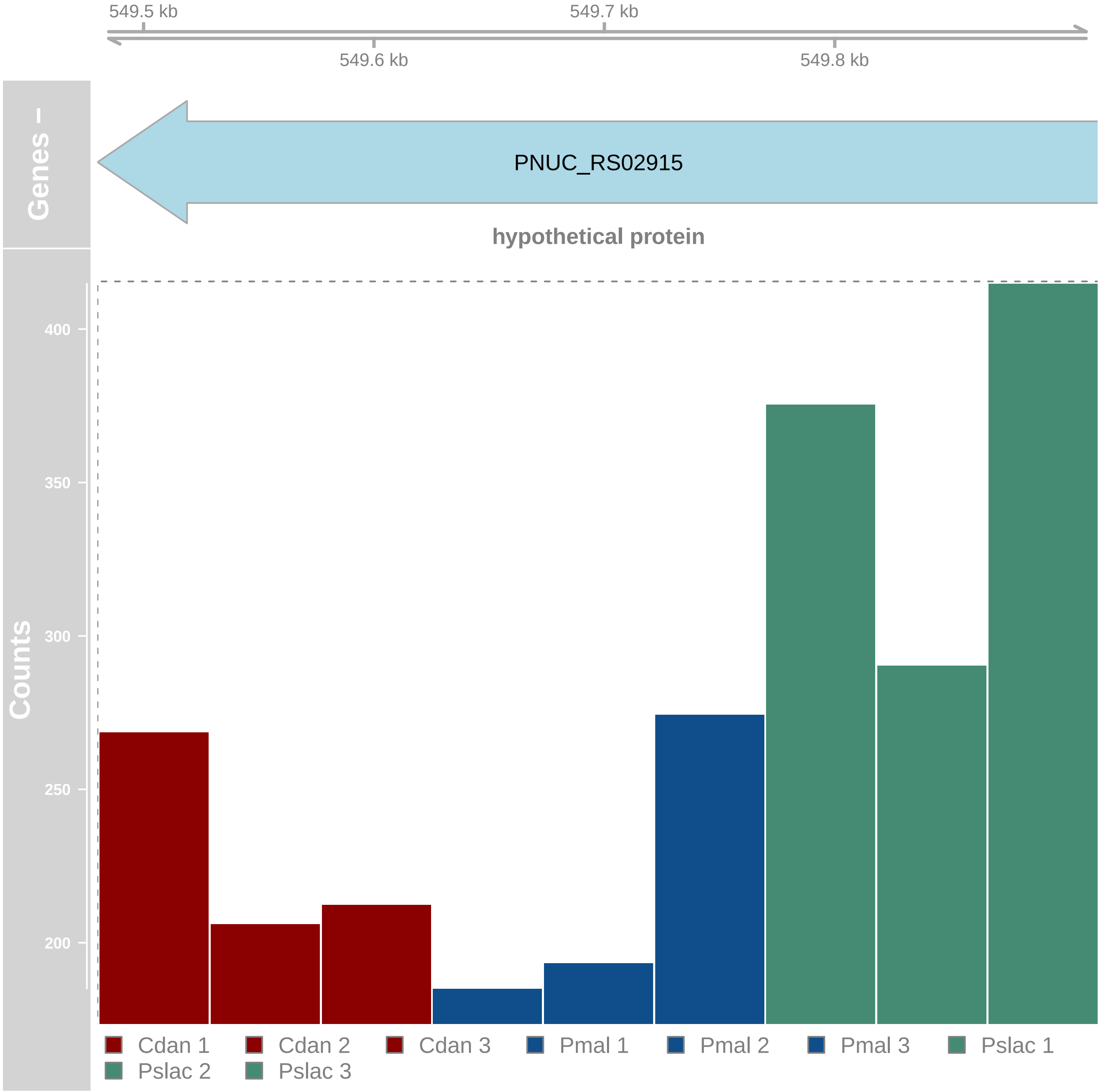
549.5 kb
549.6 kb
549.7 kb
549.8 kb
Genes –
PNUC_RS02915
hypothetical protein
Counts
400
350
300
250
200
Cdan 1
Cdan 2
Cdan 3
Pmal 1
Pmal 2
Pmal 3
Pslac 1
Pslac 2
Pslac 3

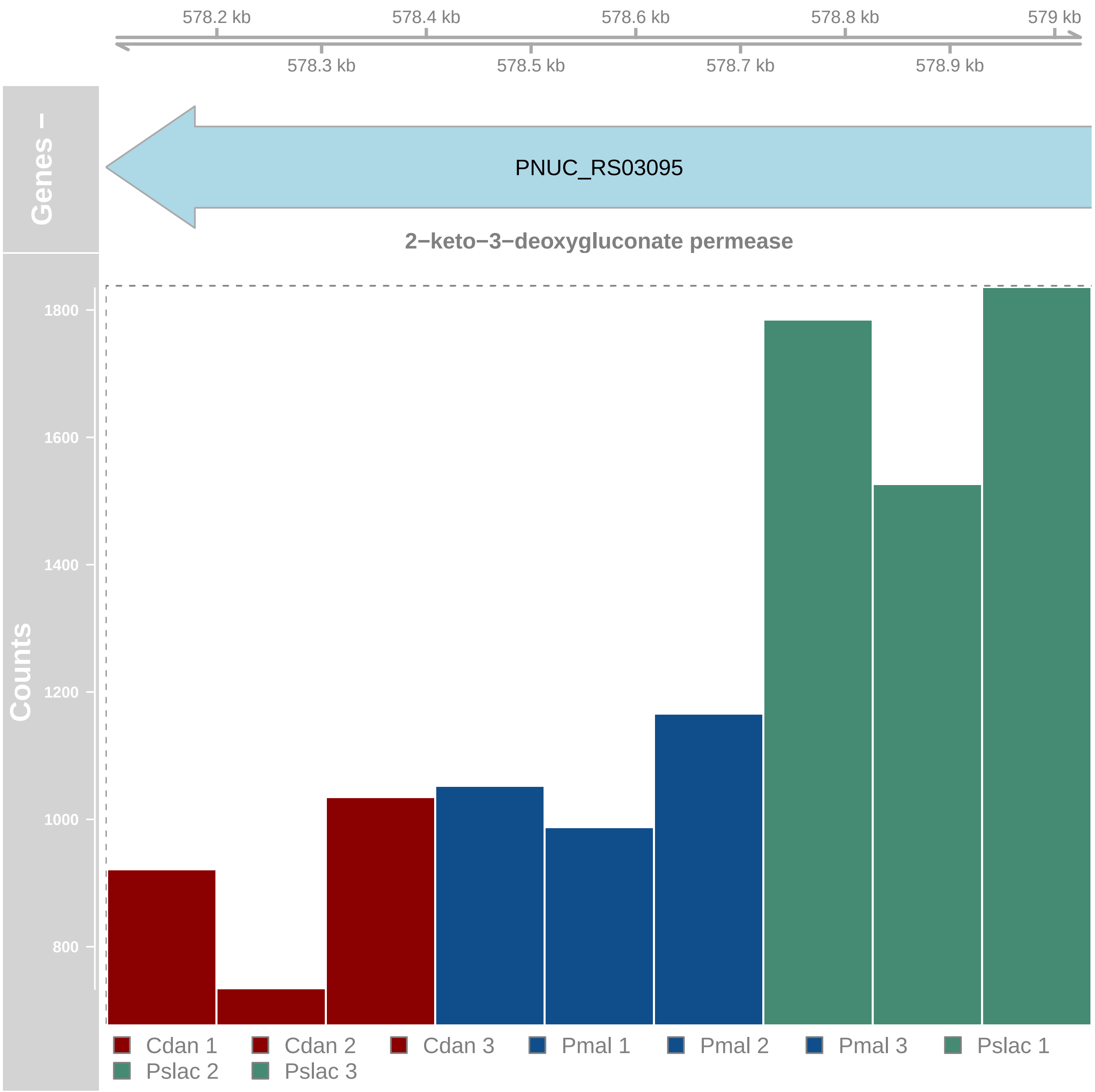
578.2 kb
578.4 kb
578.6 kb
578.8 kb
579 kb
578.3 kb
578.5 kb
578.7 kb
578.9 kb
Genes −
PNUC_RS03095
2−keto−3−deoxygluconate permease
Counts
1800
1600
1400
1200
1000
800
Cdan 1
Cdan 2
Cdan 3
Pmal 1
Pmal 2
Pmal 3
Pslac 1
Pslac 2
Pslac 3

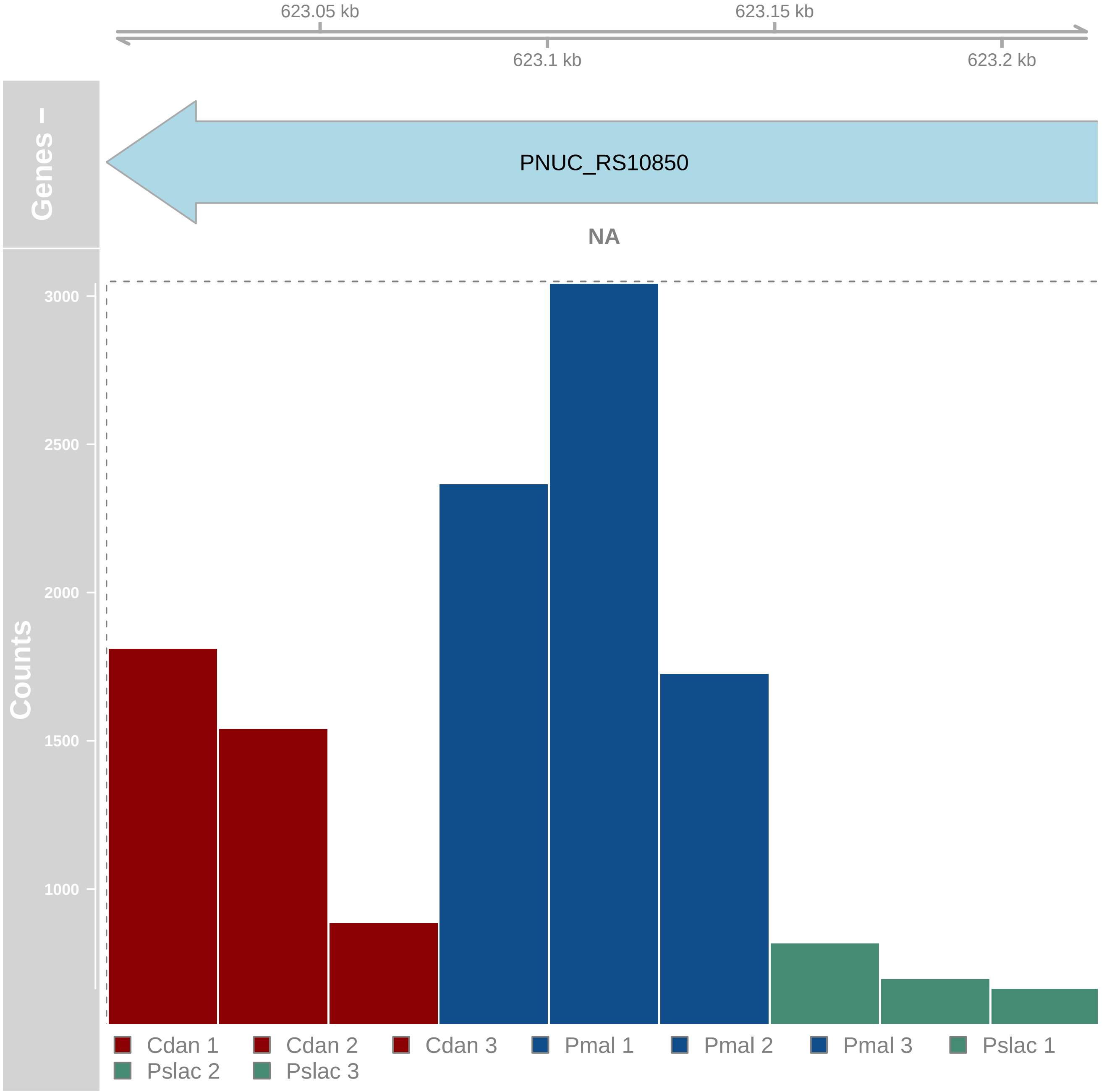

623.05 kb
623.15 kb
623.1 kb
623.2 kb
Genes –
PNUC_RS10850
NA
Counts
3000
2500
2000
1500
1000
Cdan 1
Cdan 2
Cdan 3
Pmal 1
Pmal 2
Pmal 3
Pslac 1
Pslac 2
Pslac 3

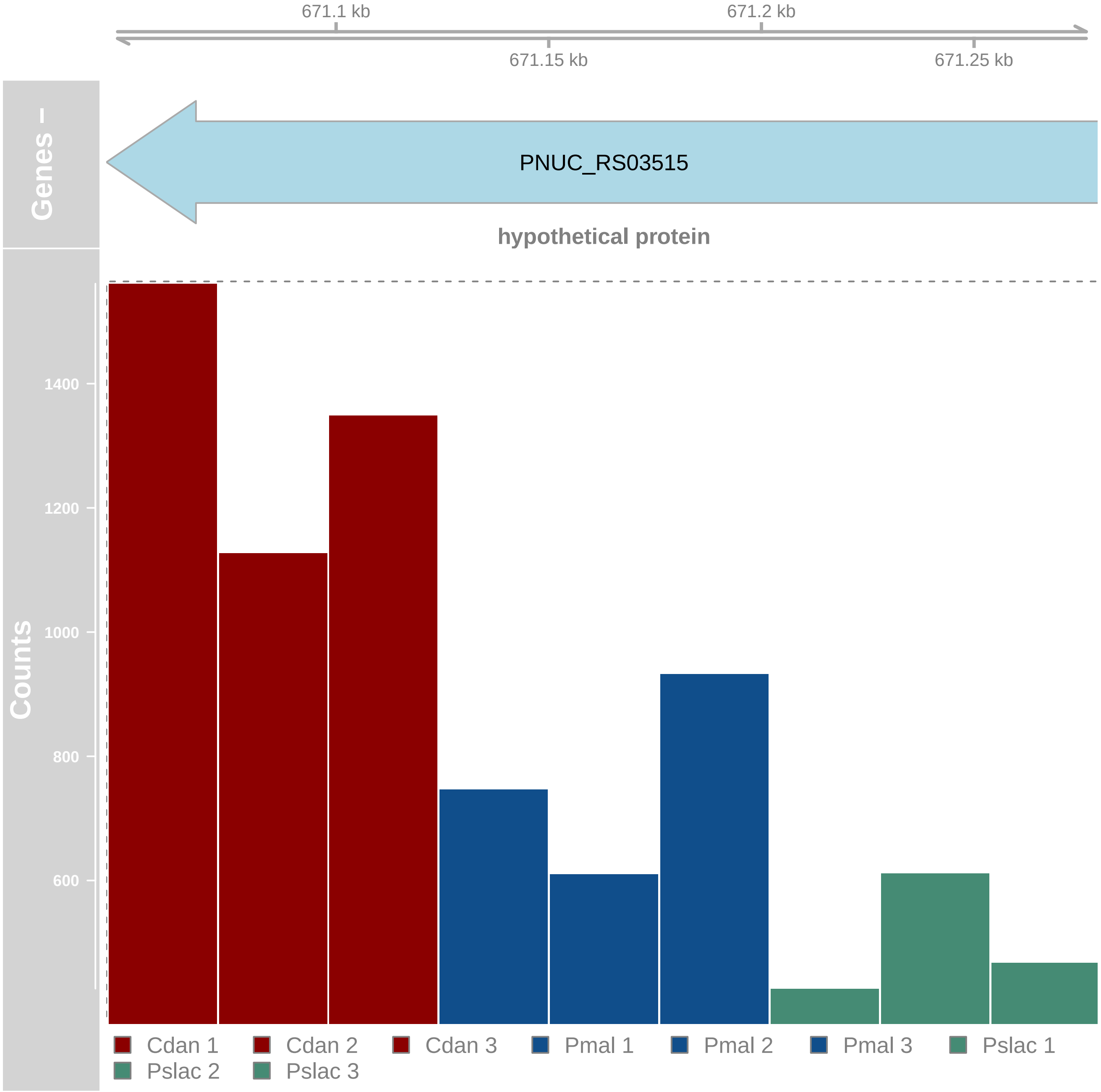

671.1 kb
671.2 kb
671.15 kb
671.25 kb
Genes -
PNUC_RS03515
hypothetical protein
Counts
1400
1200
1000
800
600
Cdan 1
Cdan 2
Cdan 3
Pmal 1
Pmal 2
Pmal 3
Pslac 1
Pslac 2
Pslac 3

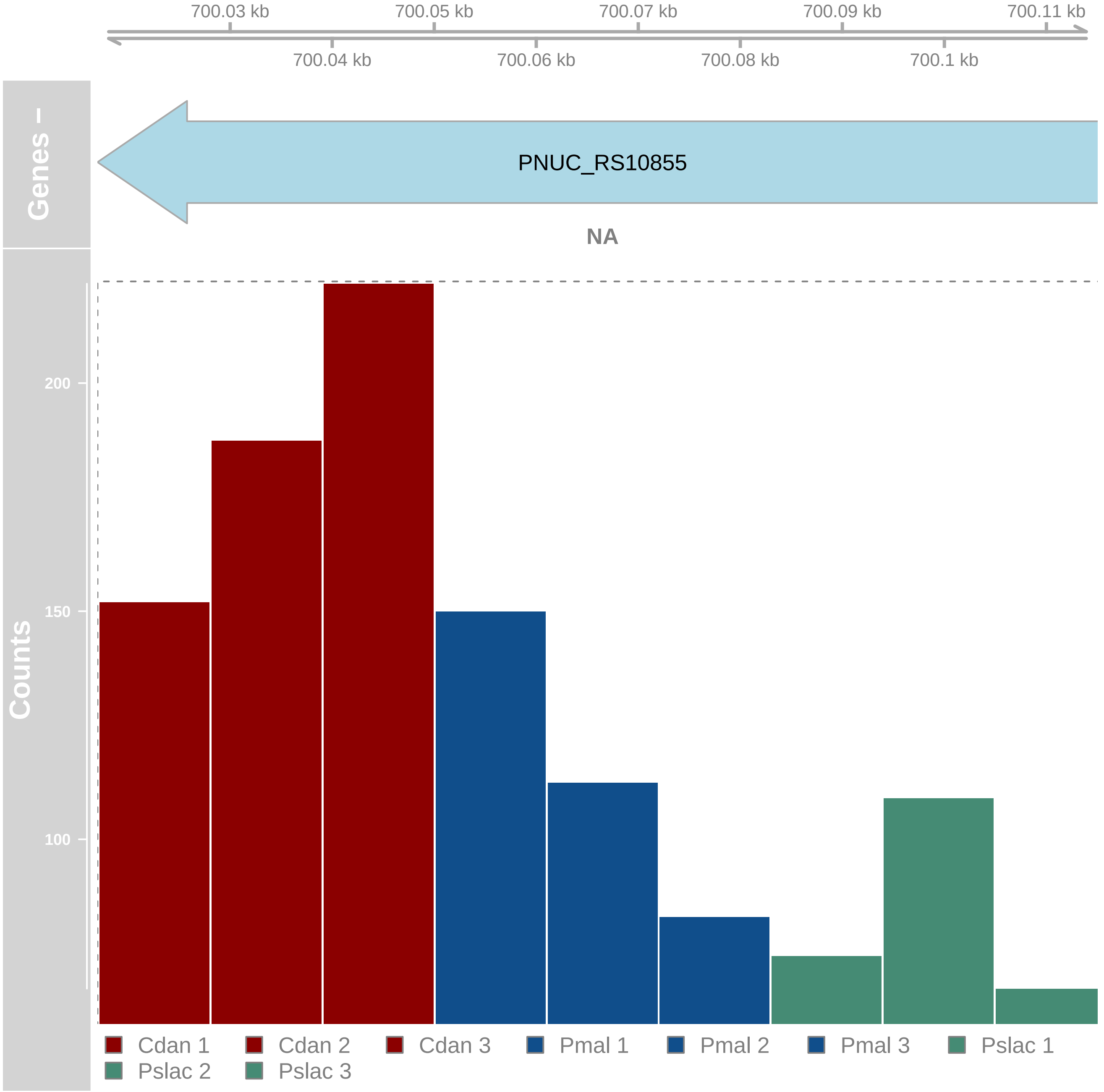

700.03 kb
700.05 kb
700.07 kb
700.09 kb
700.11 kb
700.04 kb
700.06 kb
700.08 kb
700.1 kb
Genes –
PNUC_RS10855
NA
Counts
200
150
100
Cdan 1
Cdan 2
Cdan 3
Pmal 1
Pmal 2
Pmal 3
Pslac 1
Pslac 2
Pslac 3

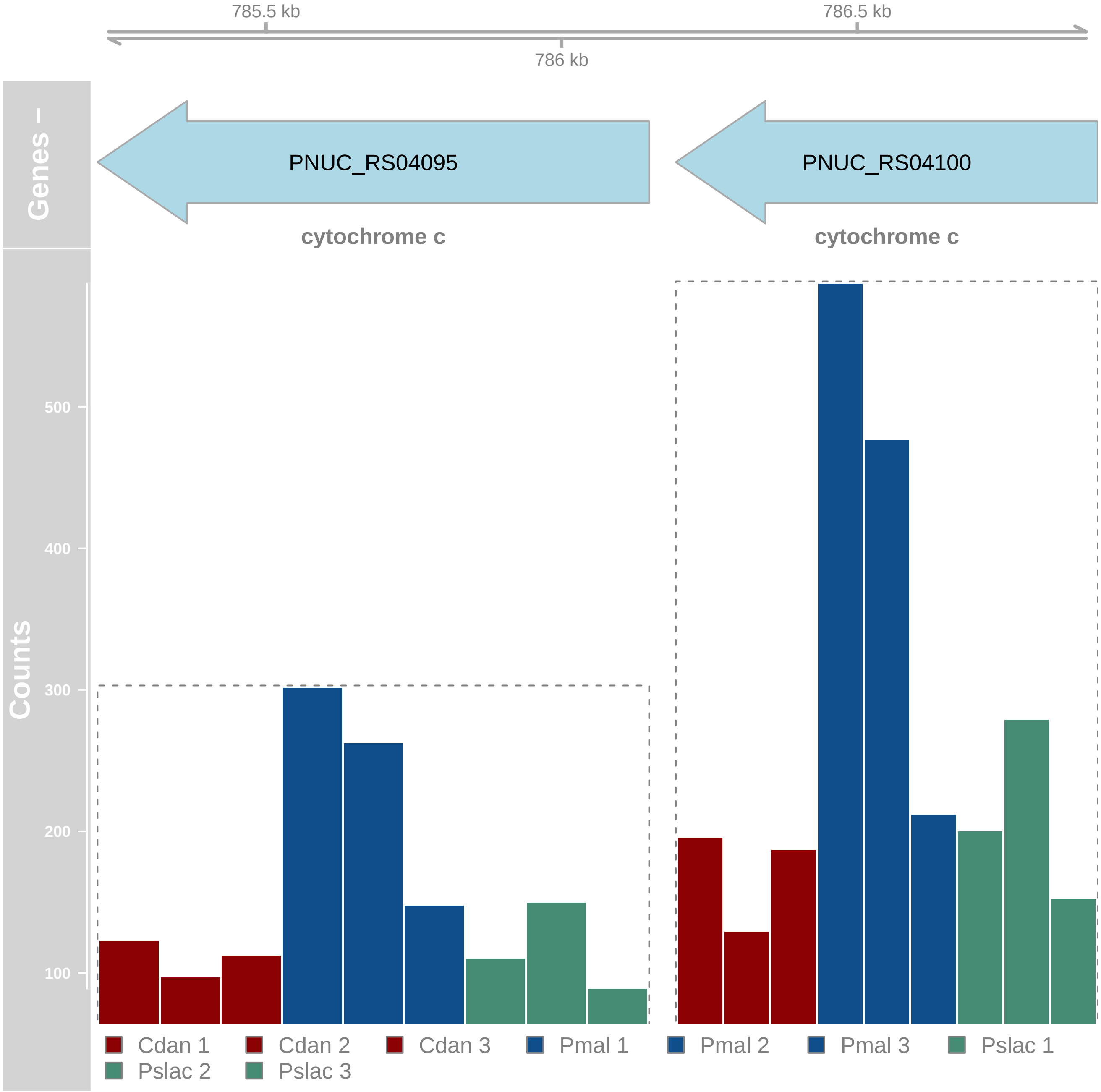

785.5 kb
786.5 kb
786 kb
Genes –
PNUC_RS04095
cytochrome c
PNUC_RS04100
cytochrome c
Counts
500
400
300
200
100
Cdan 1
Cdan 2
Cdan 3
Pmal 1
Pmal 2
Pmal 3
Pslac 1
Pslac 2
Pslac 3

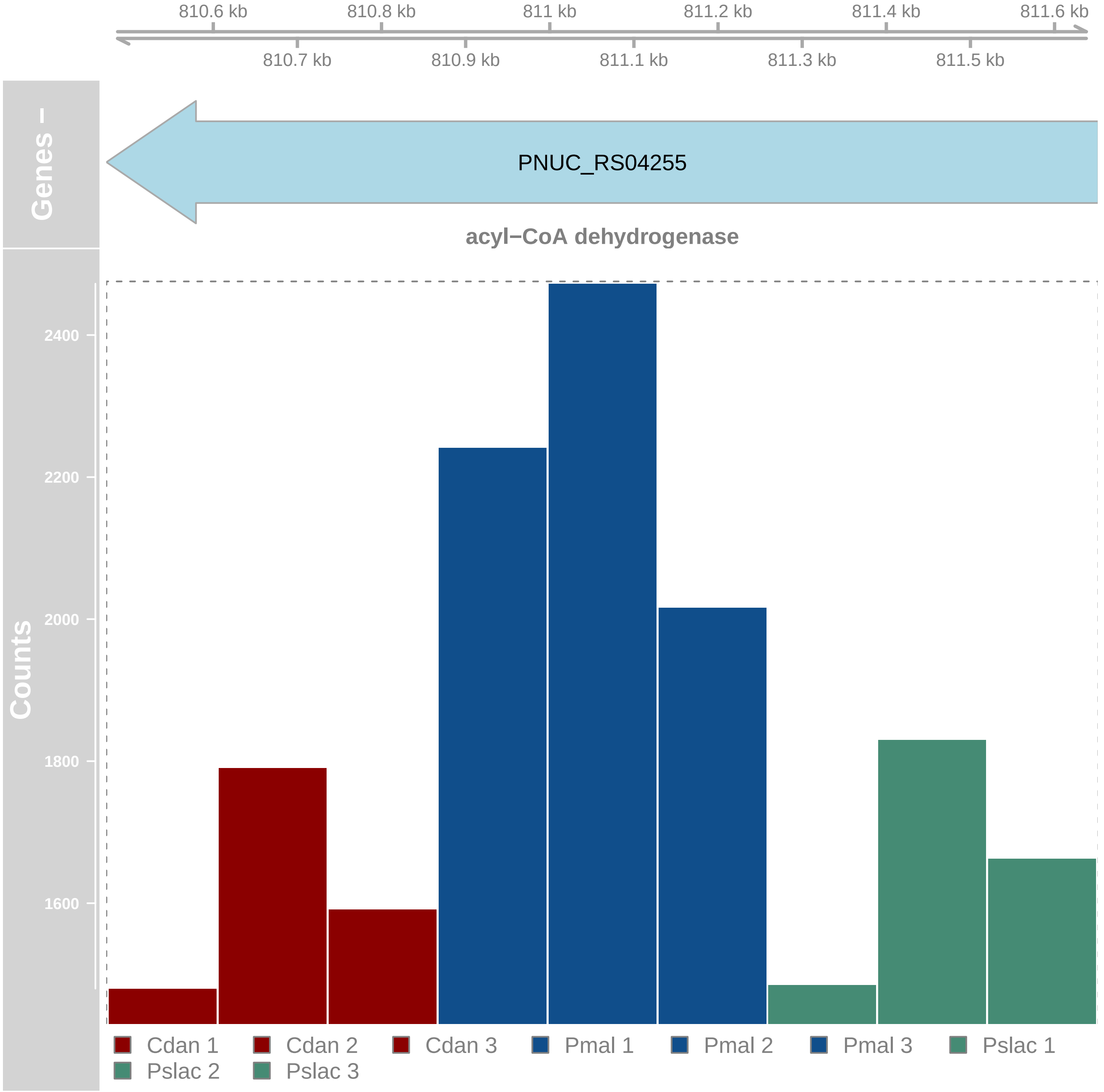

810.6 kb
810.8 kb
811 kb
811.2 kb
811.4 kb
811.6 kb
810.7 kb
810.9 kb
811.1 kb
811.3 kb
811.5 kb
Genes −
PNUC_RS04255
acyl−CoA dehydrogenase
Counts
2400
2200
2000
1800
1600
Cdan 1
Cdan 2
Cdan 3
Pmal 1
Pmal 2
Pmal 3
Pslac 1
Pslac 2
Pslac 3

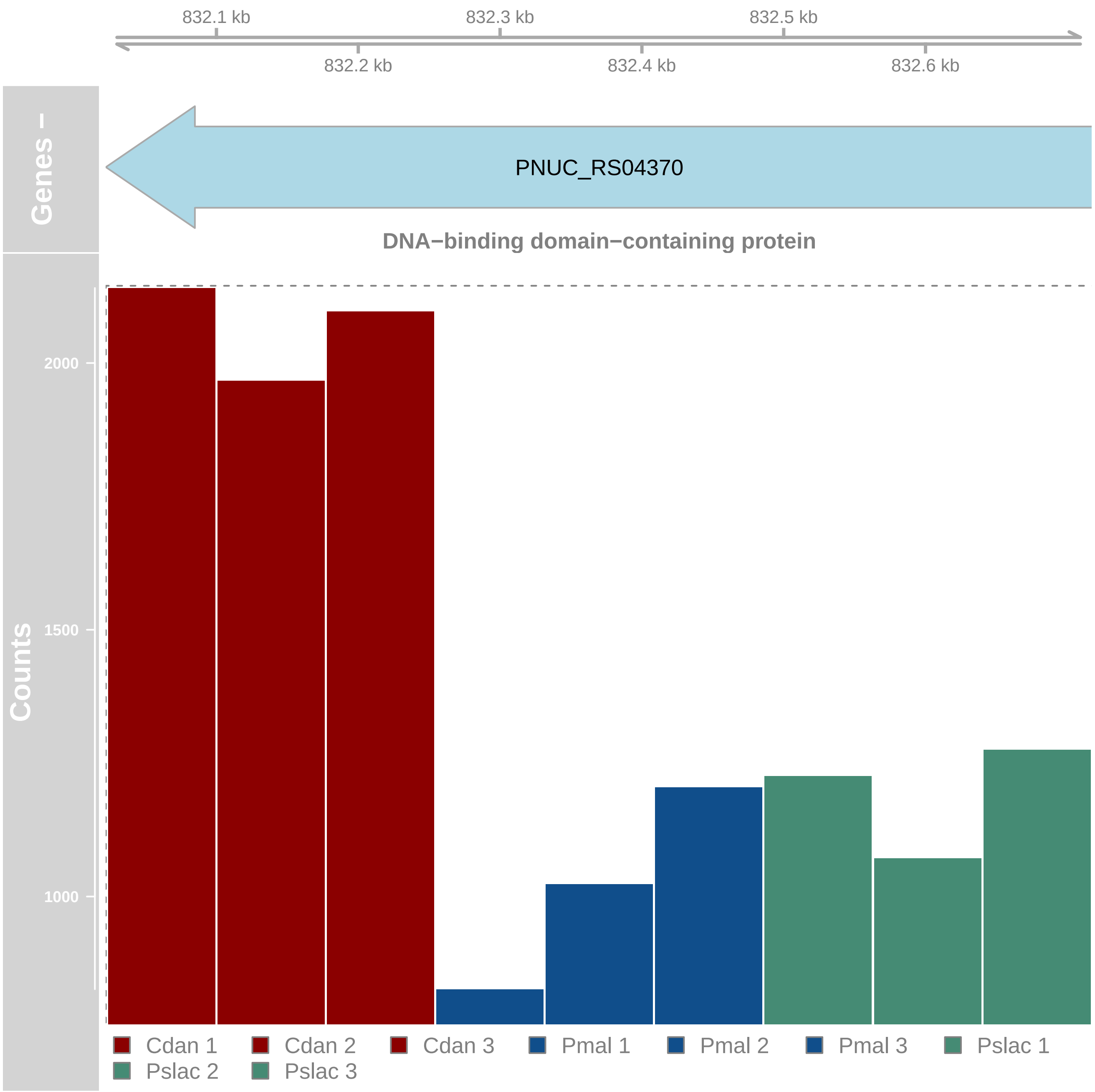

832.1 kb
832.2 kb
832.3 kb
832.4 kb
832.5 kb
832.6 kb
Genes −
PNUC_RS04370
DNA−binding domain−containing protein
Counts
2000
1500
1000
Cdan 1
Cdan 2
Cdan 3
Pmal 1
Pmal 2
Pmal 3
Pslac 1
Pslac 2
Pslac 3

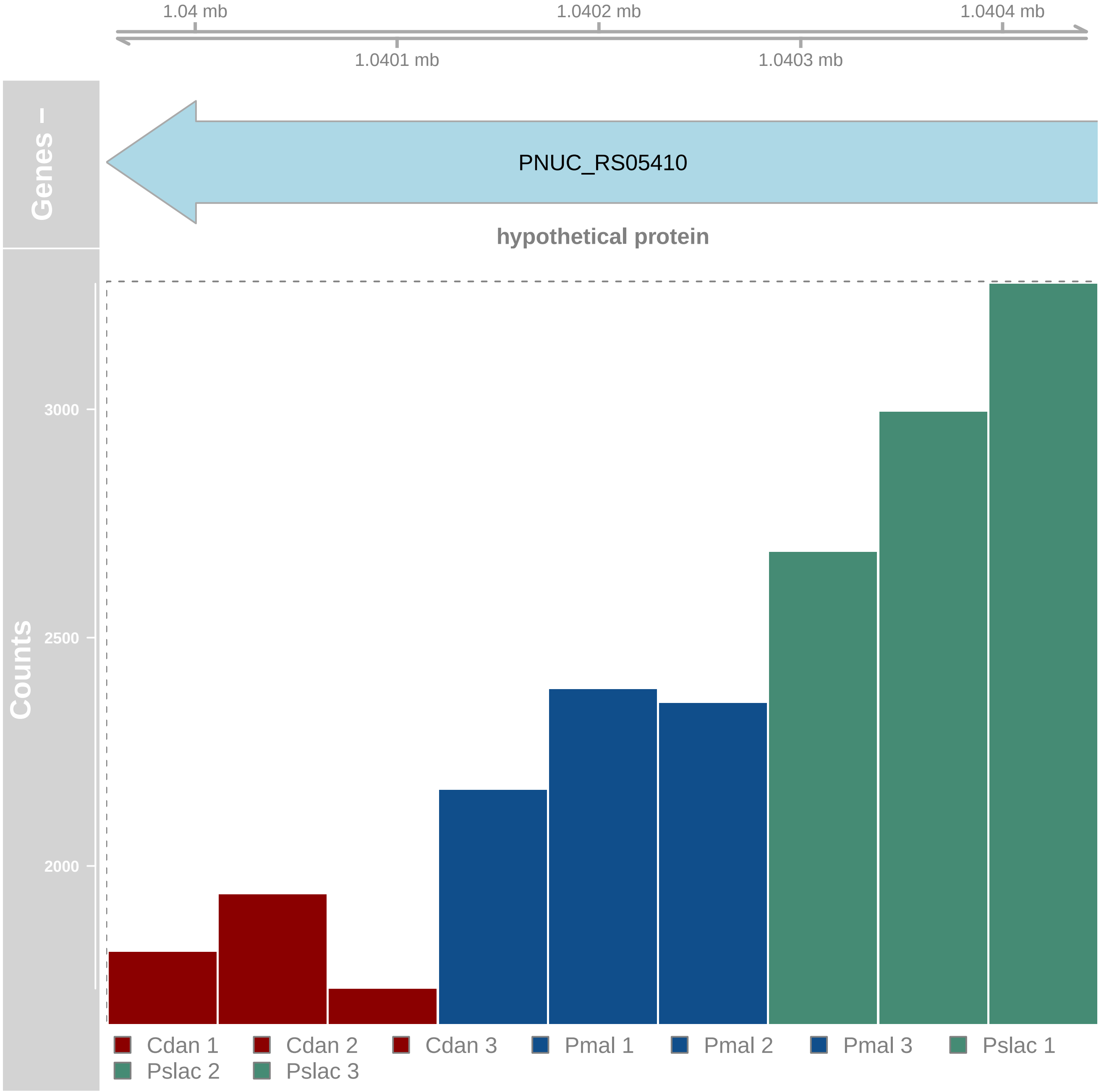
1.04 mb
1.0401 mb
1.0402 mb
1.0403 mb
1.0404 mb
Genes –
PNUC_RS05410
hypothetical protein
Counts
3000
2500
2000
Cdan 1
Cdan 2
Cdan 3
Pmal 1
Pmal 2
Pmal 3
Pslac 1
Pslac 2
Pslac 3

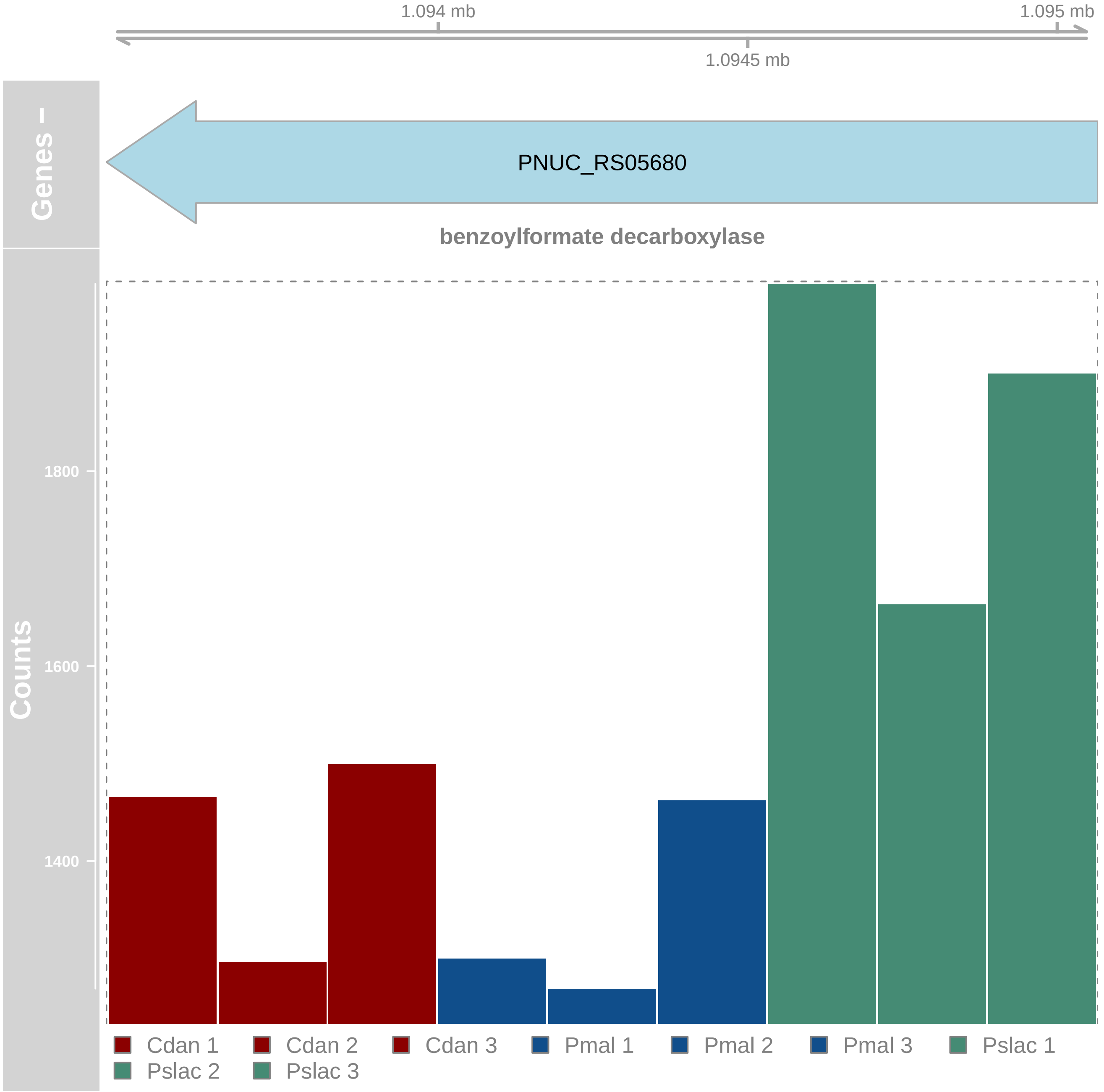
1.094 mb
1.095 mb
1.0945 mb
Genes –
PNUC_RS05680
benzoylformate decarboxylase
Counts
1800
1600
1400
Cdan 1
Cdan 2
Cdan 3
Pmal 1
Pmal 2
Pmal 3
Pslac 1
Pslac 2
Pslac 3

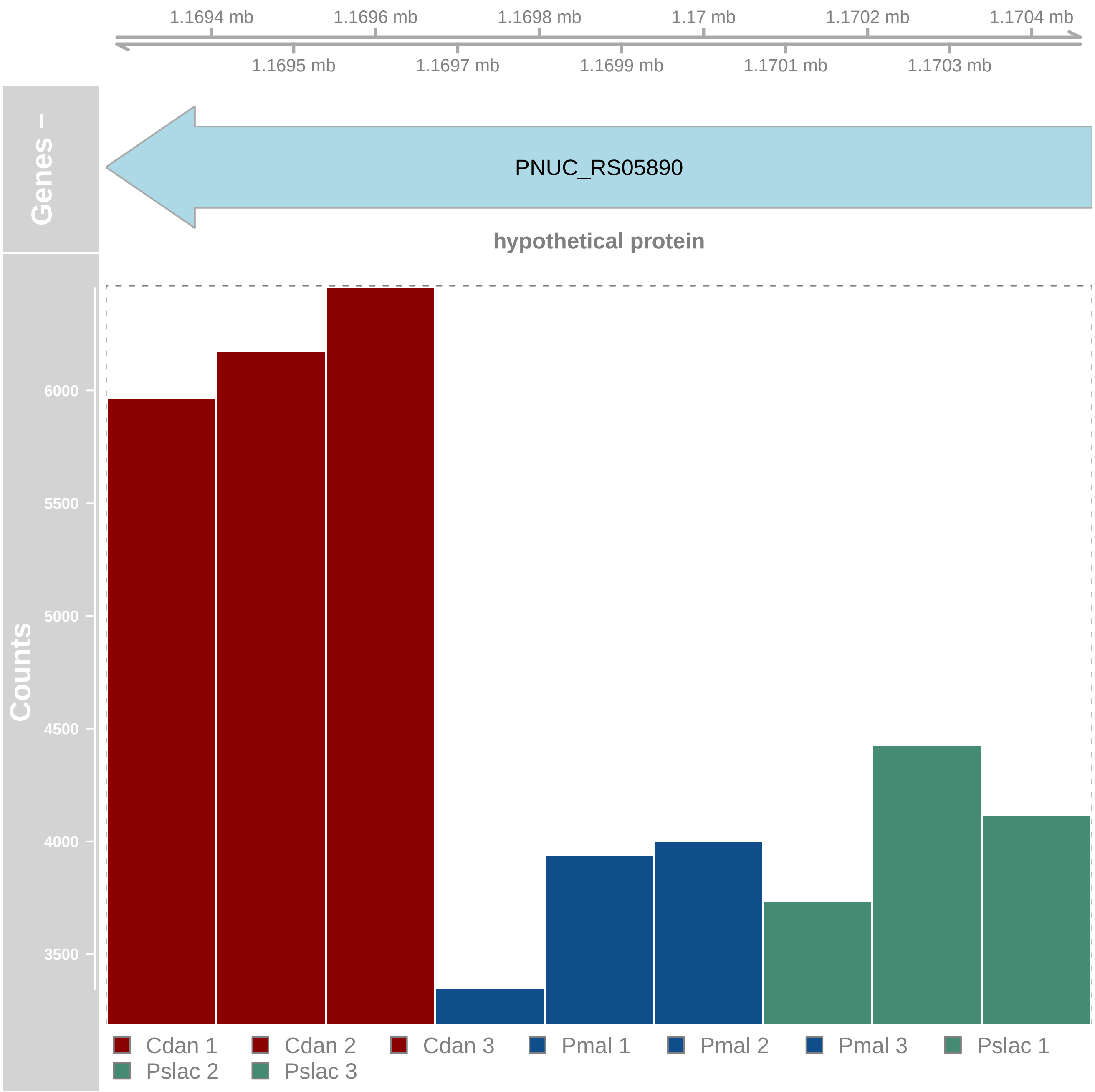

1.1694 mb
1.1695 mb
1.1696 mb
1.1697 mb
1.1698 mb
1.1699 mb
1.17 mb
1.1701 mb
1.1702 mb
1.1703 mb
1.1704 mb
Genes –
PNUC_RS05890
hypothetical protein
Counts
6000
5500
5000
4500
4000
3500
Cdan 1
Cdan 2
Cdan 3
Pmal 1
Pmal 2
Pmal 3
Pslac 1
Pslac 2
Pslac 3

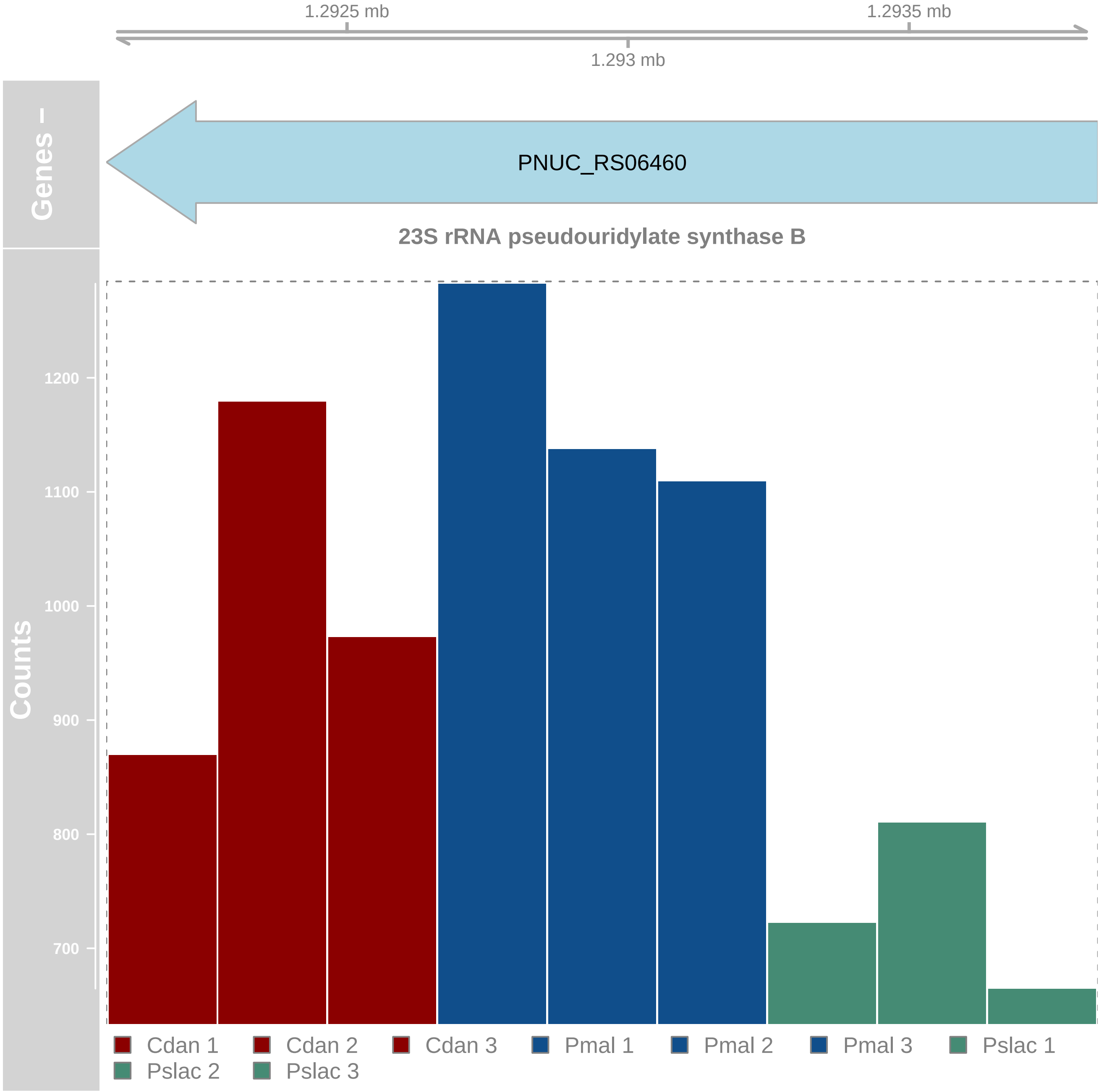
1.2925 mb
1.2935 mb
1.293 mb
Genes –
PNUC_RS06460
23S rRNA pseudouridylate synthase B
Counts
1200
1100
1000
900
800
700
Cdan 1
Cdan 2
Cdan 3
Pmal 1
Pmal 2
Pmal 3
Pslac 1
Pslac 2
Pslac 3

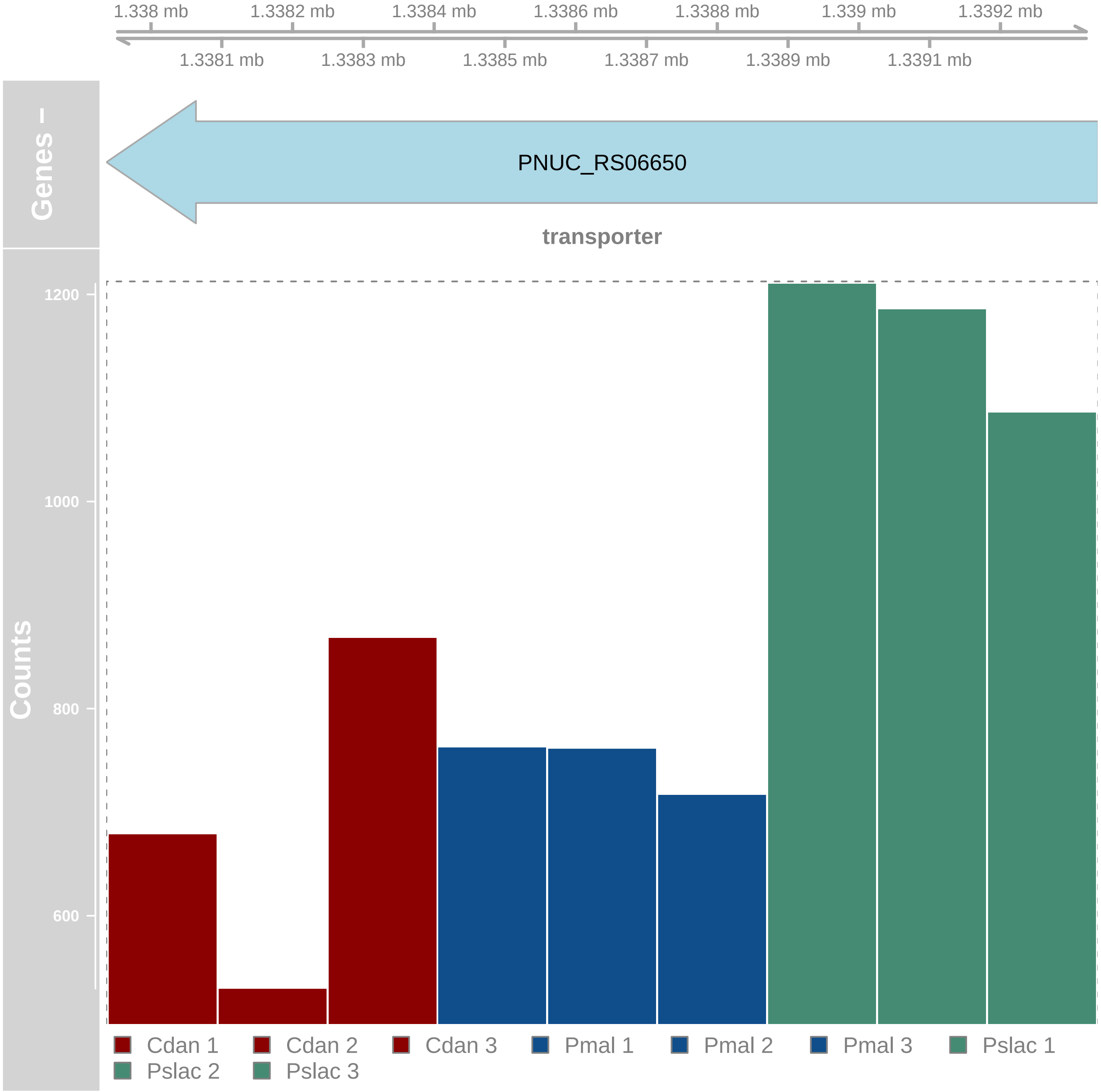

1.338 mb
1.3382 mb
1.3384 mb
1.3386 mb
1.3388 mb
1.339 mb
1.3392 mb
1.3381 mb
1.3383 mb
1.3385 mb
1.3387 mb
1.3389 mb
1.3391 mb
Genes –
PNUC_RS06650
transporter
Counts
1200
1000
800
600
Cdan 1
Cdan 2
Cdan 3
Pmal 1
Pmal 2
Pmal 3
Pslac 1
Pslac 2
Pslac 3

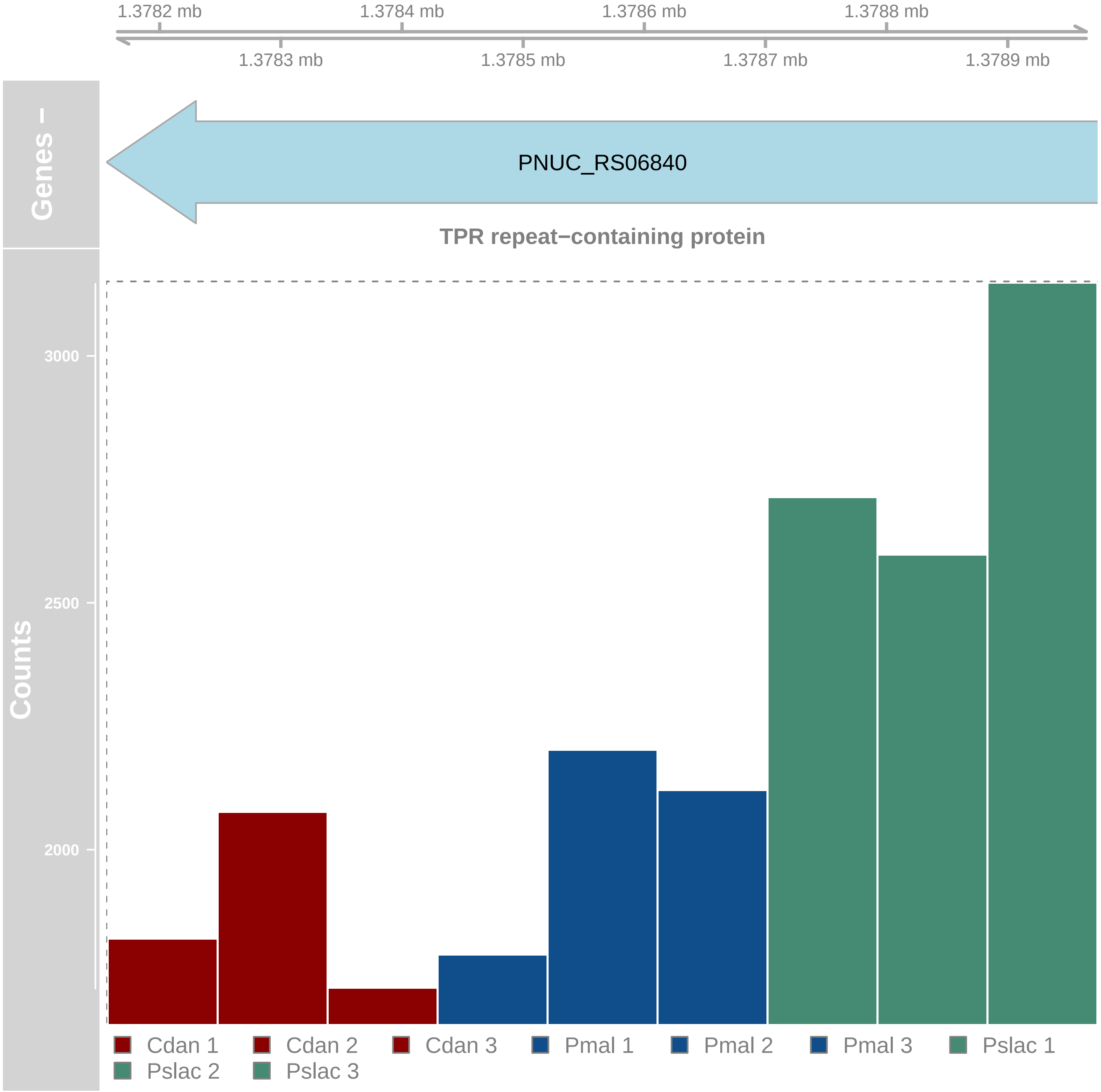
1.3782 mb
1.3783 mb
1.3784 mb
1.3785 mb
1.3786 mb
1.3787 mb
1.3788 mb
1.3789 mb
Genes −
PNUC_RS06840
TPR repeat−containing protein
Counts
3000
2500
2000
Cdan 1
Cdan 2
Cdan 3
Pmal 1
Pmal 2
Pmal 3
Pslac 1
Pslac 2
Pslac 3

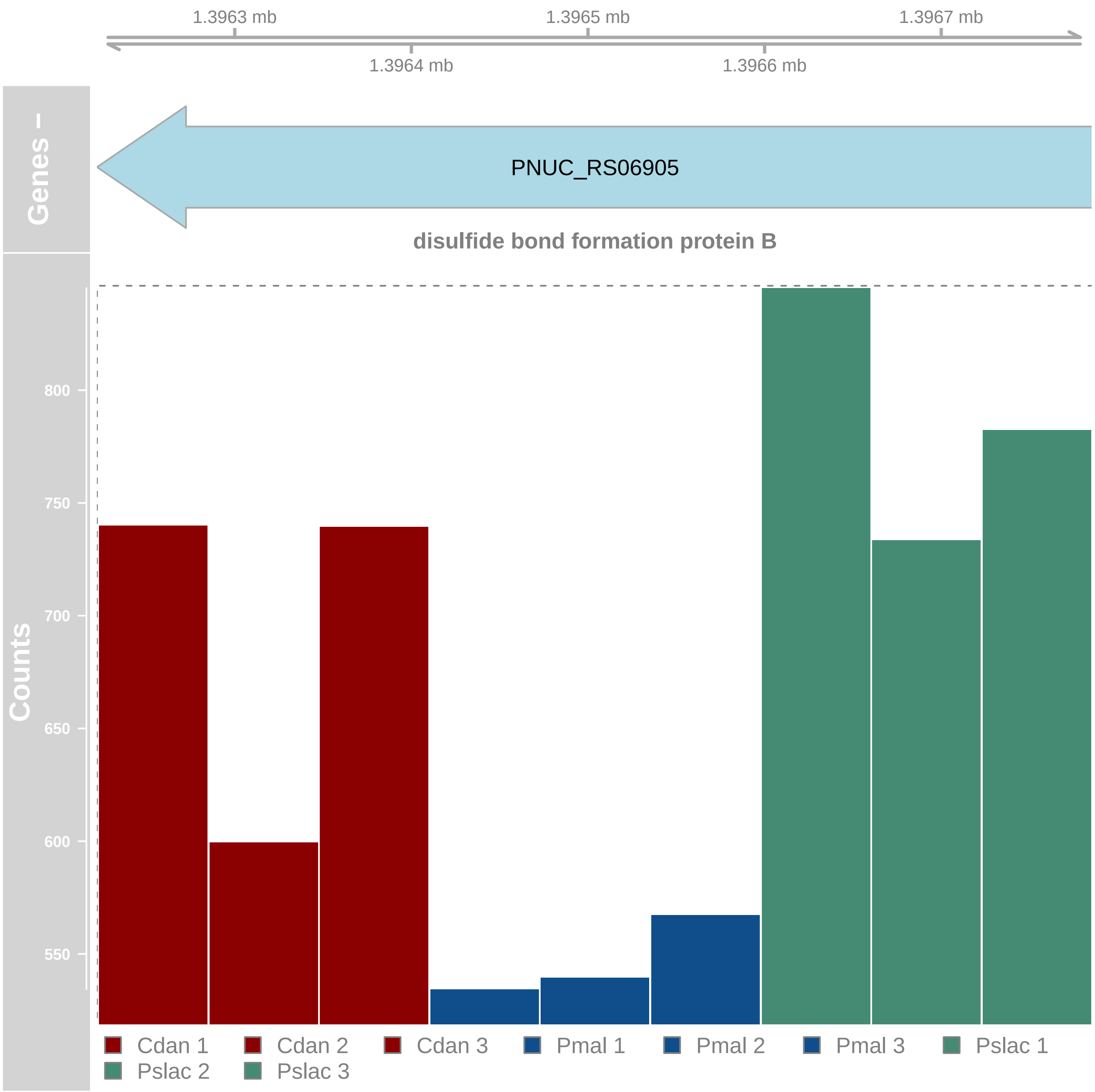
1.3963 mb
1.3965 mb
1.3967 mb
1.3964 mb
1.3966 mb
Genes –
PNUC_RS06905
disulfide bond formation protein B
Counts
800
750
700
650
600
550
Cdan 1
Cdan 2
Cdan 3
Pmal 1
Pmal 2
Pmal 3
Pslac 1
Pslac 2
Pslac 3

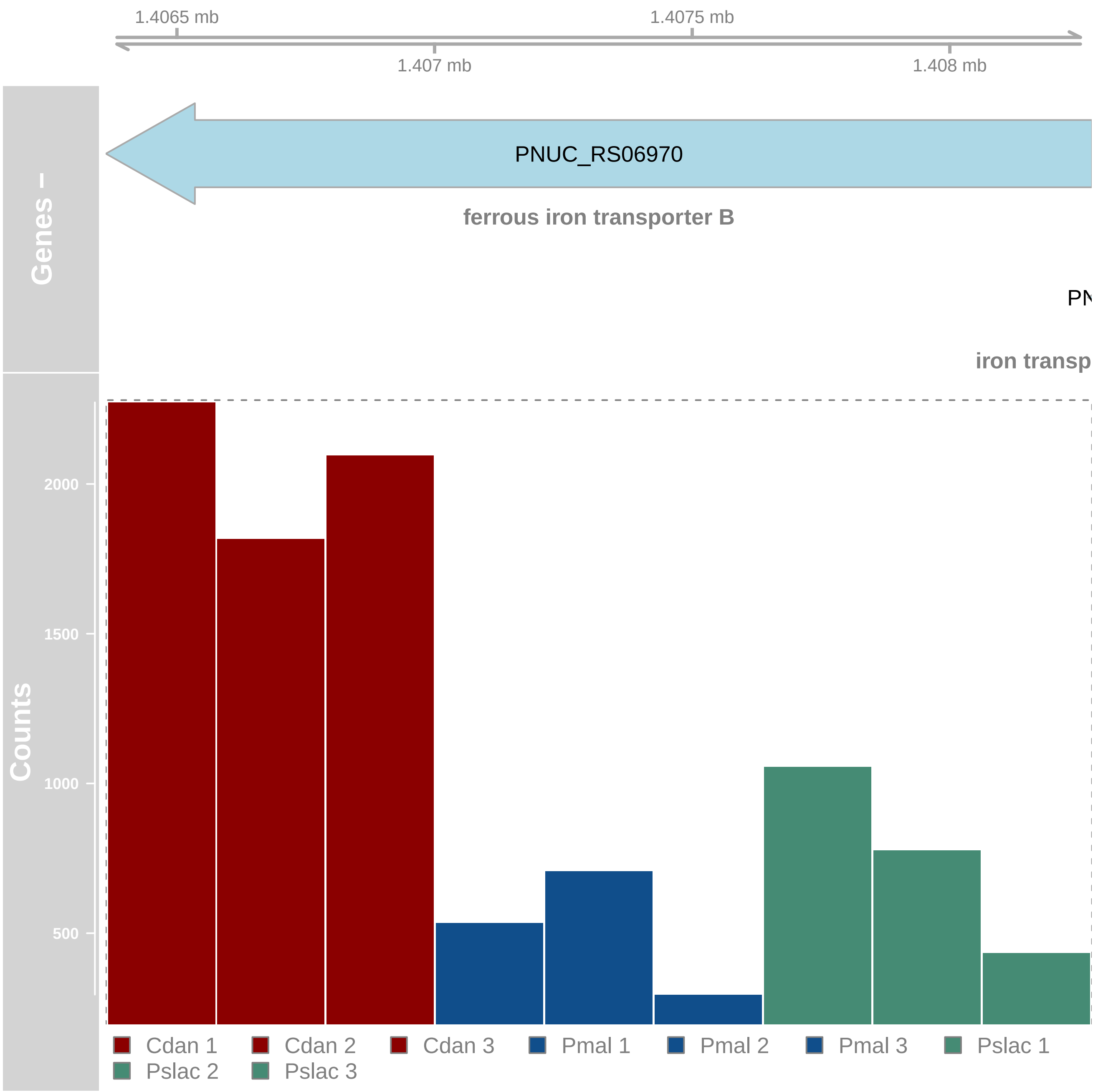

1.4065 mb
1.4075 mb
1.407 mb
1.408 mb
Genes –
PNUC_RS06970
ferrous iron transporter B
PN
iron transp
Counts
2000
1500
1000
500
Cdan 1
Cdan 2
Cdan 3
Pmal 1
Pmal 2
Pmal 3
Pslac 1
Pslac 2
Pslac 3

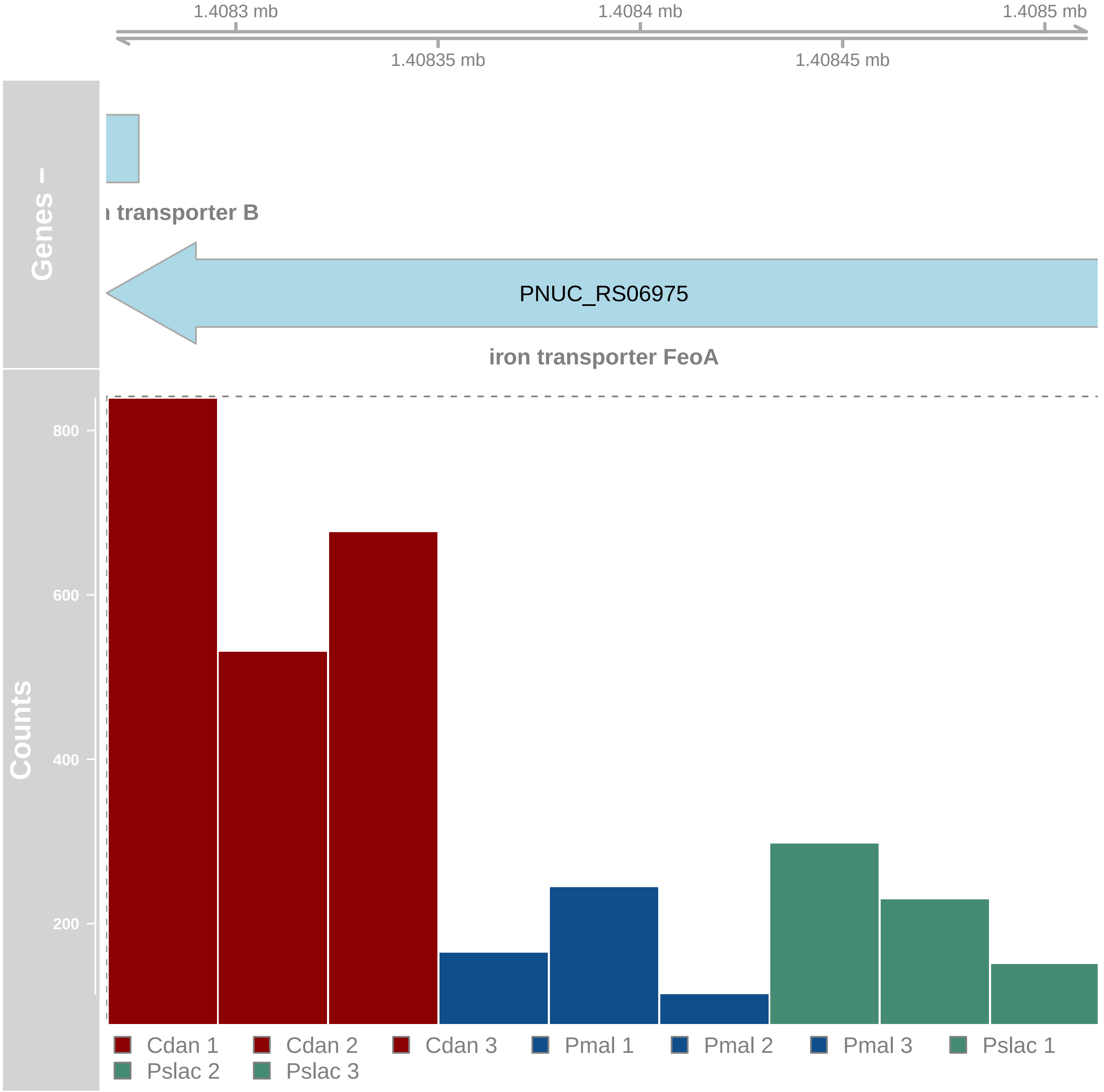

1.4083 mb
1.4084 mb
1.4085 mb
1.40835 mb
1.40845 mb
Genes –
n transporter B
PNUC_RS06975
iron transporter FeoA
Counts
800
600
400
200
Cdan 1
Cdan 2
Cdan 3
Pmal 1
Pmal 2
Pmal 3
Pslac 1
Pslac 2
Pslac 3

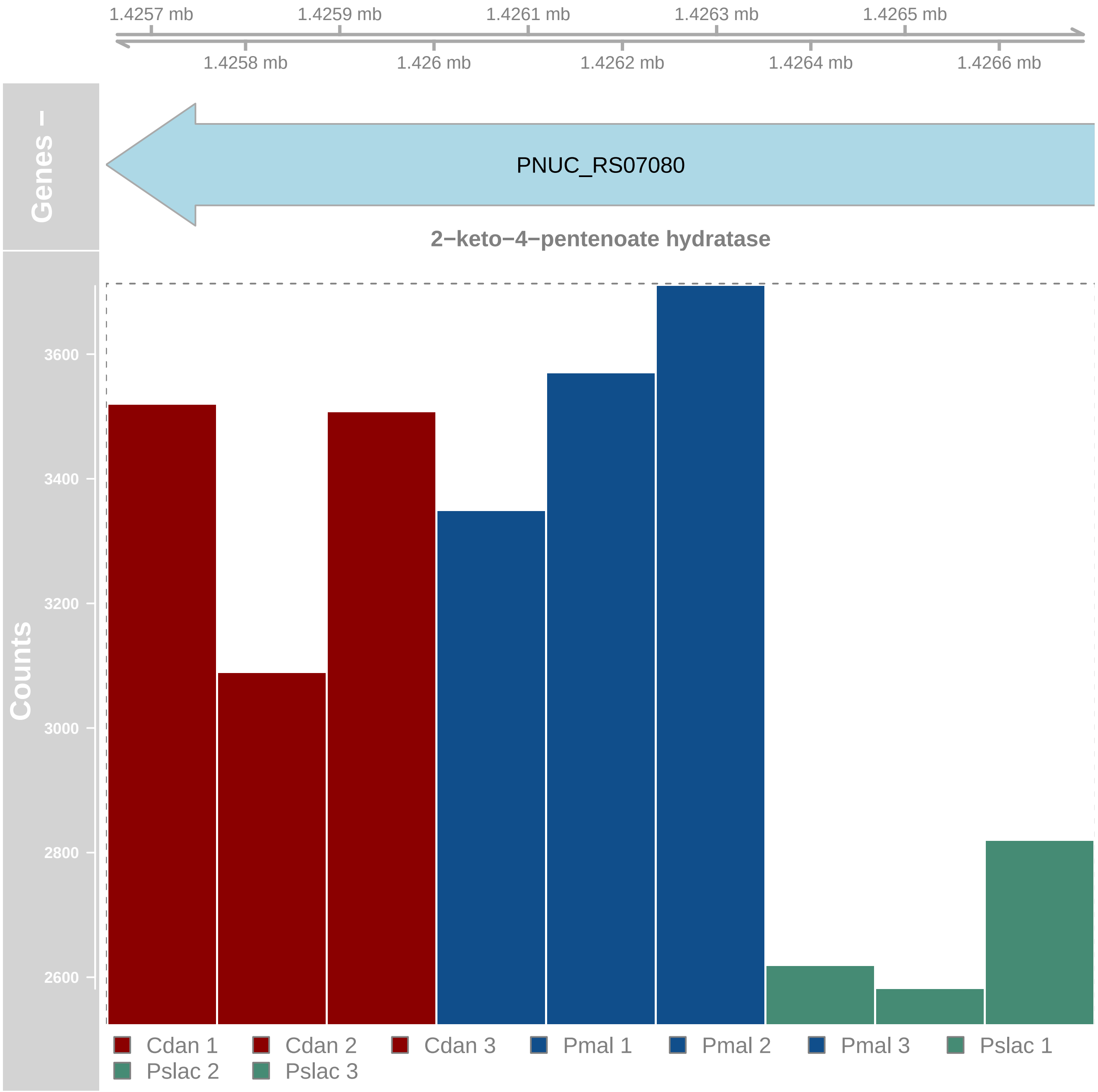

1.4257 mb
1.4258 mb
1.4259 mb
1.426 mb
1.4261 mb
1.4262 mb
1.4263 mb
1.4264 mb
1.4265 mb
1.4266 mb
Genes −
PNUC_RS07080
2−keto−4−pentenoate hydratase
Counts
3600
3400
3200
3000
2800
2600
Cdan 1
Cdan 2
Cdan 3
Pmal 1
Pmal 2
Pmal 3
Pslac 1
Pslac 2
Pslac 3

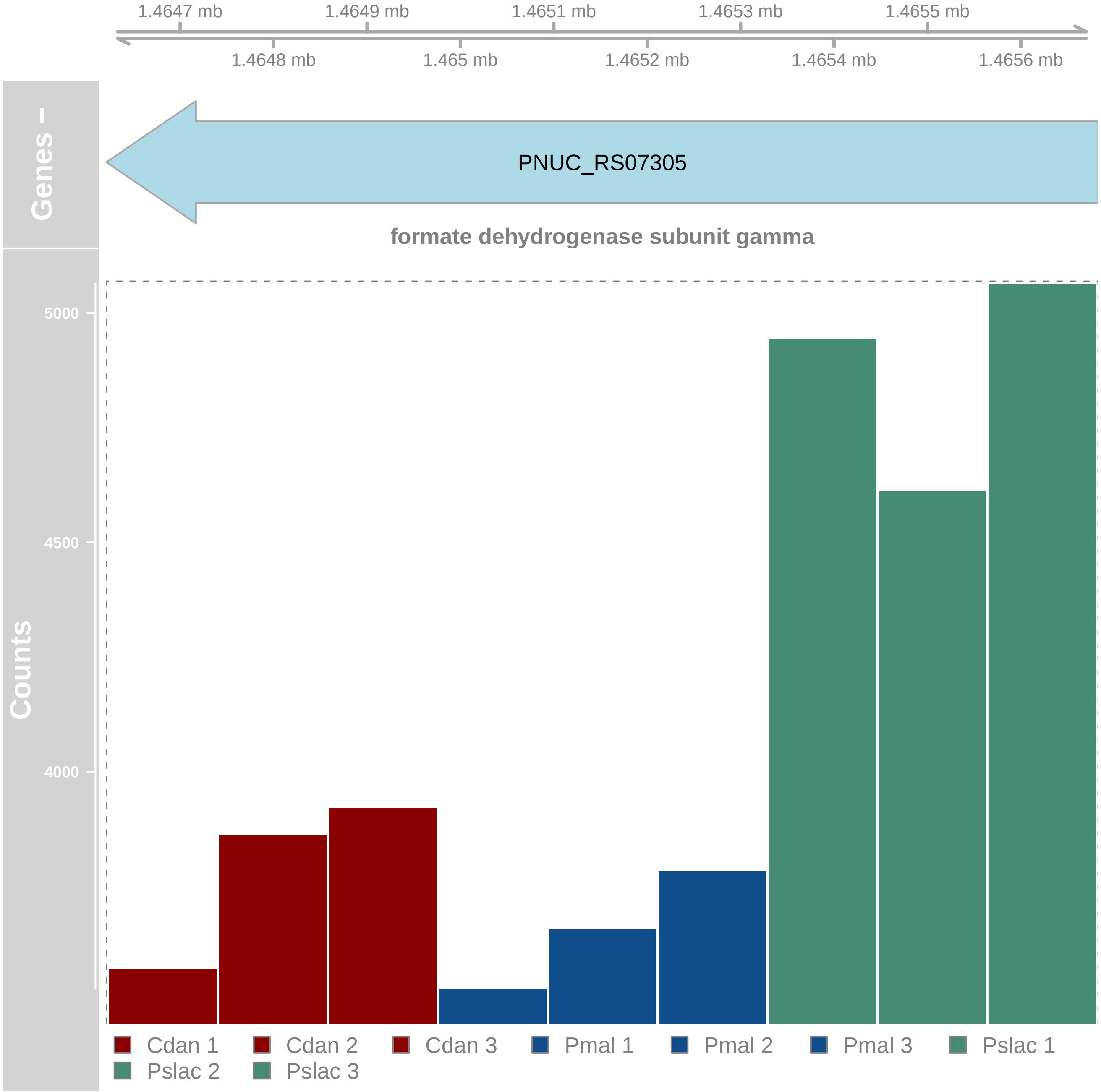
1.4647 mb
1.4649 mb
1.4651 mb
1.4653 mb
1.4655 mb
1.4648 mb
1.465 mb
1.4652 mb
1.4654 mb
1.4656 mb
Genes –
PNUC_RS07305
formate dehydrogenase subunit gamma
Counts
5000
4500
4000
Cdan 1
Cdan 2
Cdan 3
Pmal 1
Pmal 2
Pmal 3
Pslac 1
Pslac 2
Pslac 3

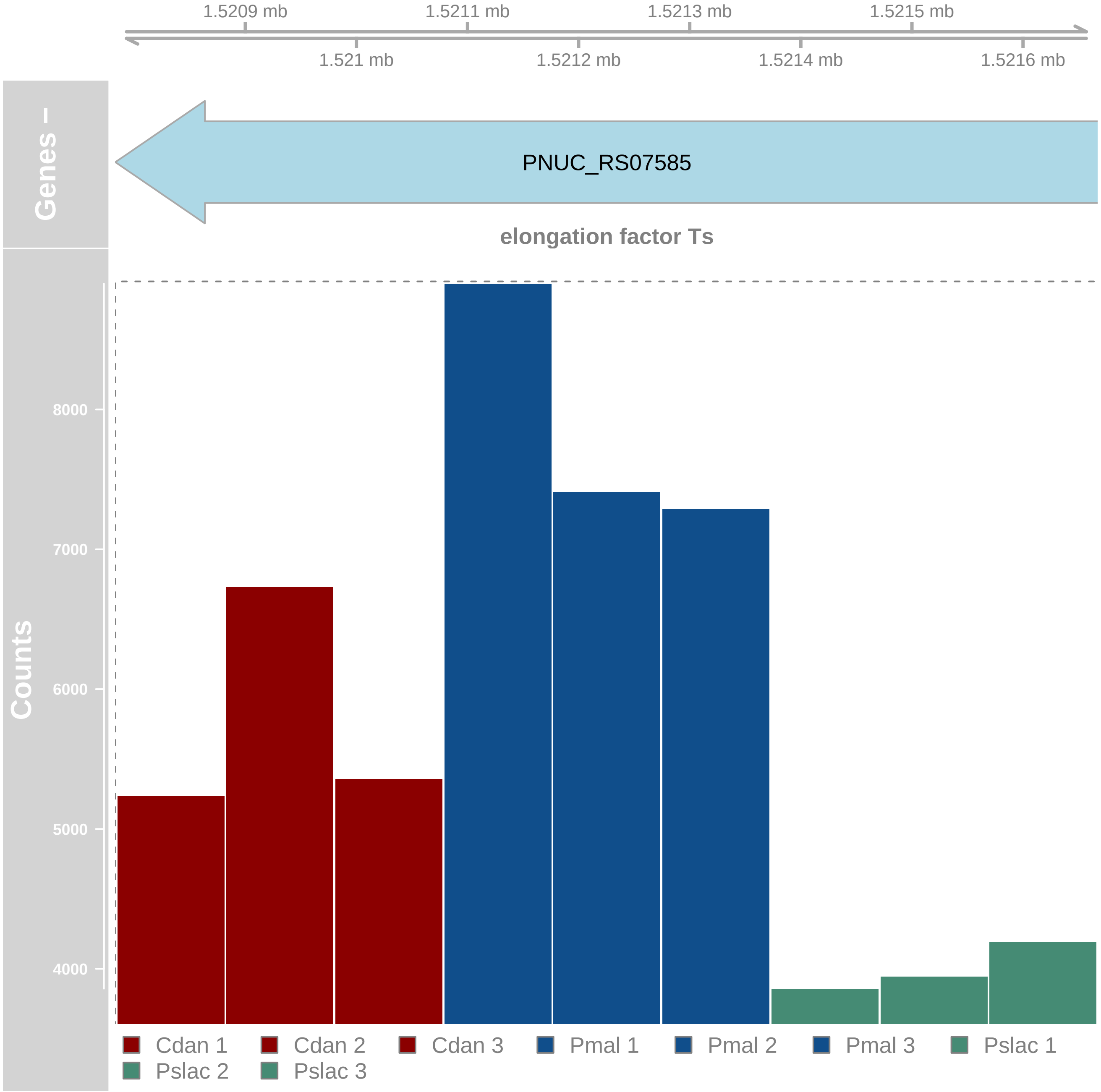

1.5209 mb
1.5211 mb
1.5213 mb
1.5215 mb
1.521 mb
1.5212 mb
1.5214 mb
1.5216 mb
Genes –
PNUC_RS07585
elongation factor Ts
Counts
8000
7000
6000
5000
4000
Cdan 1
Cdan 2
Cdan 3
Pmal 1
Pmal 2
Pmal 3
Pslac 1
Pslac 2
Pslac 3

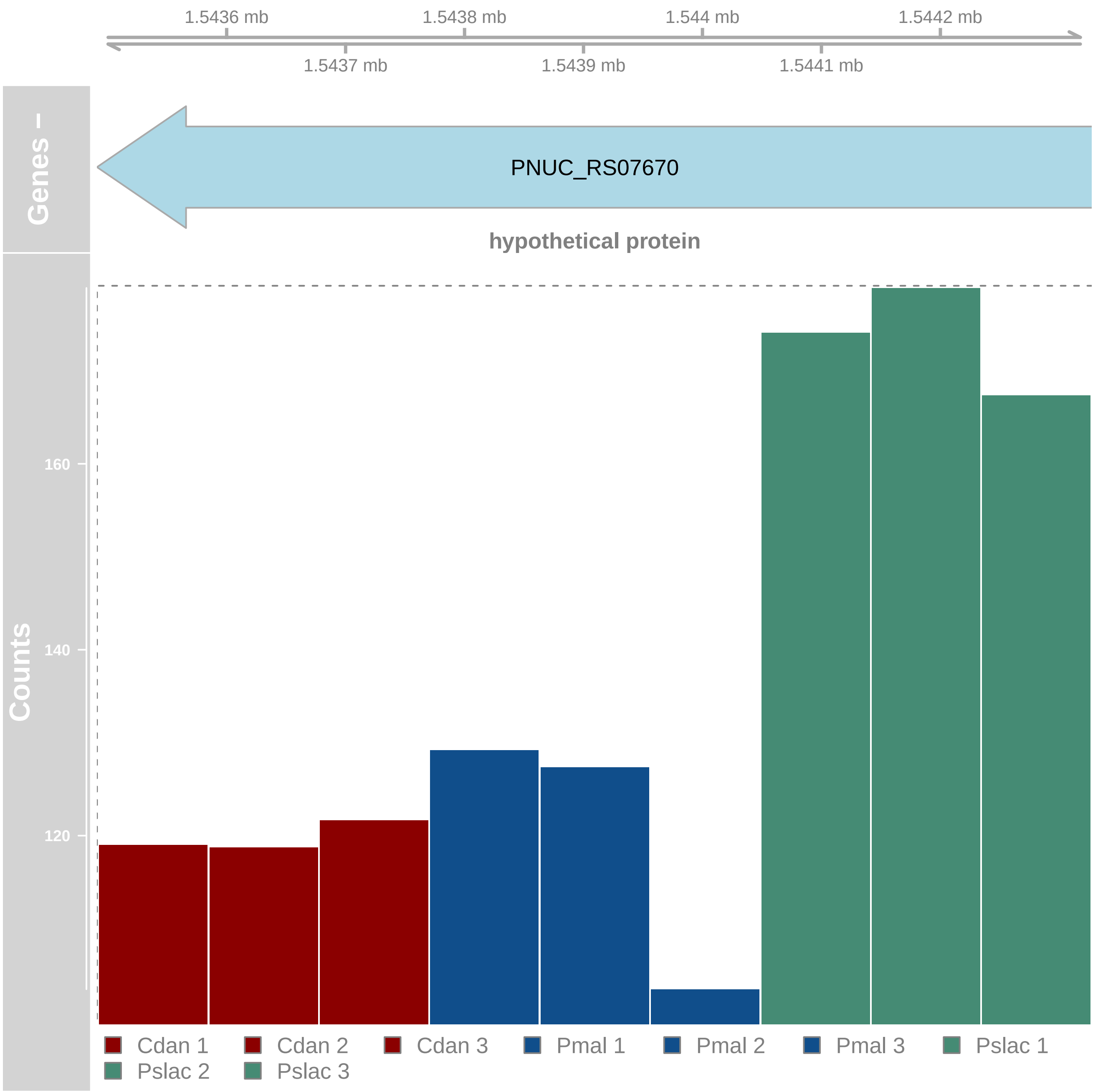
1.5436 mb
1.5437 mb
1.5438 mb
1.5439 mb
1.544 mb
1.5441 mb
1.5442 mb
Genes −
PNUC_RS07670
hypothetical protein
Counts
160
140
120
Cdan 1
Cdan 2
Cdan 3
Pmal 1
Pmal 2
Pmal 3
Pslac 1
Pslac 2
Pslac 3

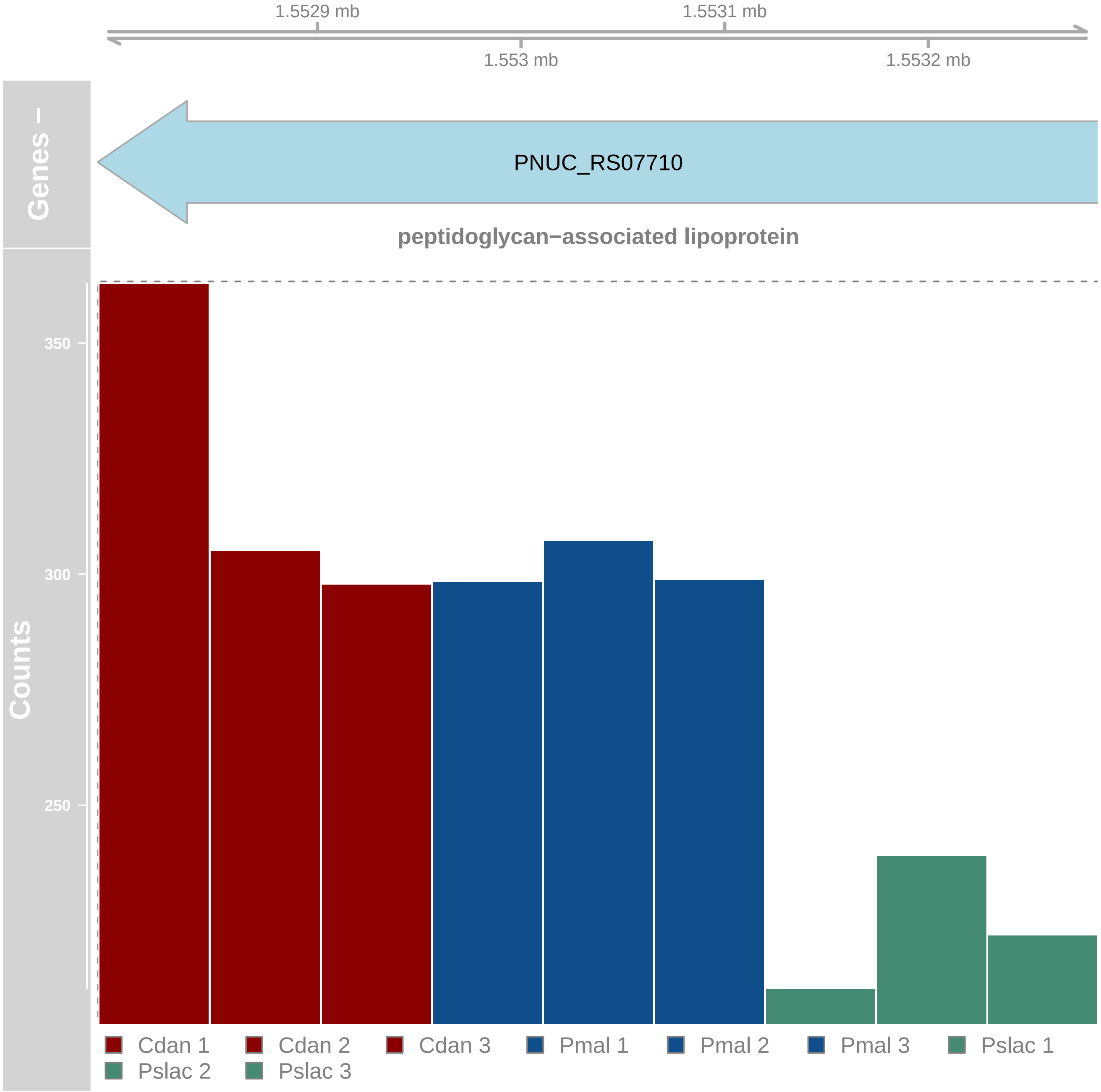
1.5529 mb
1.5531 mb
1.553 mb
1.5532 mb
Genes −
PNUC_RS07710
peptidoglycan−associated lipoprotein
Counts
350
300
250
Cdan 1
Cdan 2
Cdan 3
Pmal 1
Pmal 2
Pmal 3
Pslac 1
Pslac 2
Pslac 3

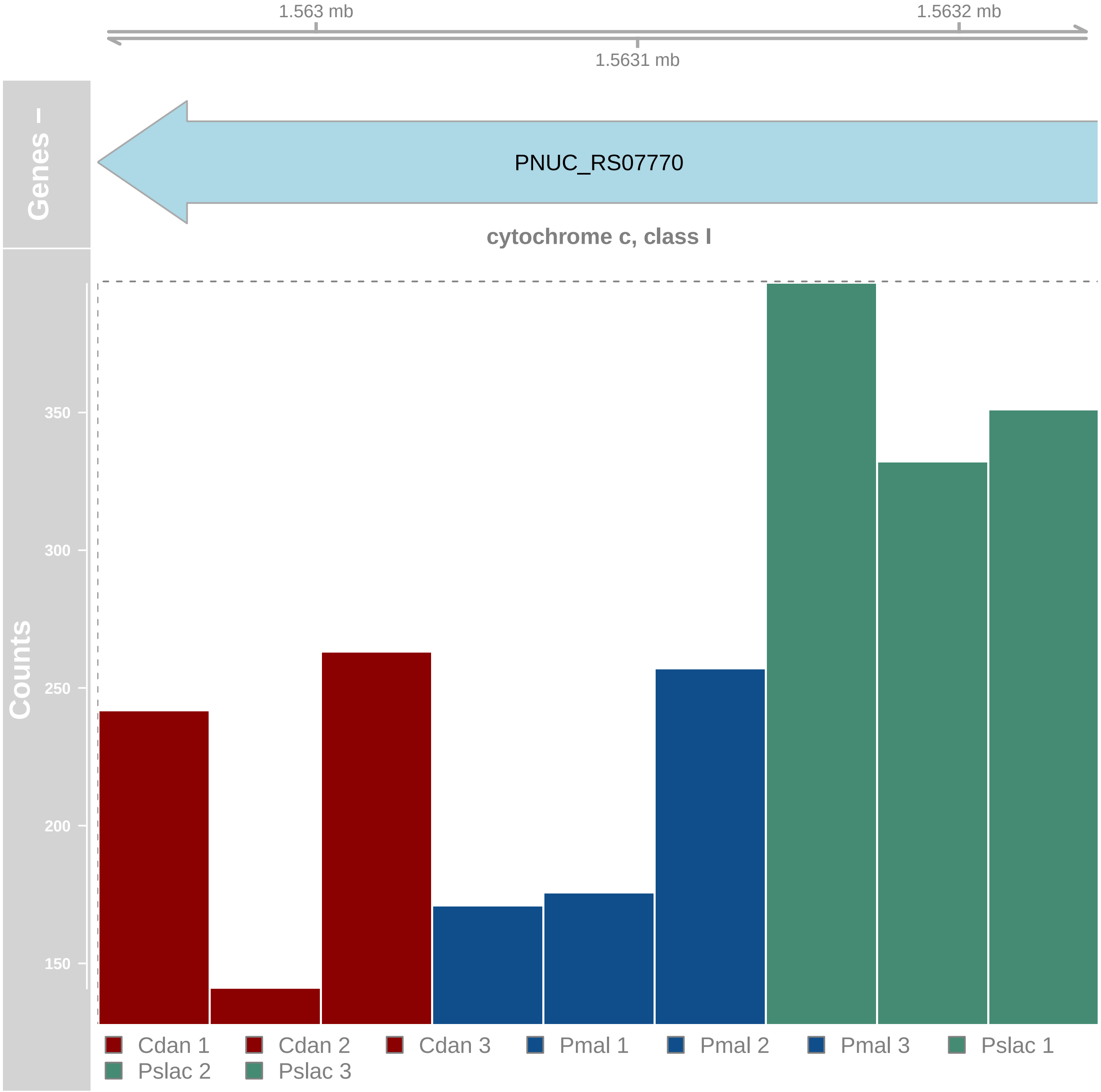

1.563 mb
1.5631 mb
1.5632 mb
Genes –
PNUC_RS07770
cytochrome c, class I
Counts
350
300
250
200
150
Cdan 1
Cdan 2
Cdan 3
Pmal 1
Pmal 2
Pmal 3
Pslac 1
Pslac 2
Pslac 3

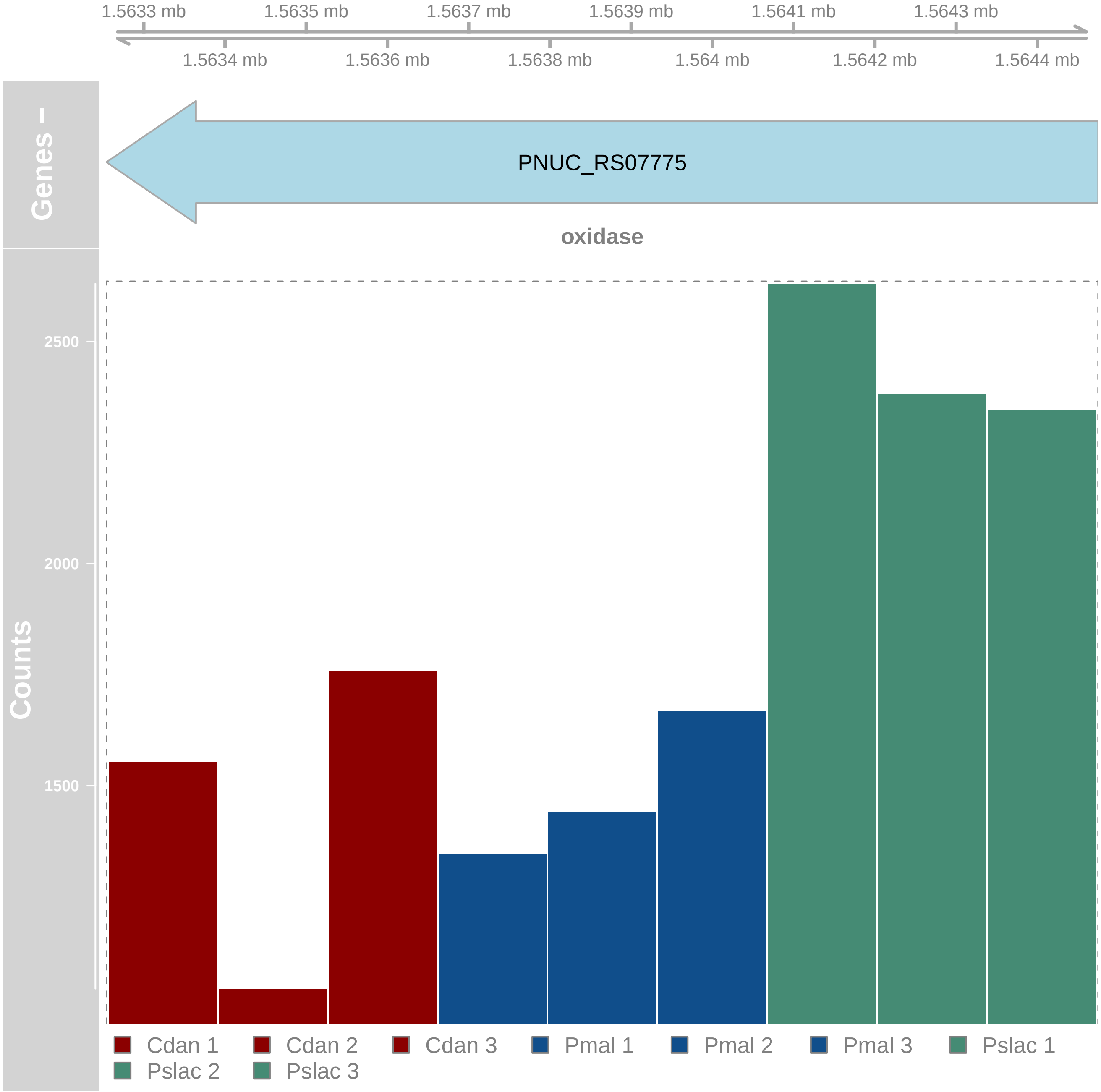

1.5633 mb
1.5635 mb
1.5637 mb
1.5639 mb
1.5641 mb
1.5643 mb
1.5634 mb
1.5636 mb
1.5638 mb
1.564 mb
1.5642 mb
1.5644 mb
Genes –
PNUC_RS07775
oxidase
Counts
2500
2000
1500
Cdan 1
Cdan 2
Cdan 3
Pmal 1
Pmal 2
Pmal 3
Pslac 1
Pslac 2
Pslac 3

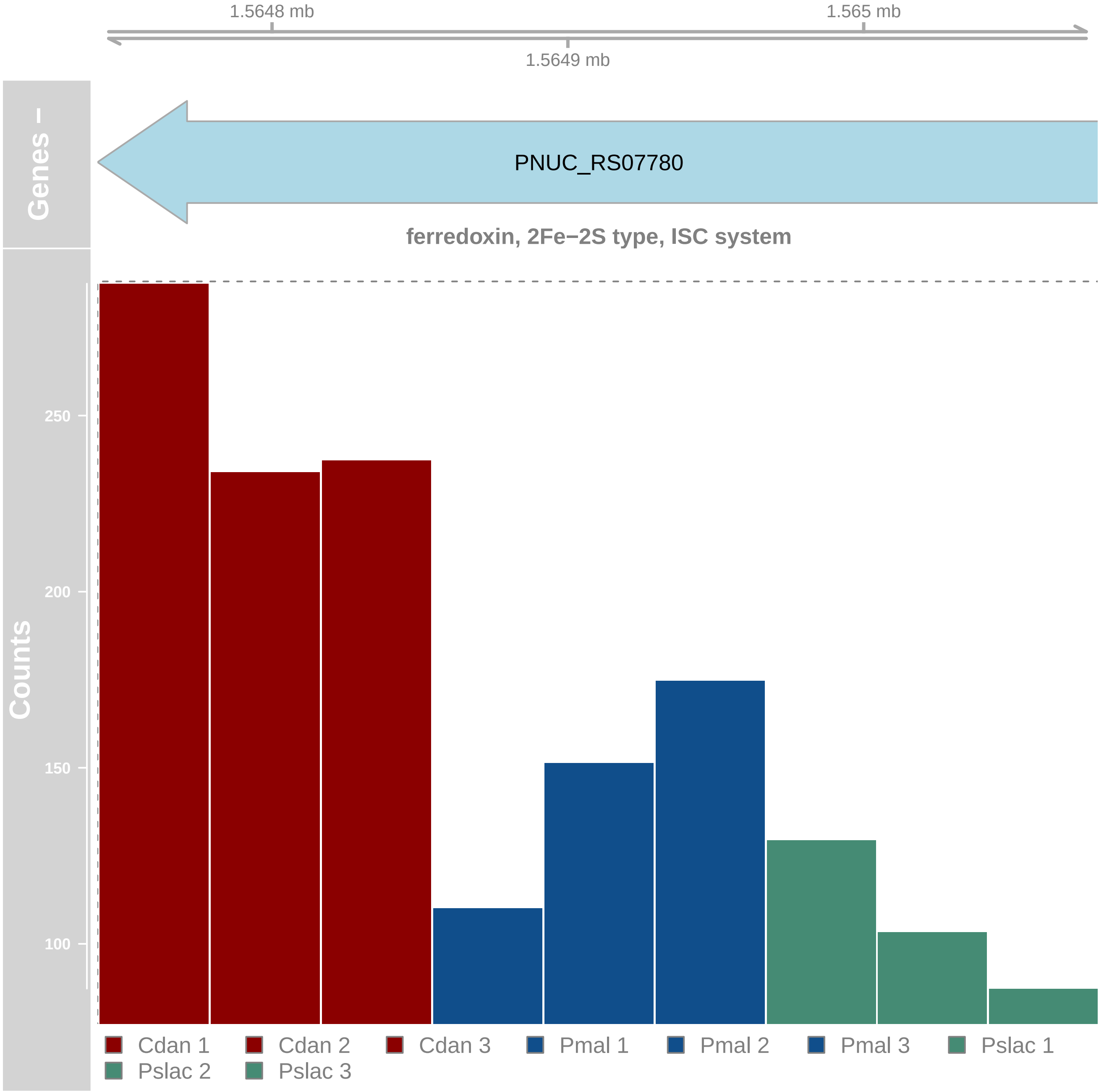

1.5648 mb
1.565 mb
1.5649 mb
Genes −
PNUC_RS07780
ferredoxin, 2Fe−2S type, ISC system
Counts
250
200
150
100
Cdan 1
Cdan 2
Cdan 3
Pmal 1
Pmal 2
Pmal 3
Pslac 1
Pslac 2
Pslac 3

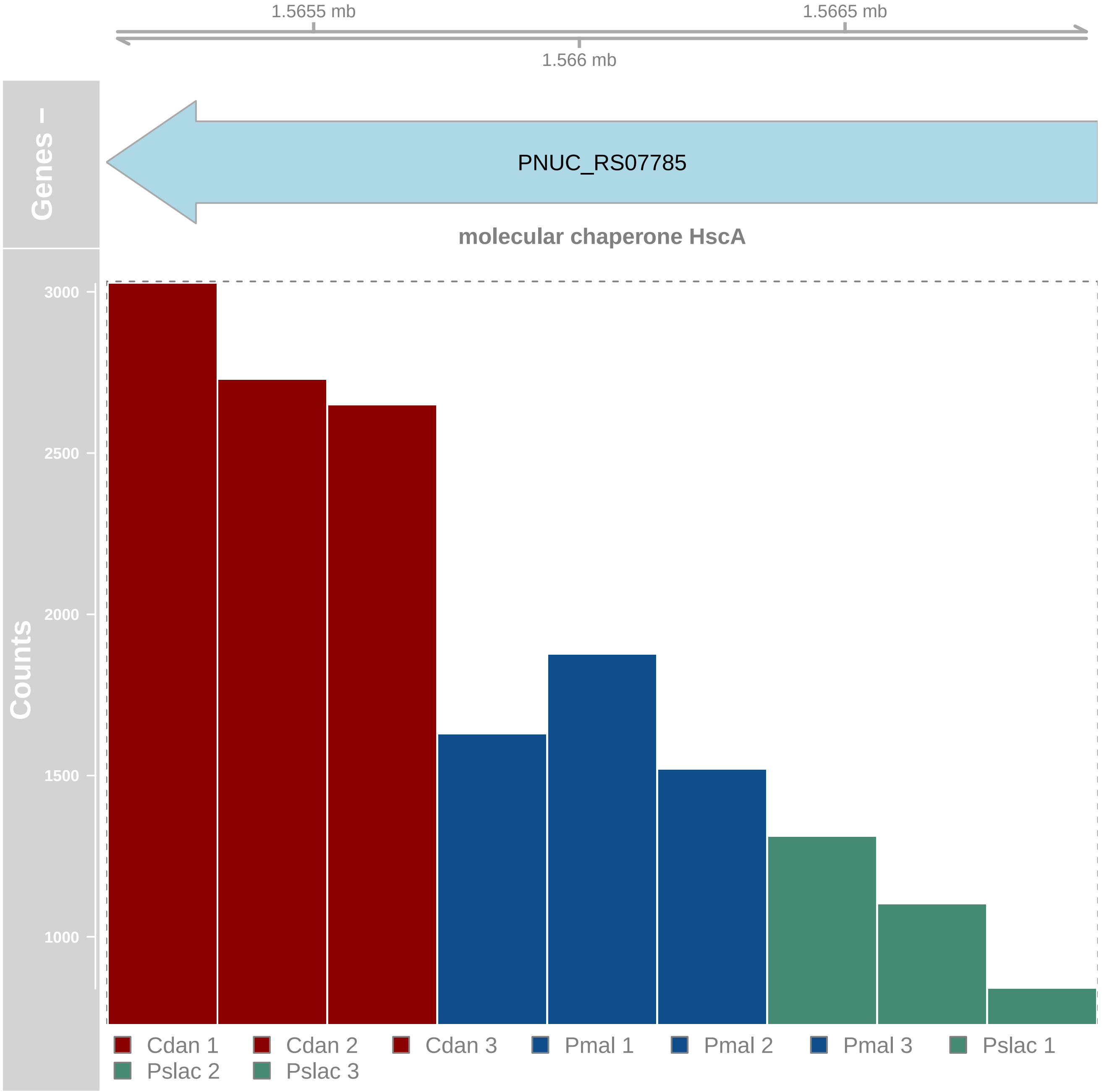

1.5655 mb
1.5665 mb
1.566 mb
Genes –
PNUC_RS07785
molecular chaperone HscA
Counts
3000
2500
2000
1500
1000
Cdan 1
Cdan 2
Cdan 3
Pmal 1
Pmal 2
Pmal 3
Pslac 1
Pslac 2
Pslac 3

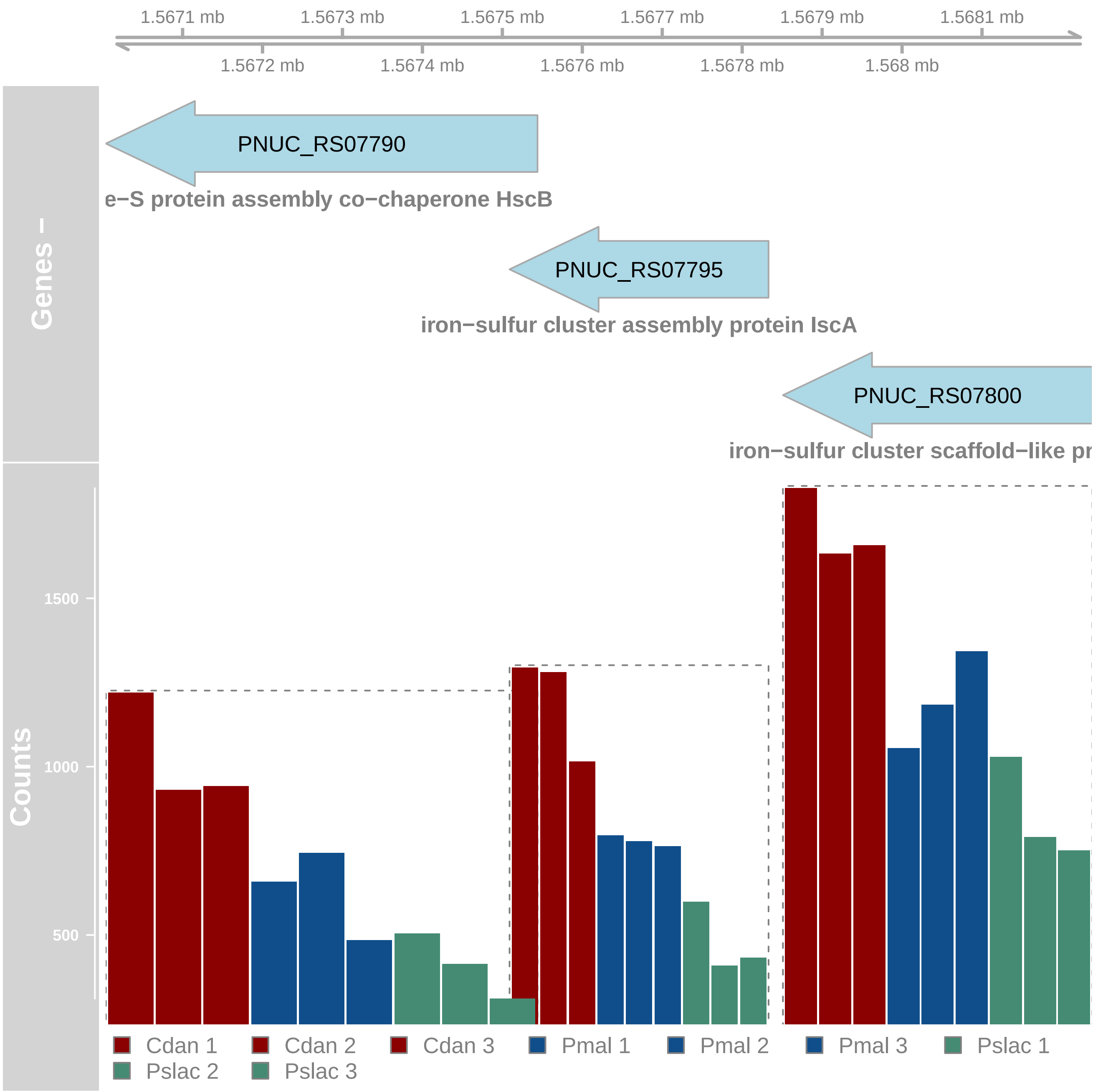

1.5671 mb
1.5673 mb
1.5675 mb
1.5677 mb
1.5679 mb
1.5681 mb
1.5672 mb
1.5674 mb
1.5676 mb
1.5678 mb
1.568 mb
Genes −
PNUC_RS07790
e−S protein assembly co−chaperone HscB
PNUC_RS07795
iron−sulfur cluster assembly protein IscA
PNUC_RS07800
iron−sulfur cluster scaffold−like pr
Counts
1500
1000
500
Cdan 1
Cdan 2
Cdan 3
Pmal 1
Pmal 2
Pmal 3
Pslac 1
Pslac 2
Pslac 3

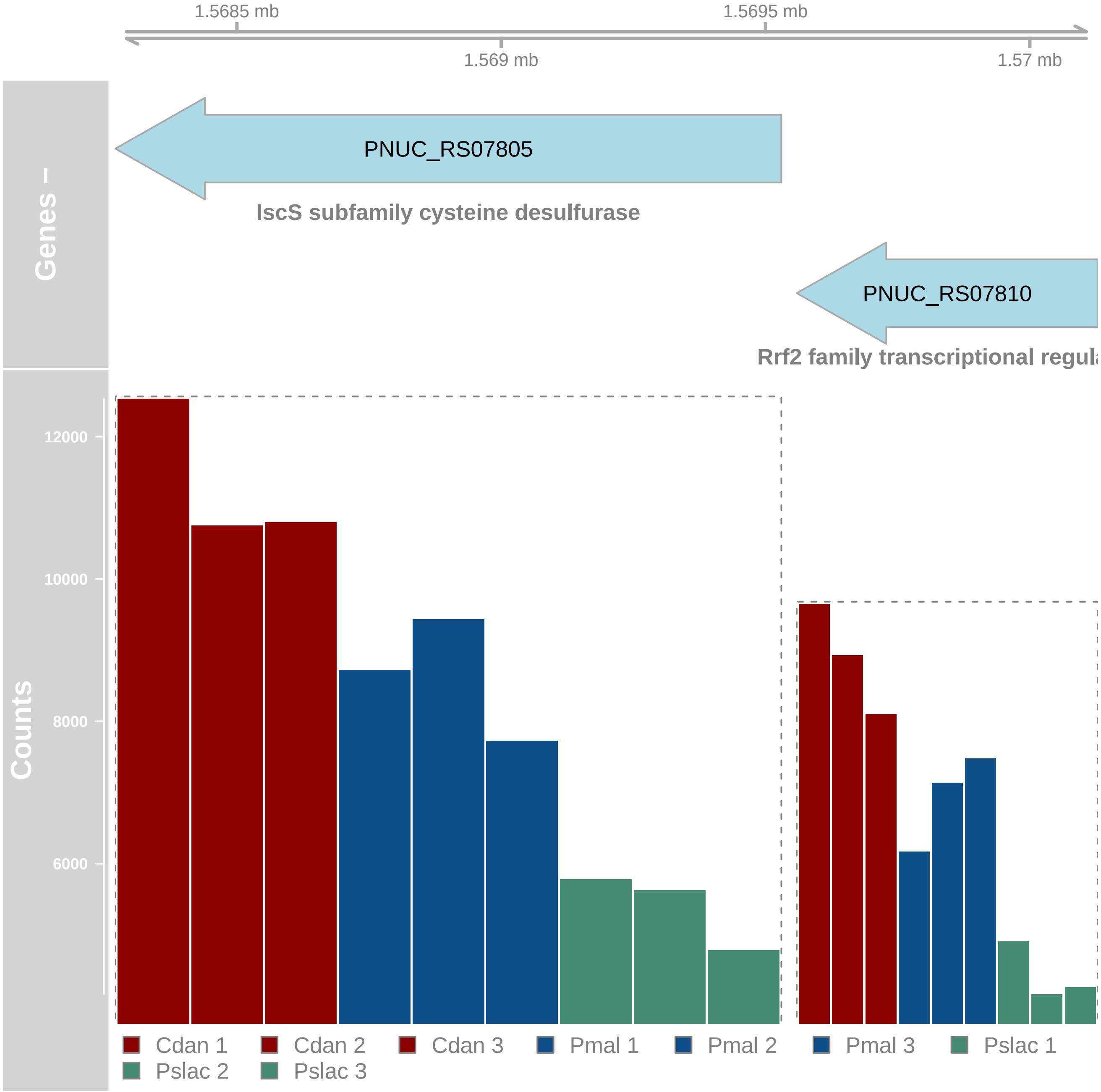

1.5685 mb
1.5695 mb
1.569 mb
1.57 mb
Genes –
PNUC_RS07805
IscS subfamily cysteine desulfurase
PNUC_RS07810
Rrf2 family transcriptional regula
Counts
12000
10000
8000
6000
Cdan 1
Cdan 2
Cdan 3
Pmal 1
Pmal 2
Pmal 3
Pslac 1
Pslac 2
Pslac 3

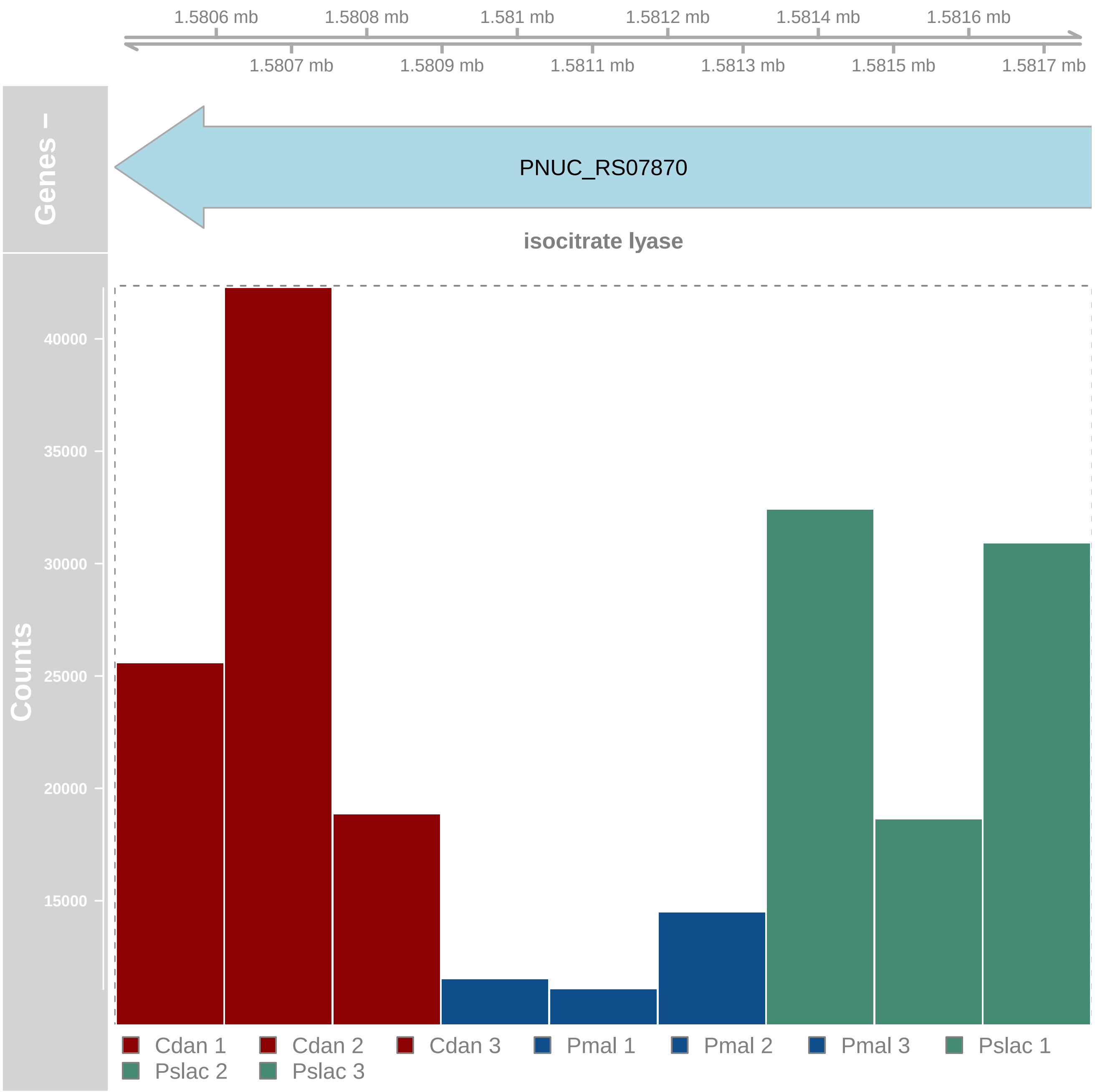
1.5806 mb
1.5807 mb
1.5808 mb
1.5809 mb
1.581 mb
1.5811 mb
1.5812 mb
1.5813 mb
1.5814 mb
1.5815 mb
1.5816 mb
1.5817 mb
Genes –
PNUC_RS07870
isocitrate lyase
Counts
40000
35000
30000
25000
20000
15000
Cdan 1
Cdan 2
Cdan 3
Pmal 1
Pmal 2
Pmal 3
Pslac 1
Pslac 2
Pslac 3

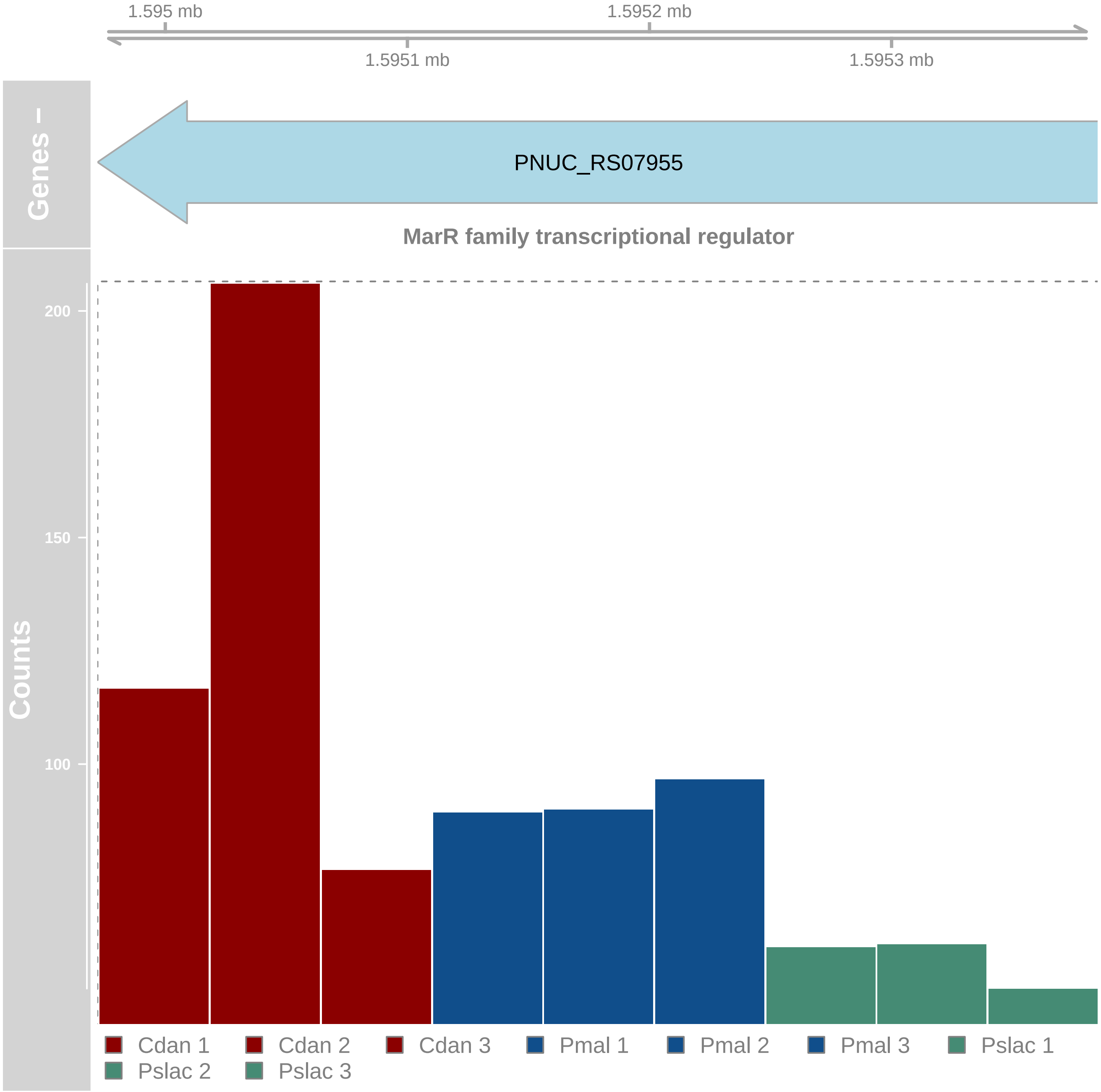

1.595 mb
1.5952 mb
1.5951 mb
1.5953 mb
Genes –
PNUC_RS07955
MarR family transcriptional regulator
Counts
200
150
100
Cdan 1
Cdan 2
Cdan 3
Pmal 1
Pmal 2
Pmal 3
Pslac 1
Pslac 2
Pslac 3

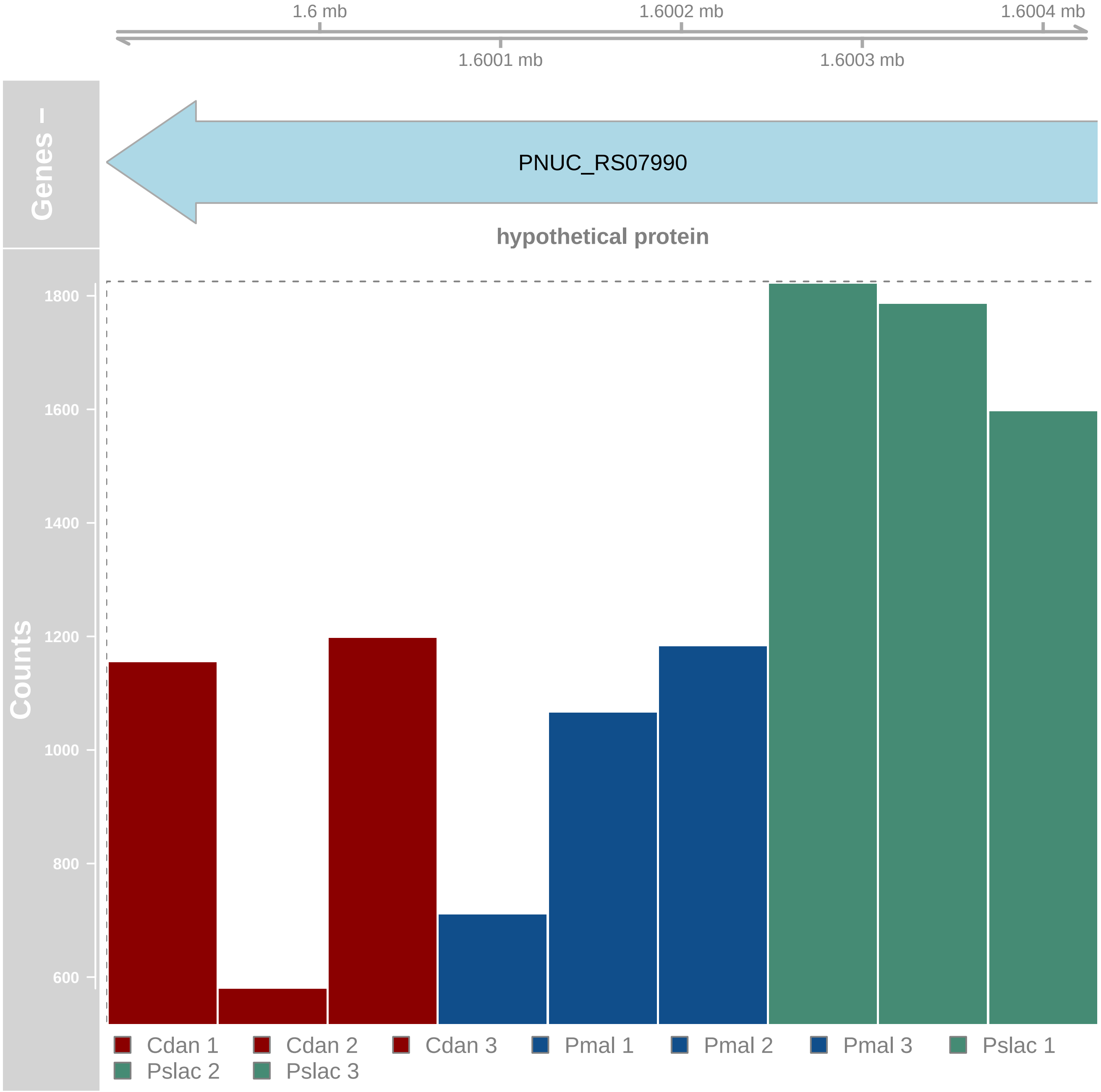
1.6 mb
1.6001 mb
1.6002 mb
1.6003 mb
1.6004 mb
Genes –
PNUC_RS07990
hypothetical protein
Counts
1800
1600
1400
1200
1000
800
600
Cdan 1
Cdan 2
Cdan 3
Pmal 1
Pmal 2
Pmal 3
Pslac 1
Pslac 2
Pslac 3

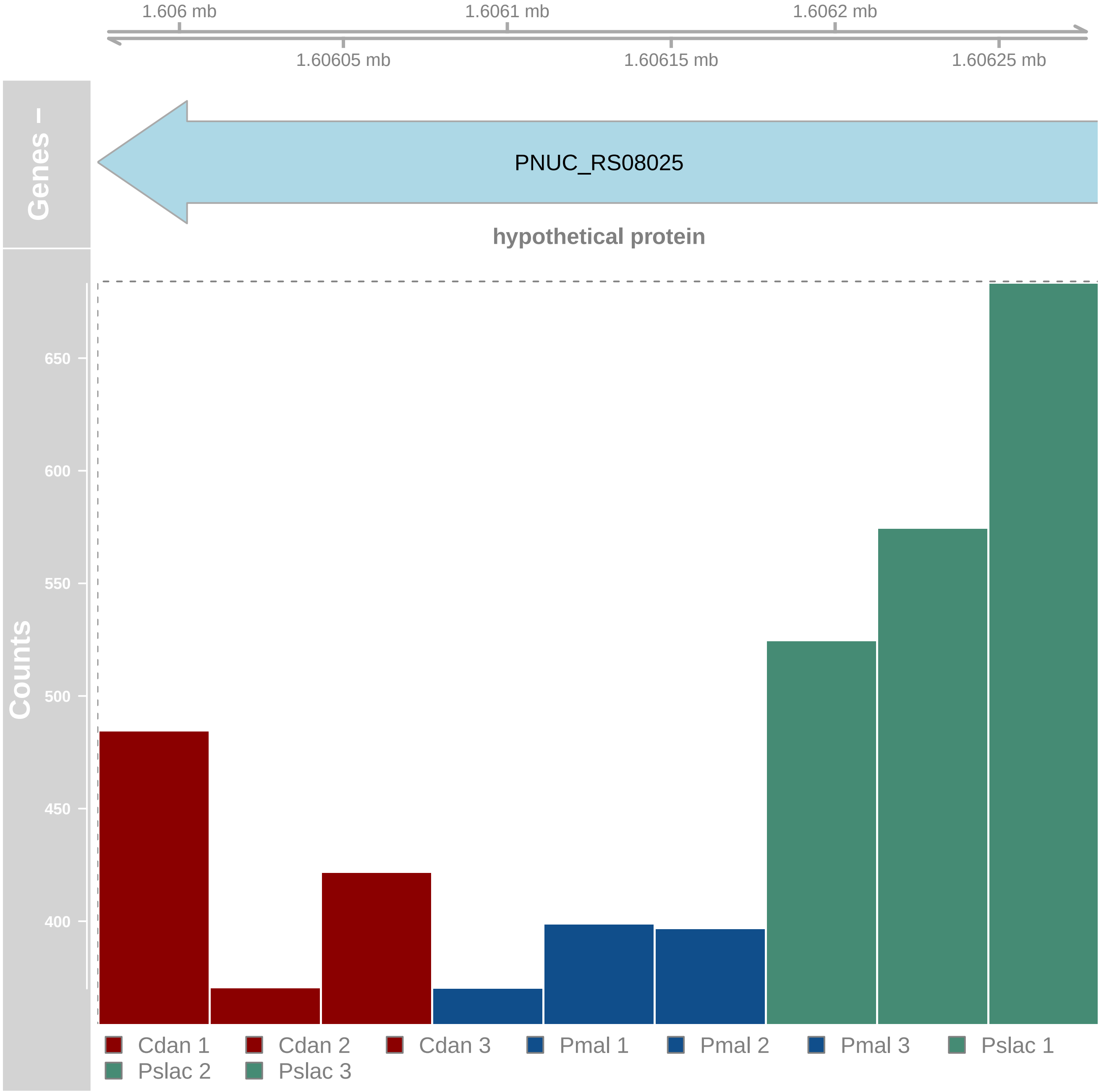
1.606 mb
1.6061 mb
1.6062 mb
1.60605 mb
1.60615 mb
1.60625 mb
Genes –
PNUC_RS08025
hypothetical protein
Counts
650
600
550
500
450
400
Cdan 1
Cdan 2
Cdan 3
Pmal 1
Pmal 2
Pmal 3
Pslac 1
Pslac 2
Pslac 3

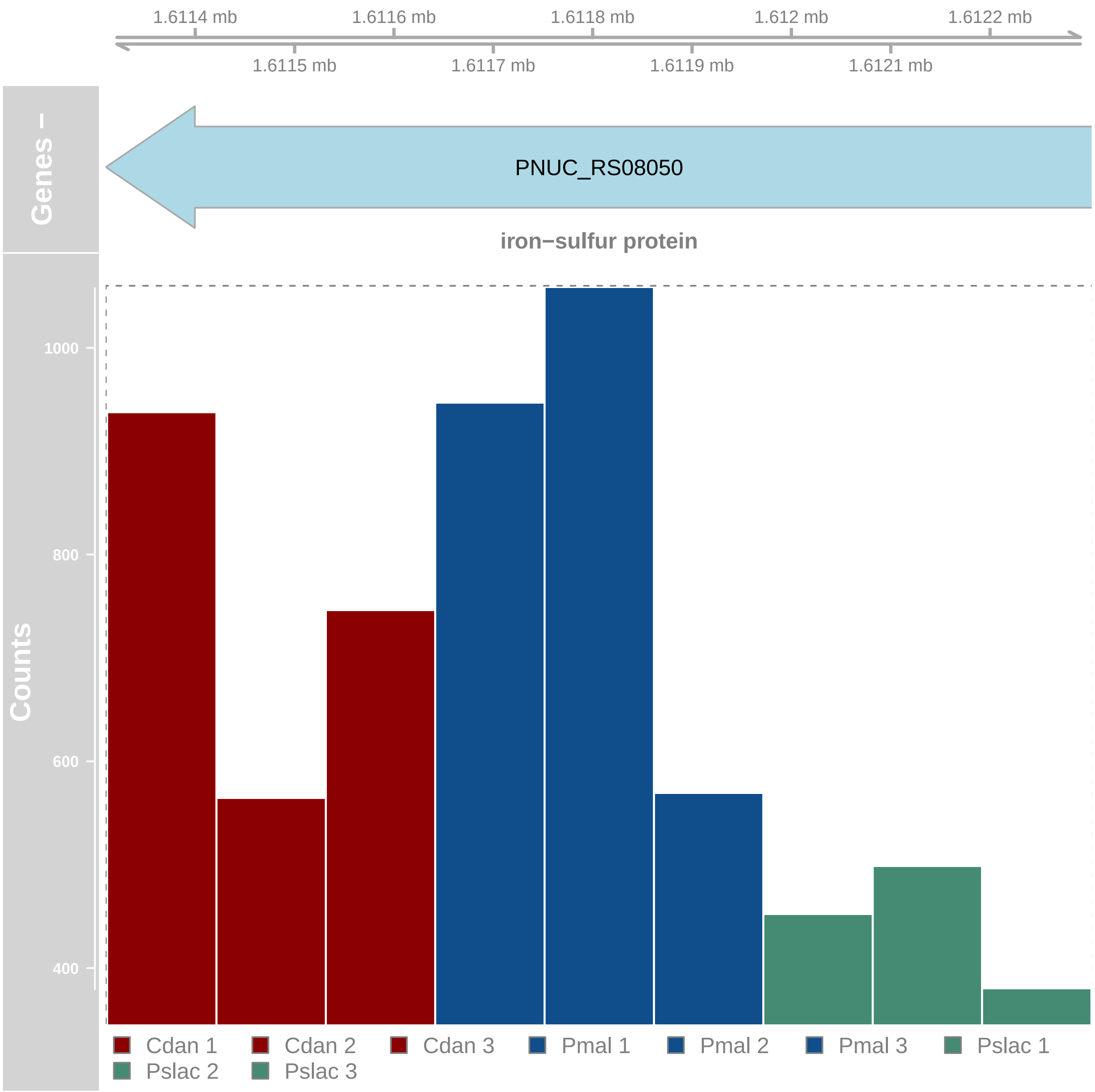

1.6114 mb
1.6116 mb
1.6118 mb
1.612 mb
1.6122 mb
1.6115 mb
1.6117 mb
1.6119 mb
1.6121 mb
Genes −
PNUC_RS08050
iron−sulfur protein
Counts
1000
800
600
400
Cdan 1
Cdan 2
Cdan 3
Pmal 1
Pmal 2
Pmal 3
Pslac 1
Pslac 2
Pslac 3

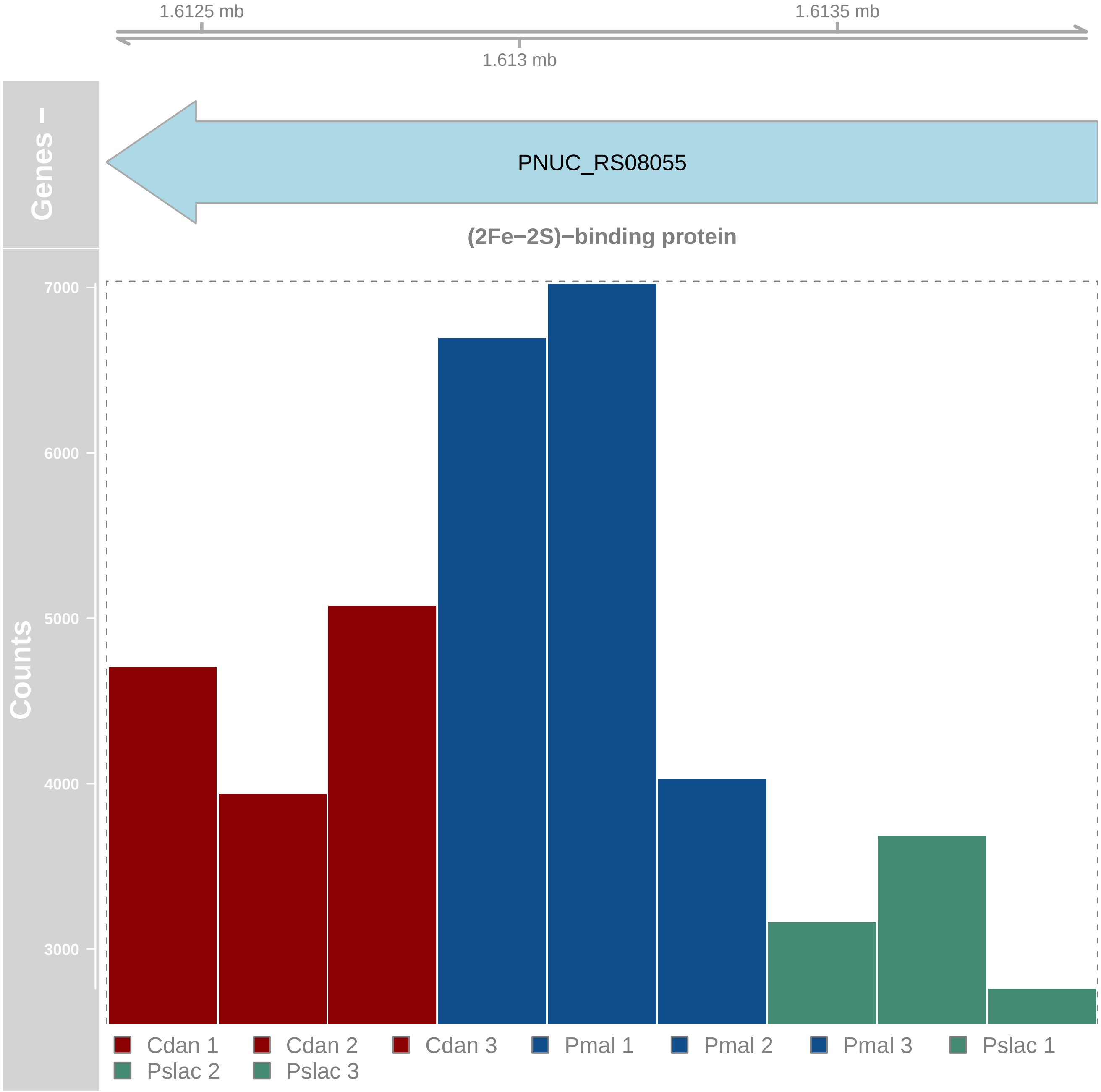

1.6125 mb
1.6135 mb
1.613 mb
Genes −
PNUC_RS08055
(2Fe−2S)−binding protein
Counts
7000
6000
5000
4000
3000
Cdan 1
Cdan 2
Cdan 3
Pmal 1
Pmal 2
Pmal 3
Pslac 1
Pslac 2
Pslac 3

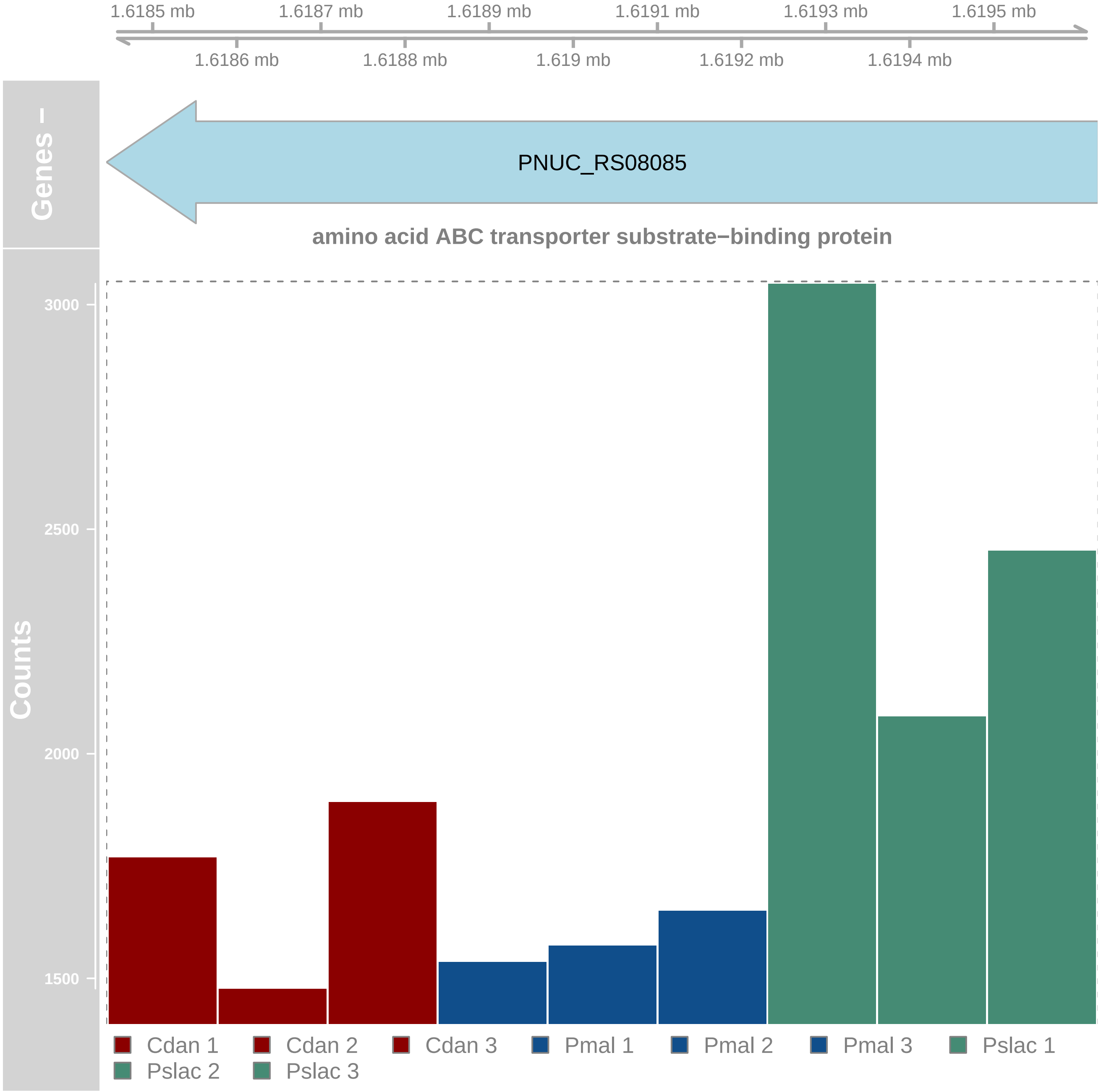

1.6185 mb
1.6186 mb
1.6187 mb
1.6188 mb
1.6189 mb
1.619 mb
1.6191 mb
1.6192 mb
1.6193 mb
1.6194 mb
1.6195 mb
Genes −
PNUC_RS08085
amino acid ABC transporter substrate−binding protein
Counts
3000
2500
2000
1500
Cdan 1
Cdan 2
Cdan 3
Pmal 1
Pmal 2
Pmal 3
Pslac 1
Pslac 2
Pslac 3

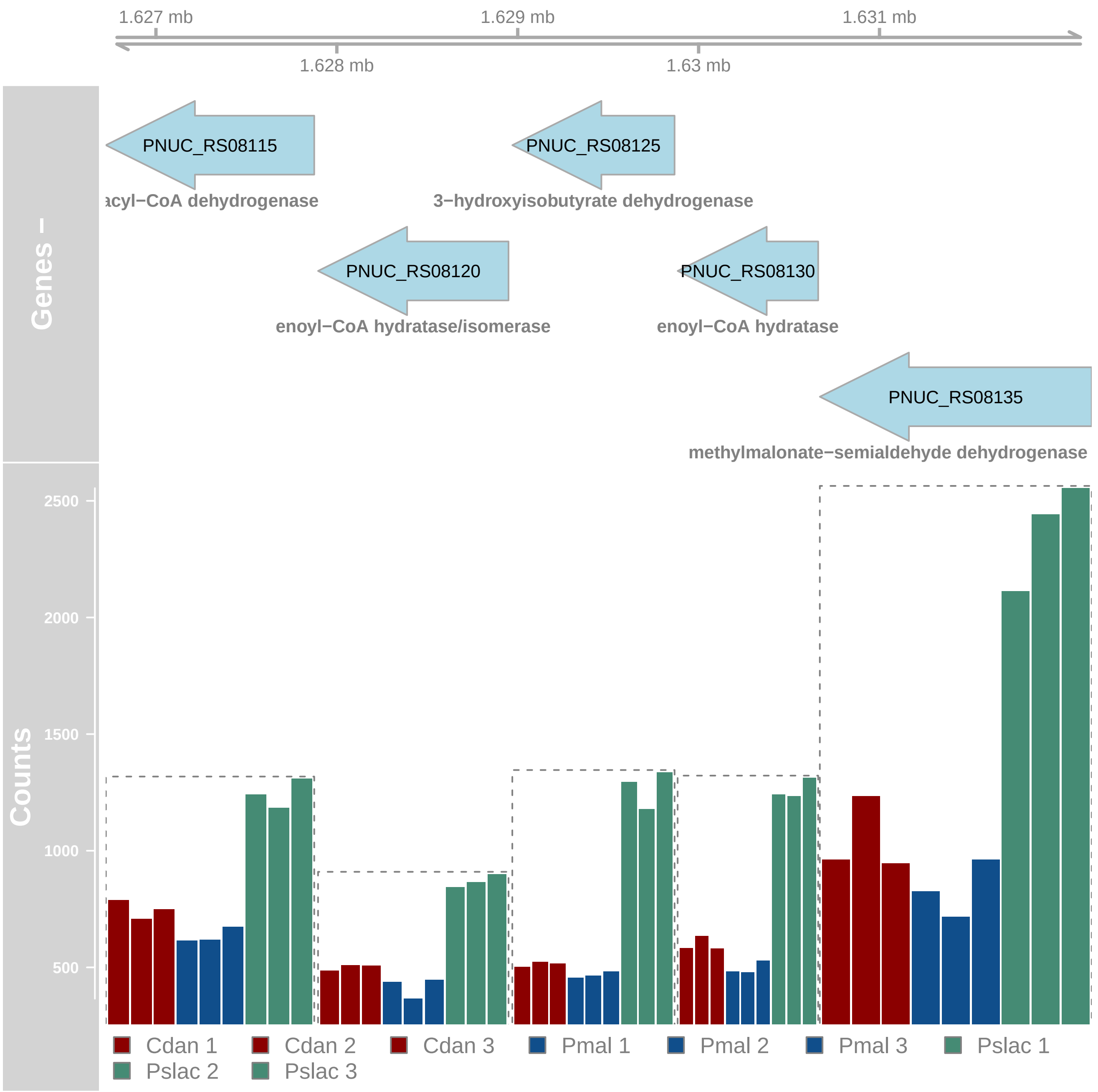
1.627 mb
1.628 mb
1.629 mb
1.63 mb
1.631 mb
Genes −
PNUC_RS08115
acyl−CoA dehydrogenase
PNUC_RS08125
3−hydroxyisobutyrate dehydrogenase
PNUC_RS08120
enoyl−CoA hydratase/isomerase
PNUC_RS08130
enoyl−CoA hydratase
PNUC_RS08135
methylmalonate−semialdehyde dehydrogenase
Counts
2500
2000
1500
1000
500
Cdan 1
Cdan 2
Cdan 3
Pmal 1
Pmal 2
Pmal 3
Pslac 1
Pslac 2
Pslac 3

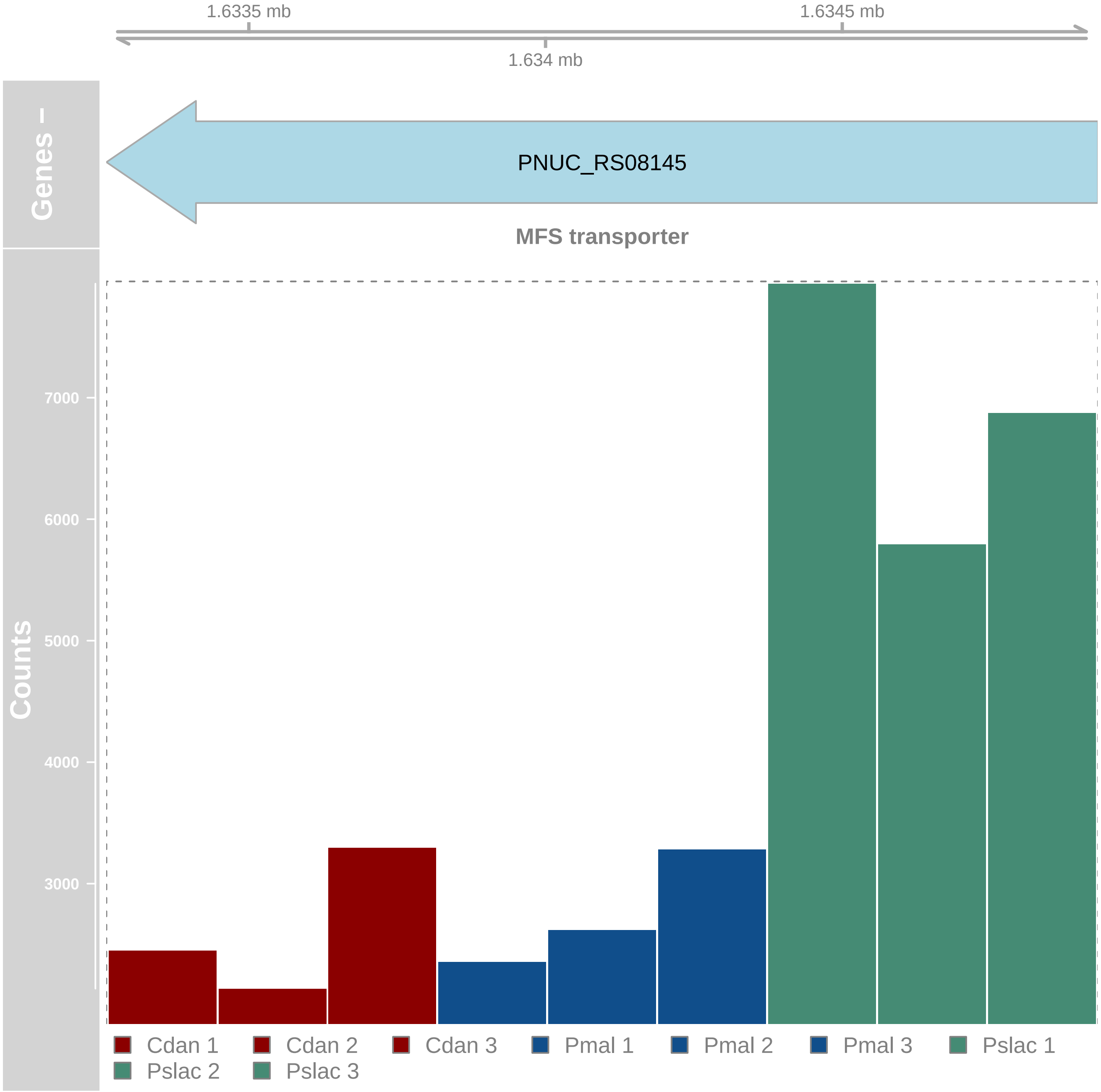
1.6335 mb
1.6345 mb
1.634 mb
Genes –
PNUC_RS08145
MFS transporter
Counts
7000
6000
5000
4000
3000
Cdan 1
Cdan 2
Cdan 3
Pmal 1
Pmal 2
Pmal 3
Pslac 1
Pslac 2
Pslac 3

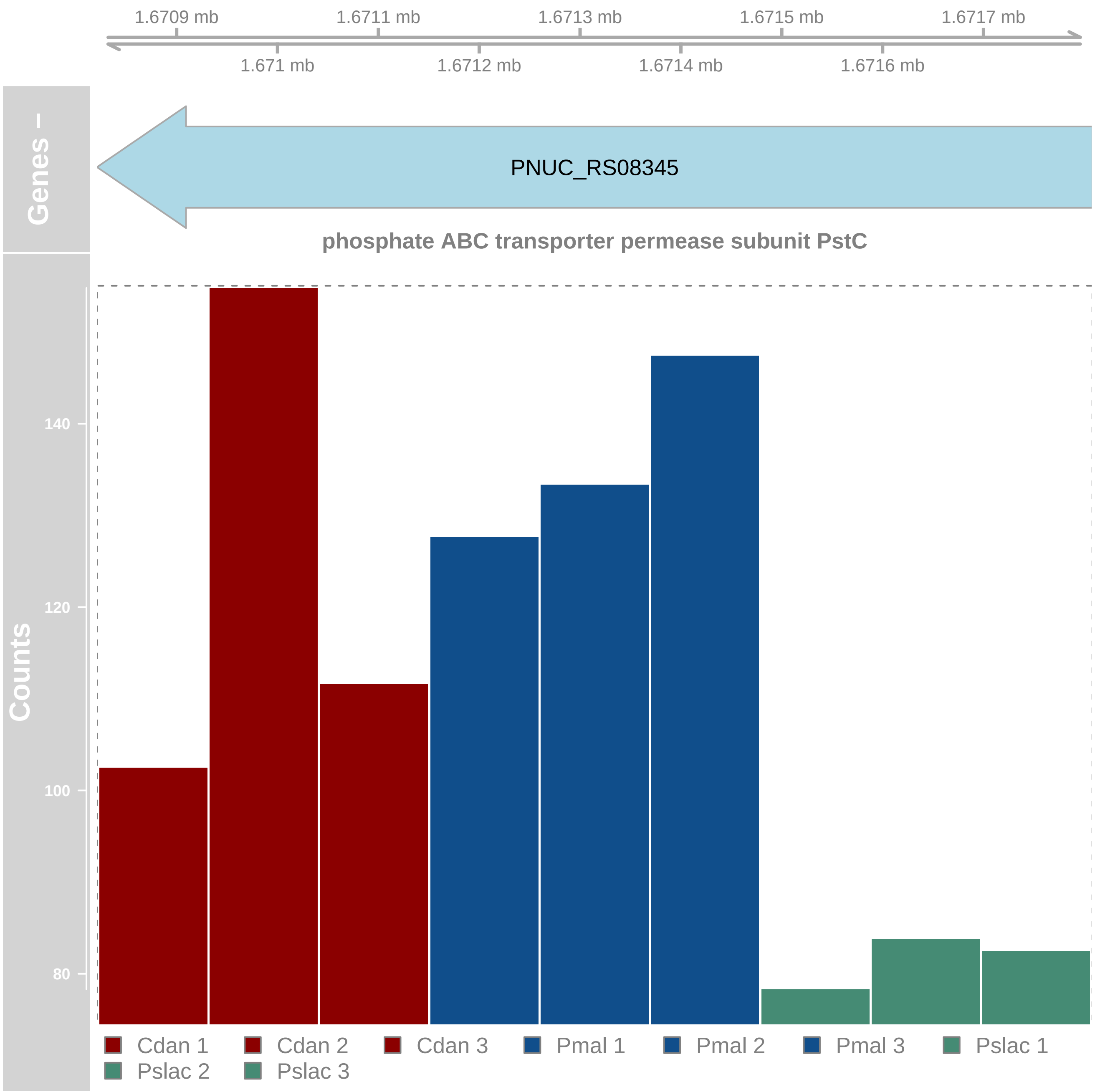
1.6709 mb
1.6711 mb
1.6713 mb
1.6715 mb
1.6717 mb
1.671 mb
1.6712 mb
1.6714 mb
1.6716 mb
Genes –
PNUC_RS08345
phosphate ABC transporter permease subunit PstC
Counts
140
120
100
80
Cdan 1
Cdan 2
Cdan 3
Pmal 1
Pmal 2
Pmal 3
Pslac 1
Pslac 2
Pslac 3

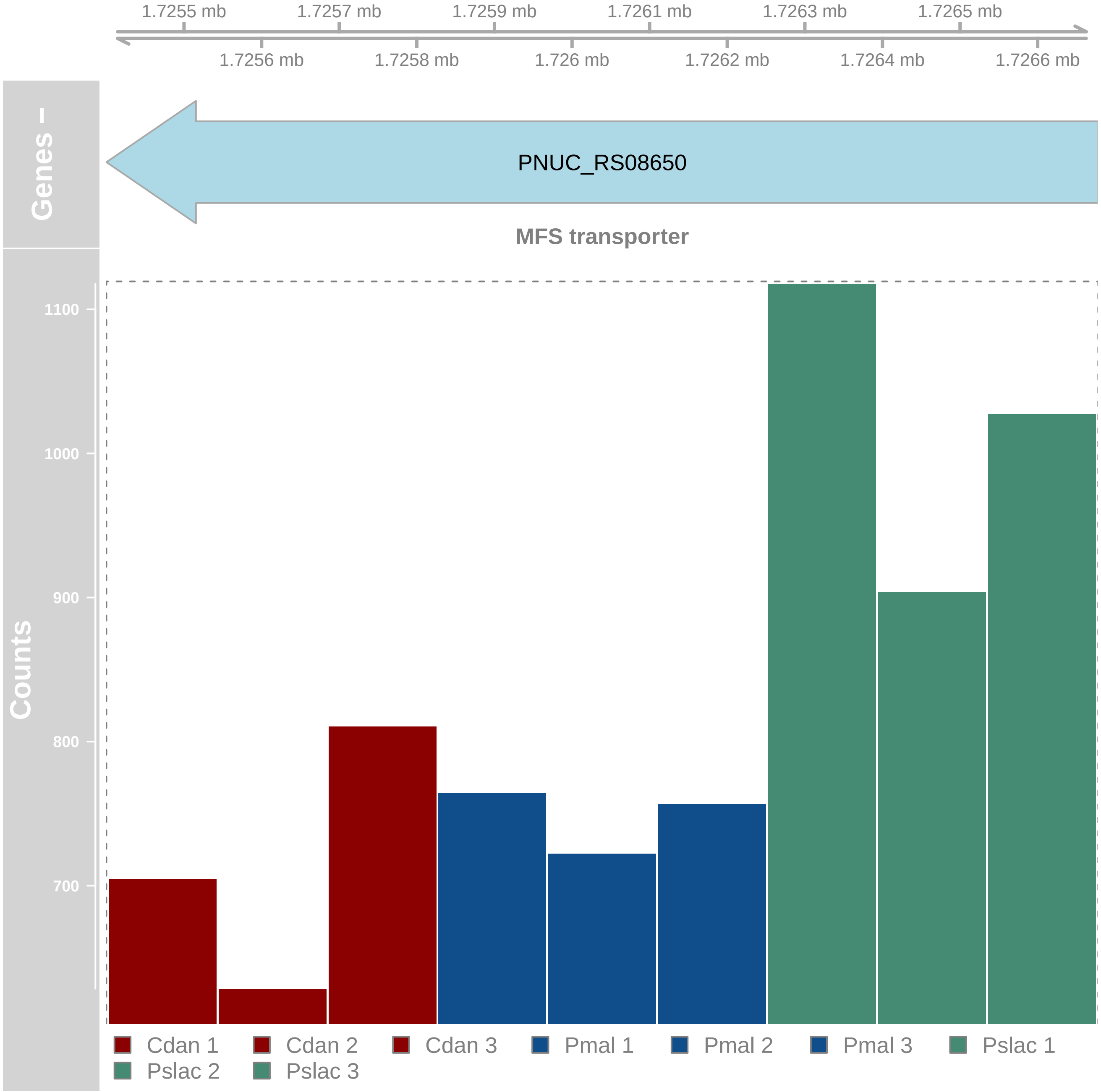
1.7255 mb
1.7257 mb
1.7259 mb
1.7261 mb
1.7263 mb
1.7265 mb
1.7256 mb
1.7258 mb
1.726 mb
1.7262 mb
1.7264 mb
1.7266 mb
Genes –
PNUC_RS08650
MFS transporter
Counts
1100
1000
900
800
700
Cdan 1
Cdan 2
Cdan 3
Pmal 1
Pmal 2
Pmal 3
Pslac 1
Pslac 2
Pslac 3

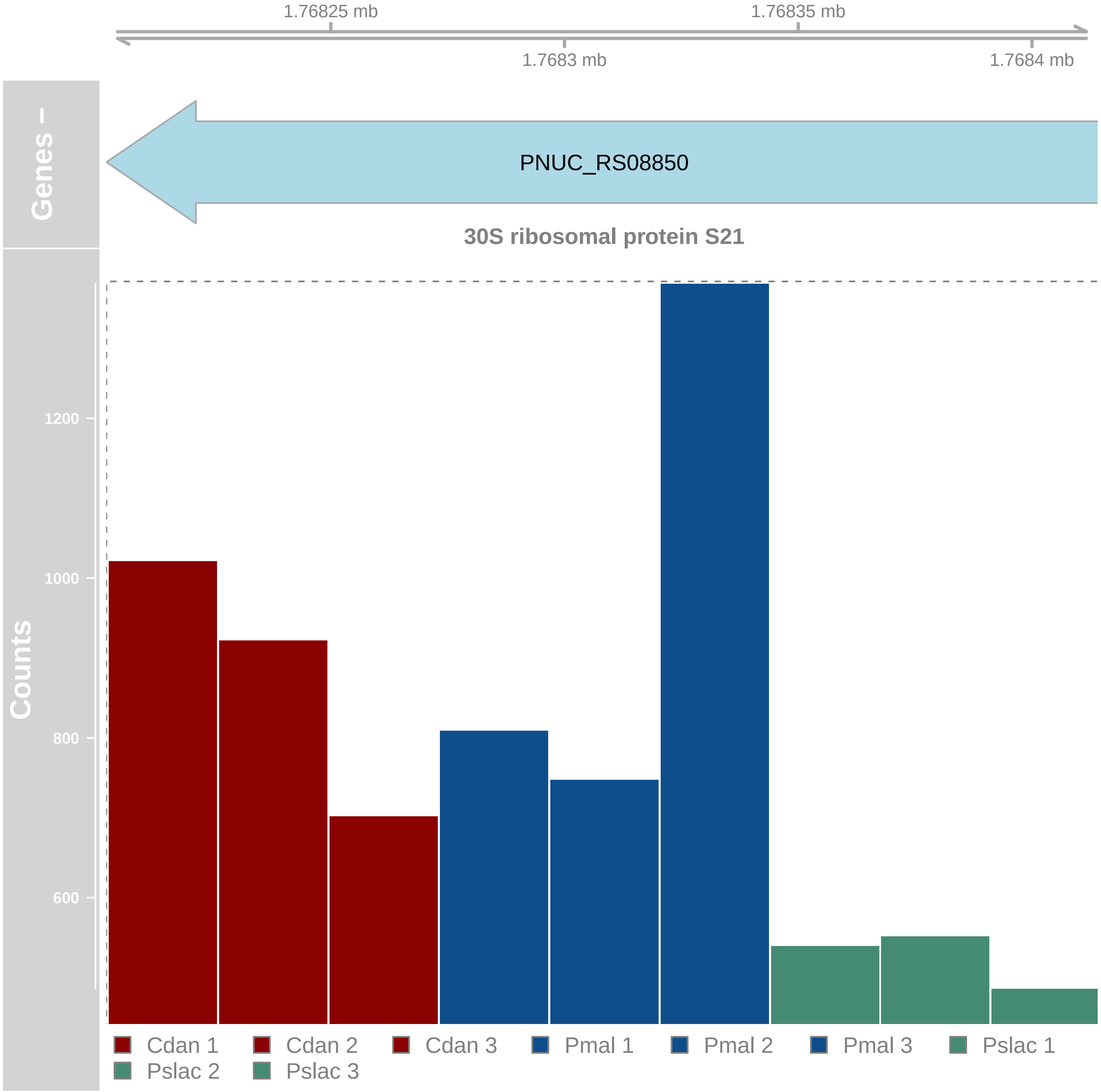
1.76825 mb
1.76835 mb
1.7683 mb
1.7684 mb
Genes –
PNUC_RS08850
30S ribosomal protein S21
Counts
1200
1000
800
600
Cdan 1
Cdan 2
Cdan 3
Pmal 1
Pmal 2
Pmal 3
Pslac 1
Pslac 2
Pslac 3

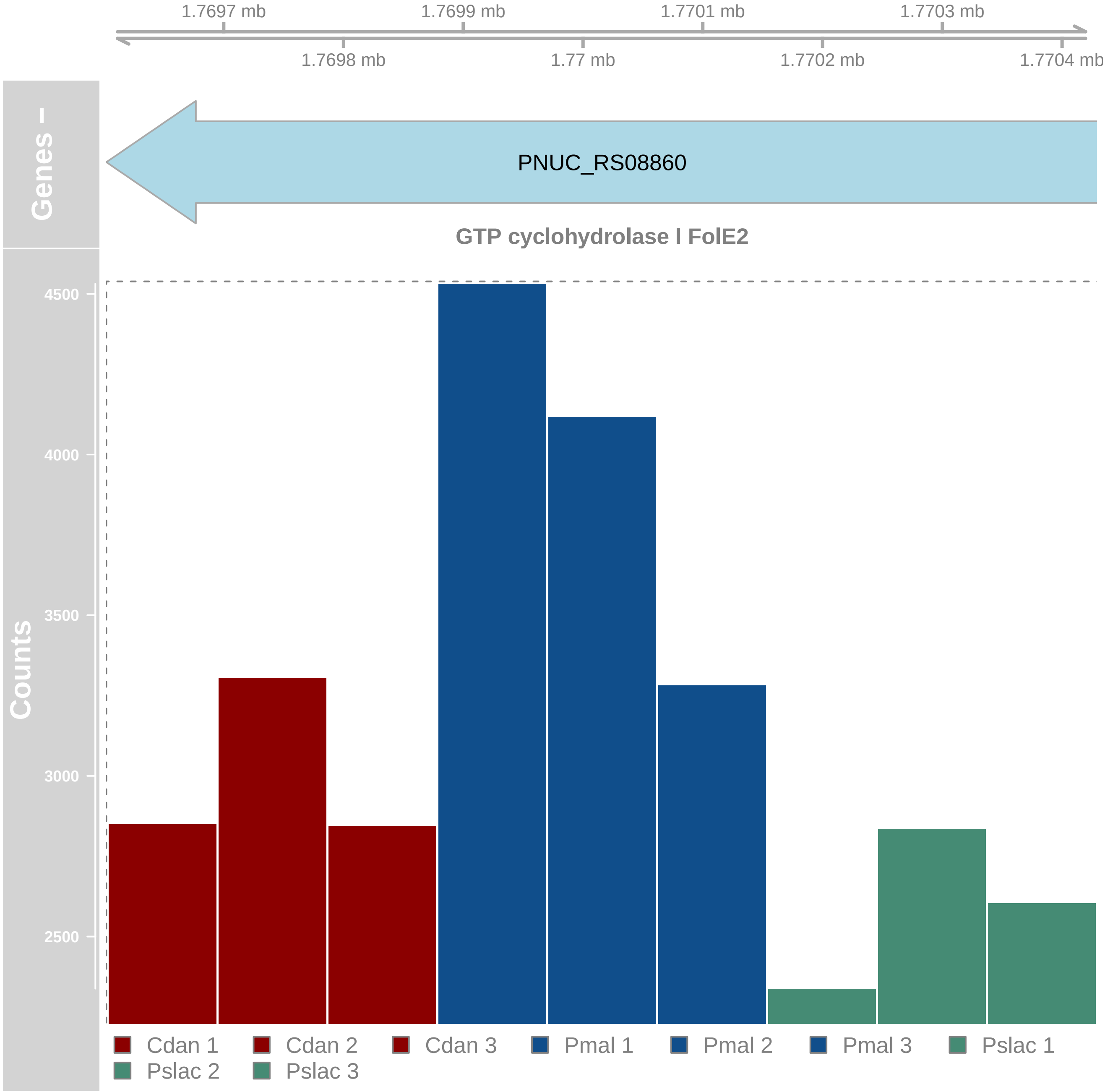

1.7697 mb
1.7698 mb
1.7699 mb
1.77 mb
1.7701 mb
1.7702 mb
1.7703 mb
1.7704 mb
Genes –
PNUC_RS08860
GTP cyclohydrolase I FolE2
Counts
4500
4000
3500
3000
2500
Cdan 1
Cdan 2
Cdan 3
Pmal 1
Pmal 2
Pmal 3
Pslac 1
Pslac 2
Pslac 3

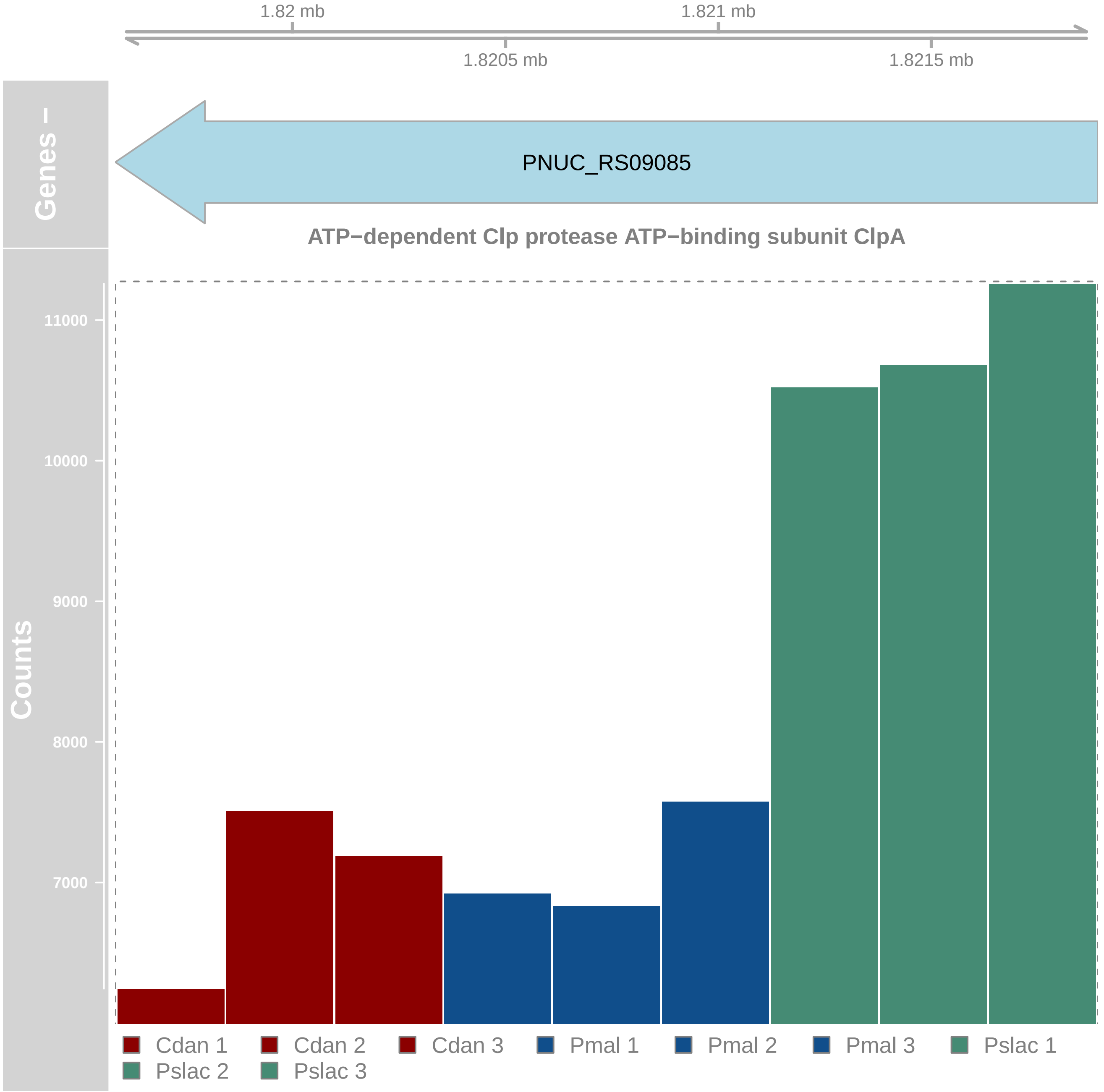
1.82 mb
1.821 mb
1.8205 mb
1.8215 mb
Genes −
PNUC_RS09085
ATP−dependent Clp protease ATP−binding subunit ClpA
Counts
11000
10000
9000
8000
7000
Cdan 1
Cdan 2
Cdan 3
Pmal 1
Pmal 2
Pmal 3
Pslac 1
Pslac 2
Pslac 3

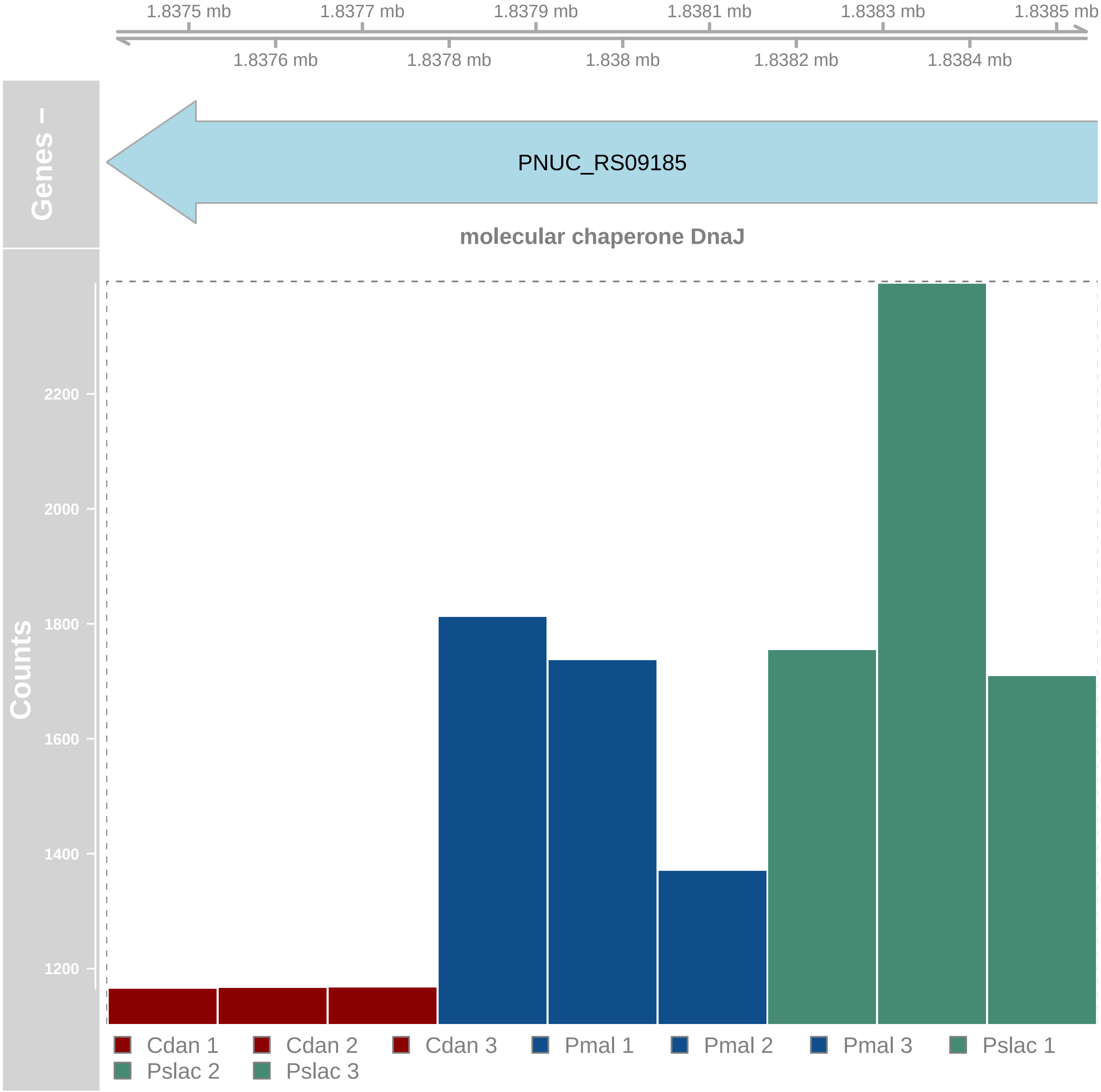
1.8375 mb
1.8376 mb
1.8377 mb
1.8378 mb
1.8379 mb
1.838 mb
1.8381 mb
1.8382 mb
1.8383 mb
1.8384 mb
1.8385 mb
Genes –
PNUC_RS09185
molecular chaperone DnaJ
Counts
2200
2000
1800
1600
1400
1200
Cdan 1
Cdan 2
Cdan 3
Pmal 1
Pmal 2
Pmal 3
Pslac 1
Pslac 2
Pslac 3

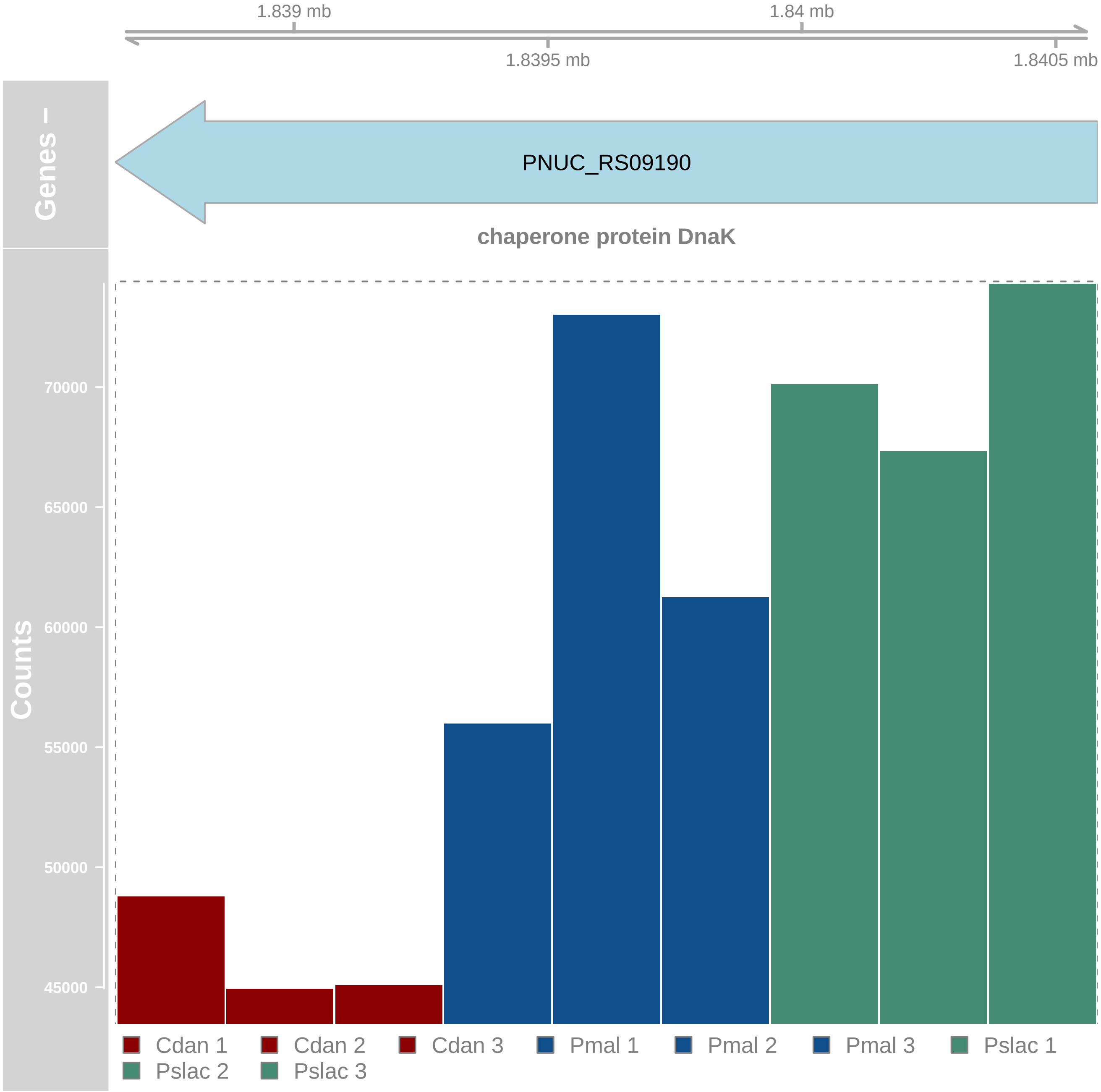
1.839 mb
1.84 mb
1.8395 mb
1.8405 mb
Genes –
PNUC_RS09190
chaperone protein DnaK
Counts
70000
65000
60000
55000
50000
45000
Cdan 1
Cdan 2
Cdan 3
Pmal 1
Pmal 2
Pmal 3
Pslac 1
Pslac 2
Pslac 3

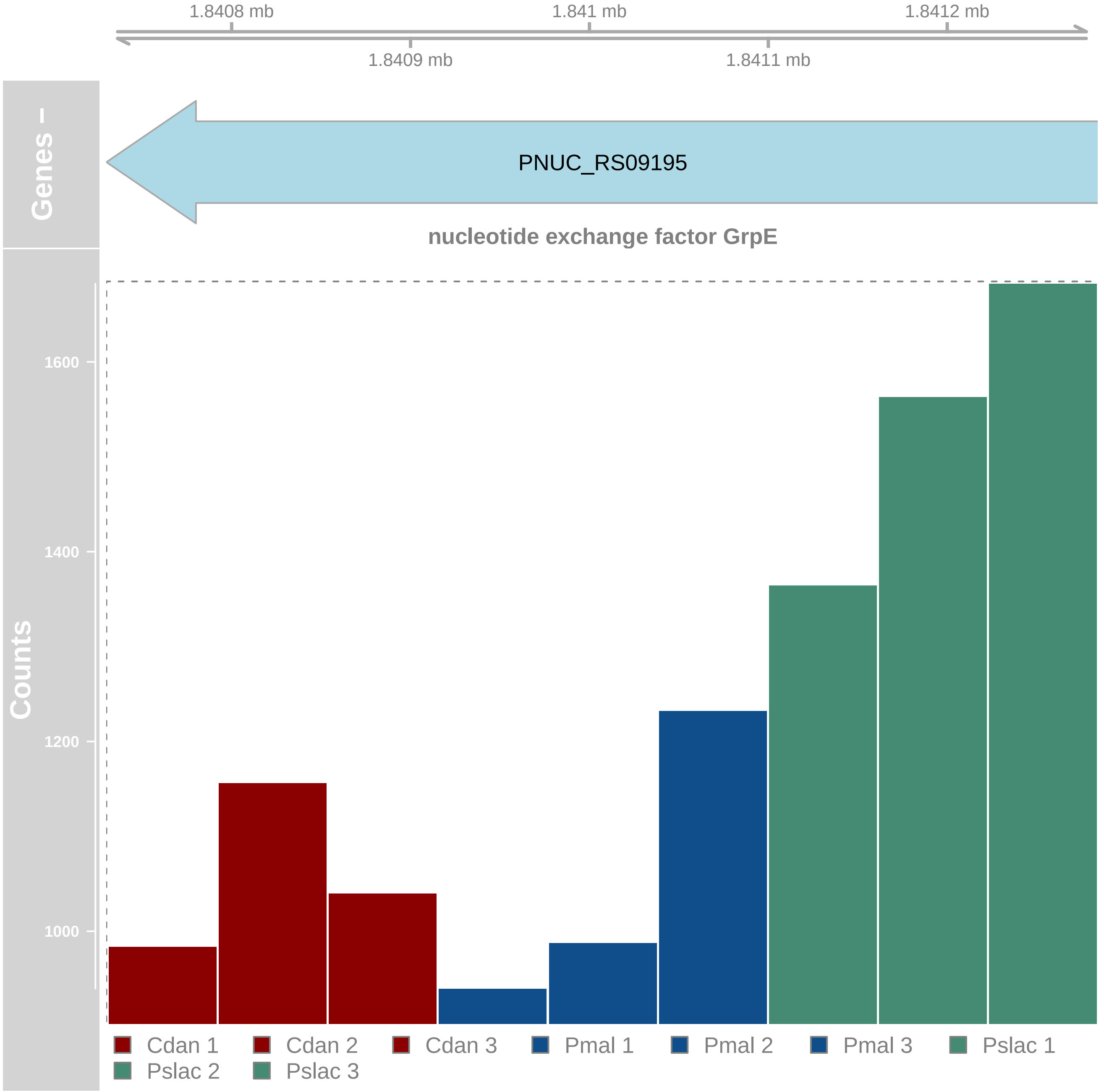

1.8408 mb
1.841 mb
1.8412 mb
1.8409 mb
1.8411 mb
Genes –
PNUC_RS09195
nucleotide exchange factor GrpE
Counts
1600
1400
1200
1000
Cdan 1
Cdan 2
Cdan 3
Pmal 1
Pmal 2
Pmal 3
Pslac 1
Pslac 2
Pslac 3

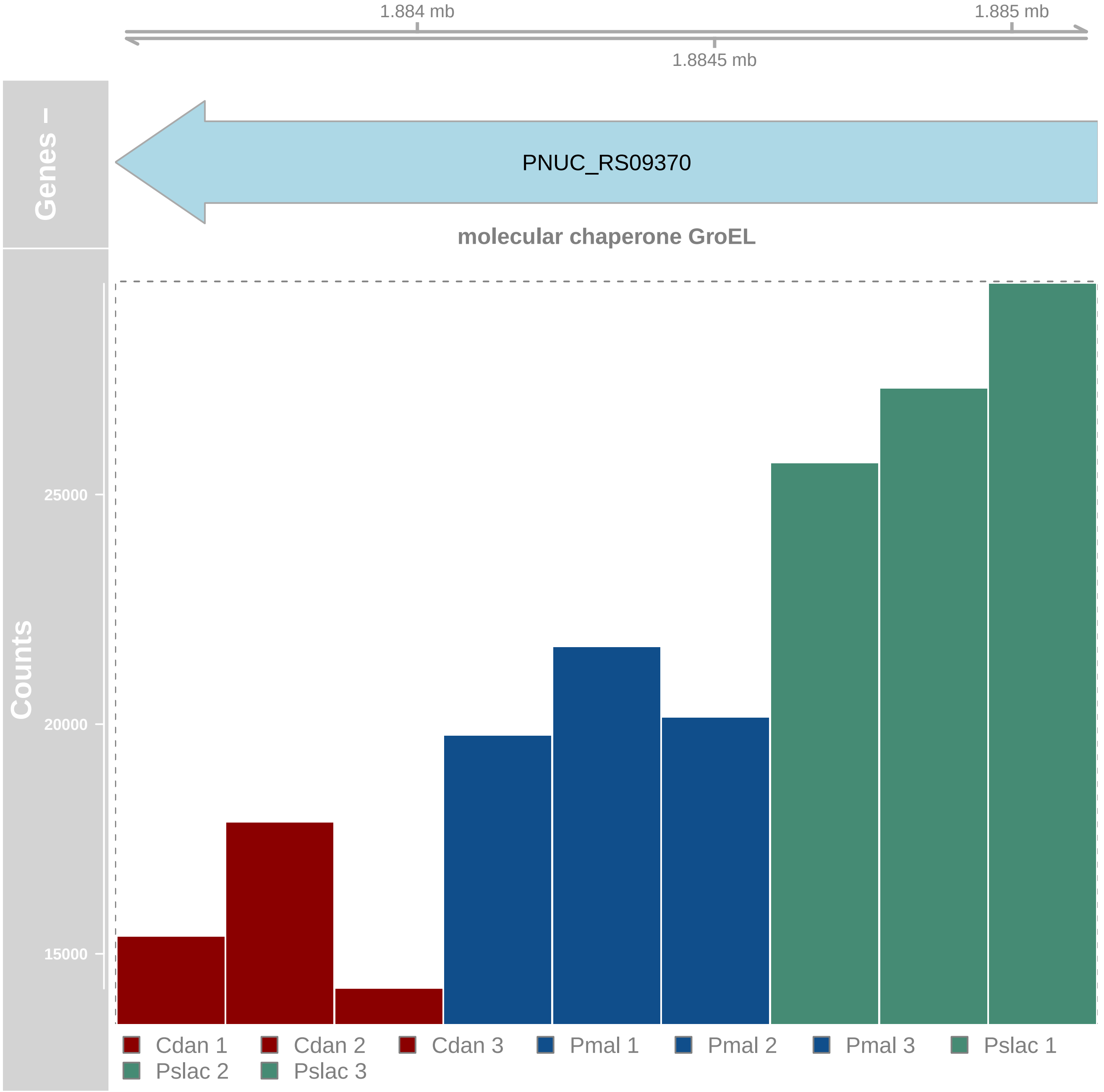

1.884 mb
1.885 mb
1.8845 mb
Genes –
PNUC_RS09370
molecular chaperone GroEL
Counts
25000
20000
15000
Cdan 1
Cdan 2
Cdan 3
Pmal 1
Pmal 2
Pmal 3
Pslac 1
Pslac 2
Pslac 3

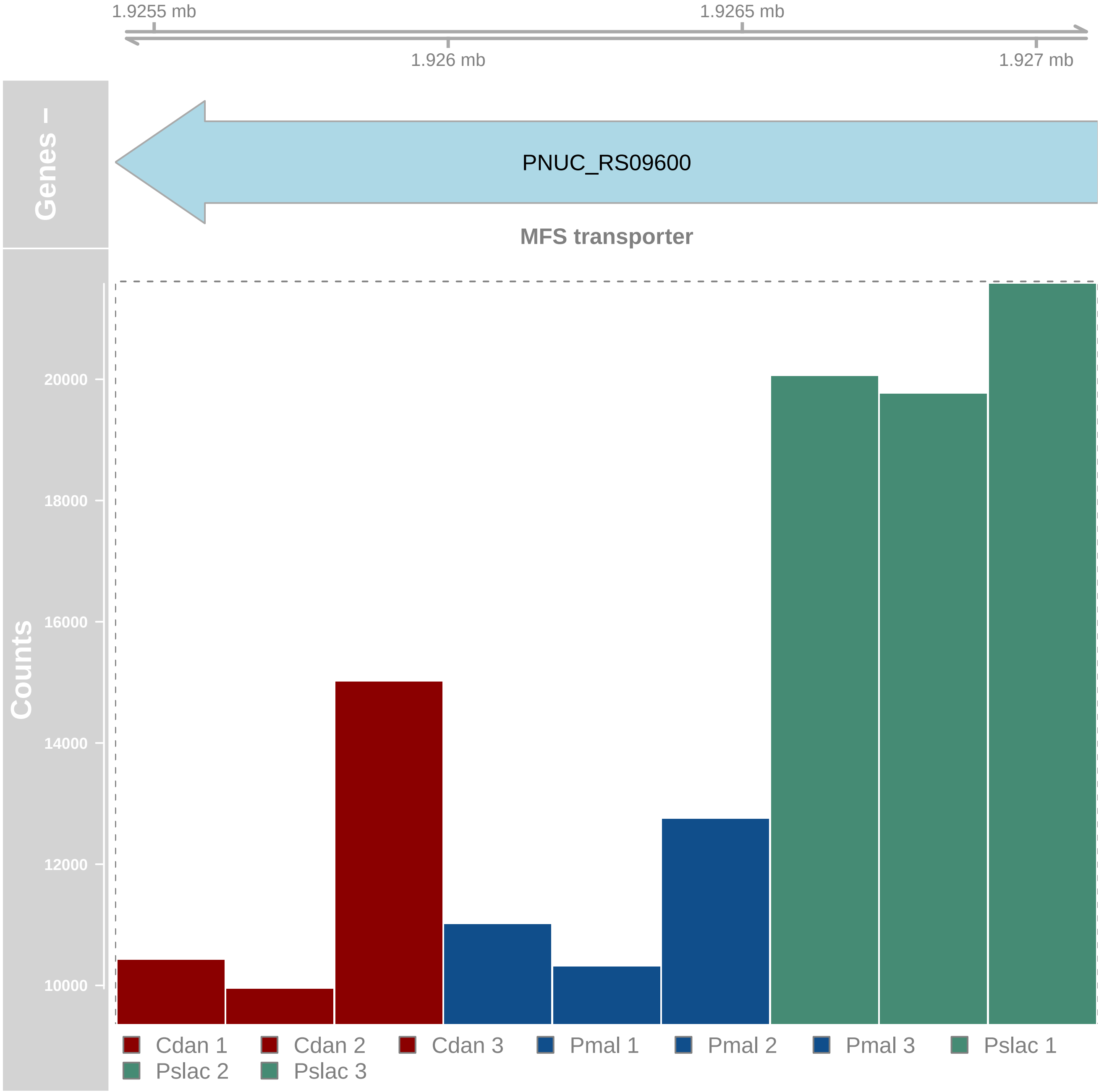

1.9255 mb
1.9265 mb
1.926 mb
1.927 mb
Genes −
PNUC_RS09600
MFS transporter
Counts
20000
18000
16000
14000
12000
10000
Cdan 1
Cdan 2
Cdan 3
Pmal 1
Pmal 2
Pmal 3
Pslac 1
Pslac 2
Pslac 3

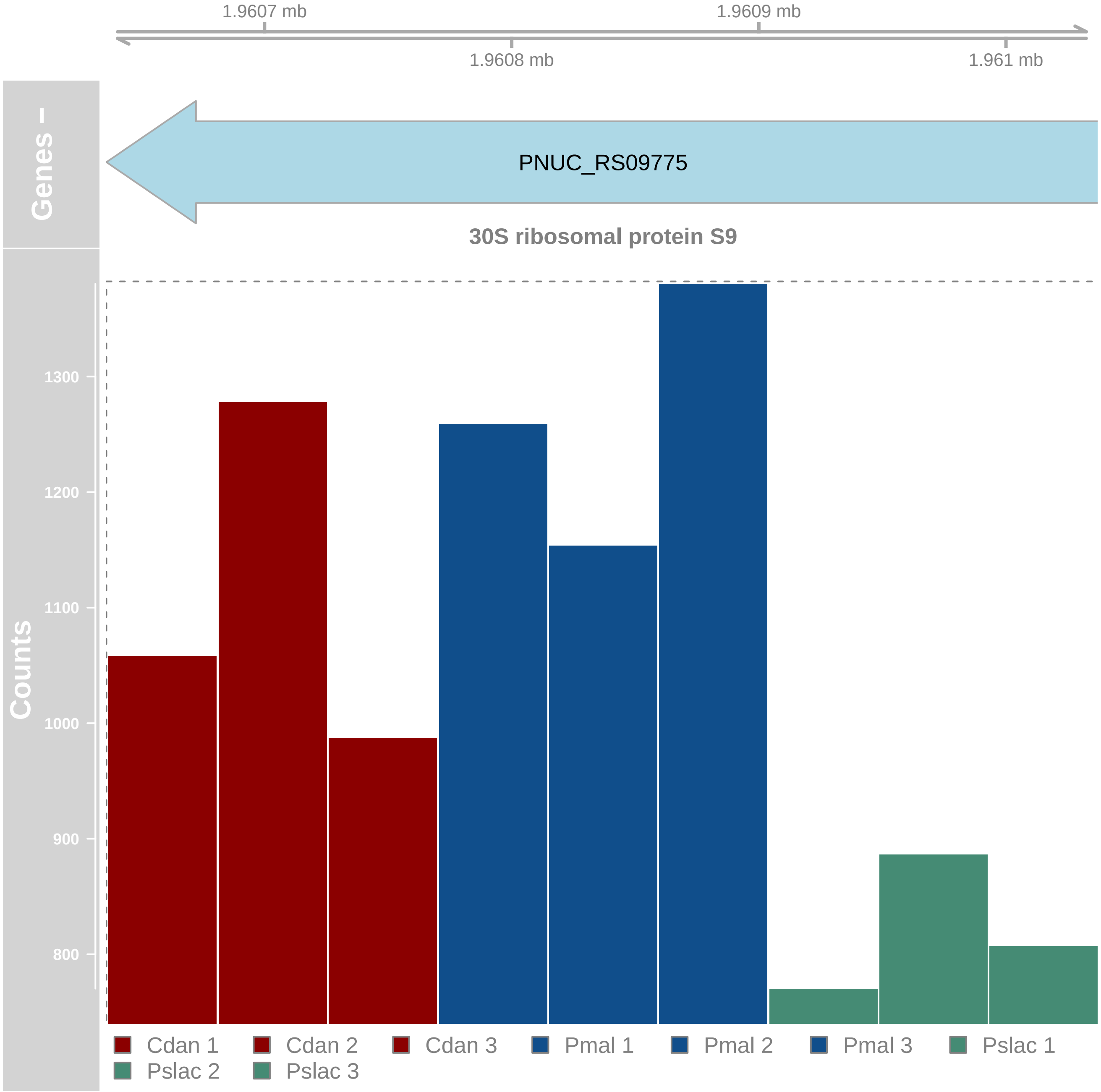
1.9607 mb
1.9608 mb
1.9609 mb
1.961 mb
Genes –
PNUC_RS09775
30S ribosomal protein S9
Counts
1300
1200
1100
1000
900
800
Cdan 1
Cdan 2
Cdan 3
Pmal 1
Pmal 2
Pmal 3
Pslac 1
Pslac 2
Pslac 3

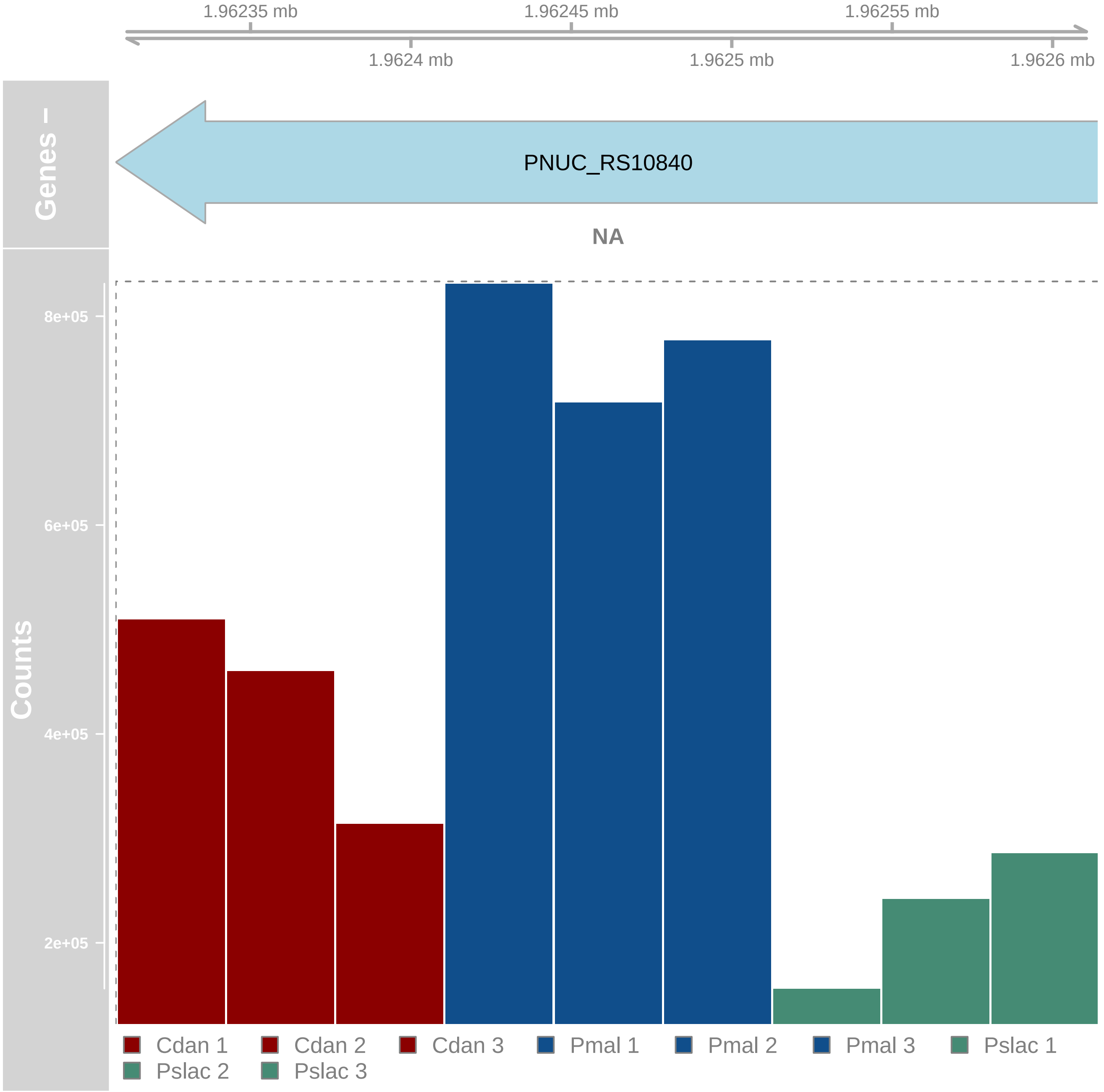
1.96235 mb
1.96245 mb
1.96255 mb
1.9624 mb
1.9625 mb
1.9626 mb
Genes –
PNUC_RS10840
NA
Counts
8e+05
6e+05
4e+05
2e+05
Cdan 1
Cdan 2
Cdan 3
Pmal 1
Pmal 2
Pmal 3
Pslac 1
Pslac 2
Pslac 3

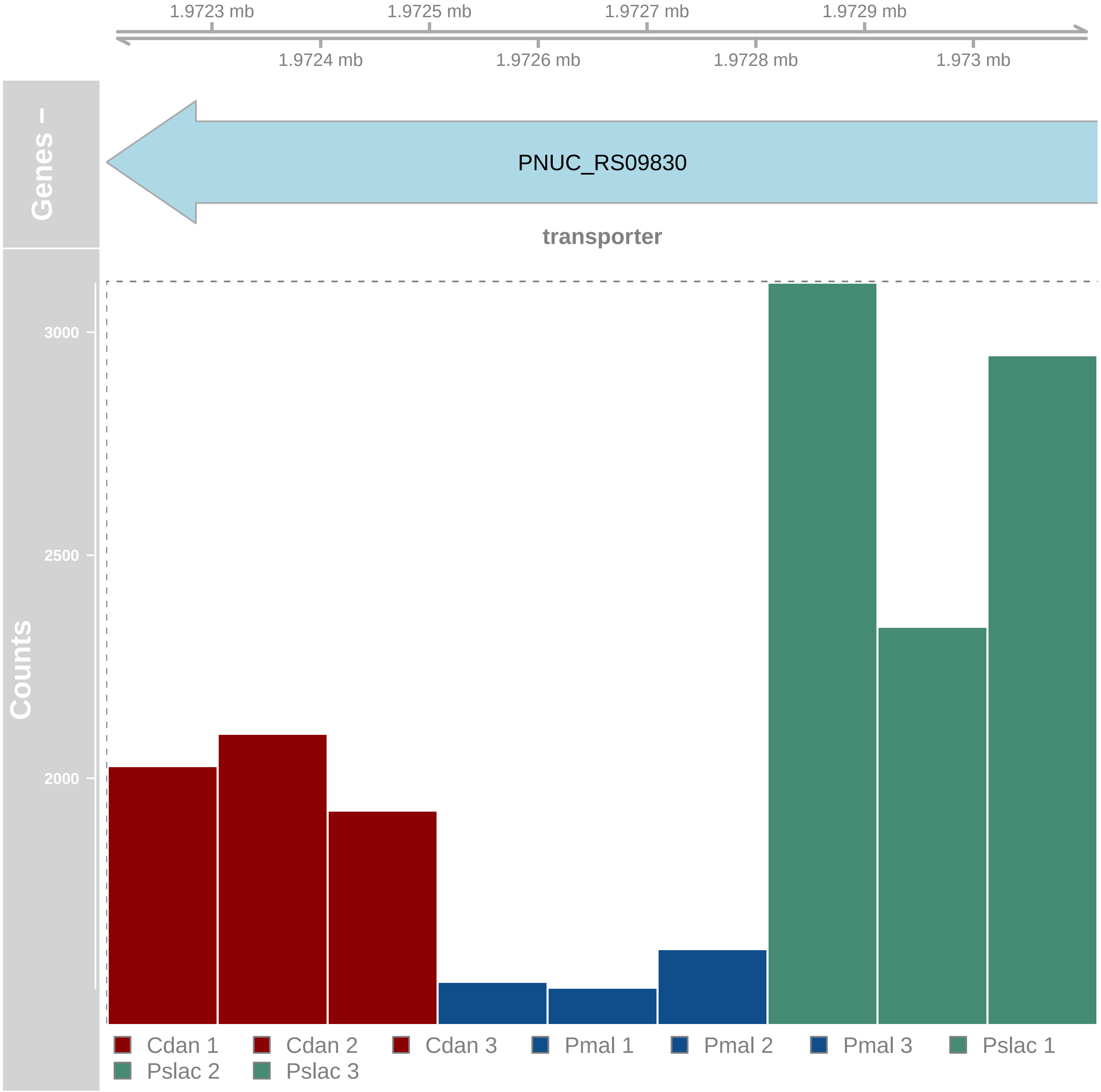
1.9723 mb
1.9724 mb
1.9725 mb
1.9726 mb
1.9727 mb
1.9728 mb
1.9729 mb
1.973 mb
Genes –
PNUC_RS09830
transporter
Counts
3000
2500
2000
Cdan 1
Cdan 2
Cdan 3
Pmal 1
Pmal 2
Pmal 3
Pslac 1
Pslac 2
Pslac 3

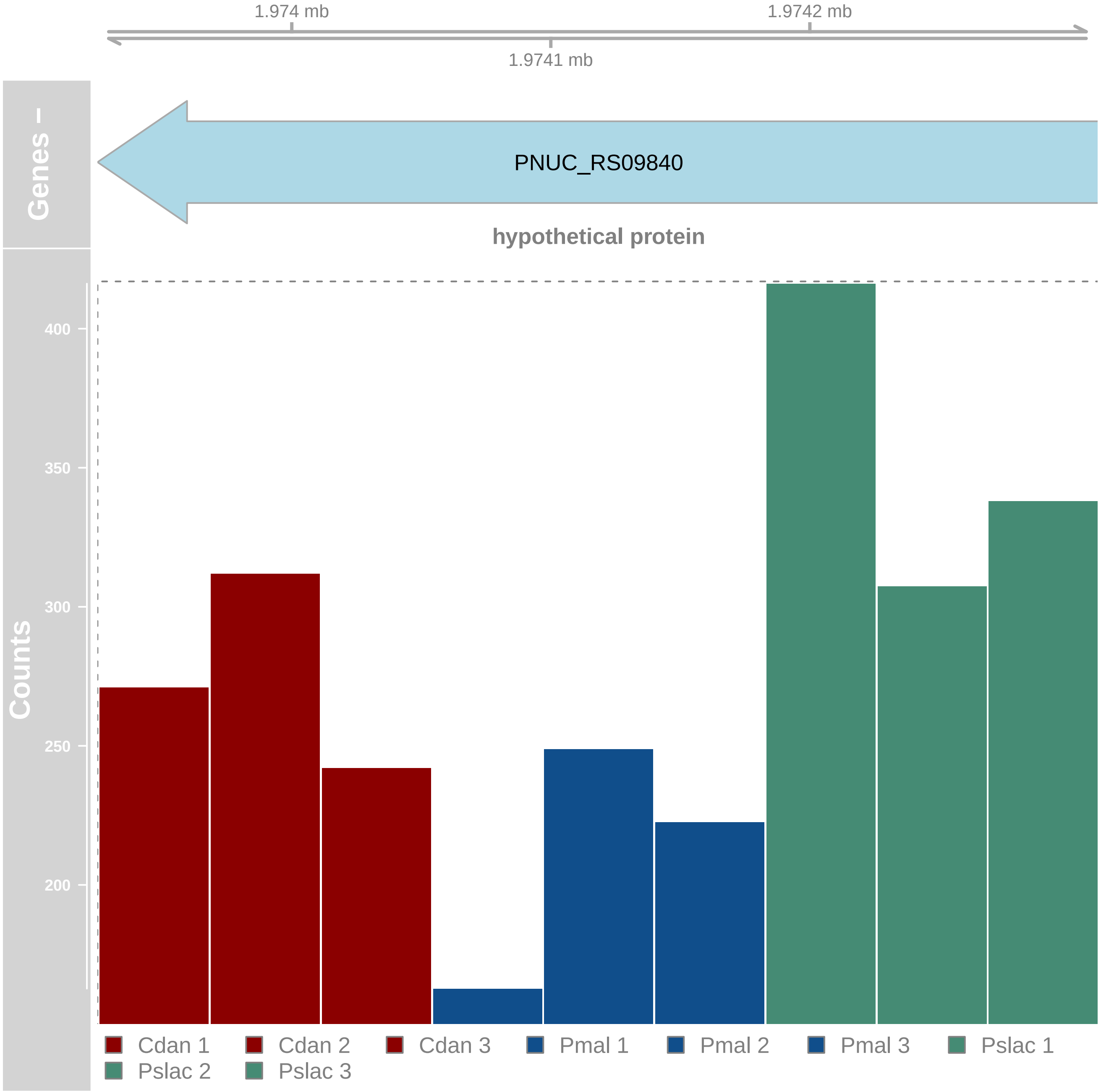
1.974 mb
1.9741 mb
1.9742 mb
Genes –
PNUC_RS09840
hypothetical protein
Counts
400
350
300
250
200
Cdan 1
Cdan 2
Cdan 3
Pmal 1
Pmal 2
Pmal 3
Pslac 1
Pslac 2
Pslac 3

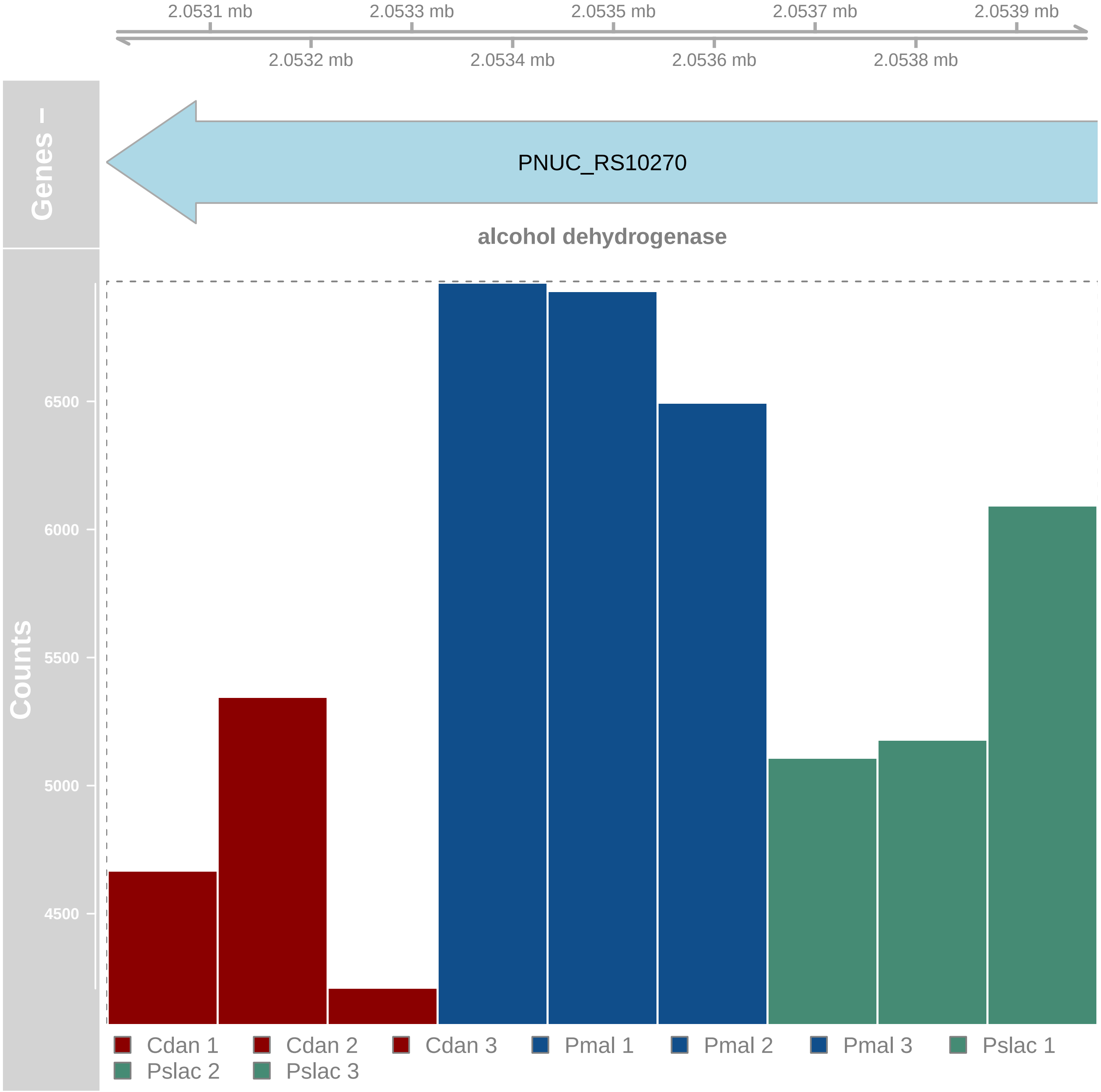
2.0531 mb
2.0533 mb
2.0535 mb
2.0537 mb
2.0539 mb
2.0532 mb
2.0534 mb
2.0536 mb
2.0538 mb
Genes –
PNUC_RS10270
alcohol dehydrogenase
Counts
6500
6000
5500
5000
4500
Cdan 1
Cdan 2
Cdan 3
Pmal 1
Pmal 2
Pmal 3
Pslac 1
Pslac 2
Pslac 3

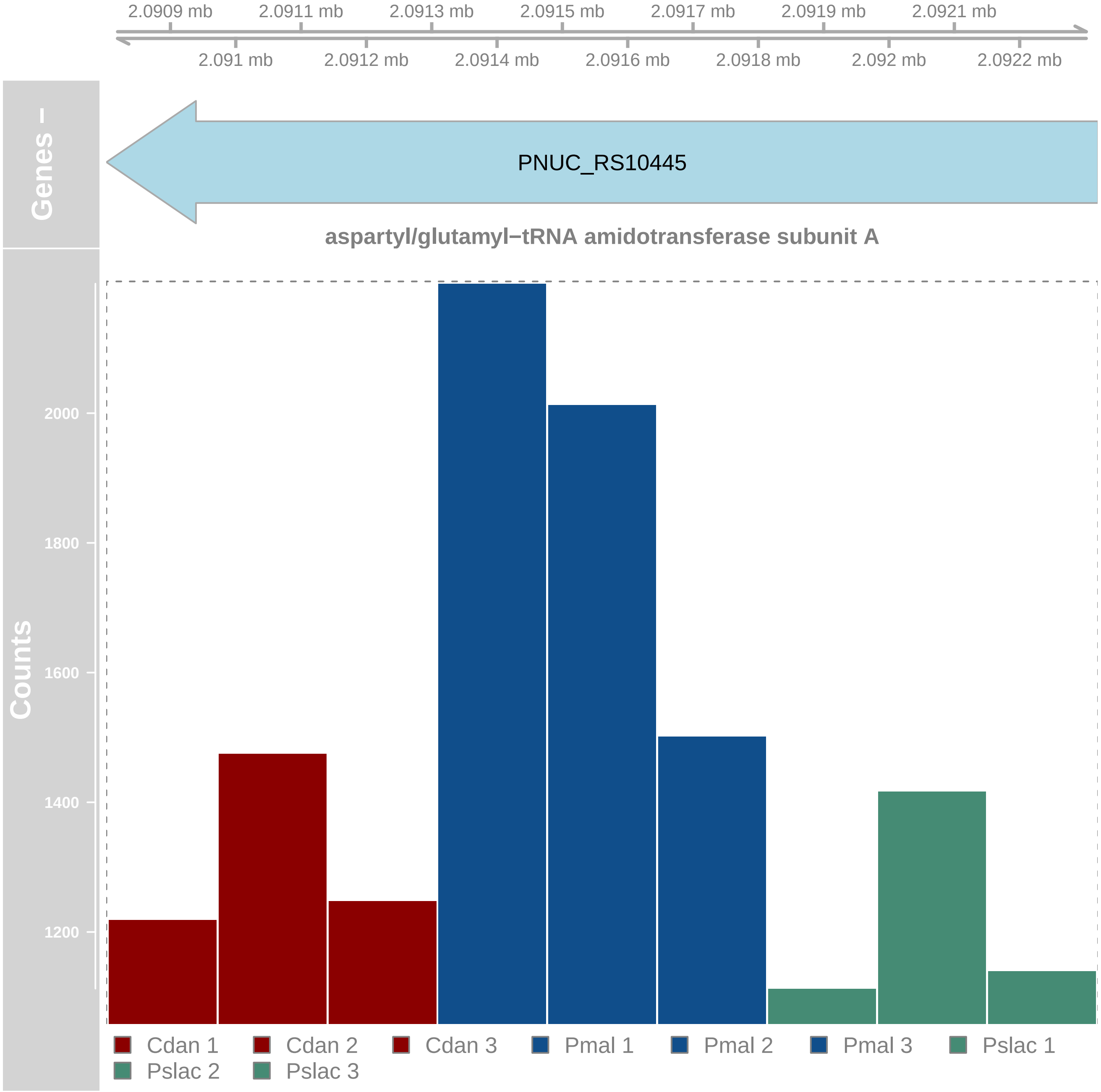

2.0909 mb
2.0911 mb
2.0913 mb
2.0915 mb
2.0917 mb
2.0919 mb
2.0921 mb
2.091 mb
2.0912 mb
2.0914 mb
2.0916 mb
2.0918 mb
2.092 mb
2.0922 mb
Genes −
PNUC_RS10445
aspartyl/glutamyl−tRNA amidotransferase subunit A
Counts
2000
1800
1600
1400
1200
Cdan 1
Cdan 2
Cdan 3
Pmal 1
Pmal 2
Pmal 3
Pslac 1
Pslac 2
Pslac 3
