# Supplementary material for: Interaction-Specific Changes in the Transcriptome of Polynucleobacter asymbioticus Caused by Varying Protistan Communities
Source: Front Microbiol. 2019 Jul 9;10:1498. doi: 10.3389/fmicb.2019.01498 (PMC6629928; doi:10.3389/fmicb.2019.01498)
Supplement: Supplementary file 6 [file Data_Sheet_6.PDF]

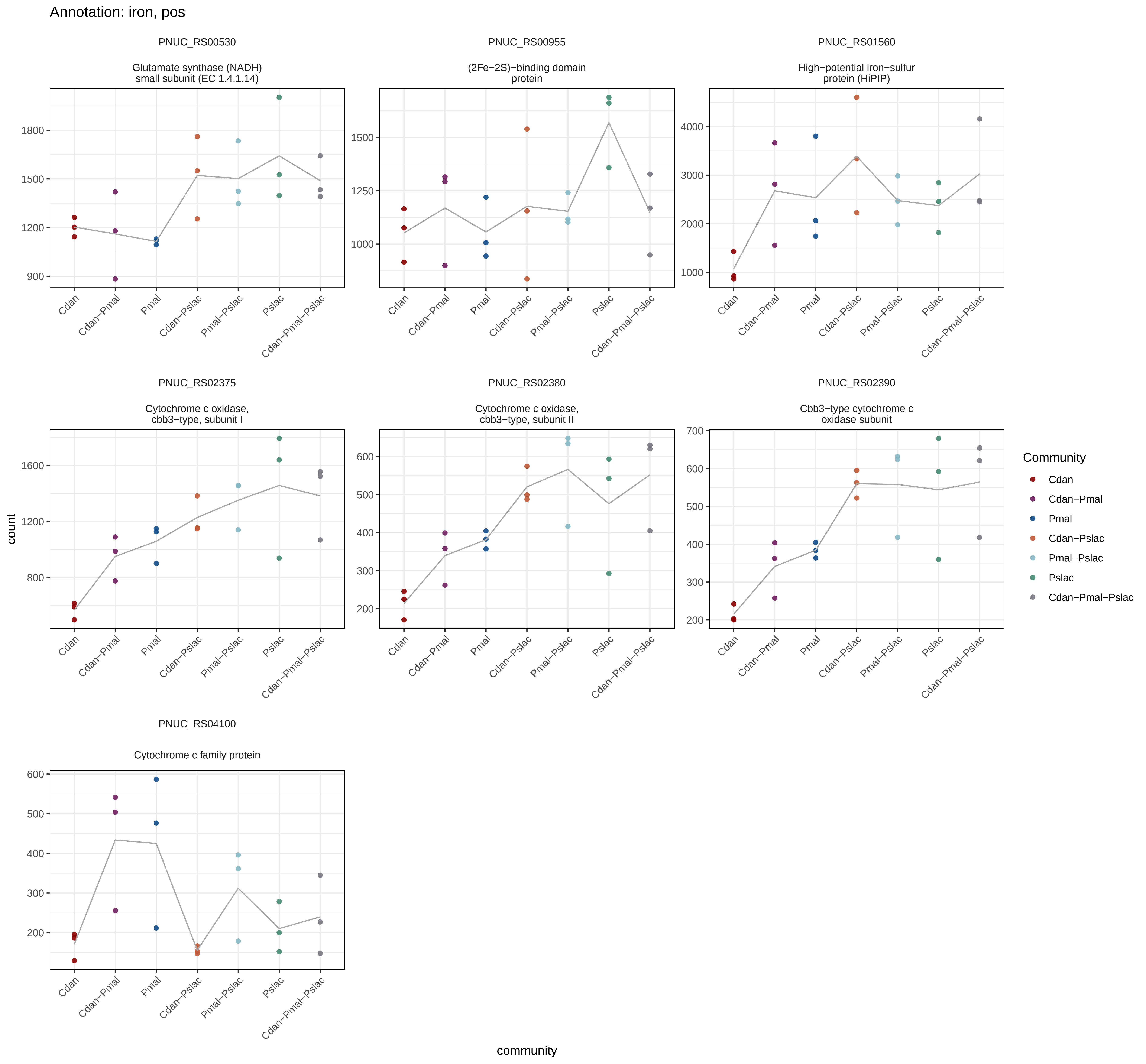
Annotation: iron, pos
PNUC_RS00530
Glutamate synthase (NADH) small subunit (EC 1.4.1.14)
PNUC_RS00955
(2Fe−2S)−binding domain protein
PNUC_RS01560
High−potential iron−sulfur protein (HiPIP)
PNUC_RS02375
Cytochrome c oxidase, cbb3−type, subunit I
PNUC_RS02380
Cytochrome c oxidase, cbb3−type, subunit II
PNUC_RS02390
Cbb3−type cytochrome c oxidase subunit
PNUC_RS04100
Cytochrome c family protein
count
community
Cdan
Cdan−Pmal
Pmal
Cdan−Pslac
Pmal−Pslac
Pslac
Cdan−Pmal−Pslac
Community
Cdan
Cdan−Pmal
Pmal
Cdan−Pslac
Pmal−Pslac
Pslac
Cdan−Pmal−Pslac

Annotation: iron, neg

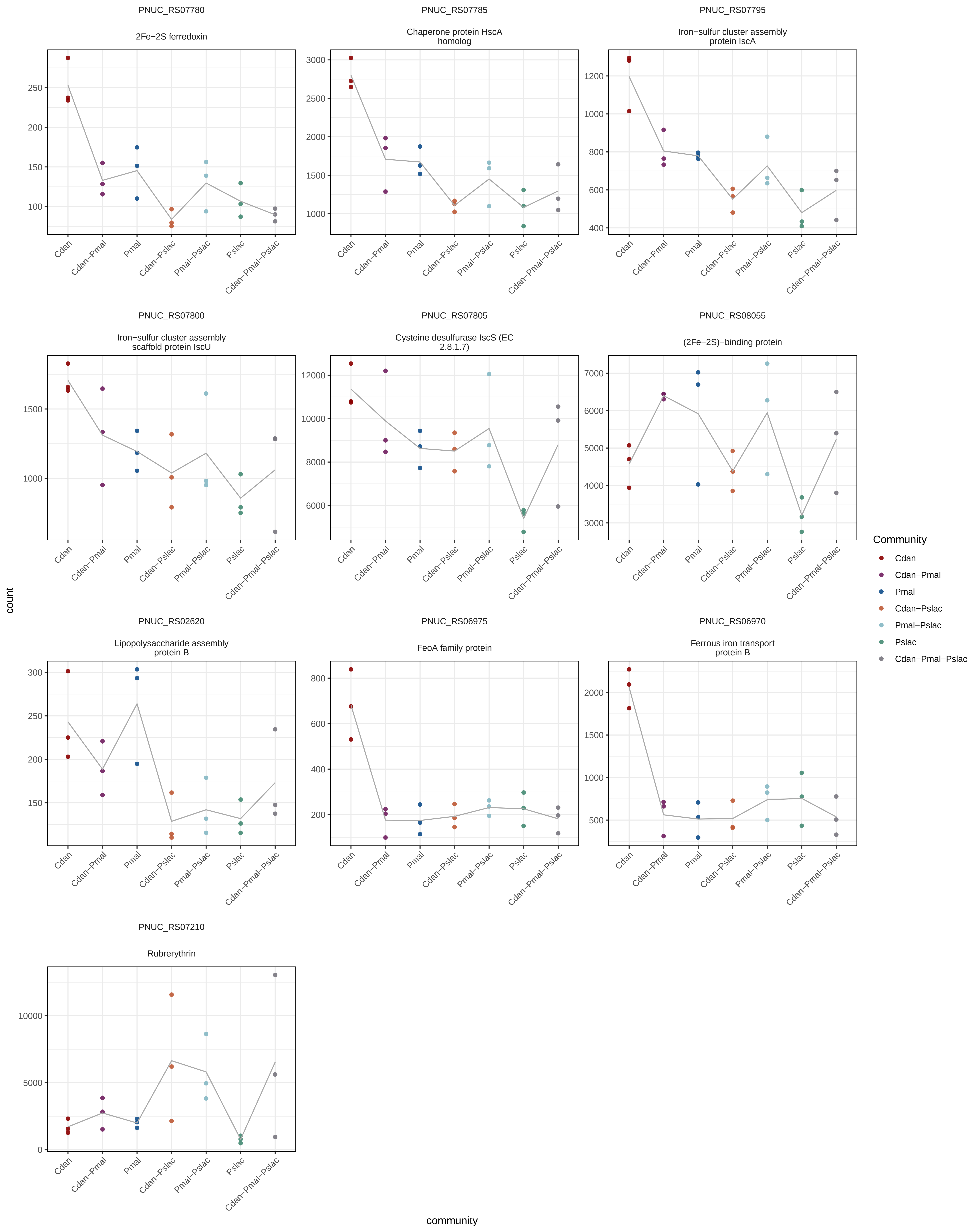

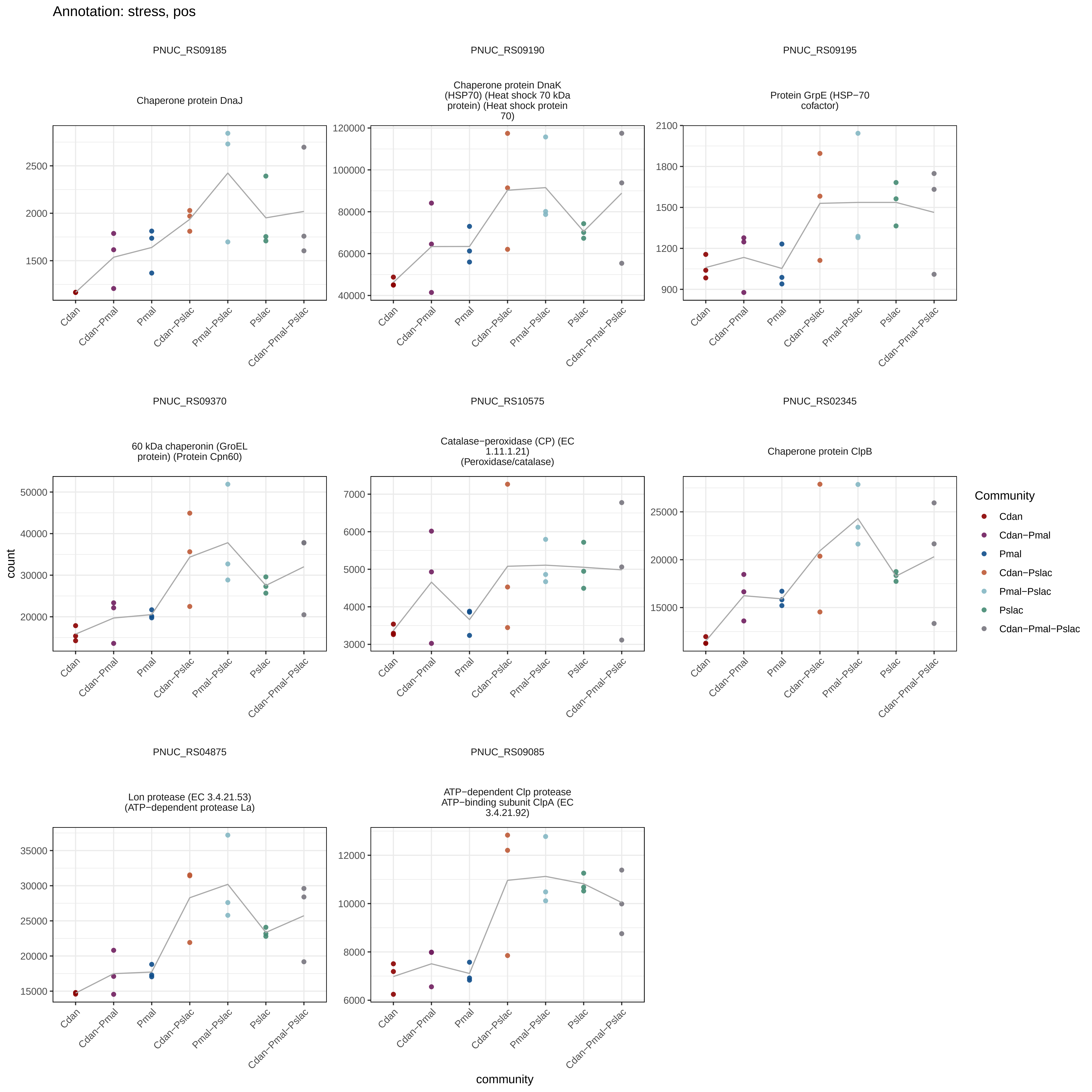
Annotation: stress, pos
PNUC_RS09185
Chaperone protein DnaJ
PNUC_RS09190
Chaperone protein DnaK (HSP70) (Heat shock 70 kDa protein) (Heat shock protein 70)
PNUC_RS09195
Protein GrpE (HSP−70 cofactor)
PNUC_RS09370
60 kDa chaperonin (GroEL protein) (Protein Cpn60)
PNUC_RS10575
Catalase−peroxidase (CP) (EC 1.11.1.21) (Peroxidase/catalase)
PNUC_RS02345
Chaperone protein ClpB
PNUC_RS04875
Lon protease (EC 3.4.21.53) (ATP−dependent protease La)
PNUC_RS09085
ATP−dependent Clp protease ATP−binding subunit ClpA (EC 3.4.21.92)
count
community
Cdan
Cdan−Pmal
Pmal
Cdan−Pslac
Pmal−Pslac
Pslac
Cdan−Pmal−Pslac
Community
Cdan
Cdan−Pmal
Pmal
Cdan−Pslac
Pmal−Pslac
Pslac
Cdan−Pmal−Pslac

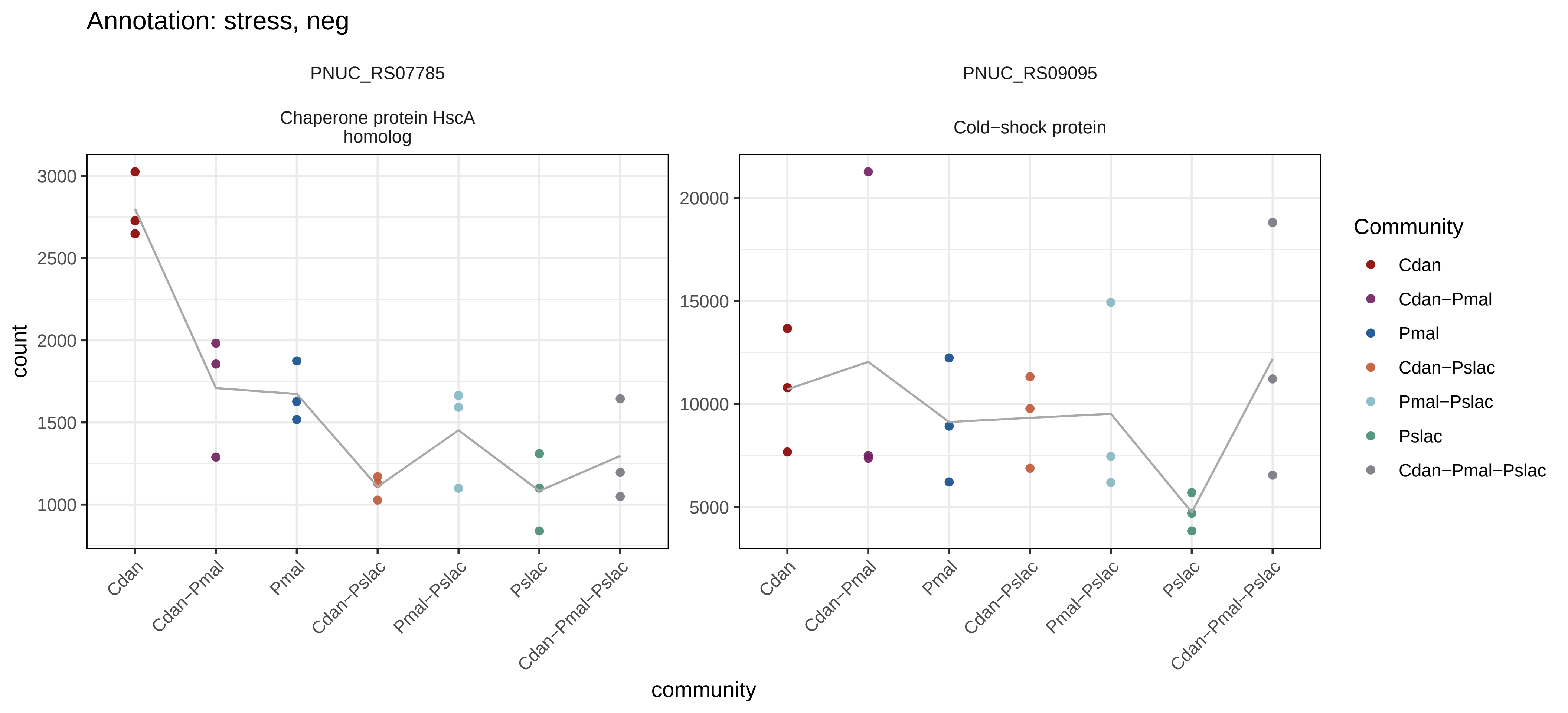

Annotation: stress, neg
PNUC_RS07785
Chaperone protein HscA homolog
PNUC_RS09095
Cold−shock protein
count
community
3000
2500
2000
1500
1000
20000
15000
10000
5000
Cdan
Cdan−Pmal
Pmal
Cdan−Pslac
Pmal−Pslac
Pslac
Cdan−Pmal−Pslac
Community
Cdan
Cdan−Pmal
Pmal
Cdan−Pslac
Pmal−Pslac
Pslac
Cdan−Pmal−Pslac

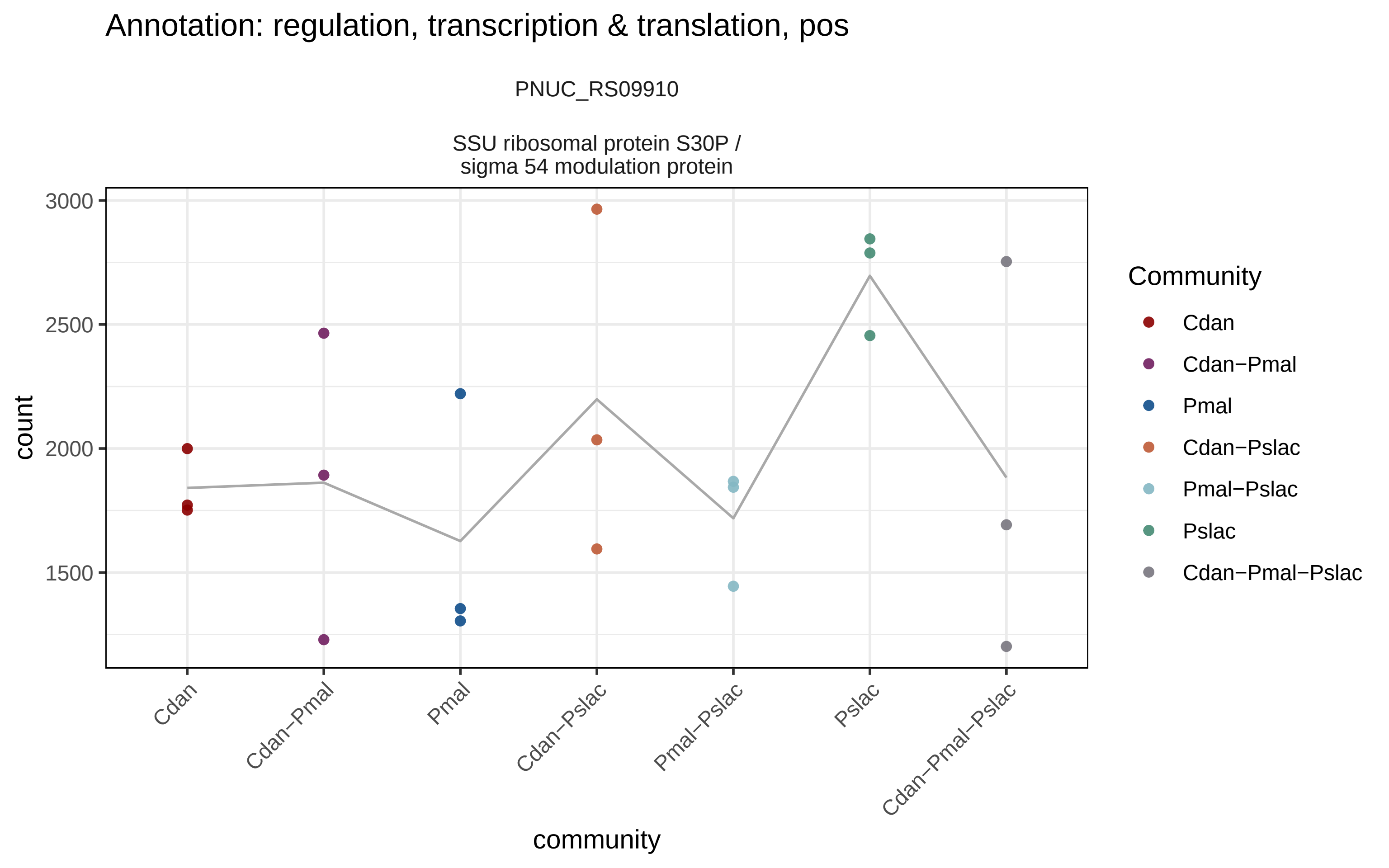
Annotation: regulation, transcription & translation, pos
PNUC_RS09910
SSU ribosomal protein S30P /
sigma 54 modulation protein
count
3000
2500
2000
1500
Cdan
Cdan−Pmal
Pmal
Cdan−Pslac
Pmal−Pslac
Pslac
Cdan−Pmal−Pslac
community
Community
Cdan
Cdan−Pmal
Pmal
Cdan−Pslac
Pmal−Pslac
Pslac
Cdan−Pmal−Pslac

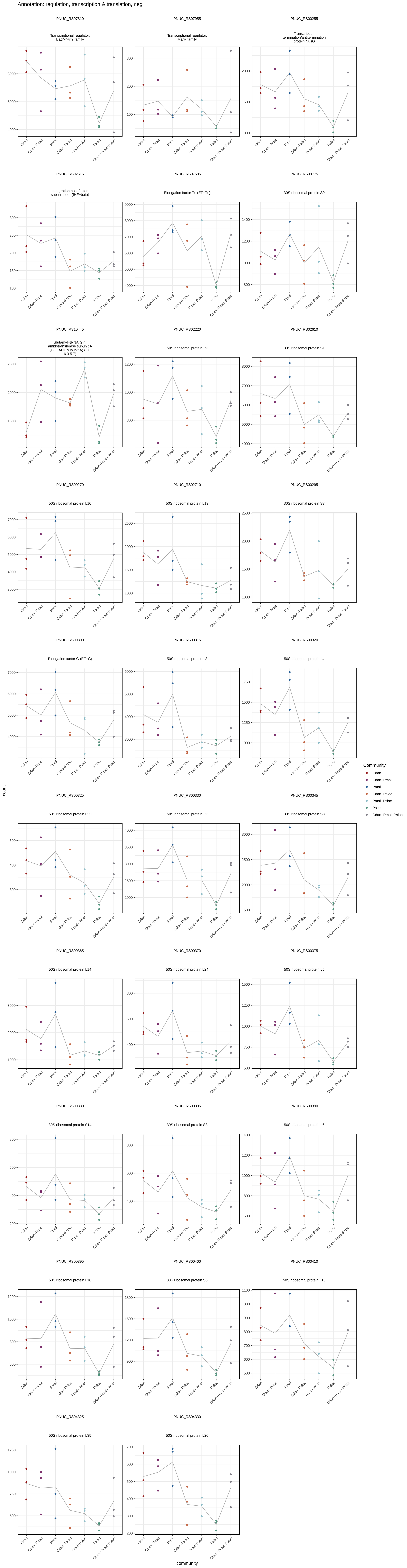
Annotation: regulation, transcription & translation, neg
PNUC_RS07810
Transcriptional regulator, BadM/Rrf2 family
PNUC_RS07955
Transcriptional regulator, MarR family
PNUC_RS00255
Transcription termination/antitermination protein NusG
PNUC_RS02615
Integration host factor subunit beta (IHF−beta)
PNUC_RS07585
Elongation factor Ts (EF−Ts)
PNUC_RS09775
30S ribosomal protein S9
PNUC_RS10445
Glutamyl−tRNA(Gln) amidotransferase subunit A (Glu−ADT subunit A) (EC 6.3.5.7)
PNUC_RS02220
50S ribosomal protein L9
PNUC_RS02610
30S ribosomal protein S1
PNUC_RS00270
50S ribosomal protein L10
PNUC_RS02710
50S ribosomal protein L19
PNUC_RS00295
30S ribosomal protein S7
PNUC_RS00300
Elongation factor G (EF−G)
PNUC_RS00315
50S ribosomal protein L3
PNUC_RS00320
50S ribosomal protein L4
PNUC_RS00325
50S ribosomal protein L23
PNUC_RS00330
50S ribosomal protein L2
PNUC_RS00345
30S ribosomal protein S3
PNUC_RS00365
50S ribosomal protein L14
PNUC_RS00370
50S ribosomal protein L24
PNUC_RS00375
50S ribosomal protein L5
PNUC_RS00380
30S ribosomal protein S14
PNUC_RS00385
30S ribosomal protein S8
PNUC_RS00390
50S ribosomal protein L6
PNUC_RS00395
50S ribosomal protein L18
PNUC_RS00400
30S ribosomal protein S5
PNUC_RS00410
50S ribosomal protein L15
PNUC_RS04325
50S ribosomal protein L35
PNUC_RS04330
50S ribosomal protein L20
Community
Cdan
Cdan−Pmal
Pmal
Cdan−Pslac
Pmal−Pslac
Pslac
Cdan−Pmal−Pslac
count
community

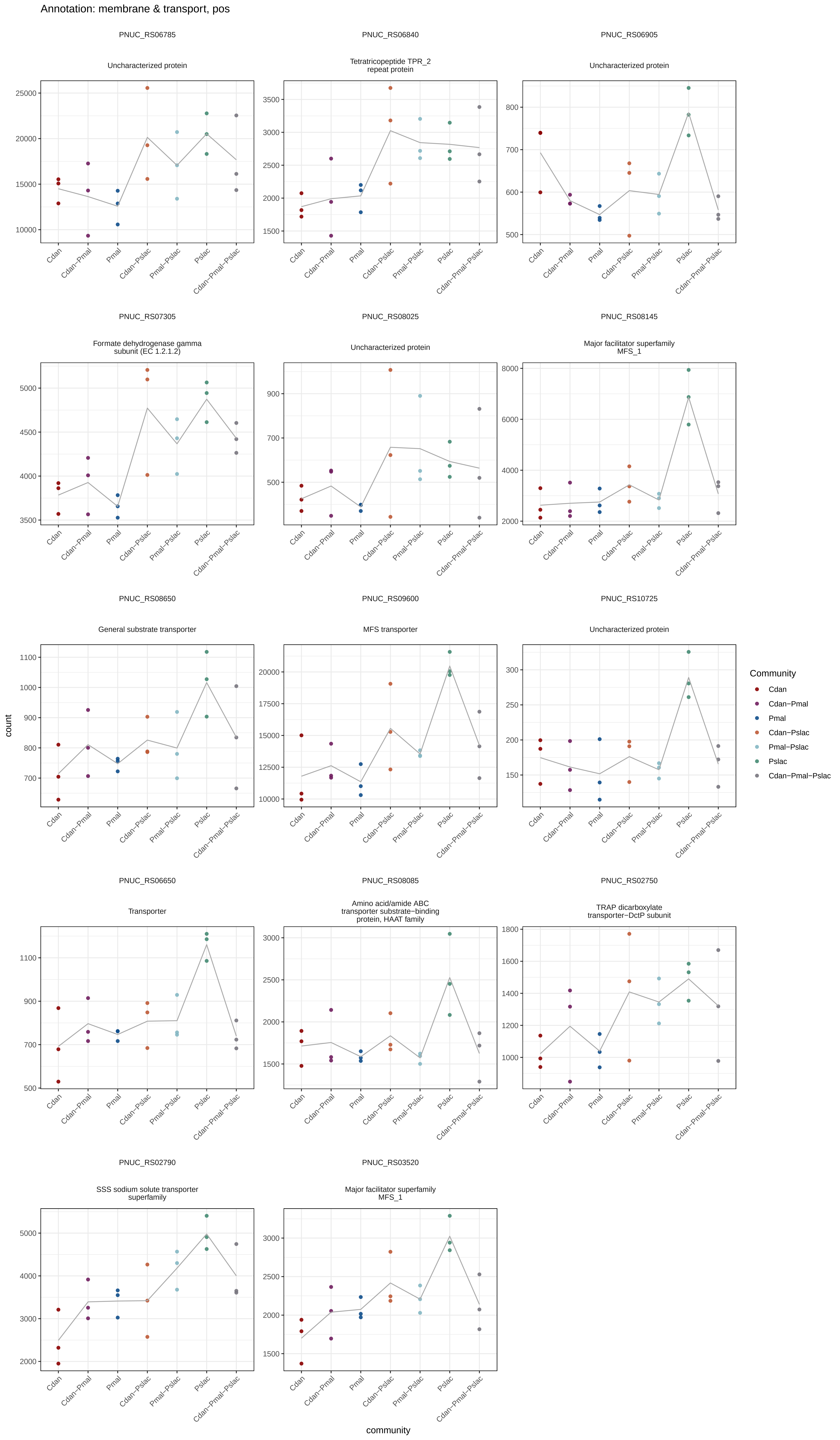
Annotation: membrane & transport, pos
PNUC_RS06785
Uncharacterized protein
PNUC_RS06840
Tetratricopeptide TPR_2 repeat protein
PNUC_RS06905
Uncharacterized protein
PNUC_RS07305
Formate dehydrogenase gamma subunit (EC 1.2.1.2)
PNUC_RS08025
Uncharacterized protein
PNUC_RS08145
Major facilitator superfamily MFS_1
PNUC_RS08650
General substrate transporter
PNUC_RS09600
MFS transporter
PNUC_RS10725
Uncharacterized protein
PNUC_RS06650
Transporter
PNUC_RS08085
Amino acid/amide ABC transporter substrate−binding protein, HAAT family
PNUC_RS02750
TRAP dicarboxylate transporter−DctP subunit
PNUC_RS02790
SSS sodium solute transporter superfamily
PNUC_RS03520
Major facilitator superfamily MFS_1
count
community
Cdan
Cdan−Pmal
Pmal
Cdan−Pslac
Pmal−Pslac
Pslac
Cdan−Pmal−Pslac
Community
Cdan
Cdan−Pmal
Pmal
Cdan−Pslac
Pmal−Pslac
Pslac
Cdan−Pmal−Pslac

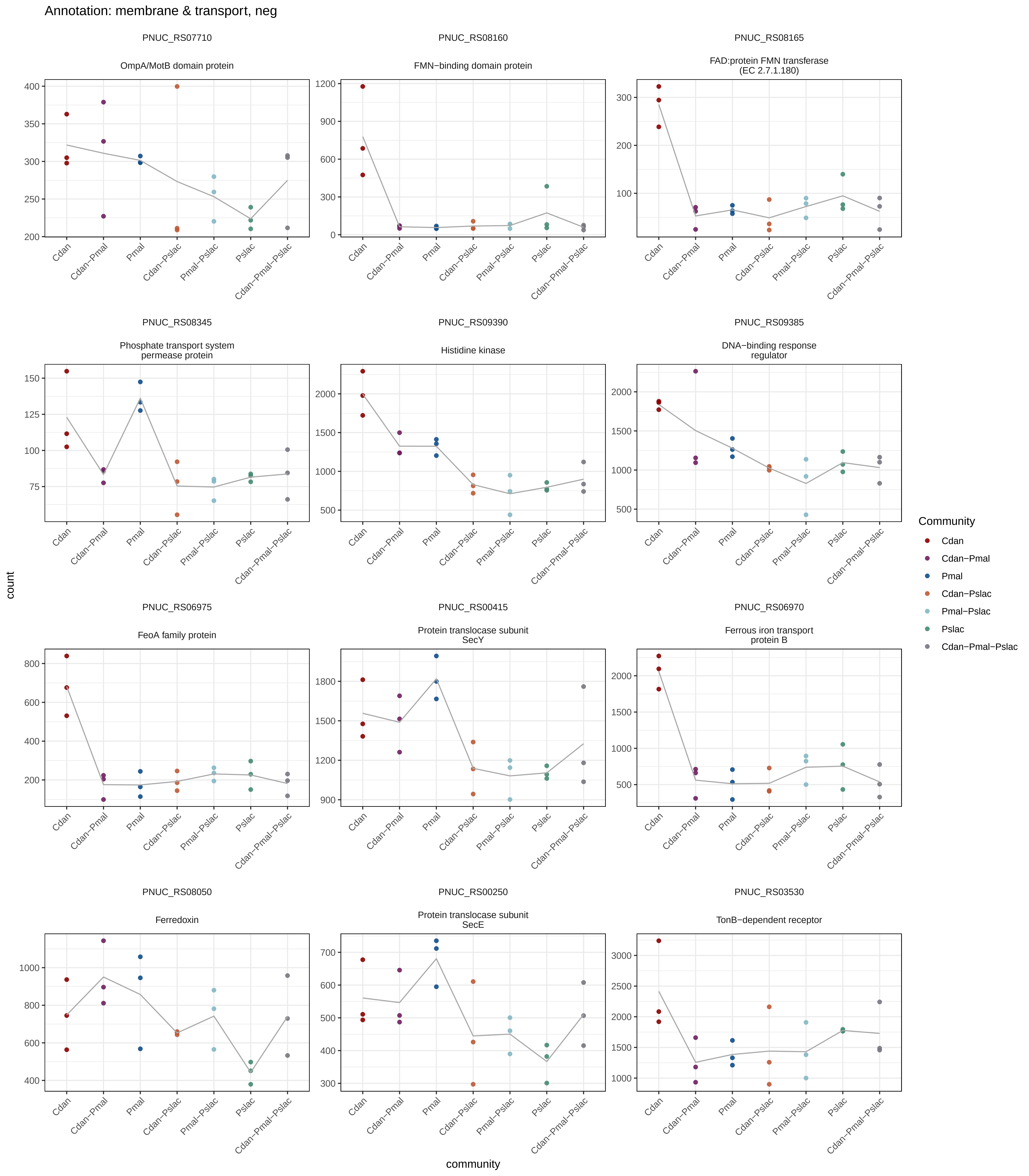

Annotation: membrane & transport, neg
PNUC_RS07710
OmpA/MotB domain protein
PNUC_RS08160
FMN−binding domain protein
PNUC_RS08165
FAD:protein FMN transferase (EC 2.7.1.180)
PNUC_RS08345
Phosphate transport system permease protein
PNUC_RS09390
Histidine kinase
PNUC_RS09385
DNA−binding response regulator
PNUC_RS06975
FeoA family protein
PNUC_RS00415
Protein translocase subunit SecY
PNUC_RS06970
Ferrous iron transport protein B
PNUC_RS08050
Ferredoxin
PNUC_RS00250
Protein translocase subunit SecE
PNUC_RS03530
TonB−dependent receptor
count
community
Cdan
Cdan−Pmal
Pmal
Cdan−Pslac
Pmal−Pslac
Pslac
Cdan−Pmal−Pslac
Community
